# Synthesis of Natural and Unnatural Cyclooligomeric Depsipeptides Enabled by Flow Chemistry

**DOI:** 10.1002/chem.201504457

**Published:** 2016-02-04

**Authors:** Daniel Lücke, Toryn Dalton, Steven V. Ley, Zoe E. Wilson

**Affiliations:** ^1^ Department of ChemistryUniversity of CambridgeLensfield RoadCambridgeCB2 1EWUK

**Keywords:** cyclooligomeric depsipeptides, flow chemistry, macrocycles, natural products, peptides

## Abstract

Flow chemistry has been successfully integrated into the synthesis of a series of cyclooligomeric depsipeptides of three different ring sizes including the natural products beauvericin (**1 a**), bassianolide (**2 b**) and enniatin C (**1 b**). A reliable flow chemistry protocol was established for the coupling and macrocyclisation to form challenging N‐methylated amides. This flexible approach has allowed the rapid synthesis of both natural and unnatural depsipeptides in high yields, enabling further exploration of their promising biological activity.

## Introduction

**Figure 1:**
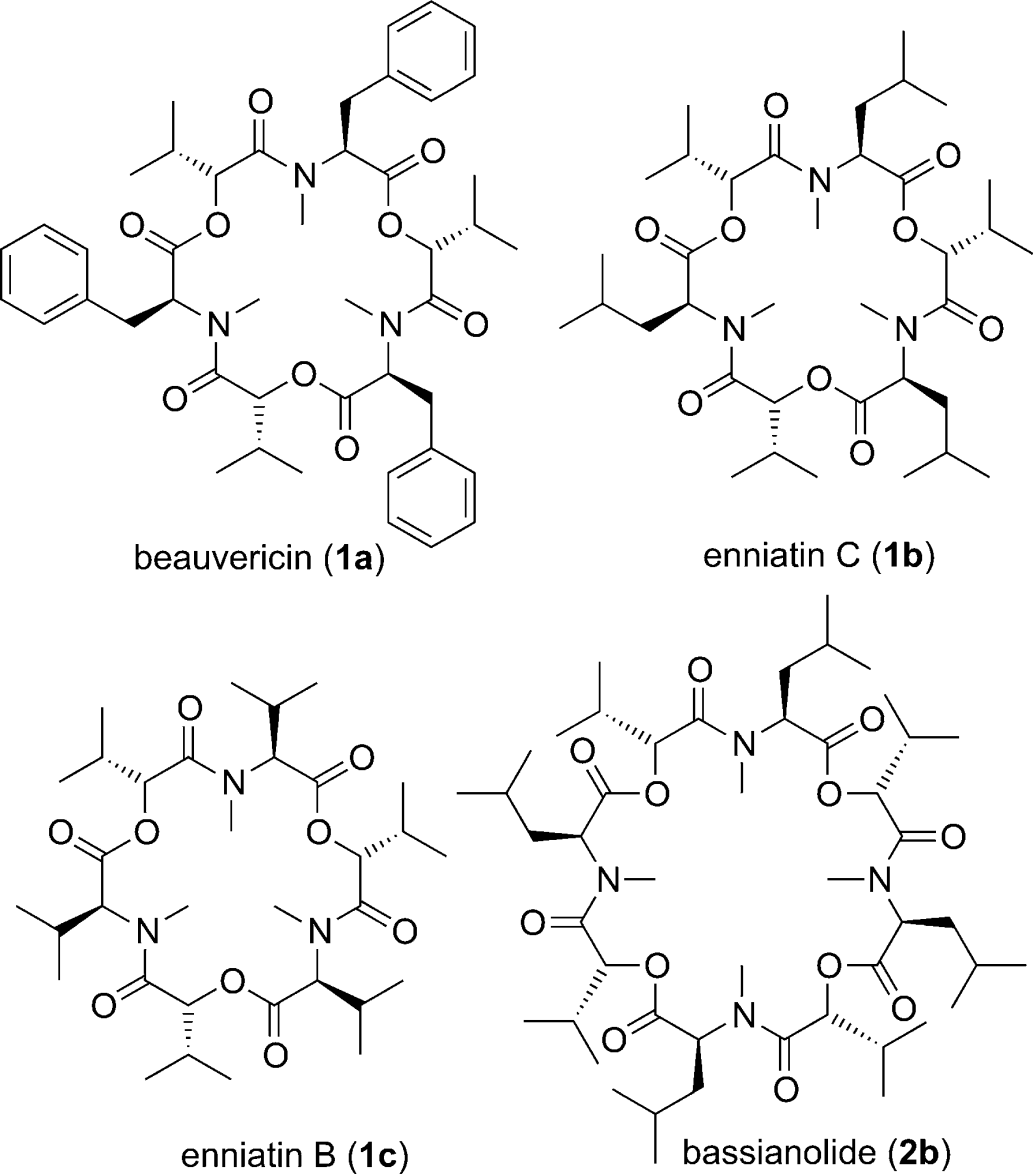
COD natural products beauvericin (**1 a**), enniatin C (**1 b**), enniatin B (**1 c**) and bassianolide (**2 b**).

Beauvericin (**1 a**), enniatin C (**1 b**) and bassianolide (**2 b**; Figure 1) are nonribosomal cyclooligomeric depsipeptides (CODs), with structures derived from repeated oligopeptidol monomer units consisting of d‐2‐hydroxyisovalerate (d‐Hiv) and N‐methylated amino acids joined head‐to‐tail in a macrocycle. Fungal CODs such as these are of particular interest because they exhibit a wide variety of biological activities, including as antibiotics or insecticides,^1^ and some are known for their ability to bind ions.^2^, 1

The structure of beauvericin (**1 a**), which consists of three repeated d‐Hiv‐l‐*N*‐Me‐Phe units, was first reported in 1969 from the fungus *Beauveria bassiana*. It exhibited toxicity towards brine shrimp and moderate inhibitory activity towards Gram‐positive bacteria, fungi and mosquito larvae.^3^ Beauvericin has subsequently been found to be produced by a range of *Fusarium* species^4^ and is a known mycotoxin, occurring naturally on corn, corn‐based foods and feeds which are infected by these fungi. Due to this, methods for the detection of **1 a** for food safety and its toxicity to normal human cells have been investigated.^5^

Bassianolide (**2 b**) was isolated in 1977 from *B. bassiana* and *Verticillium lecanii*—fungi isolated from silkworm pupae carcasses—and was shown to kill silkworm larvae by oral administration at dosage levels of more than 8 ppm, whereas beauvericin (**1 a**) had no effect by oral administration even at a dose of 1000 ppm. The structure was determined to be a COD consisting of four repeated d‐Hiv‐l‐*N*‐Me‐Leu units.^6^ A wide range of additional biological activities for both **1 a** and **2 b** have been reported since their initial isolation.^7^

Enniatin C (**1 b**), which consists of three repeated d‐Hiv‐l‐*N*‐Me‐Leu units, was first reported in 1968 when it was synthesised as an unnatural member of the enniatin family.^8^ It was also isolated by precursor‐directed biosynthesis using *Verticillium hemipterigenum* strain BCC 1449 in 2003.^9^ Enniatin C was subsequently found to be naturally produced by *V. hemipterigenum* BCC 1449 in 2004, albeit in very small amounts.^10^

The bioactivity of these compounds, particularly the fact that there are marked differences between the activities of different CODs, is of significant interest. This means that having robust synthetic access to these natural products, as well as the ability to make unnatural analogues, would enable further evaluation to potentially enhance desirable biological activity. Although directed biosynthesis has been used to successfully synthesise small libraries of beauvericin^11^ and enniatin^12^ analogues, the structures obtainable by this method are limited by the substrate scope of the synthase used.

Syntheses of the three natural product target molecules of this study have been previously reported, in all cases the peptide couplings were carried out via acid chloride intermediates. Ovchinnikov et al. reported a synthesis of enniatin C (**1 b**) in 1968^8^ and beauvericin (**1 a**) in 1971.^13^ Both syntheses used thionyl chloride as the chlorinating reagent, with **1 a** afforded in an overall yield of 9 %.^13^ The total synthesis of bassianolide (**2 b**) was reported soon after its isolation by Suzuki et al.^14^ This synthesis used PCl_5_ for the amide coupling steps and afforded **2 b** in overall 9.5 % yield, with the same route being applied to the synthesis of **1 b** to afford this molecule in 2.8 % overall yield.14b The practicality of acid chloride intermediates for peptide couplings has historically been constrained by their inherent over‐activation, which makes them prone to side reactions, especially with carboxybenzyl (Cbz) or *tert*‐butyloxycarbonyl (Boc) protection strategies, which combined with the unpleasant nature of the reagents needed for their synthesis has limited their application.^15^

Previous work in our group has established a solution phase synthesis for the 18‐membered COD enniatin B (**1 c**), which is the cyclic trimer of d‐Hiv‐l‐*N*‐Me‐Val units. This work used Ghosez reagent (1‐chloro‐*N*,*N*,2‐trimethyl‐1‐propenylamine),^16^ which allowed the formation of the required acid chloride intermediates under near neutral conditions and afforded **1 c** in a respectable overall yield of 15 %.^17^ The work herein reports the extension of this work to the synthesis of 12‐, 18‐ and 24‐membered CODs based on d‐Hiv‐l‐*N*‐Me‐Phe and d‐Hiv‐l‐*N*‐Me‐Leu monomer units, which includes the natural products beauvericin (**1 a**), enniatin C (**1 b**) and bassianolide (**2 b**), both in batch and with the integration of flow chemistry methods.

Although chemical synthesis is generally favoured to access peptides due to its inherent flexibility,^18^ existing methods are often highly inefficient, suffering from poor atom economy, challenging purifications and difficulties in scale‐up processes.^19^ The operational simplicity of solid phase peptide synthesis^20^ (SPPS) has led to it becoming widely regarded as the method of choice for peptide synthesis,^21^ however one of the major limitations of SPPS is its fundamental lack of atom economy. SPPS typically employs a four‐ to tenfold excess of building blocks to force reactions to completion, which is significant with costly monomer units, such as d‐Hiv and the related hydroxyester components needed to synthesise depsipeptides.^22^ Solid phase depsipeptide synthesis is also known to be problematic, with approaches often relying on using depsipeptide building blocks rather than forming the depsipeptide linkages on resin.^23^

After our group's previous work on peptide couplings in flow,^24^ cyclooligomeric depsipeptides were chosen as the targets for the current work as they exhibit highly desirable biological activity,^1^ contain challenging N‐methylated amide bonds,^25^ and could be built‐up iteratively as they consist of repeated dipeptidol monomer units. The accurate control of reaction parameters possible, potential for inline monitoring/automation and simplicity of scale‐up offered by flow chemistry give it exciting potential to solve the inherent problems with acid chloride mediated peptide synthesis,^15^ while in addition considerably reducing the labour involved in the synthesis through iterative protocols.

## Results and Discussion

### Batch synthesis of CODs

**Scheme 1:**
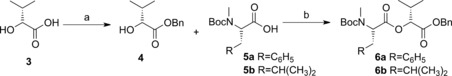
Synthesis of depsipeptide monomers. a) BnBr, Cs_2_CO_3_, DMF, 0 °C–RT, 15 h, 80 %; b) 4‐DMAP, EDCI, CH_2_Cl_2_, 0 °C–RT, 24 h, **6 a**=92 %, **6 b**=92 %.

Initially, attention focused on the batch synthesis of CODs with two to four dipeptidol units with *N*‐Me‐Phe and *N*‐Me‐Leu as the amino acid component, using the methods established in our group's previous synthesis of the related COD enniatin B (**1 c**).^17^ The synthesis began from d‐α‐hydroxyisovaleric acid (**3**), which is commercially available or could be accessed through methodology previously developed in Cambridge.^26^ The acid functionality was protected to give benzyl ester **4** which was coupled with either Boc‐*N*‐methyl‐phenylalanine (**5 a**) or Boc‐*N*‐methyl‐leucine (**5 b**) using 1‐ethyl‐3‐(3‐dimethylaminopropyl)carbodiimide hydrochloride (EDCI) and 4‐dimethylaminopyridine (4‐DMAP) to afford the dipeptidol building blocks for the two COD series, **6 a** and **6 b**, respectively, in high yields (Scheme 1).1

**Scheme 2:**
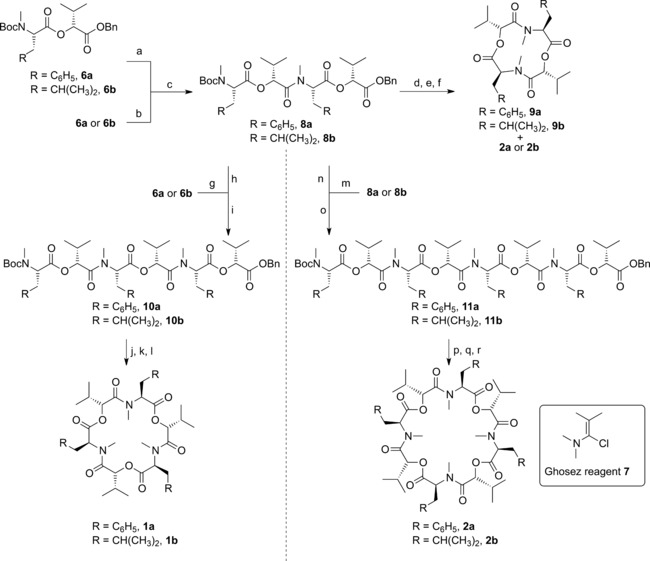
Batch synthesis of cyclooligomeric depsipeptides. a) 10 *%* Pd/C, H_2_, THF, RT, **a**: 2.5 h/**b**: 3 h; b) anhydrous HCl, dioxane, RT, 3 h; c) acid, Ghosez reagent **7**, CH_2_Cl_2_, 0 °C, **a**: 20 min/**b**: 25 min then *i*Pr_2_EtN, CH_2_Cl_2_, amine, 0 °C–RT, **a**: 17 h/**b**: 22 h, **8 a** *=*68 %, **8 b** *=*64 %; d) **a**: 10 *%* Pd/C, H_2_, THF, RT, 6 h, or **b**: H‐Cube® Pro, 10 % Pd/C CatCart®, CH_2_Cl_2_, 1 mL min^−1^, 60 *°C*, 6 bar; e) anhydrous HCl, dioxane, RT, **a**: 6 h/**b**: 5 h 25 min; f) Ghosez reagent **7**, CH_2_Cl_2_, 0 °C, **a**: 20 min/**b**: 15 min then *i*Pr_2_EtN, CH_2_Cl_2_, 0 °C–RT, **a**: 21 h/**b**: 20 h, **9 a** *=*17 % and **2 a** *=*41 %, **9 b** *=*19 % and **2 b** *=*45 %; g) 10 % Pd/C, H_2_, THF, RT, **a**: 2 h 30 min/**b**: 3 h 15 min; h) anhydrous HCl, dioxane, RT, 6 h; i) acid, Ghosez reagent **7**, CH_2_Cl_2_, 0 °C, 20 min then *i*Pr_2_EtN, CH_2_Cl_2_, amine, 0 °C–RT, 18 h, **10 a** *=*75 %, **10 b** *=*78 %; j) **a**: 10 % Pd/C, H_2_, THF, RT, 6 h, or **b**: H‐Cube® Pro, 10 % Pd/C CatCart®, CH_2_Cl_2_, 1 mL min^−1^, 60 °C, 6 bar; k) anhydrous HCl, dioxane, RT, 6 h; l) Ghosez reagent **7**, CH_2_Cl_2_, 0 °C, 30 min then *i*Pr_2_EtN, CH_2_Cl_2_, 0 °C–RT, **a**: 18 h 30 min/**b**: 18 h, **1 a** *=*70 %, **1 b** *=*42 %; m) 10 % Pd/C, H_2_, THF, RT, **a**: 6 h/**b**: 5 h 30 min; n) anhydrous HCl, dioxane, RT, **a**: 6 h/**b**: 5 h; o) acid, Ghosez reagent **7**, CH_2_Cl_2_, 0 °C, **a**: 20 min/**b**: 25 min then *i*Pr_2_EtN, CH_2_Cl_2_, amine, 0 °C–RT, **a**: 20 h 30 min/**b**: 16 h, **11 a** *=*57 %, **11 b** *=*75 %; p) **a**: H‐Cube® Pro, 10 % Pd/C CatCart®, MeOH/CH_2_Cl_2_, 1 mL min^−1^, 60 °C, 6 bar or **b**: 10 % Pd/C, H_2_, THF, RT, 6 h; q) anhydrous HCl, dioxane, RT, **a**: 6 h 50 min/**b**: 5 h; r) Ghosez reagent **7**, CH_2_Cl_2_, 0 °C, **a**: 20 min/**b**: 30 min then *i*Pr_2_EtN, CH_2_Cl_2_, 0 °C–RT, **a**: 19 h 30 min/**b**: 18 h, **2 a** *=*30 %, **2 b** *=*67 %.

Iterative deprotection and coupling steps were then used to assemble linear COD precursors of two (**8**), three (**10**) and four (**11**) dipeptidol units (Scheme 2). The amines were Boc deprotected using anhydrous hydrochloric acid (HCl) in dioxane and the benzyl protection removed from the acids by hydrogenation with palladium on charcoal, with both deprotected products able to be used directly in the coupling without further purification. The deprotected coupling partners were coupled via the acid chloride intermediate using Ghosez reagent^16^ in dichloromethane in the presence of diisopropylethylamine. Ghosez reagent was chosen as the coupling reagent as it is fully soluble and is therefore suitable for use in flow, where precipitates can be problematic. This provided access to all six target linear precursors by one (**8**) or two coupling steps (**10** and **11**) in yields (for the combined deprotections and coupling) ranging from 57–78 %. Attention then turned to the often challenging macrocyclisations. Pleasingly it was found that after the global deprotection of the linear precursors, using the established conditions, Ghosez reagent could be successfully employed to macrocyclise intermediates of all three sizes to form 12, 18 and 24 atom rings. Beauvericin (**1 a**), bassianolide (**2 b**) and enniatin C (**1 b**) were successfully synthesised in overall yields of 26, 24 and 15 %, respectively (Scheme 2). These overall yields are significant improvements upon those achieved previously.^13^, ^14^ While the synthesis of 12‐membered ring macrocycles was found to be possible, it was seen that the conditions afforded modest yields of the desired ring size (17 % for **9 a** and 19 % for **9 b**), accompanied with higher yields of the product resulting from an intermolecular dimerization occurring prior to macrocylisation (41 % of **2 a** and 45 % of bassianolide **2 b**). While this represents a shortened approach for the synthesis of bassianolide (**2 b**) and **2 a**, this was less desirable if access to the 12‐membered rings was the aim.2

**Figure 2:**
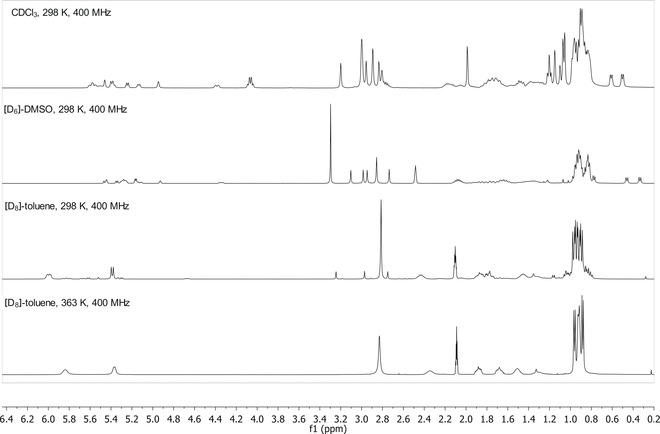
Effect of solvent and temperature on ^1^H NMR of bassianolide (**2 b**).

Room temperature ^1^H and ^13^C NMR spectroscopy showed that the 12‐ (**9 a** and **9 b**) and 18‐ (**1 a** and **1 b**) membered CODs existed in one symmetrical conformation in chloroform. In comparison, the 24‐membered CODs (**2 a** and **2 b**) existed as a mixture of conformers in chloroform, dimethylsulfoxide and toluene at room temperature. Heating to 363 K in toluene resulted in the ^1^H and ^13^C NMR spectra coalescing to a single spectrum where all the dipeptidol units were in the same environment (Figure 2).2

### Flow integrated synthesis

With the batch synthesis of the six target CODs accomplished, attention turned to establishing whether it was possible to improve these syntheses by the intelligent incorporation of flow technology. Work focused on the iterative peptide couplings, since the batch methods to produce dipeptidol starting materials **6 a** and **6 b** were high yielding and easily scalable so further optimisation was not considered to be necessary.

**Scheme 3:**
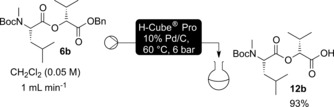
Test flow deprotection of **6 b** using standard conditions.

First it was desirable to establish whether the deprotection steps could be carried out in flow to allow potential telescoping of the steps. Benzyl protection had been selected for the carboxy terminus as this allows removal of the ester by simple hydrogenation. Flow hydrogenation has precedent using an H‐Cube® flow hydrogenator.^27^ Using dipeptidol **6 b** as a test substrate, it was seen that hydrogenation in dichloromethane using a 10 % palladium on carbon CatCart® afforded acid **12 b** in good yield (Scheme 3). As the solvent was routinely removed from the reaction mixture before being taken up in an exact volume of solvent for the next step, in some cases methanol was used as solvent as it was less prone to evaporation than dichloromethane.3

Attention next focused on the Boc deprotection in flow (Table 1), with which we have experience both using heterogeneous (catch‐and‐release) and homogenous methods.^28^ As a starting point a stream of the dipeptidol **6 b** in dichloromethane was combined with a stream of trifluoroacetic acid (TFA) and the solvent was removed from the outflow in vacuo which resulted in only 11 % conversion to **13 b**. Increasing the amount of TFA and the reaction time led to improved conversion (88 %); when anhydrous HCl in dioxane (17 equivalents) was used as the acid it was found that conversion decreased to 18 %. Returning to TFA as the acid, increasing both the flow rate and coil length, overall decreasing the reaction time to 24 min, improved the conversion to 94 %. Decreasing the reaction time further to 10 min gave a slight reduction in conversion to 90 %, but it was found that raising the reaction temperature to 35 °C could compensate for this (93 %). Further increase of the temperature to 40 °C afforded 97 % conversion. Increasing the reaction time at this temperature to 20, 30 and 40 min only resulted in a slight improvement in conversion to 98 %, and increasing the amount of TFA to 1

**Table 1:** Optimisation of the flow Boc deprotection.

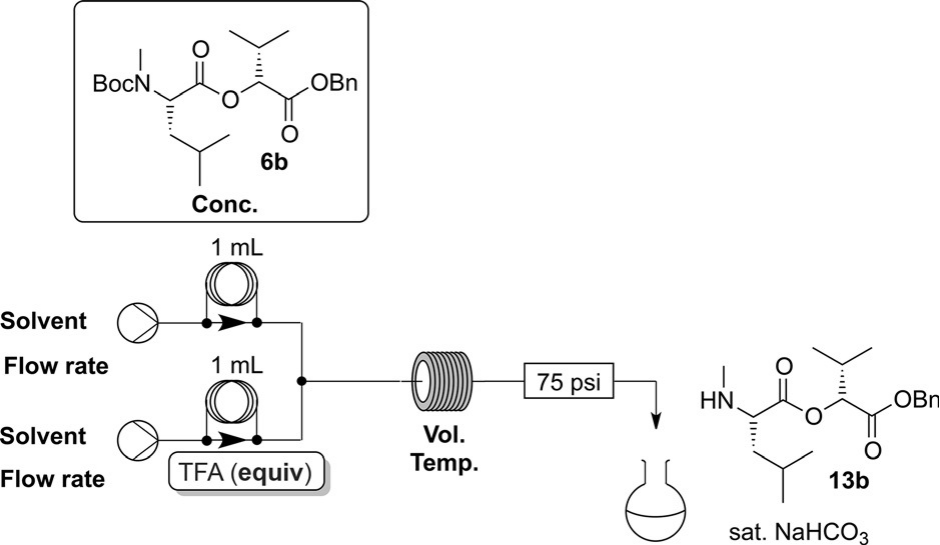											
	Entry	Acid	Equiv	Solvent	Flow rate per pump [mL min^−1^]	Conc. [m]	Vol. [mL]	Reaction time [min]	*T* [°C]	Conv. [%]^[a]^	
	1	TFA	1	CH_2_Cl_2_	0.100	0.04	0	1	RT	11	
	2	TFA	2	CH_2_Cl_2_	0.500	0.10	10	10	RT	19	
	3	TFA	20	CH_2_Cl_2_	0.100	0.04	0	1	RT	43	
	4	TFA	20	CH_2_Cl_2_	0.033	0.10	2	30	RT	88	
	5	HCl	17	1,4‐dioxane	0.033	0.10	2	30	RT	18	
	6	TFA	20	CH_2_Cl_2_	0.333	0.10	16	24	RT	94	
	7	TFA	20	CH_2_Cl_2_	0.500	0.10	10	10	RT	90	
	8	TFA	20	CH_2_Cl_2_	0.500	0.10	10	10	35	93	
	9	TFA	20	CH_2_Cl_2_	0.500	0.10	10	10	40	97	
	10	TFA	20	CH_2_Cl_2_	0.250	0.10	10	20	40	98	
	11	TFA	20	CH_2_Cl_2_	0.167	0.10	10	30	40	98	
	12	TFA	20	CH_2_Cl_2_	0.125	0.10	10	40	40	98	
	13	TFA	40	CH_2_Cl_2_	0.125	0.10	10	40	40	98	

[a] Conversion was determined using ^1^H NMR spectroscopy of crude reaction mixtures.

Although this work established that the flow deprotection of the secondary amine was possible it was considered that the batch Boc deprotection was operationally simpler and that flow offered no significant benefit. Therefore, it was decided to continue to use the established batch process for this work.

Although the batch conditions had provided an idea of an appropriate starting point for optimisation of the peptide coupling steps, it was deemed prudent to first optimise the coupling step using a model peptide coupling rather than wasting the synthetically derived dipeptidol units. The coupling of Bocleucine (**14**) with phenylalanine methyl ester⋅HCl (**15**) was chosen as a test system for this reaction as the coupling partners were readily available, but this reaction had been found to be challenging using acid chloride methods, with a yield of only 14 % resulting when the batch coupling protocol from the depsipeptide synthesis was applied to these coupling partners (Table 2, entry 1). It has been reported that Boc‐protected amino acids are not compatible with acid‐chloride‐mediated peptide couplings due to their tendency to self‐cyclise to form reactive *N*‐carboxyanhydrides^15^ which could be contributing to this low yield.2

**Table 2:** Optimisation of reaction conditions using model coupling.

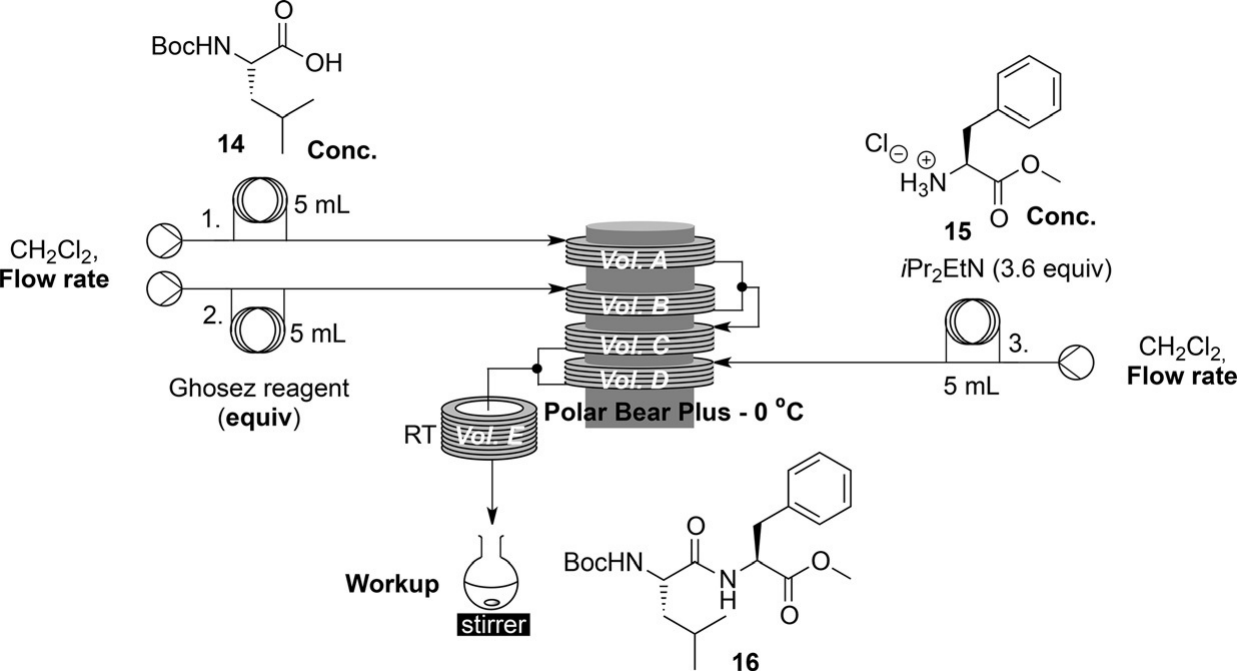														
Entry	Flow rate per	Scale	Conc.	*T*	Activation	Vol. [mL]					Equiv. Ghosez	Reaction	Workup	Yield
	pump [mL min^−1^]	[mmol]	[m]	solvent	time [min]	A	B	C	D	E	reagent	time [min]		[%]
1	batch	0.50	0.10	–	20	–	–	–	–	–	1	930	RT/1 m HCl	14
2	0.50	0.50	0.10	RT	3	2.5	2.5	3	3	10	1	6.67	RT/1 m HCl	3
3	0.50	0.50	0.10	RT	3	2.5	2.5	3	3	10	2	6.67	RT/1 m HCl	19
4	0.50	0.50	0.10	0 °C	3	2.5	2.5	3	3	10	2	6.67	RT/1 m HCl	18
5	0.25	0.50	0.10	RT	6	2.5	2.5	3	3	10	2	13.3	RT/1 m HCl	11
6	1.00	0.50	0.10	RT	1.5	2.5	2.5	3	3	10	2	3.33	RT/1 m HCl	12
7	1.00	0.50	0.10	RT	1.5	2.5	2.5	3	3	20	2	6.67	RT/1 m HCl	11
8	0.50	0.50	0.10	RT	3	2.5	2.5	3	3	20	2	13.3	RT/1 m HCl	18
9	1.00	0.50	0.10	RT	5	3	3	10	2.5	20	2	6.67	RT/1 m HCl	19
10	1.00	0.50	0.10	RT	5	3	3	10	2.5	20	2	6.67	0 °C/1 m HCl	33
11	1.00	0.50	0.10	RT	5	3	3	10	2.5	20	2	6.67	0 °C/sat. NH_4_Cl	31
12	1.00	0.50	0.10	RT	5	3	3	10	2.5	20	2	6.67	0 °C/H_2_O	28
13	1.00	0.10	0.02	RT	5	3	3	10	2.5	20	2	6.67	0 °C/1 m HCl	44
14	1.00	0.05	0.01	RT	5	3	3	10	2.5	20	2	6.67	0 °C/1 m HCl	46
15	batch	0.05	0.01	–	5	–	–	–	–	–	2	6.67	0 °C/1 m HCl	46
16	batch	0.60	0.01	–	5	–	–	–	–	–	2	6.67	0 °C/1 m HCl	23
17	1.00	0.60^[a]^	0.01	RT	5	3	3	10	2.5	20	2	6.67	0 °C/1 m HCl	47

[a] Reaction was run continuously for 1 h after reaching steady state

Previous work in our laboratories^29^ has shown that the stacking of reactor coils on a cryo‐cooling device can allow reagent streams to be pre‐cooled, with excellent results. It was envisaged that this would allow the reaction of a pre‐cooled solution of acid coupling partner with a pre‐cooled solution of Ghosez reagent to allow highly controlled generation of the required acid chlorides at low temperature, which could immediately be treated with a pre‐cooled solution of the amine coupling partner and base to affect coupling with 1:1 stoichiometry. The precise control of stoichiometry and temperature this process affords would prevent exotherms and minimise the possible side reactions of the acid chloride intermediate. It was a specific aim of the project to avoid conditions requiring an excess of either coupling partner, due to the ultimate aim of coupling more complex, and therefore expensive, coupling partners. Once the third stream had been added, the coupling reaction could be allowed to proceed at room temperature, before the output is collected in a quenching solution and worked up.

Reactions were optimised (Table 2) using plug flow, with Sudan Red dye first being used to determine the necessary injection times so that the streams met, in a similar fashion to that used previously.29a The initial set‐up used a single equivalent of Ghosez reagent and flow rates of 0.50 mL min^−1^ for each pump, resulting in a reaction time for the formation of the acid chloride at 0 °C of 3 min and the coupling was carried out at room temperature with a reaction time of 6 min 40 s, resulting in a 3 % isolated yield of the desired product. Doubling the equivalents of Ghosez reagent increased the yield to 19 %, an improvement over the yield achieved for this coupling in batch. As there were concerns over whether the Ghosez reagent was decomposing before it reached the cooled reaction coils, next the solvent reservoirs were cooled to 0 °C. This was seen to have negligible effect on the yield of the reaction, so routinely solvents were not cooled. Both decreasing and increasing the flow rate resulted in a decrease in the yield, whilst increasing the coupling time by increasing the volume of coil E had no appreciable effect on yield, indicating that the acid chloride formation time rather than the coupling reaction time was limiting yield. This was supported by the fact that when the volume of coil C was increased to give a 5 min acid chloride formation time at the 1 mL min^−1^ flow rate, the yield for this reaction increased to 19 %, even with the coupling reaction time being only 6 min 40 s. Attention next focused on the quenching of the reaction mixture. It was found that if the output stream was quenched with stirring aqueous HCl at 0 °C rather than at room temperature that there was a significant increase in yield to 33 %. Using saturated ammonium chloride or water at 0 °C resulted in slightly reduced yields. It was found that diluting the coupling partners to 0.02 or 0.01 m significantly improved the yield of the reaction to 44 and 46 %, respectively.

With the reaction significantly optimised from the original conditions, it was then desirable^30^ to determine whether the observed increase in yield was due to “flow effects” or if it was simply that the ease of screening conditions in flow had allowed us to better optimise this reaction. In order to do this, batch conditions were employed which mimicked the optimised flow conditions as closely as possible and it was seen that the batch yield was identical to when that coupling was carried out in flow, although it should be noted that the flow process was considerably simpler operationally. To investigate whether scale was a factor for this reaction, the reaction was carried out both in batch and in flow on 0.6 mmol scale. When this reaction was carried out in batch the increased volume resulted in considerable complications, with addition of the two 60 mL solutions by cannula taking over 4 min each, and the yield dropping significantly to 23 %. For the flow reaction, reaction mixtures of appropriate concentration were run continuously for 1 h after being allowed to reach what we expect to be steady state, based on the anticipated rapid kinetics of the process (1.5 reactor volumes, 30 min).^30^ Pleasingly, the continuous flow yield was comparable to the plug flow yield (47 %), highlighting one of the key benefits of flow chemistry—its ease of scale‐up.

With conditions established for the coupling and deprotections attention turned to the application of this to the synthesis of the target cyclooligomeric depsipeptides. The flow integrated synthesis of the depsipeptides began with the intermolecular couplings to give the necessary linear precursors for macrocyclisation. The appropriate coupling partners were deprotected either by flow hydrogenation to afford the acid or by batch removal of the Boc group with anhydrous HCl in dioxane to afford the HCl salt of the amine. These crude products were then taken up in solvent and coupled using the optimised flow conditions.

Pleasingly, it was seen that the couplings proceeded in improved yields for all linear precursors with the average yield for these couplings increasing from 70 % in batch to 80 % in flow (Table 3).3

**Table 3:** Flow synthesis of cyclooligomeric depsipeptide precursors.

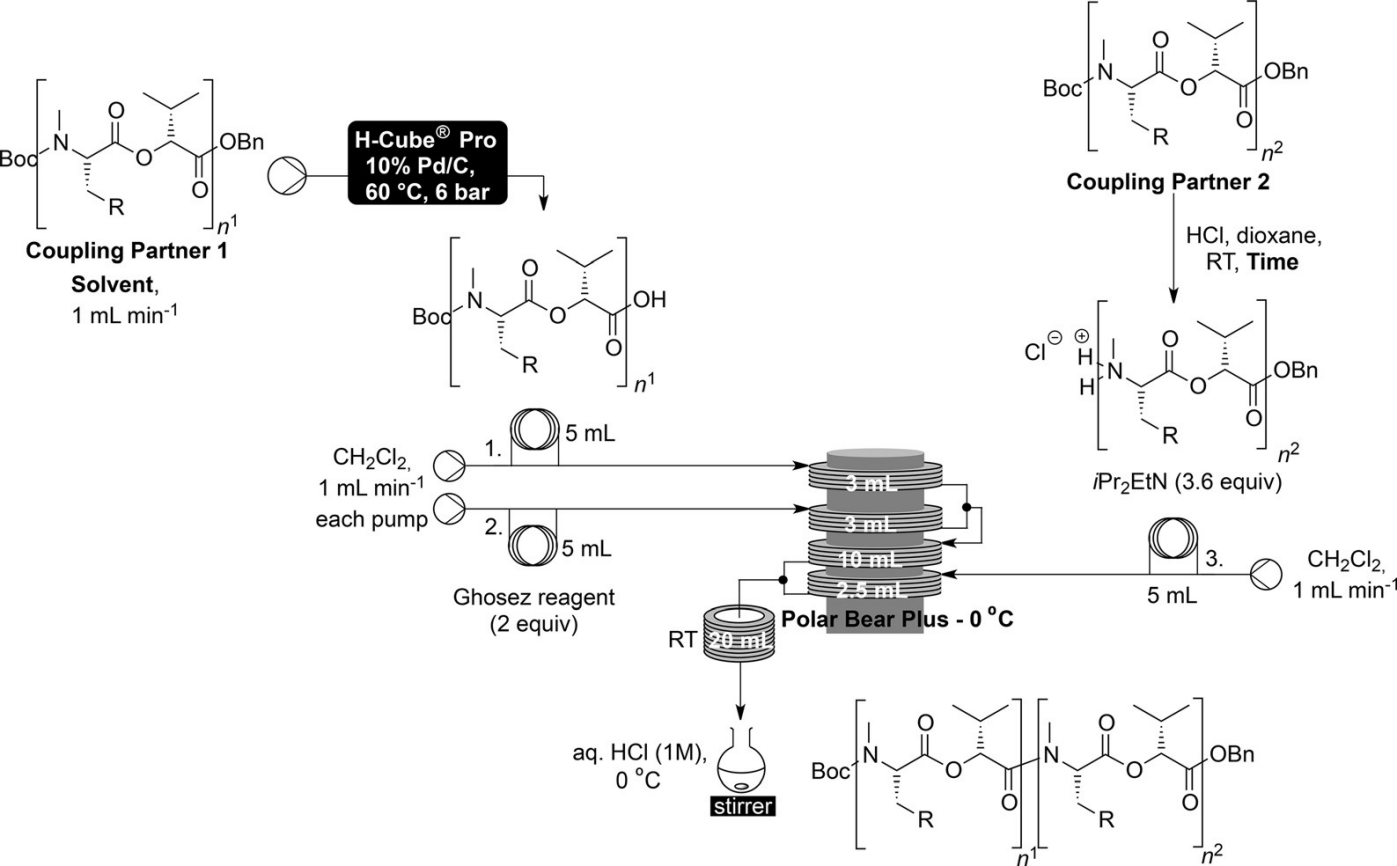									
Entry	Product	R	Coupling partner 1		Coupling partner 2		Solvent	Time	Yield
			#	*n* ^1^	#	*n* ^2^			[%]
1	**8 a**	C_6_H_5_	**6 a**	1	**6 a**	1	CH_2_Cl_2_	3 h	82
2	**10 a**	C_6_H_5_	**6 a**	1	**10 a**	2	CH_2_Cl_2_	6 h	78
3	**11 a**	C_6_H_5_	**10 a**	2	**10 a**	2	CH_2_Cl_2_	6 h	67
4	**8 b**	CH(CH_3_)_2_	**6 b**	1	**6 b**	1	MeOH	5 h	90
5	**10 b**	CH(CH_3_)_2_	**6 b**	1	**10 b**	2	MeOH	7 h 10 min	86
6	**11 b**	CH(CH_3_)_2_	**10 b**	2	**10 b**	2	MeOH	5 h 30 min	76

With the linear precursors in hand attention turned to the intramolecular macrocyclisation. Both termini were deprotected as before and in order to simplify the setup, rather than injecting the base as a third plug the third stream was run continuously as a 0.036 m solution of diisopropylethylamine (3.6 equivalents) in dichloromethane (Table 4). The macrocyclisation in flow was found to be very effective—affording all three size rings for each dipeptidol monomer in significantly improved yield from that achieved in batch—with the average yield going from 41 % in batch to 81 % in flow. Especially notable was the improvement in the macrocyclisation of **8 a** and **8 b** to give the 12‐membered cyclooligomeric depsipeptides **9 a** and **9 b**, respectively. Whereas in batch significantly more of the product resulting from the dimerization then macrocyclisation to give 24‐membered rings (**2**) was formed than the desired 12‐membered products (**9**), the dilution and precise temperature control of the optimised flow conditions resulted in high yields of the 12‐membered rings being synthesised without any detectable formation of the 24‐membered rings.4

**Table 4:** Flow macrocyclisation.

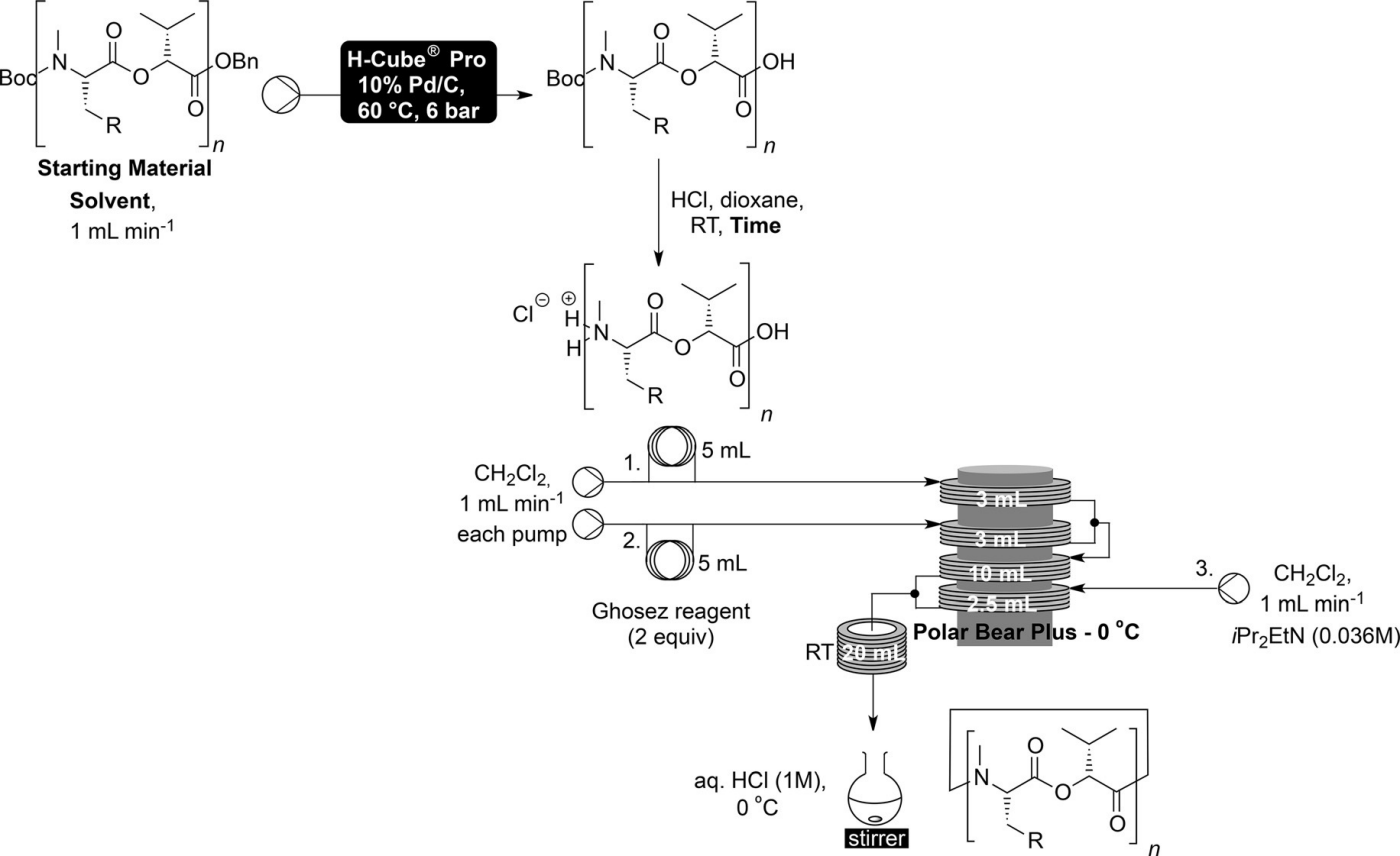							
Entry	Product	R	Starting material		Solvent	Time	Yield
			#	*n*			[%]
1	**9 a**	C_6_H_5_	**8 a**	2	CH_2_Cl_2_	6 h	68
2	**1 a**	C_6_H_5_	**10 a**	3	CH_2_Cl_2_	6 h	76
3	**2 a**	C_6_H_5_	**11 a**	4	CH_2_Cl_2_/MeOH (2 mL/5 mL)	6 h 25 min	80
4	**9 b**	CH(CH_3_)_2_	**8 b**	2	MeOH	5 h 35 min	86
5	**1 b**	CH(CH_3_)_2_	**10 b**	3	MeOH	6 h	84
6	**2 b**	CH(CH_3_)_2_	**11 b**	4	MeOH	6 h 5 min	93

## Conclusions

**Figure 3:**
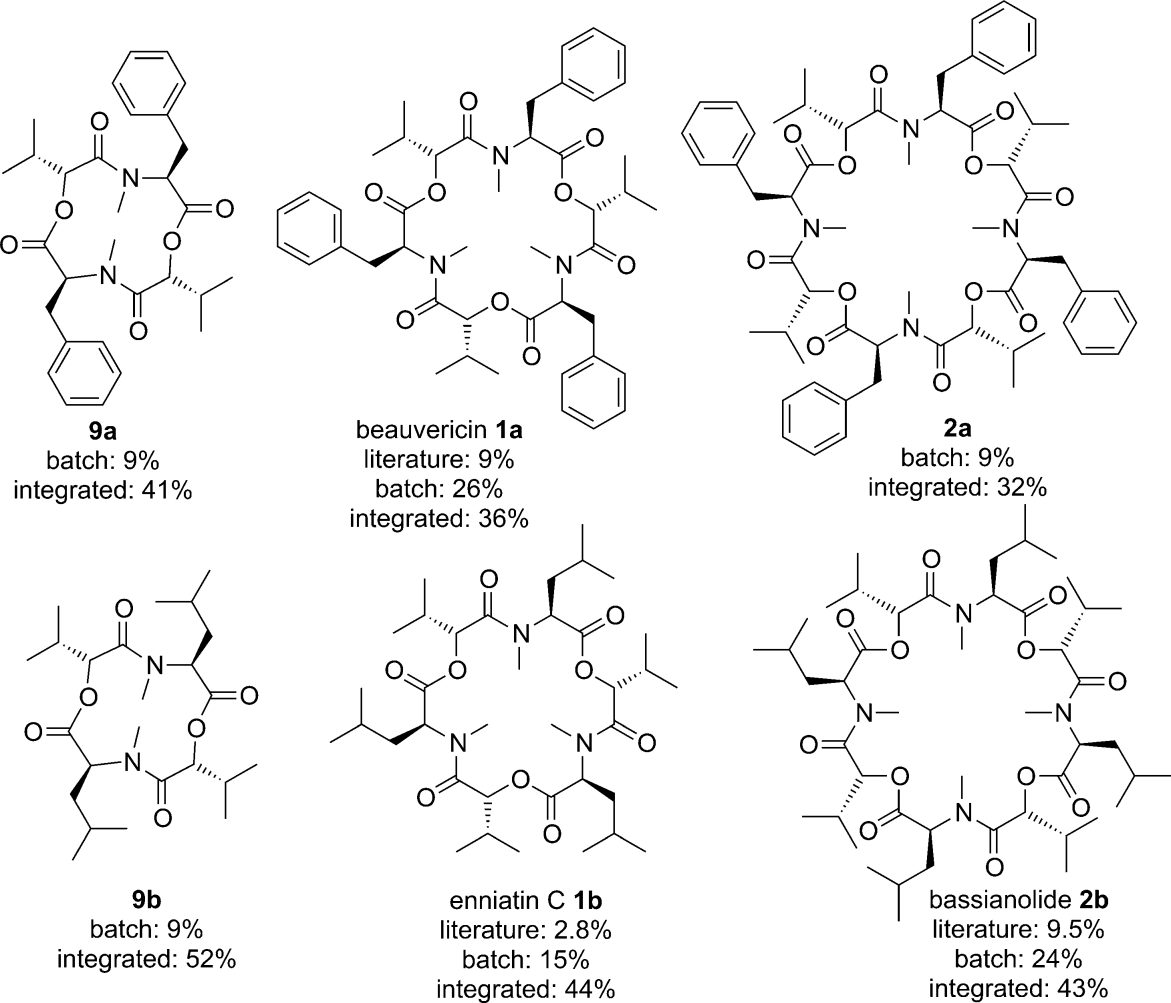
Overall yields for literature,^13^, ^14^ batch and the flow integrated syntheses of the target CODs.

The batch synthesis of a series of natural and unnatural CODs was successfully accomplished in useful overall yields. The informed use of flow chemistry in the synthesis of the target CODs significantly increased the yields for the library of compounds (Figure 3), with the overall yields improving by 10–43 % from the batch process. The optimised flow conditions allowed inter‐ and intramolecular peptide couplings with considerable reduction in effort for the chemist when compared to the batch methods, additionally creating the possibility for their future automation. The successful synthesis of the six CODs offers the opportunity to further explore the bioactivity of this family of compounds and this synthetic approach would equally be suitable for the rapid synthesis of further unnatural analogues of these molecules. Notably, the flow peptide couplings were effected with rapid overall reaction times and high yields being achieved in an atom efficient manner, with 1:1 stoichiometry of coupling partners being used. These results suggest that for other peptide syntheses the use of solution phase couplings combined with flow methods may prove attractive, particularly during scale‐up.3

## Experimental Section

### General methods

All reactions were carried out under argon atmosphere using oven‐dried glassware, and were monitored by TLC. Unless otherwise stated, reagents were obtained from commercial sources and used without further purification. Amino acids were all of the natural (l) enantiomeric form unless otherwise stated. Solvents were freshly distilled over calcium hydride and lithium aluminium hydride (tetrahydrofuran and diethyl ether) or calcium hydride (dichloromethane, methanol, toluene, ethyl acetate and 40–60 petroleum ether). Additional anhydrous solvents were obtained from commercial sources and used directly (*N,N‐*dimethylformamide).

Flow reactions were carried out using Vapourtec R2+^31^ and Syrris Asia^32^ pumps and a Polar Bear Plus cooling unit (Cambridge Reactor Design).^33^

Thin layer chromatography (TLC) was carried out using 0.25 mm thick glass backed Merck TLC Silica gel 60 F254 plates which were visualised using ultraviolet radiation and aqueous acidic ammonium molybdate (VII) solution or potassium permanganate solution. Preparatory TLC was carried out using Analtech 20×20 cm UNIPLATE Silica gel GF (preparative layer with UV254) plates of either 500, 1000 or 2000 microns depth and were visualised using UV. Flash column chromatography was carried out using high‐purity grade silica gel (Merck grade 9385) with a pore size 60 Å and 230–400 mesh particle size. Infrared spectroscopy was recorded using a PerkinElmer Spectrum One FT‐IR spectrometer using Universal ATR sampling accessories. Absorbance measurements were recorded in the range 4000–650 cm^−1^. NMR spectra were recorded using either a 400 MHz DPX‐400 Dual spectrometer, a 500 MHz AV III HD Smart Probe spectrometer or a 600 MHz Avance 600 BBI spectrometer as indicated. Unless otherwise stated, all samples were run at room temperature in deuterated solvent, with chemical shift (*δ*) reported to the nearest 0.01 (^1^H)/0.1 ppm (^13^C), relative to the residual protic solvent; *δ*(CDCl_3_)=7.26 (^1^H)/77.16 ppm (^13^C); *δ*([D_8_]‐toluene)=2.09, 6.98, 7.00, 7.09 (^1^H)/20.40, 125.49, 128.33, 129.24, 137.86 ppm (^13^C) or *δ*([D_6_]‐DMSO) 2.50 (^1^H)/39.51 ppm (^13^C). All carbon NMRs were run with broadband proton decoupling. Multiplicity of a signal in ^1^H NMR spectra is indicated by: s=singlet, d=doublet, t=triplet or, q=quartet, quint=quintet, m=multiplet, or a combination thereof. Multiplets are reported as the range of ppm values covered by the signals, otherwise the centre of the signal is given. Coupling constants (*J*) are quoted in Hz and recorded to the nearest 0.1 Hz. HRMS was performed using either a Waters Micromass LCT Premier spectrometer or by using a Bruker Bioapex 47e FTICR spectrometer, using positive ESI+. Masses are given in *m*/*z* units. Melting points (m.p.) were measured using a Stanford Research Systems OptiMelt automated melting point system using a gradient of 1 °C min^−1^, and are uncorrected. Specific optical rotation was recorded on a Perkin–Elmer Model 343 digital polarimeter, using a Na/Hal lamp set at 589 nm and with a path‐length of 100 mm. All [*α*_D_] values were measured using spectroscopy grade solvent at the specified concentration [g cm^−3^] and temperature, with units of 10^−1^ cm^2^ g^−1^. Additional data related to this publication is available at the University of Cambridge Institutional Data Repository (https://www.repository.cam.ac.uk/handle/1810/252536).

### Synthesis and characterisation

**d‐Hiv‐OBn (4)**: Caesium carbonate (4.45 g, 13.7 mmol) was added to a solution of d‐hydroxyisovaleric acid (**3**; 3.22 g, 27.3 mmol) in *N*,*N*‐dimethylformamide (20 mL) at 0 °C and the resulting mixture was stirred for 40 min. Benzyl bromide (3.6 mL, 30.0 mmol) was added and the reaction mixture was stirred at room temperature for 15 h. The reaction mixture was filtered and the filtrate was diluted with 40–60 petroleum ether/ethyl acetate (4:1, 120 mL). The organic layer was washed with saturated ammonium chloride solution (100 mL), saturated sodium bicarbonate (NaHCO_3_) solution (100 mL) and saturated sodium chloride (NaCl) solution (100 mL) before being dried over magnesium sulfate (MgSO_4_) and the solvent being removed in vacuo. The crude product was purified by flash column chromatography using 40–60 petroleum ether/ethyl acetate (10:1) as eluent to afford the title compound **4** (4.56 g, 21.9 mmol, 80 %) as a colourless oil. The ^1^H and ^13^C data were in agreement with that reported in the literature.^34^ *R*_f_=0.29 [40–60 petroleum ether/diethyl ether=10:1]; [$\alpha_{D}^{25.8}$ ]=+17.8 (*c* 3.35 EtOH) literature;^35^ [$\alpha_{D}^{20}$ ]=+15.4 (*c* 2.1, EtOH); ^1^H NMR (400 MHz, [D_6_]DMSO): *δ*=7.49–7.26 (m, 5 H, C1′c*H*–C1′e*H*), 5.32 (br s., 1 H, C2O*H*), 5.25–5.02 (m, 2 H, C1′a*H_2_*), 3.86 (d, *J*=5.0 Hz, 1 H, C2*H*), 2.04–1.81 (m, 1 H, C3*H*), 0.87 (d, *J*=6.9 Hz, 3 H, C4*H*_3_), 0.81 ppm (d, *J*=6.9 Hz, 3 H, C4*H*_3_); ^13^C NMR (151 MHz, [D_6_]‐DMSO): *δ*=172.5 (quat., *C*1), 135.6 (quat., *C*1′b), 127.6, 127.5, 127.2 (5×CH, *C*1′c–*C*1′e), 74.7 (CH_2_, *C*1′a), 64.8 (CH, *C*2), 31.1 (CH, *C*3), 17.7 (CH_3_, *C*4), 16.3 ppm (CH_3_, *C*4).

**Boc‐Me‐Phe‐d‐Hiv‐OBn (6 a)**: EDCI (1.72 g, 9.0 mmol) was added to a solution of d‐Hiv‐OBn (**4**; 1.30 g, 6.3 mmol), Boc‐*N*‐methyl‐l‐phenylalanine (**5 a**; 2.50 g, 8.95 mmol) and 4‐DMAP (0.99 g, 8.1 mmol) in dichloromethane (25 mL) at 0 °C and the resulting mixture was stirred at room temperature for 14.5 h. The reaction was ended by the addition of aqueous HCl (25 mL, 1 m) and the phases were separated. The aqueous layer was extracted with dichloromethane (3×30 mL) and the combined organic layers were washed with water (100 mL), saturated NaHCO_3_ solution (100 mL) and saturated NaCl solution (100 mL) before being dried over MgSO_4_ and the solvent being removed in vacuo. The crude product was purified by flash column chromatography using 40–60 petroleum ether/ethyl acetate (10:1) as eluent to afford the title compound **6 a** (2.69 g, 5.7 mmol, 92 %) as a colourless oil. *R*_f_=0.34 [40–60 petroleum ether/diethyl ether=10:1]; [$\alpha_{D}^{23.7}$ ]=−30.9 (*c* 1.00, CHCl_3_); ^1^H NMR (400 MHz, [D_6_]‐DMSO, *T*=403 K): *δ*=7.47–7.13 (m, 10 H, d‐Hiv‐C1′c*H*–d‐Hiv‐C1′e*H* and Phe‐C5*H*–Phe‐C7*H*), 5.20 (s, 2 H, d‐Hiv‐C1′a*H*_2_), 4.99 (dd, *J*=10.4, 5.4 Hz, 1 H, Phe‐C2*H*), 4.92 (d, *J*=4.6 Hz, 1 H, d‐Hiv‐C2*H*), 3.23 (dd, *J*=14.6, 5.4 Hz, 1 H, Phe‐C3*H*), 3.01 (dd, *J*=14.6, 10.4 Hz, 1 H, Phe‐C3*H*), 2.70 (s, 3 H, Phe‐NC*H*_3_), 2.28–2.13 (m, 1 H, d‐Hiv‐C3*H*), 1.33 (s, 9 H, Phe‐C2′c*H*_3_), 0.96 (d, *J*=6.9 Hz, 3 H, d‐Hiv‐C4*H*_3_), 0.93 ppm (d, *J*=6.9 Hz, 3 H, d‐Hiv‐C4*H*_3_); ^13^C NMR (101 MHz, [D_6_]‐DMSO, *T*=403 K): *δ*=169.6 (quat., Phe‐*C*1), 167.8 (quat., d‐Hiv‐*C*1), 155.5 (quat., Phe‐*C*2′a), 136.8 (quat., Phe‐*C*4), 135.0 (quat., d‐Hiv‐*C*1′b), 128.0, 127.7, 127.4, 127.3, 125.6 (10×CH, d‐Hiv‐*C*1′c–d‐Hiv‐*C*1′e, Phe‐*C*5–Phe‐*C*7), 78.7 (quat., Phe‐*C*2′b), 76.4 (CH, d‐Hiv‐*C*2), 65.7 (CH_2_, d‐Hiv‐*C*1′a), 58.8 (CH, Phe‐*C*2), 33.8 (CH_2_, Phe‐*C*3), 30.2 (CH_3_, Phe‐N*C*), 29.1 (CH, d‐Hiv‐*C*3), 27.3 (3×CH_3_, Phe‐*C*2′c), 17.5 (CH_3_, d‐Hiv‐*C*4), 16.2 ppm (CH_3_, d‐Hiv‐*C*4); IR (neat): $\tilde{\nu }$ =2972, 2933, 2880, 1741, 1694, 1455, 1390, 1366, 1167, 1127, 1029, 750, 697 cm^−1^; HRMS (ESI): found 470.2531 [*M*+H]^+^ C_27_H_36_NO_6_ requires 470.2543 Δ=2.4 ppm.

**Synthesis of Boc‐Me‐Phe‐d‐Hiv‐Me‐Phe‐d‐Hiv‐OBn (8 a) [batch]**: Anhydrous HCl (2.5 mL, 4 m in dioxane) was added to a solution of Boc‐Me‐Phe‐d‐Hiv‐OBn (**6 a**; 0.31 g, 0.66 mmol) in dioxane (2.5 mL) at room temperature and the resulting mixture was stirred for 3 h. The solvent was removed in vacuo and the residue was taken up sequentially in ethanol (2×5 mL) and methanol (5 mL) with the solvent removed in vacuo after each addition to afford the amine HCl salt, which was used in the next step without further purification.

Hydrogen gas was bubbled through a solution of Boc‐Me‐Phe‐d‐Hiv‐OBn (**6 a**; 0.31 g, 0.66 mmol) and palladium (70 mg, 10 % on charcoal, 0.07 mmol) in tetrahydrofuran (5 mL) for 3 min at room temperature before the resulting mixture was stirred under a hydrogen atmosphere for 2.5 h. The reaction mixture was filtered through Celite, eluting with ethyl acetate (20 mL). The solvent was removed in vacuo from the filtrate to afford the acid which was used in the next step without further purification.

Ghosez reagent (0.096 mL, 0.72 mmol) was added to a solution of the crude acid in dichloromethane (2.5 mL) at 0 °C and the resulting mixture was stirred for 20 min at this temperature. A solution of the crude amine HCl salt and *N*,*N*‐diisopropylethylamine (0.40 mL, 2.36 mmol) in dichloromethane (2.5 mL) was added and the reaction mixture was allowed to warm to room temperature and stirred for 17 h. Aqueous HCl (20 mL, 1 m) was added and the phases were separated. The aqueous layer was extracted with dichloromethane (3×20 mL) and the combined organic layers were washed with water (40 mL), saturated NaHCO_3_ solution (40 mL) and saturated NaCl solution (40 mL) before drying over MgSO_4_ and removing the solvent in vacuo. The crude product was purified by flash column chromatography using 40–60 petroleum ether/ethyl acetate (10:1) as eluent to afford the title compound **8 a** (0.33 g, 0.45 mmol, 68 %) as a colourless solid. *R*_f_=0.06 [40–60 petroleum ether/diethyl ether=10:1]; m.p.=120–122 °C; [$\alpha_{D}^{23.7}$ ]=−47.4 (*c* 1.00, CHCl_3_); ^1^H NMR (400 MHz, [D_6_]‐DMSO, *T*=403 K): *δ*=7.51–7.31 (m, 5 H, d‐Hiv^B^‐C1′c*H*–d‐Hiv^B^‐C1′e*H*), 7.31–7.08 (m, 10 H, Phe^A+B^‐C5*H*–Phe^A+B^‐C7*H*), 5.39 (br s, 1 H, Phe^B^‐C2*H*), 5.21 (d, *J*=1.4 Hz, 2 H, d‐Hiv^B^‐C1′a*H_2_*), 5.11 (d, *J*=4.8 Hz, 1 H, d‐Hiv^A^‐C2*H*), 4.99–4.90 (m, 2 H, Phe^A^‐C2*H* and d‐Hiv^B^‐C2*H*), 3.32 (dd, *J*=14.6, 5.4 Hz, 1 H, Phe^B^‐C3*H*H), 3.21 (dd, *J*=14.6, 5.2 Hz, 1 H, Phe^A^‐C3*H*H), 3.07–2.96 (m, 2H Phe^A^‐C3H*H* and Phe^B^‐C3H*H*), 2.94 (s, 3 H, Phe^B^‐NC*H_3_*), 2.72 (s, 3 H, Phe^A^‐NC*H_3_*), 2.22 (pd, *J*=6.9, 4.8 Hz, 1 H, d‐Hiv^B^‐C3*H*), 1.81 (br. s, 1 H, d‐Hiv^A^‐C3*H*), 1.31 (s, 9 H, 3×Phe^A^‐C2′c*H_3_*), 0.95 (dd, *J*=6.9, 4.3 Hz, 6 H, 2×d‐Hiv^B^‐C4*H_3_*), 0.78 (d, *J*=6.8 Hz, 3 H, d‐Hiv^A^‐C4*H_3_*), 0.71–0.55 ppm (m, 3 H, d‐Hiv^A^‐C4*H_3_*); ^13^C NMR (101 MHz, [D_6_]‐DMSO, *T*=403 K): *δ*=169.3 (quat., Phe^A^‐*C*1), 169.1 (quat., Phe^B^‐*C*1), 168.3 (quat., d‐Hiv^A^‐*C*1), 167.8 (quat., d‐Hiv^B^‐*C*1), 154.1 (quat., Phe^A^‐*C*2′a), 136.9 (quat., Phe^A^‐*C*4), 136.4 (quat., Phe^B^‐*C*4), 135.0 (quat., d‐Hiv^B^‐*C*1′b), 128.1, 128.0, 127.7, 127.5, 127.4, 127.4, 125.7, 125.6 (15×CH, Phe^A+B^‐*C*5–Phe^A+B^‐*C*7 and d‐Hiv^B^‐*C*1′c–d‐Hiv^B^‐*C*1′e), 78.5 (quat., Phe^A^‐*C*2′b), 76.7 (CH, d‐Hiv^B^‐*C*2), 74.4 (CH, d‐Hiv^A^‐*C*2), 65.7 (CH_2_, d‐Hiv^B^‐*C*1′a), 58.7 (CH, Phe^A^‐*C*2), 57.5 (CH, Phe^B^‐*C*2), 33.9 (CH_2_, Phe^B^‐*C*3), 33.6 (CH_2_, Phe^A^‐*C*3), 31.0 (CH_3_, Phe^B^‐N*C*), 30.0 (CH_3_, Phe ^A^‐N*C*), 29.0 (CH, d‐Hiv^B^‐*C*3), 28.6 (CH, d‐Hiv^A^‐*C*3), 27.3 (3×CH_3_, Phe^A^‐*C*2′c), 17.8 (CH_3_, d‐Hiv^A^‐*C*4), 17.4 (CH_3_, d‐Hiv^B^‐*C*4), 16.3 (CH_3_, d‐Hiv^B^‐*C*4), 15.7 ppm (CH_3_, d‐Hiv^A^‐*C*4); IR (neat): $\tilde{\nu }$ =2962, 2937, 1761, 1737, 1683, 1662, 1454, 1394, 1290, 1167, 1128, 1029, 745, 696 cm^−1^; HRMS (ESI): found 731.3878 [*M*+H]^+^ C_42_H_55_N_2_O_9_ requires 731.3907 Δ=3.9 ppm.

**Synthesis of Boc‐Me‐Phe‐d‐Hiv‐Me‐Phe‐d‐Hiv‐OBn (8 a) [flow]**: Dipeptidol **6 a** (0.024 g, 0.05 mmol) was taken up in dichloromethane and this solution was hydrogenated using an H‐Cube® Pro (ThalesNano) with a 10 % Pd/C CatCart®. The pump was run at 1 mL min^−1^ using dichloromethane with the temperature set to 60 °C and the pressure to 6 bar. The solvent was then removed in vacuo and the crude acid was used directly in the coupling.

Dipeptidol **6 a** (0.024 g, 0.05 mmol) was taken up in dioxane (1 mL) and anhydrous HCl (1 mL, 4 m in dioxane) was added and the reaction mixture stirred at room temperature for 3 h. The solvent was then removed in vacuo and the crude oil was taken up in ethanol (2×2 mL) and methanol (2 mL) successively, removing the solvent in vacuo after each addition to afford the amine as the HCl salt, which was used directly in the coupling.

The flow equipment was set up according to Table 3. The crude acid was taken up in dichloromethane (5 mL) and filled into loop 1. Ghosez reagent (0.013 mL, 0.1 mmol) was taken up in dichloromethane (5 mL) and filled into loop 2. The crude amine HCl salt and diisopropylethylamine (0.032 mL, 0.18 mmol) were taken up in dichloromethane (4.968 mL) and filled into loop 3. Pumps 1–3 were run a 1 mL min^−1^ with dichloromethane and injection loops 1–3 injected at the appropriate time to ensure coordinated meeting of streams at the T‐pieces (determined by runs using Sudan red dye): loop 1 at *t*=0, loop 2 at *t*=23 s and loop 3 at *t*=4 min 53 s. From *t*=15 min to *t*=26 min the reaction outflow was collected in stirring aqueous HCl (10 mL, 1 m) at 0 °C. The reaction mixture was extracted with dichloromethane (3×20 mL) and the combined organic extracts were washed with water (10 mL), saturated NaHCO_3_ (10 mL) and saturated NaCl (10 mL) before being dried over MgSO_4_ and the solvent being removed in vacuo. The resultant crude product was then purified using preparative TLC using 40–60 petroleum ether/ethyl acetate (2:1) as eluent to afford the title compound **8 a** (0.031 g, 0.042 mmol, 82 %) as a colourless solid, which was spectroscopically identical to that reported using method A.

**Synthesis of beauvericin (1 a) [batch]**: Hydrogen gas was bubbled through a solution of dipeptidol trimer **10 a** (83 mg, 84 μmol) and palladium (9 mg, 10 % on charcoal, 8.4 μmol) in tetrahydrofuran (2 mL) for 3 min at room temperature before the mixture was stirred under hydrogen for 6 h. The reaction mixture was filtered through Celite and washed with ethyl acetate (30 mL). The solvent was removed in vacuo from the filtrate to afford the acid, which was then taken up in dioxane (2 mL). Anhydrous HCl (2 mL, 4 m in dioxane) was added and the resulting mixture was stirred for 6 h. The solvent was removed in vacuo and the residue was taken up sequentially in ethanol (2×5 mL) and methanol (5 mL) removing the solvent in vacuo after each addition to afford the deprotected linear precursor. This was taken up in dichloromethane (20 mL) and cooled to 0 °C before Ghosez reagent (12 μL, 92 μmol) was added at and the resulting mixture was stirred for 30 min at this temperature. *N*,*N*‐Diisopropylethylamine (51 μL, 301 μmol) was added and the reaction mixture was warmed to room temperature and stirred for 18.5 h. The solvent was removed in vacuo and the residue was taken up in diethyl ether (60 mL) and aqueous HCl (60 mL, 1 m). The aqueous layer was extracted with diethyl ether (3×30 mL) and the combined organic layers were washed with water (100 mL), saturated NaHCO_3_ solution (100 mL) and saturated NaCl solution (100 mL) before being dried over MgSO_4_ and the solvent being removed in vacuo. The crude product was purified by flash column chromatography using 40–60 petroleum ether/ethyl acetate (2:1→1:1) as eluent to afford the title compound **1 a** (46 mg, 59 μmol, 70 %) as a colourless solid. The ^1^H and ^13^C NMR spectra were in agreement with that published in the literature.^36^ *R*_f_=0.15 [40–60 petroleum ether/diethyl ether=1:1]; m.p.=92–93 °C (literature=92–93 °C^36^); [$\alpha_{D}^{24.7}$ ]=+75 (*c* 0.1 MeOH) literature;^13^ [$\alpha_{D}^{20}$ ]=+69 (*c* 0.1, MeOH); ^1^H NMR (600 MHz, CDCl_3_): *δ*=7.41–7.07 (m, 5 H, Phe‐C5*H*–Phe‐C7*H*), 5.46 (dd, *J*=11.5, 4.9 Hz, 1 H, Phe‐C2*H*), 4.92 (d, *J*=8.6 Hz, 1 H, d‐Hiv‐C2*H*), 3.36 (dd, *J*=14.6, 4.9 Hz, 1 H, Phe‐C3*H*), 3.11–2.91 (m, 4 H, Phe‐C3*H* and Phe‐NC*H*_3_), 2.02 (hept., *J*=6.7 Hz, 1 H, d‐Hiv‐C3*H*), 0.80 (d, *J*=6.7 Hz, 3 H, d‐Hiv‐C4*H*_3_), 0.43 ppm (d, *J*=6.7 Hz, 3 H, d‐Hiv‐C4*H*_3_); ^13^C NMR (151 MHz, CDCl_3_): *δ*=170.0 (quat., Phe‐*C*1), 169.5 (quat., d‐Hiv‐*C*1), 136.8 (quat., Phe‐*C*4), 129.0, 128.7, 126.9 (5×CH, Phe‐*C*5–Phe‐*C*7), 75.6 (CH, d‐Hiv‐*C*2), 57.5 (CH, Phe‐*C*2), 34.9 (CH_2_, Phe‐*C*3), 32.5 (CH_3_, Phe‐N*C*), 29.8 (CH, d‐Hiv‐*C*3), 18.4, 17.6 ppm (2×CH_3_, d‐Hiv‐*C*4); IR (neat): $\tilde{\nu }$ =2964, 2933, 1743, 1659, 1456, 1411, 1370, 1263, 1178, 1106, 1015, 731, 697 cm^−1^; HRMS (ESI): found 784.4183 [*M*+H]^+^ C_45_H_58_N_3_O_9_ requires 784.4173 Δ=1.3 ppm.

**Synthesis of beauvericin (1 a) [flow]**: Dipeptidol trimer **10 a** (0.050 g, 0.05 mmol) was taken up in dichloromethane (5 mL) and this solution was hydrogenated using an H‐Cube® Pro (ThalesNano) with a 10 % Pd/C CatCart®. The pump was run at 1 mL min^−1^ using dichloromethane with the temperature set to 60 °C and the pressure to 6 bar. The solvent was then removed in vacuo and the crude acid was taken up in dioxane (1 mL). Anhydrous HCl (1 mL, 4 m in dioxane) was added and the reaction mixture was stirred at room temperature for 6 h. The solvent was then removed in vacuo and the crude oil taken up in ethanol (2×2 mL) and methanol (2 mL) successively, removing the solvent in vacuo after each addition to afford the crude cyclisation precursor.

The flow equipment was set up according to Table 4. The cyclisation precursor was taken up in dichloromethane (5 mL) and filled into loop 1. Ghosez reagent (0.013 mL, 0.1 mmol) was taken up in dichloromethane (5 mL) and filled into loop 2. Pumps 1 and 2 were run at 1 mL min^−1^ with dichloromethane and pump 3 at 1 mL min^−1^ with a solution of diisopropylethylamine (0.036 m) in dichloromethane. Injection loops 1 and 2 were injected at the appropriate time to ensure coordinated meeting of streams at the T‐pieces (determined by runs using Sudan red dye): loop 1 at *t*=0, loop 2 at *t*=23 s. From *t*=15 min to *t*=26 min the reaction outflow was collected in stirring aqueous HCl (20 mL, 1 m) at 0 °C. The reaction mixture was extracted with dichloromethane (3×20 mL) and the combined organic extracts were washed with water (10 mL), saturated NaHCO_3_ (10 mL) and saturated NaCl (10 mL) and were dried over MgSO_4_ and the solvent removed in vacuo. The crude product was purified using preparative TLC using toluene/ethyl acetate (2:1) as eluent to afford the title compound **1 a** (0.030 g, 0.038 mmol, 76 %) as a colourless solid, which was spectroscopically identical to that reported using method A.

## Supporting information

As a service to our authors and readers, this journal provides supporting information supplied by the authors. Such materials are peer reviewed and may be re‐organized for online delivery, but are not copy‐edited or typeset. Technical support issues arising from supporting information (other than missing files) should be addressed to the authors.

Supplementary
