# Supplementary material for: Synthesis of Natural and Unnatural Cyclooligomeric Depsipeptides Enabled by Flow Chemistry
Source: Chemistry. 2016 Feb 4;22(12):4206–17. doi: 10.1002/chem.201504457 (PMC4797712; doi:10.1002/chem.201504457)
Supplement: Supplementary file 1 — Supplementary [file CHEM-22-4206-s001.pdf]

CHEMISTRY
A European Journal

Supporting Information

Synthesis of Natural and Unnatural Cyclooligomeric Depsipeptides Enabled by Flow Chemistry

Daniel Lücke, Toryn Dalton, Steven V. Ley, and Zoe E. Wilson*[a]

chem_201504457_sm_miscellaneous_information.pdf

Contents

1 General Methods ... 3
2 Optimisation of flow conditions ... 4
2.1 Boc-Leu-Phe-OMe **16** ... 4
2.1.1 Method A (Optimised Batch) ... 4
2.1.2 Method B (Optimised Flow) ... 5
3 Flow general methods ... 5
3.1 Intermolecular coupling (General Method 1) ... 5
3.1.1 Acid deprotection ... 5
3.1.2 Amine deprotection ... 6
3.1.3 Coupling ... 6
3.2 Macrocyclisation (General Method 2) ... 6
3.2.1 Acid deprotection ... 6
3.2.2 Amine deprotection ... 6
3.2.3 Coupling ... 7
4 Synthesis of *N*-Me-Phe containing depsipeptides ... 7
4.1 D-Hydroxyisovaleric acid **3** ... 7
4.2 D-Hiv-OBn **4** ... 8
4.3 Boc-Me-Phe-D-Hiv-OBn **6a** ... 8
4.4 Boc-Me-Phe-D-Hiv-Me-Phe-D-Hiv-OBn **8a** ... 9
4.4.1 Method A (Batch) ... 9
4.4.2 Method B (Flow) ... 10
4.5 Boc-Me-Phe-D-Hiv-Me-Phe-D-Hiv-Me-Phe-D-Hiv-OBn **10a** ... 10
4.5.1 Method A (Batch) ... 10
4.5.2 Method B (Flow) ... 11
4.6 Boc-Me-Phe-D-Hiv-Me-Phe-D-Hiv -Me-Phe-D-Hiv-Me-Phe-D-Hiv-OBn **11a** ... 11
4.6.1 Method A (Batch) ... 12
4.6.2 Method B (Flow) ... 12
4.7 –[Me-Phe-D-Hiv-Me-Phe-D-Hiv]- **9a** ... 13
4.7.1 Method A (Batch) ... 13
4.7.2 Method B (Flow) ... 14
4.8 –[Me-Phe-D-Hiv-Me-Phe-D-Hiv-Me-Phe-D-Hiv]- **1a** (beauvericin) ... 14
4.8.1 Method A (Batch) ... 14
4.8.2 Method B (Flow) ... 15
4.9 –[Me-Phe-D-Hiv-Me-Phe-D-Hiv- Me-Phe-D-Hiv-Me-Phe-D-Hiv]- **2a** ... 15
4.9.1 Method A (Batch) ... 15
4.9.2 Method B (Flow) ... 16
5 Synthesis of *N*-Me-Leu containing depsipeptides ... 16
5.1 Boc-Me-Leu-D-Hiv-OBn **6b** ... 16
5.2 Boc-Me-Leu-D-Hiv-Me-Leu-D-Hiv-OBn **8b** ... 16
5.2.1 Method A (Batch) ... 17
5.2.2 Method B (Flow) ... 17
5.3 Boc-Me-Leu-D-Hiv-Me-Leu-D-Hiv-Me-Leu-D-Hiv-OBn **10b** ... 18
5.3.1 Method A (Batch) ... 18
5.3.2 Method B (Flow) ... 19
5.4 Boc-Me-Leu-D-Hiv-Me-Leu-D-Hiv-Me-Leu-D-Hiv-Me-Leu-D-Hiv-OBn **11b** ... 19
5.4.1 Method A (Batch) ... 19
5.4.2 Method B (Flow) ... 20
5.5 -[Me-Leu-D-Hiv-Me-Leu-D-Hiv]- **9b** ... 20
5.5.1 Method A (Batch) ... 20
5.5.2 Method B (Flow) ... 21
5.6 -[Me-Leu-D-Hiv-Me-Leu-D-Hiv-Me-Leu-D-Hiv]- **1b** (enniatin C) ... 21
5.6.1 Method A (Batch) ... 21
5.6.2 Method B (Flow) ... 22
5.7 -[Me-Leu-D-Hiv-Me-Leu-D-Hiv-Me-Leu-D-Hiv-Me-Leu- D-Hiv]- **2b** (bassianolide) ... 22
5.7.1 Method A (Batch) ... 22
5.7.2 Method B (Flow) ... 23
6 References ... 23
7 NMR Spectra ... 23

1 General Methods

All reactions were carried out under argon atmosphere using oven-dried glassware, and were monitored by TLC. Unless otherwise stated, reagents were obtained from commercial sources and used without further purification. Amino acids were all of the natural (L) enantiomeric form unless otherwise stated. Solvents were freshly distilled over calcium hydride and lithium aluminium hydride (tetrahydrofuran and diethyl ether) or calcium hydride (dichloromethane, methanol, toluene, ethyl acetate and 40-60 petroleum ether). Additional anhydrous solvents were obtained from commercial sources and used directly (*N,N*-dimethylformamide).

Flow reactions were carried out using Vapourtec R2+[1] and Syrris Asia[2] pumps and a Polar Bear Plus cooling unit[3] (Cambridge Reactor Design).

Thin layer chromatography (TLC) was carried out using 0.25 mm thick glass backed Merck TLC Silica gel 60 F_{254} plates which were visualised using ultraviolet radiation and aqueous acidic ammonium molybdate (VII) solution or potassium permanganate solution. Preparatory TLC was carried out using Analtech 20 x 20 cm UNIPLATE™ Silica gel GF (preparative layer with UV254) plates of either 500, 1000 or 2000 microns depth and were visualised using UV. Flash column chromatography was carried out using high-purity grade silica gel (Merck grade 9385) with a pore size 60 Å and 230–400 mesh particle size.

Infrared Spectroscopy was recorded using a PerkinElmer Spectrum One FT-IR spectrometer using Universal ATR sampling accessories. Absorbances were recorded in the range 4000-650 cm^{-1}.

Nuclear Magnetic Resonance Spectroscopy (NMR) spectra were recorded using either a 400 MHz DPX-400 Dual Spectrometer, a 500 MHz AV III HD Smart Probe Spectrometer or a 600 MHz Avance 600 BBI spectrometer as indicated. Unless otherwise stated, all samples were run at room temperature in deuterated solvent, with chemical shift (δ) reported to the nearest 0.01 (^{1}H)/0.1 (^{13}C) ppm , relative to the residual protic solvent; δ($CDCl_3$) = 7.26 (^{1}H)/77.16 (^{13}C) ppm, δ(toluene-d_8) = 2.09, 6.98, 7.00, 7.09 (^{1}H)/20.40, 125.49, 128.33, 129.24, 137.86 (^{13}C) or δ(d_6-DMSO) 2.50 (^{1}H)/39.52 (^{13}C) ppm. All carbon NMRs were run with broadband proton decoupling. Multiplicity of a signal in ^{1}H NMR is indicated by: s = singlet, d = doublet, t = triplet or, q = quartet, quint = quintet, m = multiplet, or a combination thereof. Multiplets are reported as the range of ppm values covered by the signals, otherwise the centre of the signal is given. Coupling constants, *J*, are quoted in Hz and recorded to the nearest 0.1 Hz. Assignments were confirmed using Distortionless Enhanced Polarisation Transfer NMR (DEPT 135) and two dimensional NMR (^{1}H–^{1}H Correlation Spectroscopy (COSY), Heteronuclear Single Quantum Coherence (HSQC) and Heteronuclear Multiple Bond Correlation (HMBC)) experiments gave information used to assign both the ^{1}H NMR and ^{13}C NMR spectra. Copies of all spectra are included at the end of this supporting information.

High Resolution Mass Spectrometry (HRMS) was performed using either a Waters Micromass LCT Premier spectrometer or by using a Bruker Bioapex 47e FTICR spectrometer, using positive Electron Spray Ionisation (ESI+). Masses are given in m/z-units, and the reported values all lie within Δ5 ppm of calculated values.

Melting points (mp) were measured using a Stanford Research Systems OptiMelt automated melting point system using a gradient of 1 °C/min, and are uncorrected.

Specific Optical Rotation was recorded on a Perkin-Elmer Model 343 digital polarimeter, using a Na/Hal lamp set at 589 nm and with a path length of 100 mm. All [α_D] values were measured using spectroscopy grade solvent at the specified concentration (in gcm^{-3}) and temperature, with units of $10^{-1}cm^2g^{-1}$.

The compound numbering system used is based on IUPAC conventions. Each amino acid is numbered individually, starting from the carboxyl group and proceeding up the side chain. Position labels thus refer first to the individual amino acid, and then to the position on it, with the atom to be defined (C or H) underlined. Non-amino acid derived functionality is numbered based on the position from which it branches. The entire branch is thus given the label of the position from which it begins, appended with a prime, and each position within the chain is further denoted by a lowercase letter.

2 Optimisation of flow conditions

2.1 Boc-Leu-Phe-OMe **16**

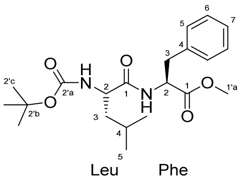

2.1.1 Method A (Optimised Batch)

A solution of Ghosez reagent (0.013 mL, 0.1 mmol) in dichloromethane (5 mL) was added to a solution of Boc-leucine **14** (0.012g, 0.05 mmol) in dichloromethane (5 mL) at 0 °C and the resulting mixture was stirred for 5 min at this temperature. A solution of phenylalanine methyl ester HCl salt **15** (0.011 g, 0.05 mmol) and *N*,*N*-diisopropylethylamine (0.032 mL, 0.18 mmol) in dichloromethane (5 mL) was added and the reaction mixture was allowed to warm to room temperature and stirred for 6 min 40 s before cooling to 0 °C and addition of aqueous HCl (10 mL, 1M). The reaction mixture was extracted with dichloromethane (3 x 10 mL) and the combined organic layers were washed with water (10 mL), saturated sodium bicarbonate ($NaHCO_3$) solution (10 mL) and saturated sodium chloride (NaCl) solution (10 mL) before drying over magnesium sulfate ($MgSO_4$) and removing the solvent *in vacuo*. The crude product was purified *via* preparative TLC using 40-60 petroleum ether:ethyl acetate (1:1) as eluent to afford the *title compound* **16** (0.009 g, 0.023 mmol, 46%) as a colourless solid.

R_f = 0.66 [40-60 petroleum ether:diethyl ether = 1:1]; m.p. = 84 – 84.5 °C (literature[4] = 83.5 – 84 °C); **$\alpha_D^{27.7}$** = +18.5 (c 1.00, $CHCl_3$); **^{1}H NMR** (600 MHz, $CDCl_3$) *δ* = 7.31 – 7.26 (m, 2H, 2 x Phe-C5H), 7.26 – 7.22 (m, 1H, Phe-C7H), 7.13 – 7.08 (m, 2H, 2 x Phe-C6H), 6.49 (d, J = 7.8 Hz, 1H, Phe-NH), 4.89 – 4.77 (m, 2H, Phe-C2H and Leu-NH), 4.14 – 4.04 (m, 1H, Leu-C2H), 3.71 (s, 3H, Phe-C1′aH_3), 3.19 – 3.06 (m, 2H, Phe-C3H_2), 1.68 – 1.57 (m, 2H, Leu-C3HH, Leu-C4H), 1.44 (br. s, 10H, Leu-C3H*H* and 3 x Leu-C2′cH_3), 0.92 (t, J = 6.6 Hz, 6H, 2 x Leu-C5H_3); **^{13}C NMR** (151 MHz, $CDCl_3$) *δ* = 172.2 (quat., Phe-C1), 171.8 (quat. Leu-C1), 155.1 (quat., Leu-C2′a), 135.9 (quat., Phe-C4), 129.4 (CH, Phe-C6), 128.7 (CH, Phe-C5), 127.3 (CH, Phe-C7), 80.2 (quat., Leu-C2′b), 53.3 (2 x CH, Phe-C2, Leu-C2), 52.4 (CH_3, Phe-C1′a), 41.4 (CH_2, Leu-C3), 38.1 (CH_2, Phe-C3), 28.4 (CH_3, 3 x Leu-C2′c), 24.8 (CH, Leu-C4), 23.0 (CH_3, Leu-C5), 22.1 (CH_3, Leu-C5); **IR** (neat/cm^{-1}): 3339, 3302, 2960, 1742, 1664, 1520, 1275, 1245, 1163, 1020, 697; **HRMS (ESI)**: found 415.2224 $(M+Na)^+$ $C_{21}H_{32}N_2O_5Na$ requires 415.2209 Δ = 3.6 ppm.

2.1.2 Method B (Optimised Flow)

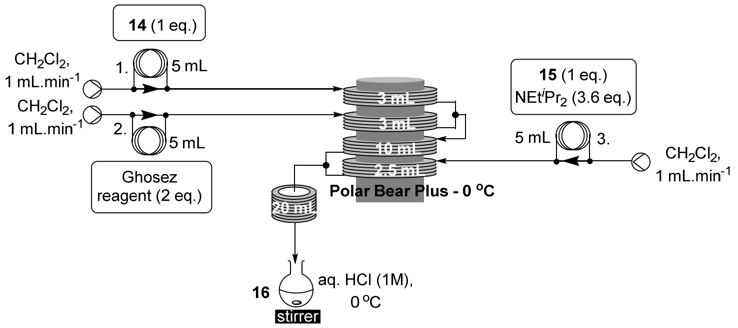

Scheme 1. Flow equipment set up for optimised flow synthesis of **16**

Plug flow: Boc-leucine **14** (0.012 g, 0.05 mmol) was taken up in dichloromethane (5 mL) and filled into loop 1. Ghosez reagent (0.013 mL, 0.1 mmol) was taken up in dichloromethane (5 mL) and filled into loop 2. Phenylalanine methyl ester HCl salt **15** (0.011 g, 0.05 mmol) and diisopropylethylamine (0.032 mL, 0.18 mmol) were taken up in dichloromethane (4.968 mL) and filled into loop 3. Pumps 1 – 3 were run a 1 mL.min^{-1} with dichloromethane and injection loops 1 – 3 injected at the appropriate time to ensure coordinated meeting of streams at the T-pieces (determined by runs using Sudan red dye) – loop 1 at t=0, loop 2 at t=23 s and loop 3 at t=4 min 53 s. From t = 15 min to 1 = 26 min the reaction outflow was collected in stirring aqueous HCl (10 mL, 1M) at 0 °C. The reaction mixture was extracted with dichloromethane (3 x 20 mL) and the combined organic extracts were washed with water (10 mL), saturated $NaHCO_3$ solution (10 mL) and saturated NaCl solution (10 mL) before drying over $MgSO_4$ and removing the solvent *in vacuo*. The resultant crude product was then purified *via* preparative TLC using 40-60 petroleum ether:ethyl acetate (1:1) as eluent to afford the *title compound* **16** (0.009 g, 0.023 mmol, 46%) as a colourless solid, which was spectroscopically identical to that reported using method A.

Continuous flow: The flow coupling was also run continuously with pump 1 pumping a solution of Boc-Leucine **14** (0.01 M) in dichloromethane, pump 2 pumping a solution of Ghosez reagent (0.02 M) in dichloromethane and pump 3 pumping a solution of phenylalanine methyl ester HCl salt **15** (0.01 M) and diisopropylethylamine (0.036 M) in dichloromethane. The reactor was allowed to reach steady state (30 min) and then the outflow was collected for 60 min into stirring aqueous HCl at 0 °C (150 mL, 1M). The reaction mixture was extracted with dichloromethane (3 x 150 mL) and the combined organic extracts washed with water (150 mL), saturated $NaHCO_3$ (150 mL) and saturated NaCl (150 mL). The combined organic extracts were dried over magnesium sulphate and the solvent removed *in vacuo*. The resultant crude product was purified using preparative TLC using 40-60 petroleum ether:ethyl acetate (1:1) as eluent to afford the *title compound* **16** (0.110 g, 0.28 mmol, 47%) as a colourless solid, which was spectroscopically identical to that reported using method A.

3 Flow general methods

3.1 Intermolecular coupling (General Method 1)

3.1.1 Acid deprotection

Coupling partner A (0.05 mmol) was taken up in ***solvent*** and this solution was hydrogenated using an H-cube® Pro (ThalesNano) with a 10% Pd/C CatCart®. The pump was run at 1 mL.min^{-1} using

solvent with the temperature set to 60 ^oC and the pressure to 6 bar. The solvent was then removed *in vacuo* and the crude ***acid*** used directly in the coupling.

3.1.2 Amine deprotection

Coupling partner B (0.05 mmol) was taken up in dioxane (1 mL) and anhydrous HCl (1 mL, 4M in dioxane) was added and the reaction mixture stirred at room temperature until the reaction was judged to be complete using TLC. The solvent was then removed *in vacuo* and the crude oil taken up in ethanol (2 x 2 mL) and methanol (2 mL) successively, removing the solvent *in vacuo* after each addition to afford the ***amine HCl*** salt which was used directly in the coupling.

3.1.3 Coupling

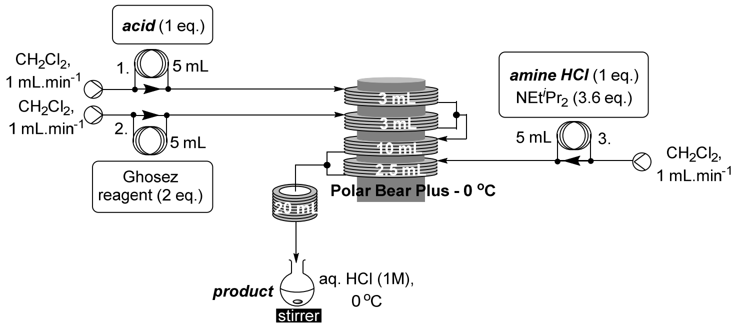

Scheme 2. Flow equipment set up for General Method 1.

Acid (0.05 mmol) was taken up in dichloromethane (5 mL) and filled into loop 1. Ghosez reagent (0.013 mL, 0.1 mmol) was taken up in dichloromethane (5 mL) and filled into loop 2. ***Amine HCl*** (0.05 mmol) and diisopropylethylamine (0.032 mL, 0.18 mmol) were taken up in dichloromethane (4.968 mL) and filled into loop 3. Pumps 1 – 3 were run a 1 mL.min^{-1} with dichloromethane and injection loops 1 – 3 injected at the appropriate time to ensure coordinated meeting of streams at the T-pieces (determined by runs using Sudan red dye) – loop 1 at t=0, loop 2 at t=23 s and loop 3 at t=4 min 53 s. From t = 15 min to 1 = 26 min the reaction outflow was collected in stirring aqueous HCl (10 mL, 1M) at 0 °C. The reaction mixture was extracted with dichloromethane (3 x 20 mL) and the combined organic extracts were washed with water (10 mL), saturated $NaHCO_3$ solution (10 mL) and saturated NaCl solution (10 mL) before drying over $MgSO_4$ and removing the solvent *in vacuo*. The resultant crude product was then purified *via* flash chromatography or preparative TLC.

3.2 Macrocyclisation (General Method 2)

3.2.1 Acid deprotection

Protected precursor (0.05 mmol) was taken up in ***solvent*** and this solution was hydrogenated using an H-cube® Pro (ThalesNano) with a 10% Pd/C CatCart®. The pump was run at 1 mL.min^{-1} using ***solvent*** with the temperature set to 60 ^oC and the pressure to 6 bar. The solvent was then removed *in vacuo* and the crude ***acid*** used directly in the amine deprotection.

3.2.2 Amine deprotection

Crude ***acid*** (0.05 mmol) was taken up in dioxane (1 mL) and anhydrous HCl (1 mL, 4M in dioxane) was added and the reaction mixture stirred at room temperature until the reaction was judged to be complete using TLC. The solvent was then removed *in vacuo* and the crude oil taken up in ethanol (2 x 2 mL) and methanol (2 mL) successively, removing the solvent *in vacuo* after each addition to afford the ***cyclisation precursor HCl*** salt which was used directly in the coupling.

3.2.3 Coupling

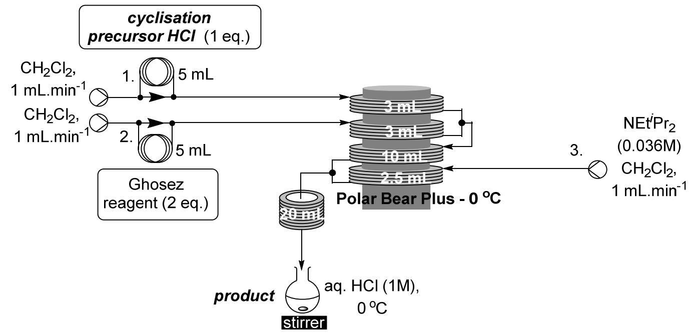

Scheme 3. Flow equipment set up for General Method 2

Cyclisation precursor HCl (0.05 mmol) was taken up in dichloromethane (5 mL) and filled into loop 1. Ghosez reagent (0.013 mL, 0.1 mmol) was taken up in dichloromethane (5 mL) and filled into loop 2. Pumps 1 and 2 were run at 1 mL.min^{-1} with dichloromethane and pump 3 at 1 mL.min^{-1} with a solution of diisopropylethylamine (0.036M) in dichloromethane. Injection loops 1 and 2 were injected at the appropriate time to ensure coordinated meeting of streams at the T-pieces (determined by runs using Sudan red dye) – loop 1 at t=0, loop 2 at t=23 s. From t = 15 min to 1 = 26 min the reaction outflow was collected in stirring aqueous HCl (20 mL, 1M) at 0 °C. The reaction mixture was extracted with dichloromethane (3 x 20 mL) and the combined organic extracts washed with water (10 mL), saturated $NaHCO_3$ solution (10 mL) and saturated NaCl solution (10 mL) and was dried over $MgSO_4$ and the solvent was removed *in vacuo* before purification *via* flash chromatography or preparative TLC.

4 Synthesis of *N*-Me-Phe containing depsipeptides

4.1 D-Hydroxyisovaleric acid **3**

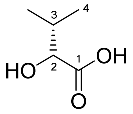

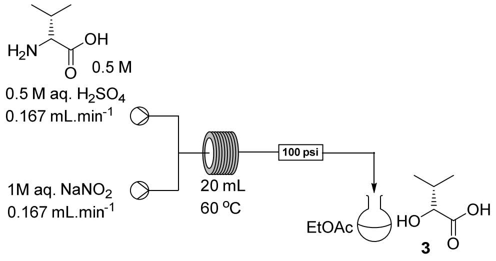

Scheme 4. Flow equipment set up for synthesis of **3**

D-Hydroxyisovaleric acid **3** was synthesised using a modification of the procedure previously reported by the Ley group.[5] A solution of D-valine (3.41 g, 29.1 mmol) in sulfuric acid (58.2 mL, 0.5 M in water) at a flow rate of 0.167 mL.min^{-1} was mixed with a solution of sodium nitrite (4.02 g, 58.2 mmol) in water (58.2 mL) at a flowrate of 0.167 mL.min^{-1} at a T-piece (0.333 mL.min^{-1} total). The reaction mixture was passed through a 20 mL reactor coil at 60 °C and a 100 psi back pressure regulator before the output was collected in stirring ethyl acetate (150 mL). The aqueous layer was extracted with ethyl acetate (4 x 100 mL) and the combined organic layers were washed with

saturated NaCl solution (250 mL) before drying over $MgSO_4$ and removing the solvent *in vacuo.* The crude *title compound* **3** (2.74 g, 23.2 mmol, 80%) was obtained as a yellow solid, which was used in the next step without further purification. The ^{1}H and ^{13}C NMR data was in agreement with that reported in the literature.[6]

^{1}H NMR (400 MHz, d_6-DMSO) δ = 3.77 (d, J = 4.8 Hz, 1H, C2H), 1.95 (hept.d, J = 6.9, 4.8 Hz, 1H, C3H), 0.93 (d, J = 6.9 Hz, 3H, C4H_3), 0.87 (d, J = 6.9 Hz, 3H, C4H_3); **^{13}C NMR** (101 MHz, d_6-DMSO) δ = 175.3 (quat., C1), 74.5 (CH, C2), 31.4 (CH, C3), 18.9 (CH_3, C4), 16.8 (CH_3, C4).

4.2 D-Hiv-OBn 4

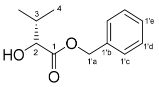

Caesium carbonate (4.45 g, 13.7 mmol) was added to a solution of D-hydroxyisovaleric acid **3** (3.22 g, 27.3 mmol) in *N*,*N*-dimethylformamide (20 mL) at 0 °C and the resulting mixture was stirred for 40 min. Benzyl bromide (3.6 mL, 30.0 mmol) was added and the reaction mixture was stirred at room temperature for 15 h. The reaction mixture was filtered and the filtrate diluted with 40-60 petroleum ether:ethyl acetate (4:1, 120 mL). The organic layer was washed with saturated ammonium chloride solution (100 mL), saturated $NaHCO_3$ solution (100 mL) and saturated NaCl solution (100 mL) before drying over $MgSO_4$ and removing the solvent *in vacuo*. The crude product was purified *via* flash column chromatography using 40-60 petroleum ether:ethyl acetate (10:1) as eluent to afford the *title compound* **4** (4.56 g, 21.9 mmol, 80%) as a colourless oil. The ^{1}H and ^{13}C data was in agreement with that reported in the literature[7].

R_f = 0.29 [40-60 petroleum ether:diethyl ether = 10:1]; **$\alpha_D^{25.8}$** = +17.8 (c 3.35 EtOH) literature[8] $\alpha_D^{20.0}$ = +15.4 (c 2.1, EtOH); **^{1}H NMR** (400 MHz, d_6-DMSO) δ = 7.49 – 7.26 (m, 5H, C1'cH – C1'eH), 5.32 (br s., 1H, C2OH), 5.25 – 5.02 (m, 2H, C1'aH_2), 3.86 (d, J = 5.0 Hz, 1H, C2H), 2.04 – 1.81 (m, 1H, C3H), 0.87 (d, J = 6.9 Hz, 3H, C4H_3), 0.81 (d, J = 6.9 Hz, 3H, C4H_3); **^{13}C NMR** (151 MHz, d_6-DMSO) δ = 172.5 (quat., C1), 135.6 (quat., C1'b), 127.6, 127.5, 127.2 (5 x CH, C1'c – C1'e), 74.7 (CH_2, C1'a), 64.8 (CH, C2), 31.1 (CH, C3), 17.7 (CH_3, C4), 16.3 (CH_3, C4).

4.3 Boc-Me-Phe-D-Hiv-OBn 6a

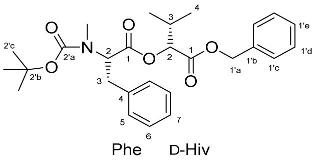

1-Ethyl-3-(3-dimethylaminopropyl)carbodiimide hydrochloride (EDCI) (1.72 g, 9.0 mmol) was added to a solution of D-Hiv-OBn **4** (1.30 g, 6.3 mmol), Boc-*N*-methyl-L-phenylalanine **5a** (2.50 g, 8.95 mmol) and 4-dimethylaminopyridine (4-DMAP) (0.99 g, 8.1 mmol) in dichloromethane (25 mL) at 0 °C and the resulting mixture was stirred at room temperature for 14.5 h. The reaction was ended by the addition of aqueous HCl (25 mL, 1M) and the phases were separated. The aqueous layer was extracted with dichloromethane (3 x 30 mL) and the combined organic layers were washed with water (100 mL), saturated $NaHCO_3$ solution (100 mL) and saturated NaCl solution (100 mL) before drying over $MgSO_4$ and removing the solvent *in vacuo*. The crude product was purified *via* flash column chromatography using 40-60 petroleum ether:ethyl acetate (10:1) as eluent to afford the *title compound* **6a** (2.69 g, 5.7 mmol, 92%) as a colourless oil.

R$_f$ = 0.34 [40-60 petroleum ether:diethyl ether = 10:1]; **$\alpha_D^{23.7}$** = -30.9 (c 1.00, $CHCl_3$); **^{1}H NMR** (400 MHz, d$_6$-DMSO, T = 403 °K) *δ* = 7.47 – 7.13 (m, 10H, D-Hiv-C1′cH – D-Hiv-C1′eH and Phe-C5H – Phe-C7H), 5.20 (s, 2H, D-Hiv-C1′aH_2), 4.99 (dd, *J* = 10.4, 5.4 Hz, 1H, Phe-C2H), 4.92 (d, *J* = 4.6 Hz, 1H, D-Hiv-C2H), 3.23 (dd, *J* = 14.6, 5.4 Hz, 1H, Phe-C3H), 3.01 (dd, *J* = 14.6, 10.4 Hz, 1H, Phe-C3H), 2.70 (s, 3H, Phe-NCH_3), 2.28 – 2.13 (m, 1H, D-Hiv-C3H), 1.33 (s, 9H, Phe-C2′cH_3), 0.96 (d, *J* = 6.9 Hz, 3H, D-Hiv-C4H_3), 0.93 (d, *J* = 6.9 Hz, 3H, D-Hiv-C4H_3); **^{13}C NMR** (101 MHz, d$_6$-DMSO, T = 403 °K) δ 169.6 (quat., Phe-C1), 167.8 (quat., D-Hiv-C1), 155.5 (quat., Phe-C2′a), 136.8 (quat., Phe-C4), 135.0 (quat., D-Hiv-C1′b), 128.0, 127.7, 127.4, 127.3, 125.6 (10 x CH, D-Hiv-C1′c – D-Hiv-C1′e, Phe-C5 – Phe-C7), 78.7 (quat., Phe-C2′b), 76.4 (CH, D-Hiv-C2), 65.7 (CH_2, D-Hiv-C1′a), 58.8 (CH, Phe-C2), 33.8 (CH_2, Phe-C3), 30.2 (CH_3, Phe-NC), 29.1 (CH, D-Hiv-C3), 27.3 (3 x CH_3, Phe-C2′c), 17.5 (CH_3, D-Hiv-C4), 16.2 (CH_3, D-Hiv-C4); **IR** (neat/cm^{-1}): 2972, 2933, 2880, 1741, 1694 , 1455, 1390, 1366, 1167, 1127, 1029, 750, 697; **HRMS (ESI)**: found 470.2531 $(M+H)^+$ $C_{27}H_{36}NO_6$ requires 470.2543 Δ = 2.4 ppm.

4.4 Boc-Me-Phe-D-Hiv-Me-Phe-D-Hiv-OBn **8a**

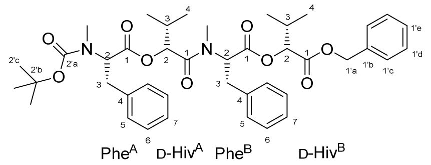

4.4.1 Method A (Batch)

Anhydrous HCl (2.5 mL, 4M in dioxane) was added to a solution of Boc-Me-Phe-D-Hiv-OBn **6a** (0.31 g, 0.66 mmol) in dioxane (2.5 mL) at room temperature and the resulting mixture was stirred for 3 h. The solvent was removed *in vacuo* and the residue was taken up sequentially in ethanol (2 x 5 mL) and methanol (5 mL) with the solvent removed *in vacuo* after each addition to afford the amine HCl salt which was used in the next step without further purification.

Hydrogen gas was bubbled through a solution of Boc-Me-Phe-D-Hiv-OBn **6a** (0.31 g, 0.66 mmol) and palladium (70 mg, 10% on charcoal, 0.07 mmol) in tetrahydrofuran (5 mL) for 3 min at room temperature before the resulting mixture was stirred under a hydrogen atmosphere for 2.5 h. The reaction mixture was filtered through celite, eluting with ethyl acetate (20 mL). The solvent was removed *in vacuo* from the filtrate to afford the acid which was used in the next step without further purification.

Ghosez reagent (0.096 mL, 0.72 mmol) was added to a solution of the crude acid in dichloromethane (2.5 mL) at 0 °C and the resulting mixture was stirred for 20 min at this temperature. A solution of the crude amine HCl salt and *N*,*N*-diisopropylethylamine (0.40 mL, 2.36 mmol) in dichloromethane (2.5 mL) was added and the reaction mixture was allowed to warm to room temperature and stirred for 17 h. Aqueous HCl (20 mL, 1M) was added and the phases were separated. The aqueous layer was extracted with dichloromethane (3 x 20 mL) and the combined organic layers were washed with water (40 mL), saturated $NaHCO_3$ solution (40 mL) and saturated NaCl solution (40 mL) before drying over $MgSO_4$ and removing the solvent *in vacuo*. The crude product was purified *via* flash column chromatography using 40-60 petroleum ether:ethyl acetate (10:1) as eluent to afford the *title compound* **8a** (0.33 g, 0.45 mmol, 68%) as a colourless solid.

R$_f$ = 0.06 [40-60 Petroleum ether:diethyl ether = 10:1]; **mp** = 120-122 °C; **$\alpha_D^{23.7}$** = -47.4 (c 1.00, $CHCl_3$); **^{1}H NMR** (400 MHz, d$_6$-DMSO, T = 403 °K) *δ* = 7.51 – 7.31 (m, 5H, D-Hiv^B-C1′cH – D-Hiv^B-C1′eH), 7.31 –

7.08 (m, 10H, Phe^{A+B}-C5H – Phe^{A+B}-C7H), 5.39 (br. s, 1H, Phe^{B}-C2H), 5.21 (d, *J* = 1.4 Hz, 2H, D-Hiv^{B}-C1'aH_2), 5.11 (d, *J* = 4.8 Hz, 1H, D-Hiv^{A}-C2H), 4.99 – 4.90 (m, 2H, Phe^{A}-C2H and D-Hiv^{B}-C2H), 3.32 (dd, *J* = 14.6, 5.4 Hz, 1H, Phe^{B}-C3H*H*), 3.21 (dd, *J* = 14.6, 5.2 Hz, 1H, Phe^{A}-C3H*H*), 3.07 – 2.96 (m, 2H Phe^{A}-C3H*H* and Phe^{B}-C3H*H*), 2.94 (s, 3H, Phe^{B}-NCH_3), 2.72 (s, 3H, Phe^{A}-NCH_3), 2.22 (pd, *J* = 6.9, 4.8 Hz, 1H, D-Hiv^{B}-C3H), 1.81 (br. s, 1H, D-Hiv^{A}-C3H), 1.31 (s, 9H, 3 x Phe^{A}-C2'cH_3), 0.95 (dd, *J* = 6.9, 4.3 Hz, 6H, 2 x D-Hiv^{B}-C4H_3), 0.78 (d, *J* = 6.8 Hz, 3H, D-Hiv^{A}-C4H_3), 0.71 – 0.55 (m, 3H, D-Hiv^{A}-C4H_3); **^{13}C NMR** (101 MHz, d_6-DMSO, T = 403 °K) δ = 169.3 (quat., Phe^{A}-C1), 169.1 (quat., Phe^{B}-C1), 168.3 (quat., D-Hiv^{A}-C1), 167.8 (quat., D-Hiv^{B}-C1), 154.1 (quat., Phe^{A}-C2'a), 136.9 (quat., Phe^{A}-C4), 136.4 (quat., Phe^{B}-C4), 135.0 (quat., D-Hiv^{B}-C1'b), 128.1, 128.0, 127.7, 127.5, 127.4, 127.4, 125.7, 125.6 (15 x CH, Phe^{A+B}-C5 – Phe^{A+B}-C7 and D-Hiv^{B}-C1'c – D-Hiv^{B}-C1'e), 78.5 (quat., Phe^{A}-C2'b), 76.7 (CH, D-Hiv^{B}-C2), 74.4 (CH, D-Hiv^{A}-C2), 65.7 (CH_2, D-Hiv^{B}-C1'a), 58.7 (CH, Phe^{A}-C2), 57.5 (CH, Phe^{B}-C2), 33.9 (CH_2, Phe^{B}-C3), 33.6 (CH_2, Phe^{A}-C3), 31.0 (CH_3, Phe^{B}-NC), 30.0 (CH_3, Phe ^{A}-NC), 29.0 (CH, D-Hiv^{B}-C3), 28.6 (CH, D-Hiv^{A}-C3), 27.3 (3 x CH_3, Phe^{A}-C2'c), 17.8 (CH_3, D-Hiv^{A}-C4), 17.4 (CH_3, D-Hiv^{B}-C4), 16.3 (CH_3, D-Hiv^{B}-C4), 15.7 (CH_3, D-Hiv^{A}-C4); **IR** (neat/cm^{-1}): 2962, 2937, 1761, 1737, 1683, 1662, 1454, 1394, 1290, 1167, 1128, 1029, 745, 696; **HRMS (ESI)**: found 731.3878 $(M+H)^+$ $C_{42}H_{55}N_2O_9$ requires 731.3907 Δ = 3.9 ppm.

4.4.2 Method B (Flow)

According to General Method 1, a solution of ***coupling partner A*** **6a** (0.024 g, 0.05 mmol) in dichloromethane (5 mL) was hydrogenated to give the acid and the nitrogen of ***coupling partner B*** **6a** (0.024 g, 0.05 mmol) was deprotected using acid (reaction time = 3 h) and the resultant crude products were coupled in flow. The crude product was purified using preparative TLC using 40-60 petroleum ether:ethyl acetate (2:1) as eluent to afford the *title compound* **8a** (0.031 g, 0.042 mmol, 82%) as a colourless solid, which was spectroscopically identical to that reported using method A.

4.5 Boc-Me-Phe-D-Hiv-Me-Phe-D-Hiv-Me-Phe-D-Hiv-OBn **10a**

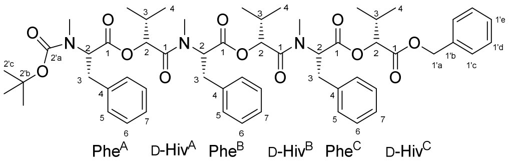

4.5.1 Method A (Batch)

Anhydrous HCl (2 mL, 4M in dioxane) was added to a solution of Boc-Me-Phe-D-Hiv-Me-Phe-D-Hiv-OBn **8a** (0.17 g, 0.23 mmol) in dioxane (2 mL) at room temperature and the resulting mixture was stirred for 6 h. The solvent was removed *in vacuo* and the residue was taken up sequentially in ethanol (2 x 5 mL) and methanol (5 mL) with the solvent removed *in vacuo* after each addition to afford the crude amine HCl salt which was used in the next step without further purification.

Hydrogen gas was bubbled through a solution of Boc-Me-Phe-D-Hiv-OBn **6a** (0.11 g, 0.23 mmol) and palladium (24 mg, 10% on charcoal, 0.02 mmol,) in tetrahydrofuran (5 mL) for 3 min at room temperature before the resulting mixture was stirred under hydrogen for 2.5 h. The reaction mixture was filtered through celite, which was washed with ethyl acetate (20 mL). The solvent was removed *in vacuo* from the filtrate to afford the crude acid which was used in the next step without further purification.

Ghosez reagent (31 µL, 0.23 mmol) was added to a solution of the crude acid in dichloromethane (3 mL) at 0 °C and the resulting mixture was stirred for 20 min. A solution of the crude amine and *N*,*N*-diisopropylethylamine (0.14 mL, 0.84 mmol) in dichloromethane (3 mL) was added and the reaction mixture was stirred at room temperature for 18 h. Aqueous HCl (5 mL, 1M) was added, the reaction mixture was diluted with diethyl ether (10 mL) and the phases were separated. The aqueous layer was extracted with diethyl ether (3 x 10 mL) and the combined organic layers were washed with water (30 mL), saturated $NaHCO_3$ solution (30 mL) and saturated NaCl solution (30 mL) before drying over $MgSO_4$ and removing the solvent *in vacuo*. The crude product was purified *via* flash column chromatography using 40-60 petroleum ether:ethyl acetate (10:1 → 5:1) as eluent to afford the *title compound* **10a** (0.18 g, 0.18 mmol, 75%) as a colourless solid.

R_f = 0.16 [40-60 petroleum ether:diethyl ether = 5:1]; **mp** = 142-143; **$\alpha_D^{23.7}$** = -60.8 (c 1.00 $CHCl_3$); **^{1}H NMR** (500 MHz, d_6-DMSO, T = 393 °K) δ = 7.45 – 7.33 (m, 5H, D-Hiv^C-C1′cH – D-Hiv^C-C1′eH), 7.33 – 7.12 (m, 15H, Phe^{A-C}-C5H – Phe^{A-C}C7H), 5.37, 5.33 (2 overlaid br. s, 2H, Phe^{B+C}-C2H), 5.21 (d, *J* = 2.9 Hz, 2H, D-Hiv^C-C1′aH_2), 5.12 (d, *J* = 4.8 Hz, 2H, D-Hiv^{A+B}-C2H), 4.96-4.92 (m, 2H, D-Hiv^C-C2H and Phe^A-C2H), 3.38 – 3.24 (m, 2H, Phe^{B+C}-C3H*H*), 3.20 (dd, *J* = 14.6, 5.1 Hz, 1H, Phe^A-C3H*H*), 3.12 – 2.90 (m, 9H, Phe^{A-C}-C3H*H*, Phe^{B+C}-NCH_3), 2.70 (s, 3H, Phe^A-NCH_3), 2.31 – 2.16 (1H, m, D-Hiv^C-C3H), 1.81 (br. s, 2H, D-Hiv^{A+B}-C3H), 1.30 (s, 9H, Phe^A-C2′cH_3), 0.95 (dd, *J* = 6.9, 5.2 Hz, 6H, D-Hiv^C-C4H_3), 0.78 (d, *J* = 6.8 Hz, 6H, D-Hiv$^{A\text{ or }B}$-C4H_3), 0.66 (s, 6H, D-Hiv$^{A\text{ or }B}$-C4H_3); **^{13}C NMR** (126 MHz, d_6-DMSO, T = 393 °K) δ = 170.4, 170.3, 170.0 (3 x quat., Phe^{A+B+C}-C1), 169.5, 169.3 (2 x quat., D-Hiv^{A+B}-C1), 168.9 (quat., D-Hiv^c-C1), 155.3 (quat., Phe^A-C2′a), 138.1 (quat., Phe^A-C4), 137.6, 137.5 (2 x quat., Phe^{B+C}-C4), 136.1 (quat., D-Hiv^B-C1′b), 129.3, 129.1, 128.8, 128.7, 128.6, 128.6, 128.6, 126.9, 126.9, 126.7 (20 x CH, Phe^{A+B+C}-C5 – Phe^{A+B+C}C7 and D-Hiv^C-C1′c – D-Hiv^C-C1′e), 79.6 (quat., Phe^A-C2′b), 77.9 (CH, D-Hiv^C-C2), 76.0, 75.6 (2 x CH, D-Hiv^{A+B}-C2), 66.9 (CH_2, D-Hiv^C-C1′a), 59.9 (CH, Phe^A-C2), 58.6, 58.5 (2 x CH, Phe^{B+C}-C2), 35.0 (CH_2, Phe^A-C3), 34.8, 34.7 (2 x CH_2, Phe^{B+C}-C3), 32.4, 32.2 (2 x CH_3, Phe^{B+C}-NC), 31.2 (CH_3, Phe^A-NC), 30.2 (CH, D-Hiv^C-C3), 29.7, 29.6 (2 x CH, D-Hiv^{A+B}-C3), 28.4 (3 x CH_3, Phe^A-C2′c), 19.0, 18.9 (2 x CH_3, D-Hiv$^{A\text{ or }B}$-C4), 18.6, 17.5 (2 x CH_3, D-Hiv^C-C4), 16.9, 16.8 (2 x CH_3, D-Hiv$^{A\text{ or }B}$-C4); **IR** (neat/cm^{-1}): 2968, 2936, 1733, 1694, 1653, 1455, 1391, 1367, 1264, 1169, 1130, 1025, 741, 696; **HRMS (ESI)**: found 992.5297 $(M+H)^+$ $C_{57}H_{74}N_3O_{12}$ requires 992.5273 Δ = 2.4 ppm.

4.5.2 Method B (Flow)

According to General Method 1, a solution of ***coupling partner A*** **6a** (0.024 g, 0.05 mmol) in dichloromethane (5 mL) was hydrogenated to give the acid and the nitrogen of ***coupling partner B*** **8a** (0.037 g, 0.05 mmol) was deprotected using HCl (reaction time = 6 h) and the resultant crude products were coupled in flow. The crude product was purified using preparative TLC using 40-60 petroleum ether:ethyl acetate (2:1) as eluent to afford the *title compound* **10a** (0.039 g, 0.039 mmol, 78%) as a colourless solid, which was spectroscopically identical to that reported using method A.

4.6 Boc-Me-Phe-D-Hiv-Me-Phe-D-Hiv -Me-Phe-D-Hiv-Me-Phe-D-Hiv-OBn **11a**

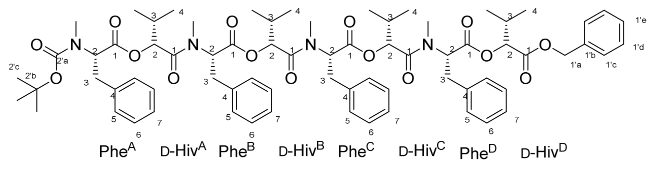

4.6.1 Method A (Batch)

Anhydrous HCl (3 mL, 4M in dioxane) was added to a solution of **8a** (0.10 g, 0.14 mmol) in dioxane (3 mL) and the reaction mixture was stirred at room temperature for 6 h before the solvent was removed *in vacuo*. Ethanol (2 x 3 mL) and methanol (3 mL) were added successively, removing the solvent *in vacuo* after each addition to afford the crude amine HCl salt which was used in the next step without further purification.

Hydrogen gas was bubbled through a solution of **8a** (0.10 g, 0.14 mmol) and palladium (0.015 g, 10% on charcoal, 0.014 mmol) in tetrahydrofuran (4 mL) for 5 min before stirring under a hydrogen atmosphere for 6 h. The reaction mixture was filtered through cotton wool and a syringe filter and the solvent was removed *in vacuo* to afford crude acid which was used in the next step without further purification.

Ghosez reagent (0.02 mL, 0.15 mmol) was added to a solution of the crude acid in dichloromethane (5 mL) at 0 °C and the reaction mixture was stirred at this temperature for 20 min before a solution of the crude amine HCl salt and diisopropylethylamine (0.088 mL, 0.5 mmol) in dichloromethane (5 mL) was added and the reaction mixture was warmed to room temperature and stirred for 20.5 h. The solvent was removed *in vacuo* and the crude taken up in diethyl ether (5 mL) and aqueous HCl (5 mL, 1M). The reaction mixture was extracted with diethyl ether (3 x 5 mL) and the combined organic extracts were washed with water (5 mL), saturated $NaHCO_3$ (5 mL) and saturated NaCl (5 mL) and were dried over $MgSO_4$ before the solvent was removed *in vacuo* to afford a crude oil. This was purified using preparative TLC using 40-60 petroleum ether:ethyl acetate (2:1) as eluent to afford the *title compound* **11a** (0.10 g, 0.08 mmol, 57%) as a colourless solid.

R_f = 0.36 [40-60 petroleum ether:diethyl ether = 2:1]; **mp** = 168 – 169 °C; **$\alpha_D^{27.8}$** = -48.8 (c 1.00 $CHCl_3$); **^{1}H NMR** (500 MHz, d_6-DMSO, T = 393 °K) δ = 7.47 – 7.31 (m, 5H, D-Hiv^D-C1′cH – D -Hiv^C-C1′eH), 7.30 – 7.10 (m, 20H, Phe^{A-D}-C5H – Phe^{A-D}-C7H), 5.58 – 5.25 (m, 3H, Phe^{B-D}-C2H), 5.25 – 5.15 (m, 2H, D -Hiv^D-C1′aH), 5.12 (d, *J* = 6.1 Hz, 3H, D-Hiv^{A-C}-C2H), 5.02 – 4.78 (m, 2H, Phe^A-C2H and D-Hiv^D-C2H), 3.39 – 3.22 (m, 3H, Phe^{B-D}-C3H*H*), 3.19 (dd, *J* = 14.6, 5.1 Hz, 1H, Phe^A-C3H*H*), 3.11 – 2.81 (m, 13H, Phe^{A-D}-C3H*H*, Phe^{B-D}-NCH_3), 2.69 (s, 3H, Phe^A-NCH_3), 2.32 – 2.06 (m, 1H, D-Hiv^D-C3H), 1.77 (br. s, 3H, D-Hiv^{A-C}-C3H), 1.29 (s, 9H, Phe^A-C2′cH_3), 0.94 (t, *J* = 6.2 Hz, 6H, D-Hiv^D-C4H_3), 0.76 (s, 9H, D-Hiv^{A-C}-C4H_3), 0.63 (s, 9H, D-Hiv^{A-C}-C4H_3); **^{13}C NMR** (126 MHz, d_6-DMSO, T = 393 °K) δ = 170.6 (quat., Phe^A-C1), 170.2, 169.93, 169.86 (3 x quat., Phe^{B-D}-C1), 169.4, 169.3 (quat., Hiv^{A-C}-C1), 168.9 (quat., Hiv^D-C1), 155.2 (quat., Phe^A-C2′a), 138.0 (quat., Phe^A-C4), 137.6, 137.4, 136.0 (3 x quat., Phe^{B-D}-C4), 129.2 (quat., D-Hiv^B-C1′b), 129.1, 128.8, 128.6, 128.5, 128.5, 126.8, 126.8, 126.6 (25 x CH, Phe^{A-D}-C5 – Phe^{A-D}-C7 and D-Hiv^D-C1′c – D-Hiv^D-C1′e), 79.5 (quat., Phe^A-C2′b), 77.8 (CH, D-Hiv^D-C2), 75.9, 75.5 (3 x CH, D-Hiv^{A-C}-C2), 66.8 (CH_2, D-Hiv^D-C1′a), 59.8 (CH, Phe^A-C2), 58.5(3 x CH, Phe^{B-D}-C2), 34.9 (4 x CH_2, Phe^{A-D}-C3), 32.3 (3 x CH_3, Phe^{B-D}-NC), 31.1 (CH_3, Phe^A-NC), 30.1 (CH, D-Hiv^D-C3), 29.6 (3 x CH, D-Hiv^{A-C}-C3), 28.4 (3 x CH_3, Phe^A-C2′c), 18.94, 18.89, 18.85 (4 x CH_3, from D-Hiv^{A-C}-C4), 18.5, 17.4 (2 x CH_3, D-Hiv^D-C4), 16.8, 16.8 (2 x CH_3, from D-Hiv^{A-C}-C4); **IR** (neat/cm^{-1}): 2965, 2929, 1733, 1685, 1657, 1457, 1270, 1225, 1170, 1027, 739, 698; **HRMS (ESI)**: found 1275.6443 $(M+Na)^+$ $C_{72}H_{92}N_4O_{15}Na$ requires 1275.6451, Δ = 0.6 ppm.

4.6.2 Method B (Flow)

According to General Method 1, a solution of ***coupling partner A*** **8a** (0.037 g, 0.05 mmol) in dichloromethane (5 mL) was hydrogenated to give the acid and ***coupling partner B*** **8a** (0.037 g,

0.05 mmol) was acid deprotected (reaction time = 6 h) and the resultant crude products were coupled in flow. The crude product was purified using preparative TLC using 40-60 petroleum ether:ethyl acetate (2:1) as eluent to afford the *title compound* **11a** (0.042 g, 0.034 mmol, 67%) as a colourless solid, which was spectroscopically identical to that reported using method A.

4.7 –[Me-Phe-D-Hiv-Me-Phe-D-Hiv]- **9a**

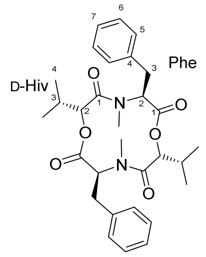

4.7.1 Method A (Batch)

Palladium (0.02 g, 10% on charcoal, 0.01 mmol) was added to a solution of Boc-Me-Phe-D-Hiv-Me-Phe-D-Hiv-OBn **6a** (0.10 g, 0.14 mmol) and hydrogen was bubbled through the reaction mixture for 5 min before stirring under a hydrogen atmosphere for 6 h. The reaction mixture was filtered through a plug of cotton wool and a syringe filter and the solvent was removed *in vacuo* from the filtrate to afford the crude acid. This was taken up in dioxane (3 mL) and anhydrous HCl (3 mL, 4M in dioxane) added. The reaction mixture was stirred at room temperature for 6 h before the solvent was removed *in vacuo*. Ethanol (2 x 3 mL) and methanol (3 mL) were added successively and the solvent removed *in vacuo* after each addition to afford the doubly deprotected depsipeptide HCl salt. This was taken up in dichloromethane (14 mL) and was cooled to 0 °C before Ghosez reagent (0.02 mL, 0.15 mmol) was added. The reaction mixture was stirred at 0 °C for 20 min before diisopropylethylamine (0.09 mL, 0.50 mmol) was added and the reaction mixture warmed to room temperature and stirred for 21 h. The solvent was removed *in vacuo* and the crude taken up in diethyl ether (5 mL) and aqueous HCl (5 mL, 1M). The reaction mixture was extracted with diethyl ether (3 x 5 mL) and the combined organic extracts were washed with water (5 mL), saturated $NaHCO_3$ (5 mL) and saturated NaCl (5 mL). The combined aqueous extracts were further extracted with ethyl acetate (3 x 5 mL) and the combined organics were dried over $MgSO_4$ before the solvent was removed *in vacuo*. The resulting crude oil was purified using preparative TLC using 40-60 petroleum ether:ethyl acetate (1:1) as eluent to afford the *title compound* **9a** (0.012 g, 0.024 mmol, 17%) as a colourless solid and *cyclic tetramer* **2a** (0.030 g, 0.029 mmol, 41%) as a colourless solid which was spectroscopically identical to that reported for this compound below.

Characterisation of 9a: R*f* = 0.30 [toluene:ethyl acetate = 2:1]; **mp** = 54.5 – 55 °C, **$\alpha_D^{27.8}$** = +16.7 (c 1.00, $CHCl_3$); **^{1}H NMR** (600 MHz, $CDCl_3$) δ = 7.31 (dd, J = 8.1, 6.6 Hz, 2H, 2 x Phe-C5H), 7.28 – 7.23 (m, 1H, Phe-C7H), 7.18 (dd, J = 6.9, 1.7 Hz, 2H, 2 x Phe-C6H), 4.88 (t, J = 7.8 Hz, 1H, Phe-C2H), 4.76 (d, J = 10.4 Hz, 1H, D-Hiv-C2H), 3.14 (s, 4H, Phe-C3H*H* and Phe-NCH$_3$), 3.03 (dd, J = 13.8, 8.2 Hz, 1H, Phe-C3H*H*), 1.69 (ddt, J = 16.3, 12.5, 6.3 Hz, 1H, D-Hiv-C3H), 0.67 (dd, J = 13.3, 6.6 Hz, 6H, 2 x D-Hiv-C4H$_3$); **^{13}C NMR** (151 MHz, $CDCl_3$) δ = 169.4 (quat., Phe-C1), 168.5 (quat., D-Hiv-C1), 135.4 (quat., Phe-C4), 129.1 (2 x CH, Phe-C5), 129.0 (2 x CH, Phe-C6), 127.5 (CH, Phe-C7), 83.3 (CH, D-Hiv-C2), 59.9 (CH, Phe-C2), 36.8 (CH_2, Phe-C3), 30.6 (CH, D-Hiv-C3), 30.3 (CH_3, Phe-NC), 19.7 (CH_3, D-Hiv-C4), 18.1 (CH_3, D-Hiv-C4); **IR** (neat/cm^{-1}): 2966, 2929, 1749, 1652, 1435, 1404, 1366,. 1285, 1007, 700, 668; **HRMS (ESI)**: found 523.2800 $(M+H)^+$ $C_{30}H_{39}N_2O_6$ requires 523.2803, Δ = 0.5 ppm.

4.7.2 Method B (Flow)

According to General Method 2, a solution of ***protected precursor*** **8a** (0.037 g, 0.05 mmol) in dichloromethane (5 mL) was hydrogenated to give the acid and then the nitrogen acid deprotected (reaction time = 6 h) and the resultant crude product macrocyclised in flow. The crude product was purified using preparative TLC using toluene:ethyl acetate (2:1) as eluent to afford the *title compound* **9a** (0.018 g, 0.034 mmol, 68%) as a colourless solid, which was spectroscopically identical to that reported using method A.

4.8 –[Me-Phe-D-Hiv-Me-Phe-D-Hiv-Me-Phe-D-Hiv]- **1a** (beauvericin)

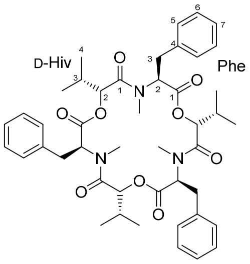

4.8.1 Method A (Batch)

Hydrogen gas was bubbled through a solution of Boc-Me-Phe-D-Hiv-Me-Phe-D-Hiv-Me-Phe-D-Hiv-OBn **10a** (83 mg, 84 µmol) and palladium (9 mg, 10% on charcoal, 8.4 µmol) in tetrahydrofuran (2 mL) for 3 min at room temperature before the mixture was stirred under hydrogen for 6 h. The reaction mixture was filtered through celite, washing with ethyl acetate (30 mL). The solvent was removed *in vacuo* from the filtrate to afford the crude acid, which was then taken up in dioxane (2 mL). Anhydrous HCl (2 mL, 4M in dioxane) was added and the resulting mixture was stirred for 6 h. The solvent was removed *in vacuo* and the residue was taken up sequentially in ethanol (2 x 5 mL) and methanol (5 mL) removing the solvent *in vacuo* after each addition to afford the deprotected linear precursor. This was taken up in dichloromethane (20 mL) and cooled to 0 °C before Ghosez reagent (12 µL, 92 µmol) was added at and the resulting mixture was stirred for 30 min at this temperature. *N*,*N*-diisopropylethylamine (51 µL, 301 µmol) was added and the reaction mixture was warmed to room temperature and stirred for 18.5 h. The solvent was removed *in vacuo* and the residue was taken up in diethyl ether (60 mL) and aqueous HCl (60 mL, 1M). The aqueous layer was extracted with diethyl ether (3 x 30 mL) and the combined organic layers were washed with water (100 mL), saturated $NaHCO_3$ solution (100 mL) and saturated NaCl solution (100 mL) before drying over $MgSO_4$ and removing the solvent *in vacuo*. The crude product was purified *via* flash column chromatography using 40-60 petroleum ether:ethyl acetate (2:1 → 1:1) as eluent to afford the *title compound* **1a** (46 mg, 59 µmol, 70%) as a colourless solid. The ^{1}H and ^{13}C NMR spectrum were in agreement with that published in the literature.[9]

R_f = 0.15 [40-60 petroleum ether:diethyl ether = 1:1]; **mp** = 92 – 93 °C (literature = 92 – 93 °C[9]); **$\alpha_D^{24.7}$** = +75 (c 0.1 MeOH) literature[10] $\alpha_D^{20.0}$ = +69 (c 0.1, MeOH); **^{1}H NMR** (600 MHz, $CDCl_3$) δ = 7.41 – 7.07 (m, 5H, Phe-C5H – Phe-C7H), 5.46 (dd, *J* = 11.5, 4.9 Hz, 1H, Phe-C2H), 4.92 (d, *J* = 8.6 Hz, 1H, D-Hiv-C2H), 3.36 (dd, *J* = 14.6, 4.9 Hz, 1H, Phe-C3H), 3.11 – 2.91 (m, 4H, Phe-C3H and Phe-NCH_3), 2.02 (hept., *J* = 6.7 Hz, 1H, D-Hiv-C3H), 0.80 (d, *J* = 6.7 Hz, 3H, D-Hiv-C4H_3), 0.43 (d, *J* = 6.7 Hz, 3H, D-Hiv-C4H_3); **^{13}C NMR** (151 MHz, $CDCl_3$) δ = 170.0 (quat., Phe-C1), 169.5 (quat., D-Hiv-C1), 136.8 (quat., Phe-C4), 129.0, 128.7, 126.9 (5 x CH, Phe-C5 – Phe-C7), 75.6 (CH, D-Hiv-C2), 57.5 (CH, Phe-C2), 34.9 (CH_2, Phe-C3), 32.5 (CH_3, Phe-NC), 29.8 (CH, D-Hiv-C3), 18.4, 17.6 (2 x CH_3, D-Hiv-C4); **IR** (neat/cm^{-1}):

2964, 2933, 1743, 1659, 1456, 1411, 1370, 1263, 1178, 1106, 1015, 731, 697; **HRMS (ESI)**: found 784.4183 $(M+H)^+$ $C_{45}H_{58}N_3O_9$ requires 784.4173 Δ = 1.3 ppm.

4.8.2 Method B (Flow)

According to General Method 2, a solution of ***protected precursor*** **10a** (0.050 g, 0.05 mmol) in dichloromethane (5 mL) was hydrogenated to give the acid and then the nitrogen acid deprotected (reaction time = 6 h) and the resultant crude product macrocyclised in flow. The crude product was purified using preparative TLC using toluene:ethyl acetate (2:1) as eluent to afford the *title compound* **1a** (0.030 g, 0.038 mmol, 76%) as a colourless solid, which was spectroscopically identical to that reported using method A.

4.9 –[Me-Phe-D-Hiv-Me-Phe-D-Hiv- Me-Phe-D-Hiv-Me-Phe-D-Hiv]- **2a**

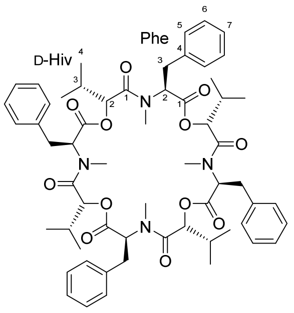

4.9.1 Method A (Batch)

Boc-Me-Phe-D-Hiv-Me-Phe-D-Hiv-Me-Phe-D-Hiv-Me-Phe-D-Hiv-OBn **11a** (0.086 g, 0.068 mmol) was taken up in methanol (5 mL) and dichloromethane (2 mL) and this solution was hydrogenanted using an H-cube® Pro (ThalesNano) with a 10% Pd/C CatCart®. The pump was run at 1 $mL.min^{-1}$ using methanol with the temperature set to 60 °C and the pressure to 6 bar. The solvent was then removed *in vacuo* and the crude acid was taken up in dioxane (1.5 mL) and anhydrous HCl (1.5 mL, 4 M in dioxane) was added. The reaction mixture was stirred at room temperature for 6 h 50 min before the solvent was removed *in vacuo*. The resultant crude was taken up successively in ethanol (2 x 2 mL) and methanol (2 mL) removing the solvent *in vacuo* after each addition. The resultant HCl salt was taken up in dichloromethane (10 mL) and was cooled to 0 °C before Ghosez reagent (0.01 mL, 0.075 mmol) was added and the reaction mixture was stirred at this temperature for 20 min. Diisopropylethylamine (0.042 mL, 0.24 mmol) was added and the reaction mixture was warmed to room temperature and stirred for 19.5 h before the solvent was removed *in vacuo*. The resultant oil was taken up in aqueous HCl (10 mL, 1M) and was extracted with ethyl acetate (3 x 15 mL). The combined organic extracts were washed with water (10 mL), saturated $NaHCO_3$ (10 mL) and saturated NaCl (10 mL) before drying over $MgSO_4$ and removing the solvent *in vacuo*. The resulting crude oil was purified using preparative TLC using toluene:ethyl acetate (1:1) as eluent to afford the *title compound* **2a** (0.021 g, 0.020 mmol, 30%) as a colourless solid.

$\mathbf{R_f}$ = 0.35 [toluene:diethyl ether = 1:1]; **mp** = 102 – 103 °C; $\boldsymbol{\alpha}_D^{27.8}$ = -20.2 (c 1.00, $CHCl_3$); **^{1}H NMR** (500 MHz, d_8-toluene, T = 363 °K) δ = 7.34 – 6.88 (m, 5H, Phe-C5H – Phe-C7H), 6.43 – 6.06 (m, 1H, Phe-C2H), 5.25 (d, J = 7.8 Hz, 1H, D-Hiv-C2H), 3.42 (dd, J = 14.8, 5.3 Hz, 1H, Phe-C3H*H*), 3.04 – 2.67 (m, 4H, Phe-C3H*H* and Phe-NCH_3), 2.24 – 2.09 (m, 1H, D-Hiv-C3H), 0.78 (d, J = 6.7 Hz, 3H, D-Hiv-C4H_3), 0.56 (d, J = 6.7 Hz, 3H, D-Hiv-C4H_3); **^{13}C NMR** (126 MHz, d_8-toluene, 363 °K) δ = 170.5 (quat., Phe-C1), 169.2 (quat., D-Hiv-C1), 137.4 (quat., Phe-C4), 129.5, 128.7, 126.8 (5 x CH, Phe-C5 – Phe-C7), 75.2 (CH, D-Hiv-C2), 57.3 (CH, Phe-C2), 34.9 (CH_2, Phe-C3), 31.2 (CH_3, Phe-NC), 30.4 (CH, D-Hiv-C3), 18.3,

18.1 (2 x CH_3, D-Hiv-C4); **IR** (neat/cm^{-1}): 2963, 2929, 1742, 1663, 1456, 1413, 1261., 1493, 1261, 1194, 1091, 1018, 745, 699; **HRMS (ESI)**: found 1045.5528 $(M+H)^+$ $C_{60}H_{77}N_4O_{12}$ requires 1045.5533 Δ = 0.5 ppm.

4.9.2 Method B (Flow)

According to General Method 2, a solution of ***protected precursor*** **11a** (0.063 g, 0.05 mmol) in methanol (5 mL) and dichloromethane (2 mL) and was hydrogenated to give the acid and then the nitrogen acid deprotected (reaction time = 6 h 25 min) and the resultant crude product macrocyclised in flow. The crude product was purified using preparative TLC using toluene:ethyl acetate (1:1) as eluent to afford the *title compound* **2a** (0.041 g, 0.040 mmol, 80%) as a colourless solid, which was spectroscopically identical to that reported using method A.

5 Synthesis of *N*-Me-Leu containing depsipeptides

5.1 Boc-Me-Leu-D-Hiv-OBn **6b**

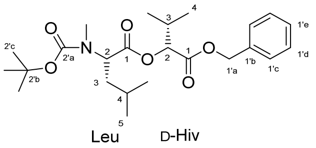

EDCI (0.37 g, 2.0 mmol) was added to a solution of D-Hiv-OBn **4** (2.14 g 10.3 mmol), Boc-*N*-methyl-L-leucine **5b** (3.78 g, 15.4 mmol) and 4-DMAP (1.63 g, 13.6 mmol) in dichloromethane (30 mL) at 0 °C and the resulting mixture was stirred at room temperature for 14 h. Aqueous HCl (30 mL, 1M) was added and the phases were separated. The aqueous layer was extracted with dichloromethane (3 x 30 mL) and the combined organic layers were washed with water (100 mL), saturated $NaHCO_3$ solution (100 mL) and saturated NaCl solution (100 mL) before drying over $MgSO_4$ and removing the solvent *in vacuo*. The crude product was purified *via* flash column chromatography using 40-60 petroleum ether:ethyl acetate (10:1) as eluent to afford the *title compound* **6b** (4.13 g, 9.5 mmol, 92%) as a colourless oil.

R_f = 0.45 [40-60 petroleum ether:diethyl ether = 10:1]; **$\alpha_D^{23.7}$** = -32.2 (c 1.00, $CHCl_3$); **^{1}H NMR** (500 MHz, d_6-DMSO, T = 393 °K) δ = 7.60 – 7.09 (m, 5H, D-Hiv-C1′cH – D-Hiv-C1′eH), 5.18 (d, *J* = 1.5 Hz, 2H, D-Hiv-C1′aH_2), 4.90 (d, *J* = 4.6 Hz, 1H, D-Hiv-C2H), 4.75 (dd, *J* = 8.8, 6.2 Hz, 1H, Leu-C2H), 2.73 (s, 3H, Leu-NCH_3), 2.22 (pd, *J* = 6.8, 4.6 Hz, 1H, D-Hiv-C3H), 1.70 – 1.65 (m, 2H, Leu-C3H_2), 1.65 – 1.54 (m, 1H, Leu-C4H), 1.43 (s, 9H, Leu-C2′cH_3), 1.01 – 0.87 (m, 12H,D-Hiv-C4H_3 and Leu-C5H_3); **^{13}C NMR** (126 MHz, d_6-DMSO, T = 393 °K) δ = 170.4 (quat., Leu-C1), 167.8 (quat., D-Hiv-C1), 154.4 (quat., Leu-C2′a), 134.9 (quat., D-Hiv-C1′b), 127.6, 127.4, 127.3 (5 x CH, D-Hiv-C1′c – D-Hiv-C1′e), 78.7 (quat., Leu-C2′b), 76.2 (CH, D-Hiv-C2), 65.7 (CH_2, D-Hiv-C1′a), 55.8 (CH, Leu-C2), 36.7 (CH, Leu-C3), 29.4 (CH_3, Leu-NC), 29.0 (CH, D-Hiv-C3), 27.4 (CH_3, Leu-C2′c), 23.9 (CH, Leu-C4), 21.8, 20.6 (CH_3, Leu-C5) , 17.5, 16.1 (CH_3, D-Hiv-C4); **IR** (neat/cm^{-1}): 2966, 2875, 1741, 1694, 1456, 1390, 1366, 1318, 1188, 1149, 1126, 1026, 1004, 906, 751, 697; **HRMS (ESI)**: found 436.2701 $(M+H)^+$ $C_{24}H_{38}NO_6$ requires 436.2699 Δ = 0.4 ppm.

5.2 Boc-Me-Leu-D-Hiv-Me-Leu-D-Hiv-OBn **8b**

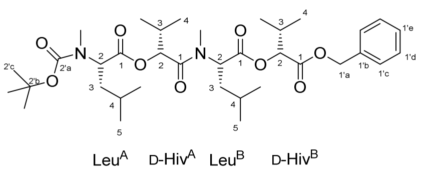

5.2.1 Method A (Batch)

Anhydrous HCl (2.5 mL, 4M in dioxane) was added to a solution of Boc-Me-Leu-D-Hiv-OBn **6b** (0.12 g, 0.26 mmol) in dioxane (2.5 mL) at room temperature and the resulting mixture was stirred for 3 h. The solvent was removed *in vacuo* and the residue was taken up sequentially in ethanol (2 x 5 mL) and methanol (5 mL) with the solvent removed *in vacuo* after each addition to afford the amine HCl salt which was used directly in the next step.

Hydrogen gas was bubbled through a solution of Boc-Me-Leu-D-Hiv-OBn **6b** (0.12 g, 0.26 mmol) and palladium (26 mg, 10% on charcoal, 26.4 µmol) in tetrahydrofuran (5 mL) for 3 min at room temperature before the resulting mixture was stirred under a hydrogen atmosphere for 3 h. The reaction mixture was filtered through celite, eluting with ethyl acetate (20 mL). The solvent was removed *in vacuo* from the filtrate to afford the acid which was used directly in the next step.

Ghosez reagent (38 µL, 0.29 mmol) was added to a solution of the acid in dichloromethane (5 mL) at 0 °C and the resulting mixture was stirred for 25 min. A solution of amine HCl salt and *N*,*N*-diisopropylethylamine (0.17 mL, 0.95 mmol) in dichloromethane (5 mL) was added, the reaction mixture was allowed to warm to room temperature and stirred for 22 h. Aqueous HCl (20 mL, 1M) was added, the reaction mixture was diluted with diethyl ether (40 mL) and the phases were separated. The aqueous layer was extracted with diethyl ether (3 x 20 mL) and the combined organic layers were washed with water (100 mL), saturated $NaHCO_3$ solution (100 mL) and saturated NaCl solution (100 mL) before drying over $MgSO_4$ and removing the solvent *in vacuo*. The crude product was purified *via* flash column chromatography using 40-60 petroleum ether:ethyl acetate (10:1 → 5:1) as eluent to afford the *title compound* **8b** (0.11 g, 0.17 mmol, 64%) as a colourless solid.

R_f = 0.12 [40-60 petroleum ether:diethyl ether = 10:1]; **mp** = 80 – 82 °C; **$\alpha_D^{23.8}$** = -33.0 (c 1.00, $CHCl_3$); **^{1}H NMR** (500 MHz, d_6-DMSO, T = 393 °K) *δ* = 7.42 – 7.30 (m, 5H, D-Hiv^B-C1′c$\underline{H}$ – D-Hiv^B-C1′e$\underline{H}$), 5.23 – 5.16 (m, 3H, D-Hiv^B-C1′a$\underline{H}_2$ and D-Hiv^B-C2$\underline{H}$), 5.03 (br. s, 1H, Leu^B-C2$\underline{H}$), 4.88 (d, *J* = 4.7 Hz, 1H, D-Hiv^A-C2$\underline{H}$), 4.73 (t, *J* = 7.7 Hz, 1H, Leu^A-C2$\underline{H}$), 2.83 (s, 3H, Leu^B-NC$\underline{H}_3$), 2.77 (s, 3H, Leu^A-NC$\underline{H}_3$), 2.21 (ddd, *J* = 13.7, 6.9, 4.8 Hz, 1H, D-Hiv^A-C3$\underline{H}$), 2.18 – 2.09 (m, 1H, D-Hiv^B-C3$\underline{H}$), 1.75 – 1.54 (m, 6H, Leu^{A+B}-C3$\underline{H}_2$ and Leu^{A+B}-C4$\underline{H}$), 1.43 (s, 9H, Leu^A-C2′c$\underline{H}_3$), 1.01 – 0.85 (m, 24H, D-Hiv^{A+B}-C4$\underline{H}_3$ and Leu^{A+B}-C5$\underline{H}_3$); **^{13}C NMR** (126 MHz, d_6-DMSO, T = 393 °K) *δ* = 170.2 (quat., Leu^A-$\underline{C}$1), 169.8 (quat., Leu^B-$\underline{C}$1), 168.3 (quat., D-Hiv^A-$\underline{C}$1), 167.8 (quat., D-Hiv^B-$\underline{C}$1), 154.5 (quat., Leu^A-$\underline{C}$2′a), 134.9 (quat., D-Hiv^B-$\underline{C}$1′b), 127.6, 127.4, 127.3 (5 x CH, D-Hiv^B-$\underline{C}$1′c – D-Hiv^B-$\underline{C}$1′e), 78.6 (quat., Leu^A-$\underline{C}$2′b), 76.6 (CH, D-Hiv^A-$\underline{C}$2), 74.6 (CH, D-Hiv^B-$\underline{C}$2), 65.7 (CH_2, D-Hiv^B-$\underline{C}$1′a), 55.8 (2 x CH, Leu^{A+B}-$\underline{C}$2), 36.8 (2 x CH_2, Leu^{A+B}-$\underline{C}$3), 29.4, 29.0 (2 x CH_3, Leu^{A+B}-N$\underline{C}$), 28.7 (2 x CH, D-Hiv^{A+B}-$\underline{C}$3), 27.4 (3 x CH_3, Leu^A-$\underline{C}$2′c), 23.9, 23.7 (2 x CH, Leu^{A+B}-$\underline{C}$4), 21.9, 21.8, 20.7, 20.6 (4 x CH_3, Leu^{A+B}-$\underline{C}$5), 17.9, 17.4, 16.3, 16.0 (4 x CH_3, Leu^{A+B}-$\underline{C}$5); **IR** (neat/cm^{-1}): 2961, 2873, 1737, 1694, 1666, 1456, 1390, 1367, 1318, 1258, 1236, 1189, 1149, 1125, 1089, 1023, 1004, 906, 752, 698 ; **HRMS (ESI)**: found 663.4215 $(M+H)^+$ $C_{36}H_{59}N_2O_9$ requires 663.4220 Δ = 0.8 ppm.

5.2.2 Method B (Flow)

According to General Method 1, a solution of ***coupling partner A*** **6b** (0.022 g, 0.05 mmol) in methanol (5 mL) was hydrogenated to give the acid and ***coupling partner B*** **6b** (0.022 g, 0.05 mmol) was deprotected using HCl (reaction time = 5 h) and the resultant crude products were coupled in flow. The product was purified using flash column chromatography using 40-60 petroleum ether:ethyl acetate (10:1) as eluent to afford the *title compound* **8b** (0.030 g, 0.045 mmol, 90%) as a colourless solid, which was spectroscopically identical to that reported using method A.

5.3 Boc-Me-Leu-D-Hiv-Me-Leu-D-Hiv-Me-Leu-D-Hiv-OBn **10b**

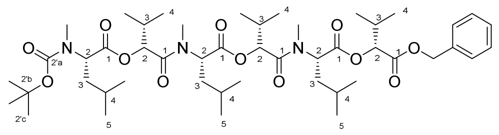

5.3.1 Method A (Batch)

Anhydrous HCl (3 mL, 4M in dioxane) was added to a solution of Boc-Me-Leu-D-Hiv-OBn **6b** (0.10 g, 0.15 mmol) in dioxane (3 mL) at room temperature and the resulting mixture was stirred for 6 h. The solvent was removed *in vacuo* and the residue was taken up sequentially in ethanol (2 x 4 mL) and methanol (4 mL) with the solvent removed *in vacuo* after each addition to afford the amine HCl salt which was used directly in the next step.

Hydrogen gas was bubbled through a solution of Boc-Me-Leu-D-Hiv-OBn **6b** (0.07 g, 0.15 mmol) and palladium (16 mg, 10% on charcoal, 0.015 mmol) in tetrahydrofuran (4 mL) for 5 min at room temperature before the resulting mixture was stirred under a hydrogen atmosphere for 3 h 15 min. The reaction mixture was filtered through cotton wool and syringe filter (0.2 μm) and the solvent was removed *in vacuo* from the filtrate to afford the acid which was used directly in the next step.

Ghosez reagent (0.022 mL, 0.17 mmol) was added to a solution of the acid in dichloromethane (5 mL) at 0 °C and the resulting mixture was stirred for 20 min. A solution of amine hydrochloride salt and *N*,*N*-diisopropylethylamine (0.094 mL, 0.54 mmol) in dichloromethane (5 mL) was added, the reaction mixture was allowed to warm to room temperature and stirred for 18 h. The solvent was removed *in vacuo* and aqueous HCl (5 mL, 1M) was added before the reaction mixture was extracted with ethyl acetate (3 x 15 mL) and the combined organic layers were washed with water (5 mL), saturated $NaHCO_3$ solution (5 mL) and saturated NaCl solution (5 mL) before drying over $MgSO_4$ and removing the solvent *in vacuo*. The crude product was purified *via* preparative TLC using 40-60 petroleum ether:ethyl acetate (2:1) as eluent to afford the *title compound* **10b** (0.10 g, 0.12 mmol, 78%) as a colourless foam.

R$_f$ = 0.67 [40-60 petroleum ether:diethyl ether = 2:1], 0.14 [40-60 petroleum ether:diethyl ether = 5:1]; **$\alpha_D^{27.7}$** = -56.2 (c 1.00, $CHCl_3$); **^{1}H NMR** (500 MHz, d_6-DMSO, T = 393 °K) δ = 7.58 – 7.16 (m, 5H, D-Hiv^D-C1′c<u>H</u> – D-Hiv^D-C1′e<u>H</u>), 5.24 – 5.15 (m, 4H, D-Hiv^C-C1′a<u>H</u>$_2$ and D-Hiv^{A+B}-C2<u>H</u>), 5.06 (br. s, 2H, Leu^{B+C}-C2<u>H</u>), 4.88 (br. s, 1H, D-Hiv^C-C2<u>H</u>), 4.74 (t, J = 8.0 Hz, 1H, Leu^A-C2<u>H</u>), 3.03 (br. s, 3H, Leu$^{B\text{ or }C}$-NC<u>H</u>$_3$), 2.96 (br. s, 3H, Leu$^{B\text{ or }C}$-NC<u>H</u>$_3$), 2.77 (s, 3H, Leu^A-NC<u>H</u>$_3$), 2.27 – 2.07 (m, 3H, D-Hiv^{A+B+C}-C3<u>H</u>), 1.86 – 1.65 (m, 6H, Leu^{A+B+C}-C3<u>H</u>$_2$), 1.62 – 1.51 (m, 3H, Leu^{A+B+C}-C4<u>H</u>), 1.43 (s, 9H, Leu^A-C2′c<u>H</u>$_3$), 1.02 – 0.88 (m, 36H, D-Hiv^{A+B+C}-C4<u>H</u>$_3$ and Leu^{A+B+C}-C5<u>H</u>$_3$); **^{13}C NMR** (126 MHz, d_6-DMSO, T = 393 °K) δ = 170.2 (quat., Leu^A-<u>C</u>1), 169.9, 169.7 (quat., Leu^{B+C}-<u>C</u>1), 168.4, 168.3 (quat., D-Hiv^{A+B}-<u>C</u>1), 167.8 (quat., D-Hiv^C-<u>C</u>1), 154.5 (quat., Leu^A-<u>C</u>2′a), 135.0 (quat., D-Hiv^D-<u>C</u>1′b), 127.7, 127.5, 127.4 (5 x CH, D-Hiv^C-<u>C</u>1′c – D-Hiv^C-<u>C</u>1′e), 78.6 (quat., Leu^A-<u>C</u>2′b), 76.6 (CH, D-Hiv^C-<u>C</u>2), 74.9, 74.7 (2 x CH, D-Hiv^{A+B}-<u>C</u>2), 65.7 (CH_2, D-Hiv^D-<u>C</u>1′a), 55.8 (CH, Leu^A-<u>C</u>2), 54. 5 (2 x CH, Leu^{B+C}-<u>C</u>2), 36.8 (CH_2, Leu^{A-C}-<u>C</u>3), 30.6 (2 x CH_3, Leu^{B+C}-N<u>C</u>), 29.4 (CH_3, Leu^A-N<u>C</u>), 29.0 (CH, D-Hiv^C-<u>C</u>3), 28.7 28.6, (2 x CH, D-Hiv^{A+B}-<u>C</u>3), 27.4 (3 x CH_3, Leu^A-<u>C</u>2′c), 23.9 (CH, Leu^A-<u>C</u>4), 23.8 (2 x CH, Leu^{B+C}-<u>C</u>4), 22.0, 21.9, 20.6, 18.0 (6 x CH_3, Leu^{A+B+C}-<u>C</u>5) , 17.9, 17.4, 16.3, 16.0 (6 x CH_3, D-Hiv^{A+B+C}-<u>C</u>4); **IR** (neat/cm^{-1}): 2960, 2873, 1737, 1695, 1662, 1470, 1390, 1368, 1262, 1203, 1150, 1125, 1020, 699; **HRMS (ESI)**: found 890.5738, $(M+H)^+$ $C_{48}H_{80}N_3O_{12}$ requires 890.5737 Δ = 0.1 ppm.

5.3.2 Method B (Flow)

According to General Method 1, a solution of ***coupling partner A*** **6b** (0.022 g, 0.05 mmol) in methanol (5 mL) was hydrogenated to give the acid and ***coupling partner B*** **8b** (0.033 g, 0.05 mmol) was deprotected using HCl (reaction time = 7 h 10 min) and the resultant crude products were coupled in flow. The crude product was purified using flash column chromatography using 40-60 petroleum ether:ethyl acetate (5:1) as eluent to afford the *title compound* **10b** (0.038 g, 0.043 mmol, 86%) as a colourless foam, which was spectroscopically identical to that reported using method A.

5.4 Boc-Me-Leu-D-Hiv-Me-Leu-D-Hiv-Me-Leu-D-Hiv-Me-Leu-D-Hiv-OBn **11b**

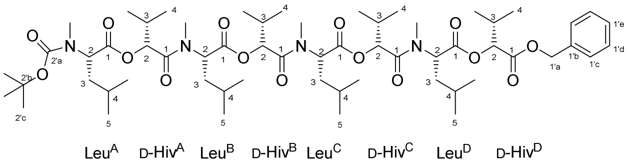

5.4.1 Method A (Batch)

Anhydrous HCl (2 mL, 4M in dioxane) was added to a solution of Boc-Me-Leu-D-Hiv-Me-Leu-D-Hiv-OBn **8b** (80 mg, 0.12 mmol) in dioxane (2 mL) at room temperature and the resulting mixture was stirred for 5 h. The solvent was removed *in vacuo* and the residue was taken up sequentially in ethanol (2 x 5 mL) and methanol (5 mL) with the solvent removed *in vacuo* after each addition to afford the HCl salt of the amine which was used directly in the next step.

Hydrogen gas was bubbled through a solution of Boc-Me-Leu-D-Hiv-Me-Leu-D-Hiv-OBn **8b** (80 mg, 0.12 mmol) and palladium (12 mg, 10% on charcoal, 12 µmol) in tetrahydrofuran (2 mL) for 3 min at room temperature before the mixture was stirred under a hydrogen atmosphere for 5.5 h. The reaction mixture was filtered through celite, eluting with ethyl acetate (20 mL). The solvent was removed *in vacuo* from the filtrate to afford the acid which was used directly in the next step.

Ghosez reagent (18 µL, 0.13 mmol) was added to a solution of the crude acid in dichloromethane (2.5 mL) at 0 °C and the resulting mixture was stirred for 25 min before a solution of crude amine and *N*,*N*-diisopropylethylamine (0.07 mL, 0.43 mmol) in dichloromethane (2.5 mL) was added and the reaction mixture was allowed to warm to room temperature and stirred for 16 h. Aqueous HCl (10 mL, 1M) was added, the reaction mixture was diluted with diethyl ether (20 mL) and the phases were separated. The aqueous layer was extracted with diethyl ether (3 x 10 mL) and the combined organic layers were washed with water (50 mL), saturated $NaHCO_3$ solution (50 mL) and saturated NaCl solution (50 mL) before drying over $MgSO_4$ and removing the solvent *in vacuo*. The crude product was purified *via* flash column chromatography using 40-60 petroleum ether:ethyl acetate (5:1 → 3:1) as eluent to afford the *title compound* **11b** (0.10 g, 91 µmol, 75%) as a colourless solid.

R_f = 0.12 [40-60 petroleum ether:ethyl acetate = 5:1]; **mp** = 124 – 125 °C; **$\alpha_D^{23.8}$** = -68.9 (c 1.00, $CHCl_3$); **^{1}H NMR** (500 MHz, d_6-DMSO, T = 393 °K) δ = 7.42 – 7.30 (m, 5H, D-Hiv^D-C1′cH – D-Hiv^D-C1′eH), 5.27 – 5.15 (m, 5H, D-Hiv^D-C1′aH_2 and D-Hiv$^{A\text{-}C}$-C2H), 5.02 (br. s, 3H, Leu$^{B\text{-}D}$-C2H), 4.88 (d, *J* = 4.6 Hz, 1H, D-Hiv^D-C2H), 4.78 – 4.70 (m, 1H, Leu^A-C2H), 3.14 – 2.94 (m, 9H, Leu$^{B\text{-}D}$-CNCH_3), 2.77 (s, 3H, Leu^A-NCH_3), 2.26 – 2.09 (m, 4H, D-Hiv$^{A\text{-}D}$-C3H), 1.76 – 1.62 (m, 8H, Leu$^{A\text{-}D}$-C3H_2), 1.63 – 1.52 (m, 4H, Leu$^{A\text{-}D}$-C4H), 1.43 (s, 9H, Leu^A-C2′cH_3), 0.99 – 0.87 (m, 48H, D-Hiv^{A+B+CD}-C4H_3 and Leu$^{A+B+C+D}$-C5H_3); **^{13}C NMR** (126 MHz, d_6-DMSO, T = 393 °K) δ = 170.4, 170.3, 170.0, 169.9 (4 x quat., Leu$^{A\text{-}D}$-C1), 168.9, 168.8, 168.7 (3 x quat., D-Hiv$^{A\text{-}C}$-C1), 168.2 (quat., D-Hiv^D-C1), 155.4 (quat., Leu^A-C2′a), 135.1 (quat., D-Hiv^D-

C1'b), 128.0, 127.8, 127.8 (5 x CH, D-Hiv^{D}-C1'c – D-Hiv^{D}-C1'e), 78.8 (quat., Leu^{A}-C2'b), 76.6 (CH, D-Hiv^{D}-C2), 75.07, 75.05, 74.8 (3 x CH, D-Hiv^{A-C}-C2), 66.0 (CH_2, D-Hiv^{D}-C1'a), 57.0 (CH, Leu^{A}-C2), 54.1 (3 x CH, Leu^{B-D}-C2), 36.8, 36.5, 36.3 (4 x CH_2, Leu^{A-D}-C3), 30.8, 30.8, 30.7, 30.4 (4 x CH_3, Leu^{A-D}-NC), 29.5, 29.3, 28.8, 28.7 (4 x CH, D-Hiv^{A-D}-C3), 27.7 (3 x CH_3, Leu^{A}-C2'c), 24.1, 24.0, 23.9 (4 x CH, Leu^{A-D}-C4), 22.5, 22.3, 22.0, 20.74, 20.67, 20.64 (8 x CH_3, Leu^{A-D}-C5), 18.8, 18.3, 18.20, 18.18, 17.9, 17.8, 16.5, 16.2, 16.0 (8 x CH_3, D-Hiv^{A-D}-C4); **IR** (neat/cm^{-1}): 2960, 2932, 2873, 1736, 1695, 1659, 1469, 1390, 1368, 1263, 1192, 1125, 1088, 1020, 698; **HRMS (ESI)**: found 1139.7107 $(M+Na)^+$ $C_{60}H_{100}N_4O_{15}Na$ requires 1139.7083 Δ = 2.1 ppm.

5.4.2 Method B (Flow)

According to General Method 1, a solution of ***coupling partner A*** **8b** (0.033 g, 0.05 mmol) in methanol (5 mL) was hydrogenated to give the acid and ***coupling partner B*** **8b** (0.033 g, 0.05 mmol) was acid deprotected (reaction time = 5 h 30 min) and the resultant crude products were coupled in flow. The crude product was purified using flash column chromatography using 40-60 petroleum ether:ethyl acetate (5:1) as eluent to afford the *title compound* **11b** (0.042 g, 0.038 mmol, 76%) as a colourless solid, which was spectroscopically identical to that reported using method A.

5.5 -[Me-Leu-D-Hiv-Me-Leu-D-Hiv]- **9b**

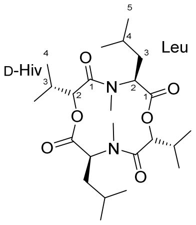

5.5.1 Method A (Batch)

Boc-Me-Leu-D-Hiv-Me-Leu-D-Hiv-OBn **8b** (0.050 g, 0.075 mmol) was taken up in dichloromethane (1.5 mL) and this solution was hydrogenated using an H-cube® Pro (ThalesNano) with a 10% Pd/C CatCart®. The pump was run at 1 mL.min^{-1} using dichloromethane with the temperature set to 60 °C and the pressure to 6 bar. The solvent was then removed *in vacuo* and the crude acid was taken up in dioxane (1 mL) and anhydrous HCl (1 mL, 4 M in dioxane) was added. The reaction mixture was stirred at room temperature for 5 h 25 min before the solvent was removed *in vacuo*. The resultant crude was taken up successively in ethanol (2 x 5 mL) and methanol (5 mL) removing the solvent *in vacuo* after each addition. The resultant HCl salt was taken up in dichloromethane (0.5 mL) and was cooled to 0 °C before Ghosez reagent (0.011 mL, 0.083 mmol) was added and the reaction mixture was stirred at this temperature for 15 min. Diisopropylethylamine (0.05 mL, 0.27 mmol) was added and the reaction mixture was warmed to room temperature and stirred for 20 h before the solvent was removed *in vacuo*. The resultant oil was taken up in aqueous HCl (6 mL, 1M) and was extracted with diethyl ether (3 x 6 mL). The combined organic extracts were washed with water (6 mL), saturated $NaHCO_3$ solution (6 mL) and saturated NaCl solution (6 mL) before drying over $MgSO_4$ and removing the solvent *in vacuo*. The resulting crude oil was purified using flash column chromatography using 2:1 toluene:ethyl acetate as eluent to afford the *title compound* **9b** (0.0065 g, 0.014 mmol, 19%) as a colourless solid and *cyclic tetramer* **2b** (0.015 g, 0.017 mmol, 45%) as a colourless solid which was spectroscopically identical to that reported for this compound below.

R_f = 0.36 [toluene:ethyl acetate= 2:1]; **mp** = 155.5 – 157 °C (literature = 156 – 1578 °C[11]); **$\alpha_D^{27.8}$** = +34.4 (c 0.90 $CHCl_3$), literature[11] = **α_D^{20}** = +35 (c 1.0 $CHCl_3$); **^{1}H NMR** (600 MHz, $CDCl_3$) δ = 4.90 (d, *J* =

9.9 Hz, 1H, D-Hiv-C2H), 4.82 (dd, *J* = 8.9, 6.7 Hz, 1H, Leu-C2H), 3.02 (s, 3H, Leu-NCH_3), 2.09 (dt, *J* = 9.9, 6.7 Hz, 1H, D-Hiv-C3H), 1.92 (ddd, *J* = 13.9, 8.9, 5.3 Hz, 1H, Leu-C3H*H*), 1.58 – 1.52 (m, 1H, Leu-C4H), 1.49 (ddd, *J* = 13.4, 8.2, 6.7 Hz, 1H, Leu-C3H*H*), 1.11 – 0.98 (m, 12H, D-Hiv-C4H_3 and Leu-C5H_3); **^{13}C NMR** (151 MHz, $CDCl_3$) δ = 170.3 (quat., Leu-C1), 168.5 (quat., D-Hiv-C1), 83.3 (CH, D-Hiv-C2), 56.3 (CH, Leu-C2), 39.7 (CH_2, Leu-C3), 30.5 (CH, D-Hiv-C3), 29.9 (CH_3, Leu-NC), 25.0 (CH, Leu-C4), 23.1, 22.1 (2 x CH_3, Leu-C5), 19.9, 18.6 (2 x CH_3, D-Hiv-C4); **IR** (neat/cm^{-1}): 2957, 2937, 2875, 1750, 1655, 1468, 1369, 1175, 1147, 1001; **HRMS (ESI)**: found 455.3113 $(M+H)^+$ $C_{24}H_{43}N_2O_6$ requires 455.3116 Δ = 0.6 ppm.

5.5.2 Method B (Flow)

According to General Method 2, a solution of ***protected precursor*** **8b** (0.033 g, 0.05 mmol) in methanol (5 mL) and was hydrogenated to give the acid and then the nitrogen acid deprotected (reaction time = 5 h 35 min) and the resultant crude product macrocyclised in flow. The crude product was purified using preparative TLC using toluene:ethyl acetate (1:1) as eluent to afford the *title compound* **9b** (0.020 g, 0.043 mmol, 86%) as a colourless solid, which was spectroscopically identical to that reported using method A.

5.6 -[Me-Leu-D-Hiv-Me-Leu-D-Hiv-Me-Leu-D-Hiv]- **1b** (enniatin C)

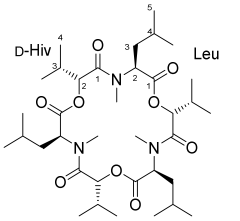

5.6.1 Method A (Batch)

Boc-Me-Leu-D-Hiv-Me-Leu-D-Hiv-Me-Leu-D-Hiv-OBn **10b** (0.23 g, 0.26 mmol) was taken up in methanol (30 mL) and this solution was hydrogenanted using an H-cube® Pro (ThalesNano) with a 10% Pd/C CatCart®. The pump was run at 1 mL.min^{-1} using dichloromethane with the temperature set to 60 °C and the pressure to 6 bar. The solvent was then removed *in vacuo* and the crude acid was taken up in dioxane (7 mL) and anhydrous HCl (7 mL, 4 M in dioxane) was added. The reaction mixture was stirred at room temperature for 6 h before the solvent was removed *in vacuo*. The crude was taken up successively in ethanol (2 x 7 mL) and methanol (7 mL) removing the solvent *in vacuo* after each addition. The resultant HCl salt was taken up in dichloromethane (20 mL) and was cooled to 0 °C before Ghosez reagent (0.038 mL, 0.29 mmol) was added and the reaction mixture was stirred at this temperature for 30 min. Diisopropylethylamine (0.16 mL, 0.94 mmol) was added and the reaction mixture was warmed to room temperature and stirred for 18 h before the solvent was removed *in vacuo*. The resultant oil was taken up in aqueous HCl (20 mL, 1M) and was extracted with ethyl acetate (3 x 20 mL). The combined organic extracts were washed with water (20 mL), saturated $NaHCO_3$ solution (20 mL) and saturated NaCl solution (20 mL) before drying over $MgSO_4$ and removing the solvent *in vacuo*. The resulting crude oil was purified using flash column chromatography using 1:2 40-60 petroleum ether:ethyl acetate as eluent to afford the *title compound* **1b** (0.075 g, 0.11 mmol, 42%) as a colourless solid.

R_f = 0.13 [40-60 petroleum ether:ethyl acetate= 1:2]; **mp** = 166 – 167 °C (literature = 129 °C[12]); **$\alpha_D^{27.8}$** = -21.8 (c 1.00, $CHCl_3$) literature[12] $\alpha_D^{20.0}$ = -24 (c 0.1, $CHCl_3$); **^{1}H NMR** (600 MHz, $CDCl_3$) δ = 5.25 (dd, *J* = 11.3, 4.9 Hz, 1H, Leu-C2H), 4.93 (d, *J* = 8.6 Hz, 1H, D-Hiv-C2H), 3.09 (s, 3H, Leu-NCH_3), 2.30 –

2.17 (m, 1H, D-Hiv-C3H), 1.72 (ddd, *J* = 15.3, 11.3, 4.3 Hz, 1H, Leu-C3H*H*), 1.65 (ddd, *J* = 14.3, 10.0, 4.9 Hz, 1H, Leu-C3H*H*), 1.53 – 1.38 (m, 1H, Leu-C4H), 1.02 – 0.86 (m, 12H, D-Hiv-C4H_3 and Leu-C5H_3); **^{13}C NMR** (151 MHz, $CDCl_3$) δ = 171.0 (quat., Leu-C1), 169.9 (quat., D-Hiv-C1), 75.6 (CH, D-Hiv-C2), 54.4 (CH, Leu-C2), 37.6 (CH_2, Leu-C3), 31.8 (CH_3, Leu-NC), 30.1 (CH, D-Hiv-C3), 25.3 (CH, Leu-C4), 23.5, 21.3 (2 x CH_3, Leu-C5), 18.8, 18.2 (2 x CH_3, D-Hiv-C4); **IR** (neat/cm^{-1}): 2965, 2929, 2869, 1746, 1662, 1470, 1413, 1370, 1266, 1178, 1129, 1010; **HRMS (ESI)**: found 682.4635 $(M+H)^+$ $C_{36}H_{64}N_3O_9$ requires 682.4637 Δ = 0.33 ppm.

5.6.2 Method B (Flow)

According to General Method 2, a solution of ***protected precursor*** **10b** (0.044 g, 0.05 mmol) in methanol (5 mL) was hydrogenated to give the acid and then the nitrogen acid deprotected (reaction time = 6 h) and the resultant crude product macrocyclised in flow. The resulting crude oil was purified using flash column chromatography using 1:2 40-60 petroleum ether:ethyl acetate as eluent to afford the *title compound* **1b** (0.029 g, 0.042 mmol, 84%) as a colourless solid, which was spectroscopically identical to that reported using method A.

5.7 -[Me-Leu-D-Hiv-Me-Leu-D-Hiv-Me-Leu-D-Hiv-Me-Leu- D-Hiv]- **2b** (bassianolide)

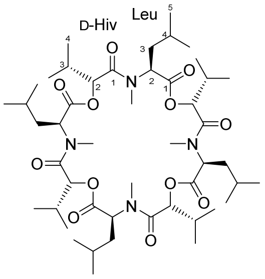

5.7.1 Method A (Batch)

Hydrogen gas was bubbled through a solution of Boc-Me-Leu-D-Hiv-Me-Leu-D-Hiv-Me-Leu-D-Hiv-Me-Leu-D-Hiv-OBn **11b** (102 mg, 91 µmol) and palladium (10 mg, 10% on charcoal, 9.1 µmol) in tetrahydrofuran (2 mL) for 3 min at room temperature before the mixture was stirred under a hydrogen atmosphere for 6 h. The reaction mixture was filtered through celite, eluting with ethyl acetate (30 mL). The solvent was removed *in vacuo* from the filtrate to afford crude acid as a colourless oil that was taken up in dioxane (2 mL). HCl (2 mL, 4M in dioxane) was added and the resulting mixture was stirred for 5 h. The solvent was removed *in vacuo* and the residue was taken up sequentially in ethanol (2 x 5 mL) and methanol (5 mL) removing the solvent *in vacuo* after each addition to afford the deprotected linear precursor. This was taken up in dichloromethane (20 mL) and cooled to 0 °C before Ghosez reagent (13 µL, 100 µmol) was added. The resulting mixture was stirred for 30 min at 0 °C before *N*,*N*-diisopropylethylamine (55 µL, 326 µmol) was added and the reaction mixture was warmed to room temperature and stirred for 18 h. The solvent was removed *in vacuo* and the residue was taken up in diethyl ether (60 mL) and aqueous HCl (60 mL, 1M). The aqueous layer was extracted with diethyl ether (3 x 30 mL) and the combined organic layers were washed with water (100 mL), saturated $NaHCO_3$ solution (100 mL) and saturated NaCl solution (100 mL) before drying over $MgSO_4$ and removing the solvent *in vacuo*. The crude product was purified *via* flash column chromatography using 40-60 petroleum ether:ethyl acetate (3:1 → 2:1) as eluent followed by preparatory TLC using 40-60 petroleum ether:ethyl acetate (2:1) as eluent to afford the *title compound* **2b** (56 mg, 61 µmol, 67%) as a colourless solid. The ^{1}**R*f*** = 0.17 [40-60 petroleum ether:ethyl acetate= 2:1]; **mp** = 100 –

101 °C; **$\alpha_D^{24.6}$** = -56.3 (c 3.3 $CHCl_3$) literature[14] $\alpha_D^{22.0}$ = -69 (c 3.3, $CHCl_3$); **^{1}H NMR** (500 MHz, d_8-toluene, T = 363 °K) δ = 5.84 (br. s, 1H, Leu-C2H), 5.37 (d, J = 7.1 Hz, 1H, D-Hiv-C2H), 2.83 (s, 3H, Leu-NCH$_3$), 2.35 (br. s, 1H, D-Hiv-C3H), 1.96 – 1.81 (m, 1H, Leu-C3H*H*), 1.75 – 1.59 (m, 1H, Leu-C3H*H*), 1.51 (s, 1H, Leu-C4H), 1.01 – 0.85 (m, 12H, D-Hiv-C4H$_3$ and Leu-C5H$_3$); **^{13}C NMR** (126 MHz, d_8-toluene, T = 363 °K) δ = 171.5 (quat., Leu-C1), 169.7 (quat., D-Hiv-C1), 75.5 (CH, D-Hiv-C2), 55.4 (CH, Leu-C2), 37.9 (CH_2, Leu-C3), 31.1 (CH_3, Leu-NC), 30.9 (CH, D-Hiv-C3), 26.0 (CH, Leu-C4), 23.7, 22.1 (2 x CH_3, D-Hiv-C4), 19.1, 18.4 (2 x CH_3, Leu-C5); **IR** (neat/cm^{-1}): 2959, 2873, 1740, 1660, 1469, 1412, 1388, 1262, 1191, 1125, 1087, 1009, 758; **HRMS (ESI)**: found 931.6010 $(M+Na)^+$ $C_{48}H_{84}O_{12}N_4Na$ requires 931.5983 Δ = 2.9 ppm.

5.7.2 Method B (Flow)

According to General Method 2, a solution of ***protected precursor*** **11b** (0.056 g, 0.05 mmol) in methanol (5 mL) and was hydrogenated to give the acid and then the nitrogen acid deprotected (reaction time = 6 h 5 min) and the resultant crude product macrocyclised in flow. The crude product was purified using flash column chromatography using toluene:ethyl acetate (2:1) as eluent to afford the *title compound* **2b** (0.042 g, 0.046 mmol, 93%) as a colourless solid, which was spectroscopically identical to that reported using method A.

6 References

[1] http://www.vapourtec.co.uk/products/rseriessystem/reactors.
[2] http://syrris.com/flow-products/asia-flow-chemistry.
[3] http://www.cambridgereactordesign.com/polarbearplus/polar-bear-plus.html.
[4] K. Hioki, S. Kameyama, S. Tani, M. Kunishima, *Chem. Pharm. Bull.* **2007**, *55*, 825-828.
[5] D. X. Hu, M. O'Brien, S. V. Ley, *Org. Lett.* **2012**, *14*, 4246-4249.
[6] S. C. Feifel, T. Schmiederer, T. Hornbogen, H. Berg, R. D. Sussmuth, R. Zocher, *ChemBioChem* **2007**, *8*, 1767-1770.
[7] A. Agarkov, S. J. Greenfield, T. Ohishi, S. E. Collibee, S. R. Gilbertson, *J. Org. Chem.* **2004**, *69*, 8077-8085.
[8] T. P. Kogan, T. C. Somers, M. C. Venuti, *Tetrahedron* **1990**, *46*, 6623-6632.
[9] J. X. Zhan, A. M. Burns, M. P. X. Liu, S. H. Faeth, A. A. L. Gunatilaka, *J. Nat. Prod.* **2007**, *70*, 227-232.
[10] Y. A. Ovchinnikov, V. T. Ivanov, I. I. Mikhaleva, *Tetrahedron Lett.* **1971**, 159-162.
[11] M. M. Shemyakin, Y. A. Ovchinnikov, V. T. Ivanov, A. A. Kiryushkin, *Tetrahedron* **1963**, *19*, 581-591.
[12] I. I. Mikhaleva, I. D. Ryabova, T. A. Romanova, T. I. Tarasova, V. T. Ivanov, Y. A. Ovchinnikov, M. M. Shemyakin, *Zh. Obsch. Khim.* **1968**, *38*, 1228 -1239.
[13] M. Kanaoka, A. Isogai, S. Murakoshi, M. Ichinoe, A. Suzuki, S. Tamura, *Agr. Biol. Chem. Tokyo.* **1978**, *42*, 629-635.
[14] M. Kanaoka, A. Isogai, A. Suzuki, *Tetrahedron Lett.* **1977**, 4049-4050.

7 NMR Spectra

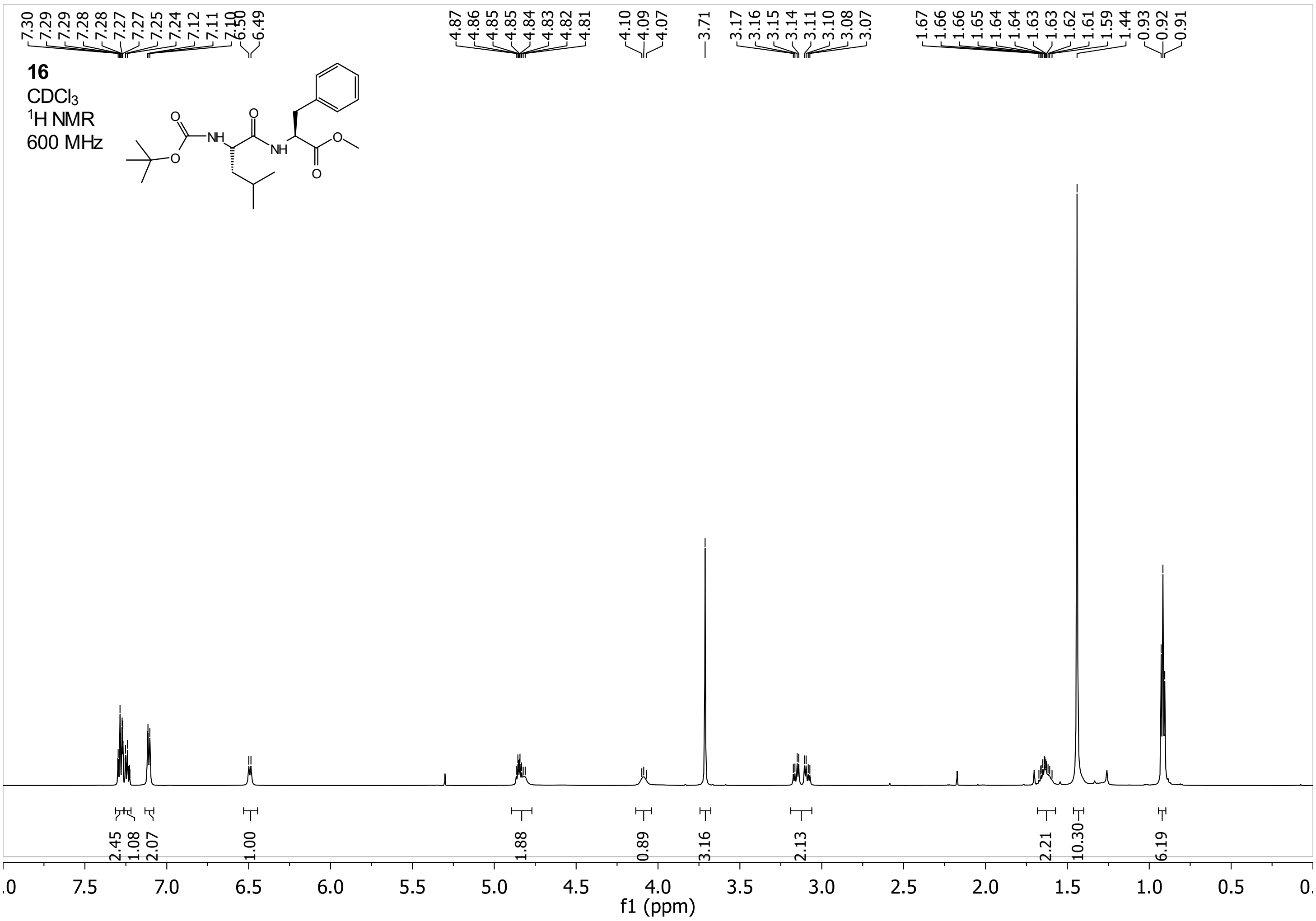
16
CDCl3
1H NMR
600 MHz
7.30 7.29 7.29 7.28 7.28 7.27 7.27 7.25 7.24 7.12 7.11 7.10 6.50 6.49
4.87 4.86 4.85 4.85 4.84 4.83 4.82 4.81
4.10 4.09 4.07
3.71
3.17 3.16 3.15 3.14 3.11 3.10 3.08 3.07
1.67 1.66 1.66 1.65 1.64 1.64 1.63 1.63 1.62 1.61 1.59 1.44 0.93 0.92 0.91
2.45 1.08 2.07 1.00 1.88 0.89 3.16 2.13 2.21 10.30 6.19
f1 (ppm)

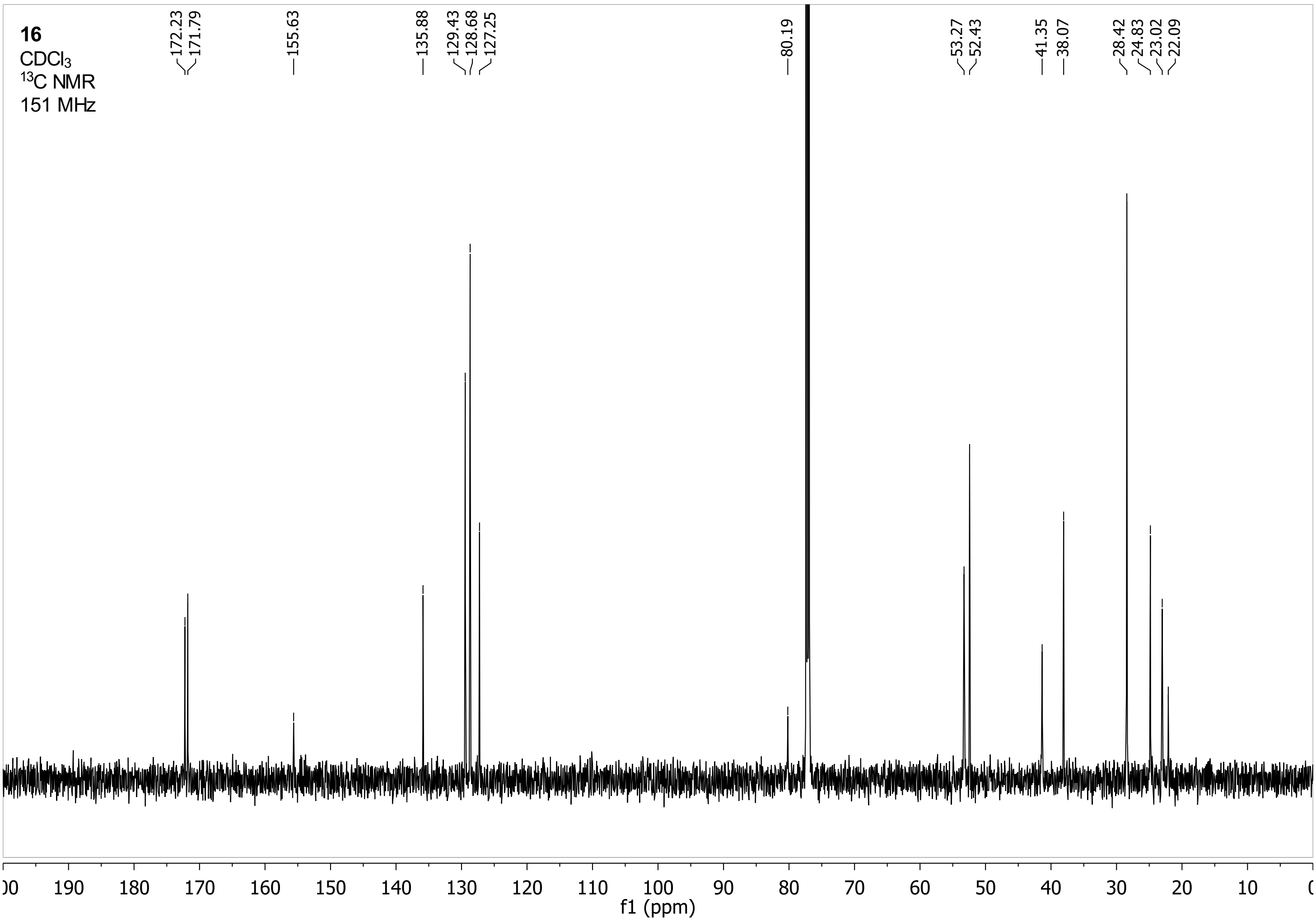
16
CDCl3
13C NMR
151 MHz
172.23 171.79
155.63
135.88
129.43 128.68 127.25
80.19
53.27 52.43
41.35 38.07
28.42 24.83 23.02 22.09
f1 (ppm)

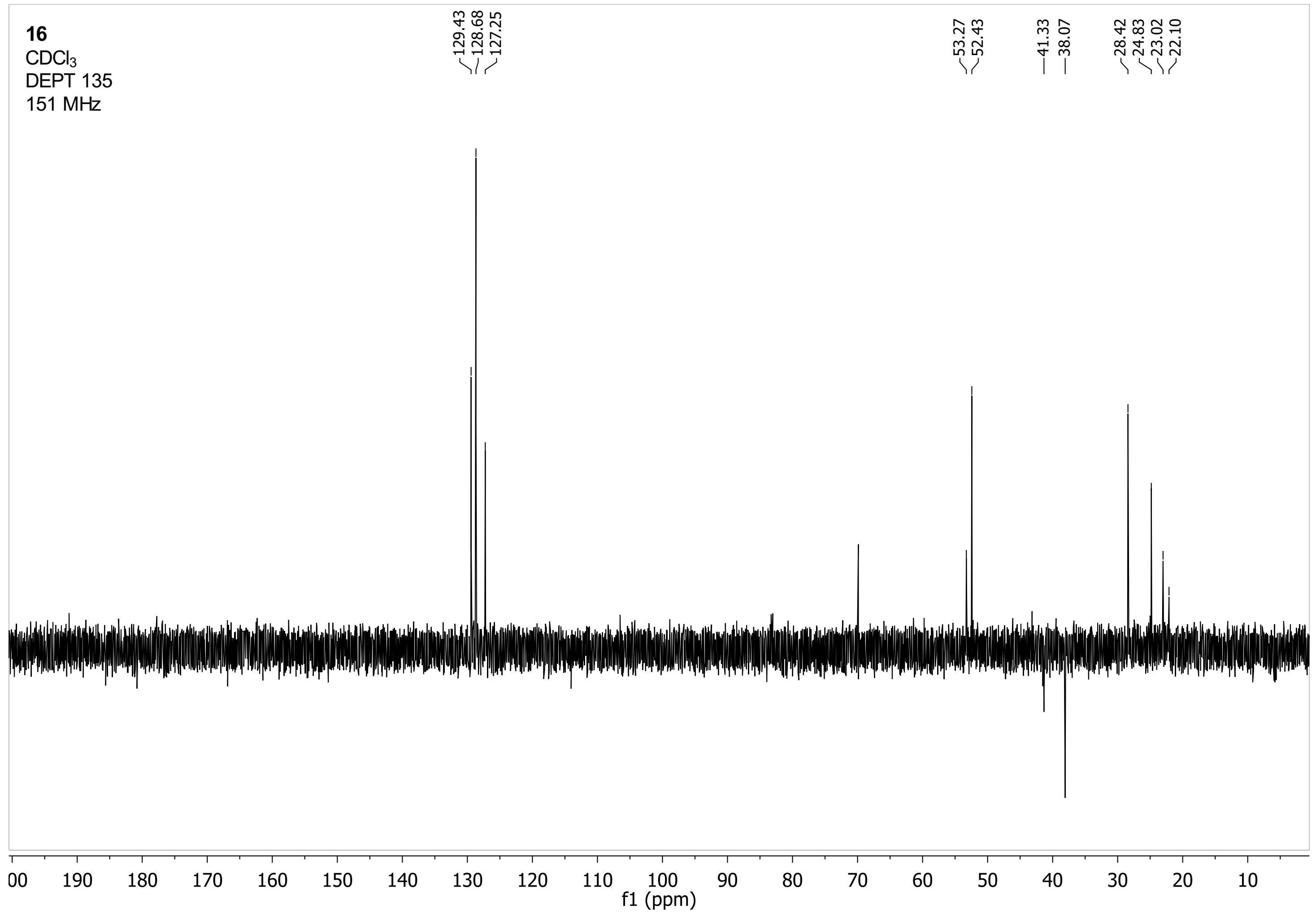
16
CDCl3
DEPT 135
151 MHz
129.43
128.68
127.25
53.27
52.43
41.33
38.07
28.42
24.83
23.02
22.10
f1 (ppm)

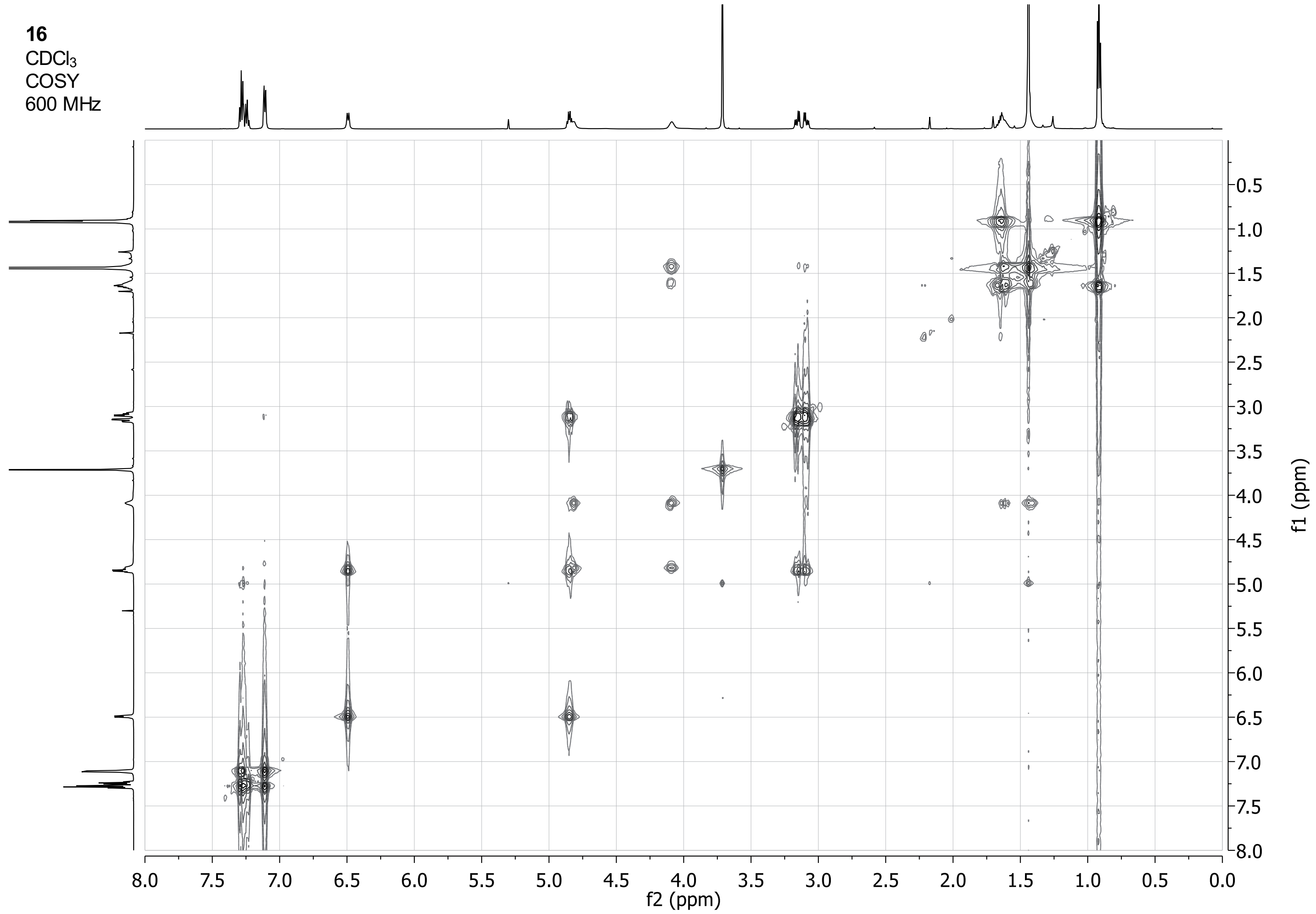
16
CDCl3
COSY
600 MHz
f2 (ppm)
f1 (ppm)

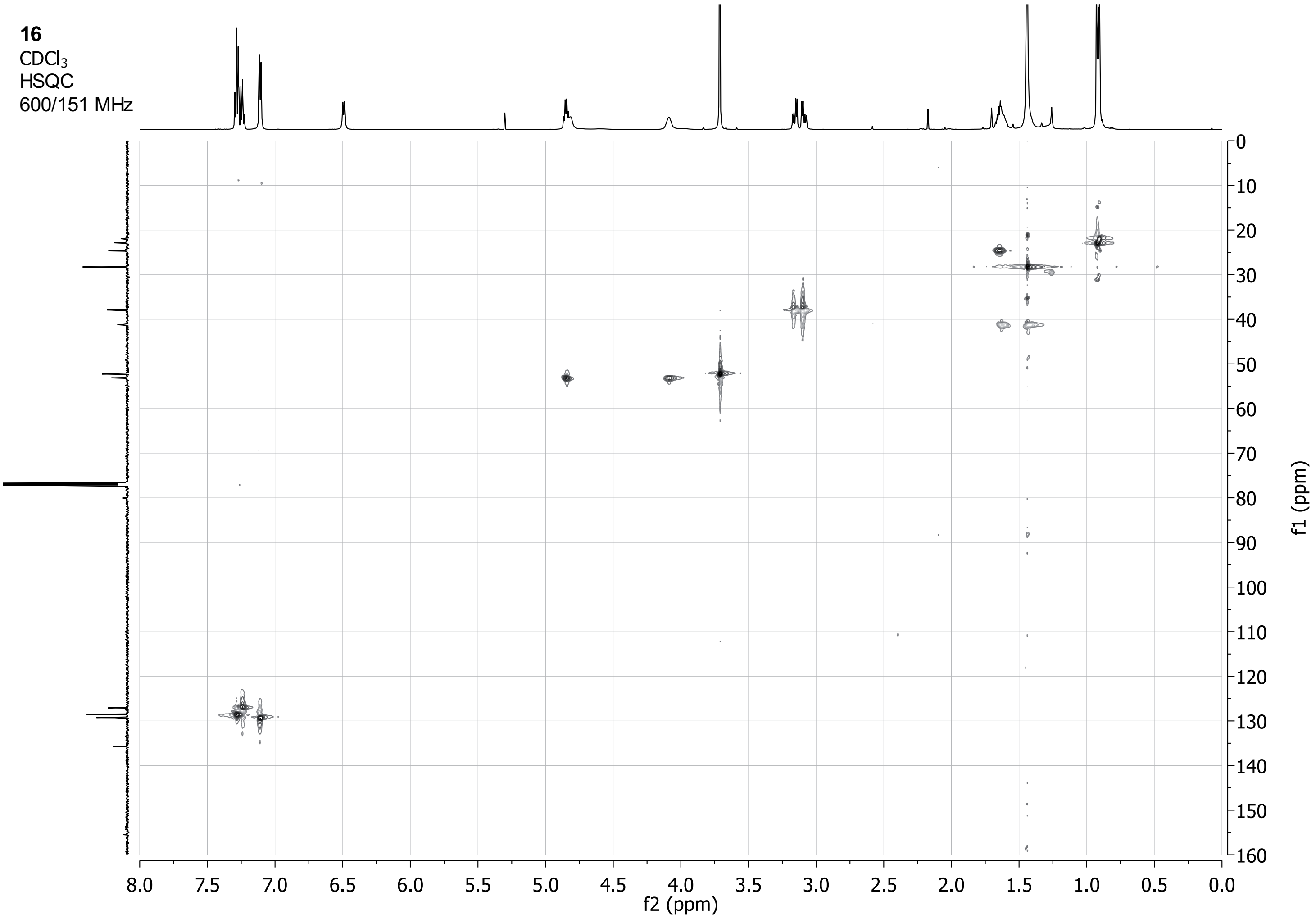
16
CDCl3
HSQC
600/151 MHz
f2 (ppm)
f1 (ppm)

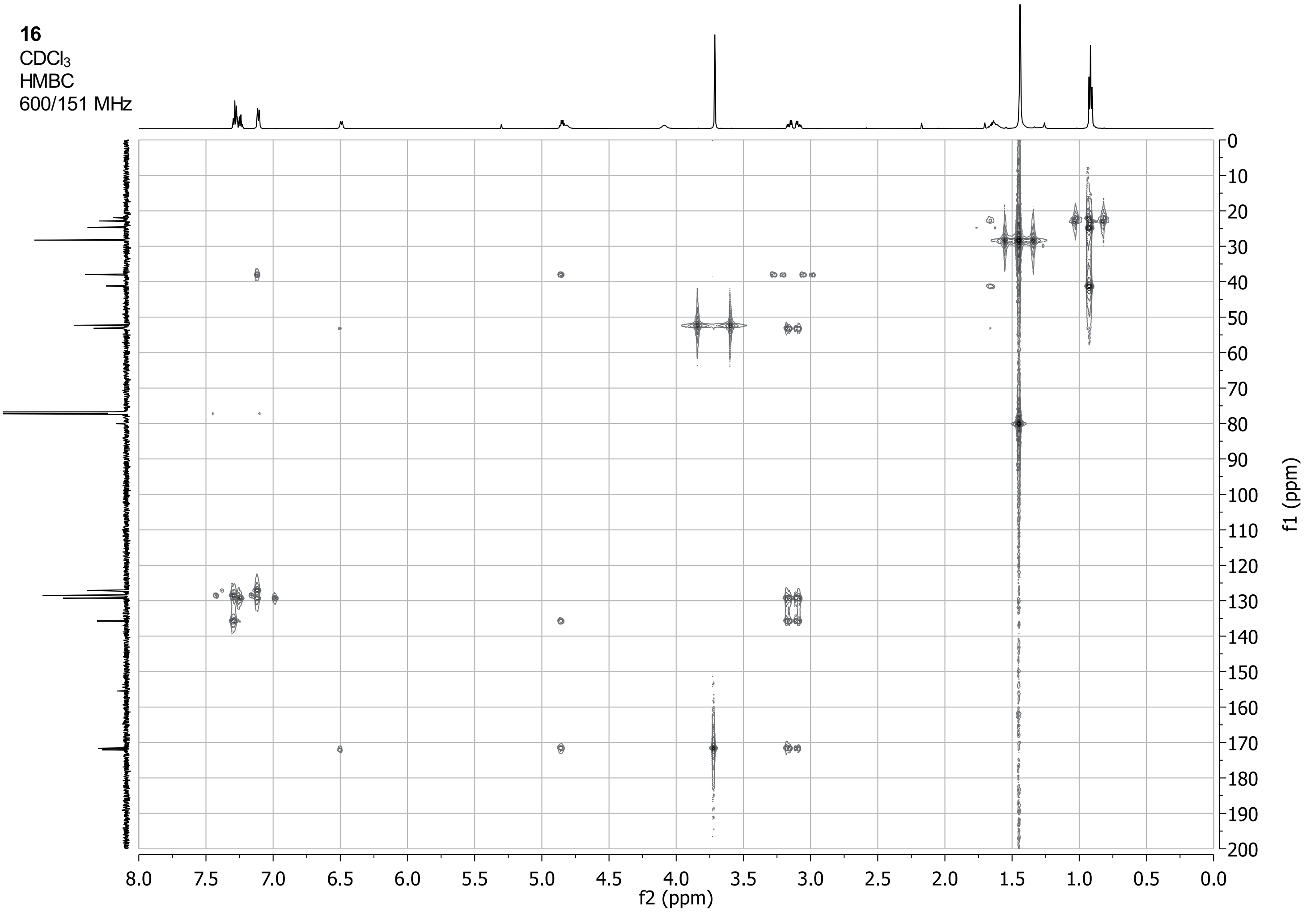
16
CDCl3
HMBC
600/151 MHz
f2 (ppm)
f1 (ppm)

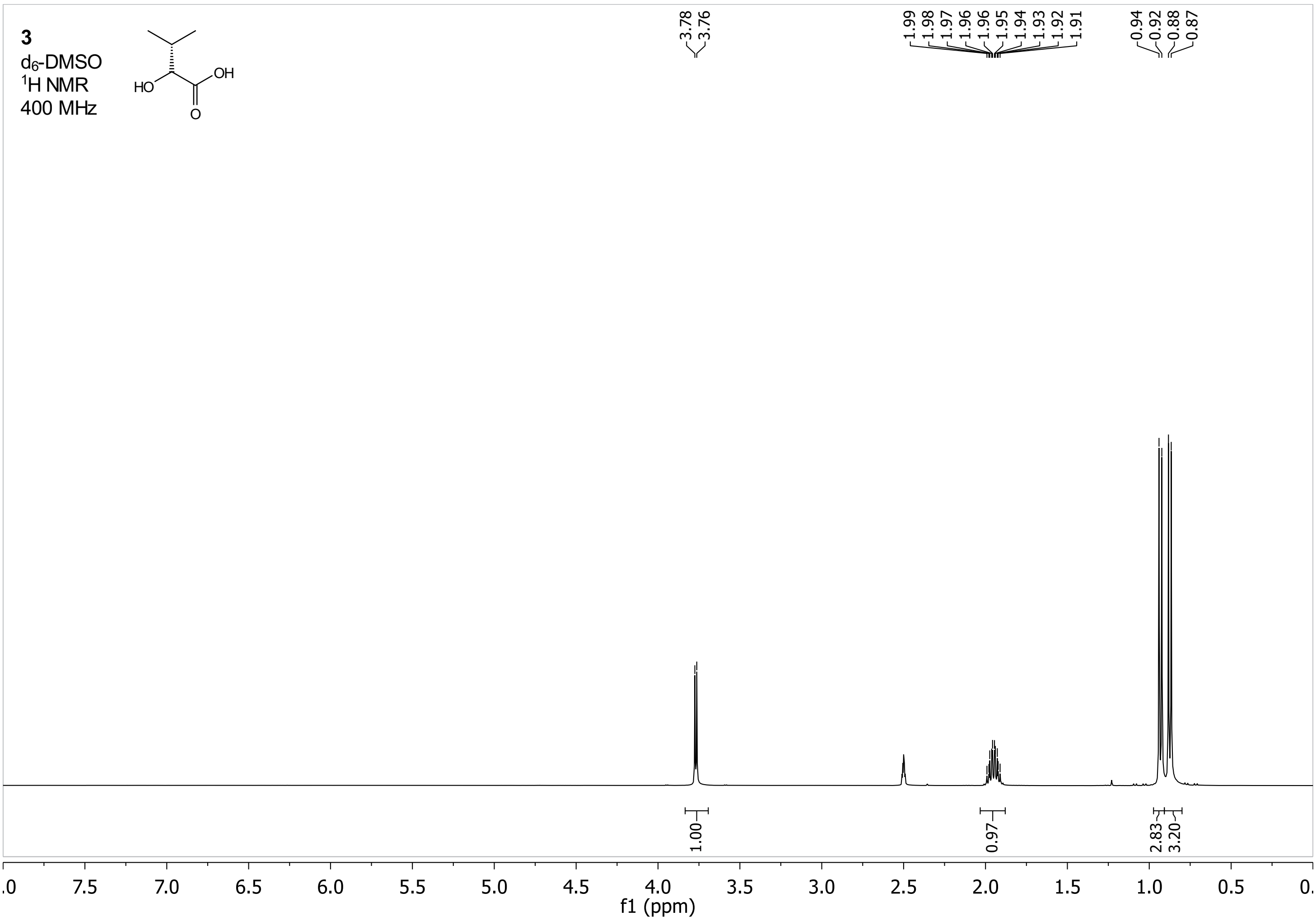
3
d6-DMSO
1H NMR
400 MHz
HO
OH
O
3.78
3.76
1.99
1.98
1.97
1.96
1.96
1.95
1.94
1.93
1.92
1.91
0.94
0.92
0.88
0.87
1.00
0.97
2.83
3.20
7.5
7.0
6.5
6.0
5.5
5.0
4.5
4.0
3.5
3.0
2.5
2.0
1.5
1.0
0.5
f1 (ppm)

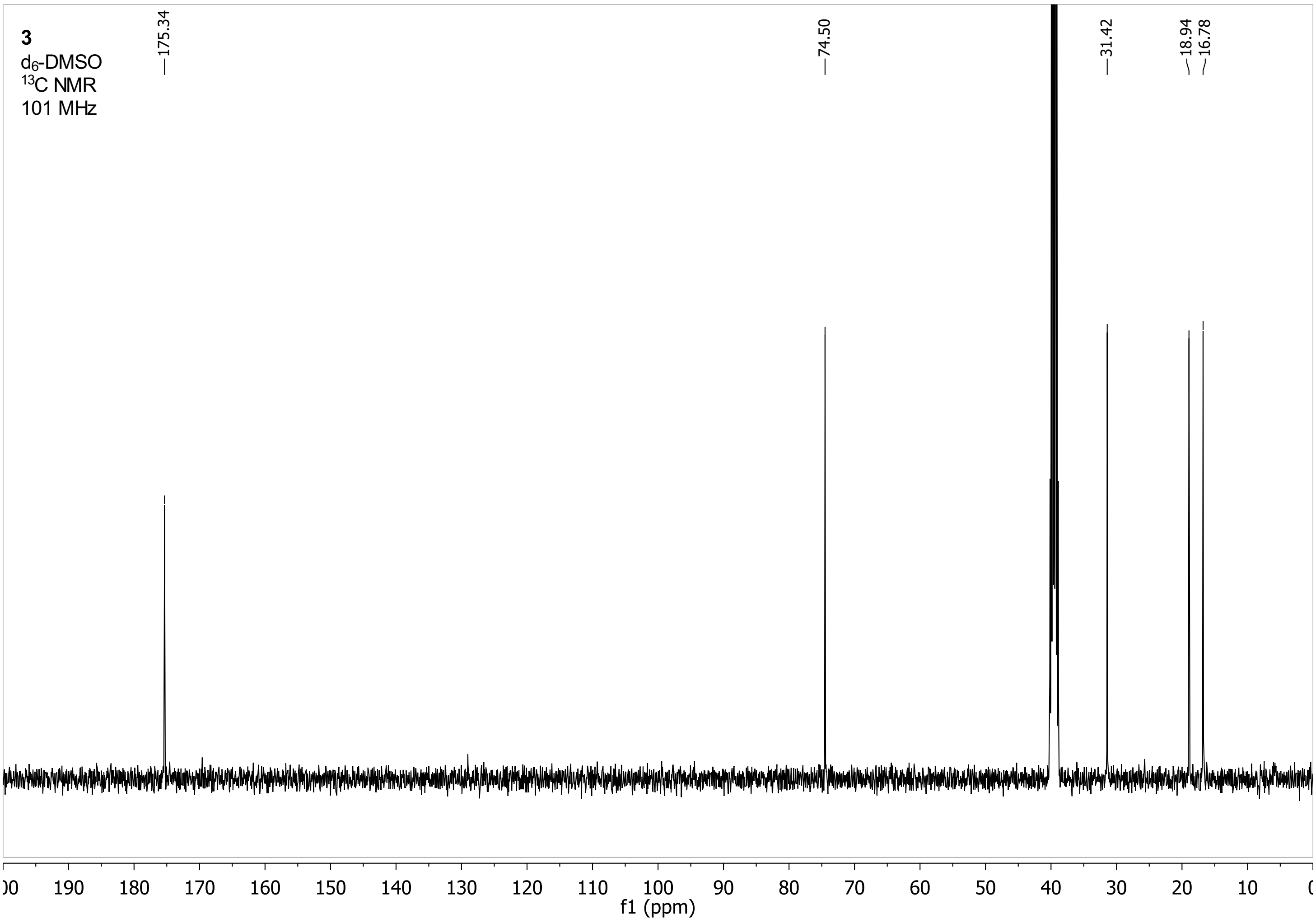
3
d6-DMSO
13C NMR
101 MHz
175.34
74.50
31.42
18.94
16.78
190
180
170
160
150
140
130
120
110
100
90
80
70
60
50
40
30
20
10
f1 (ppm)

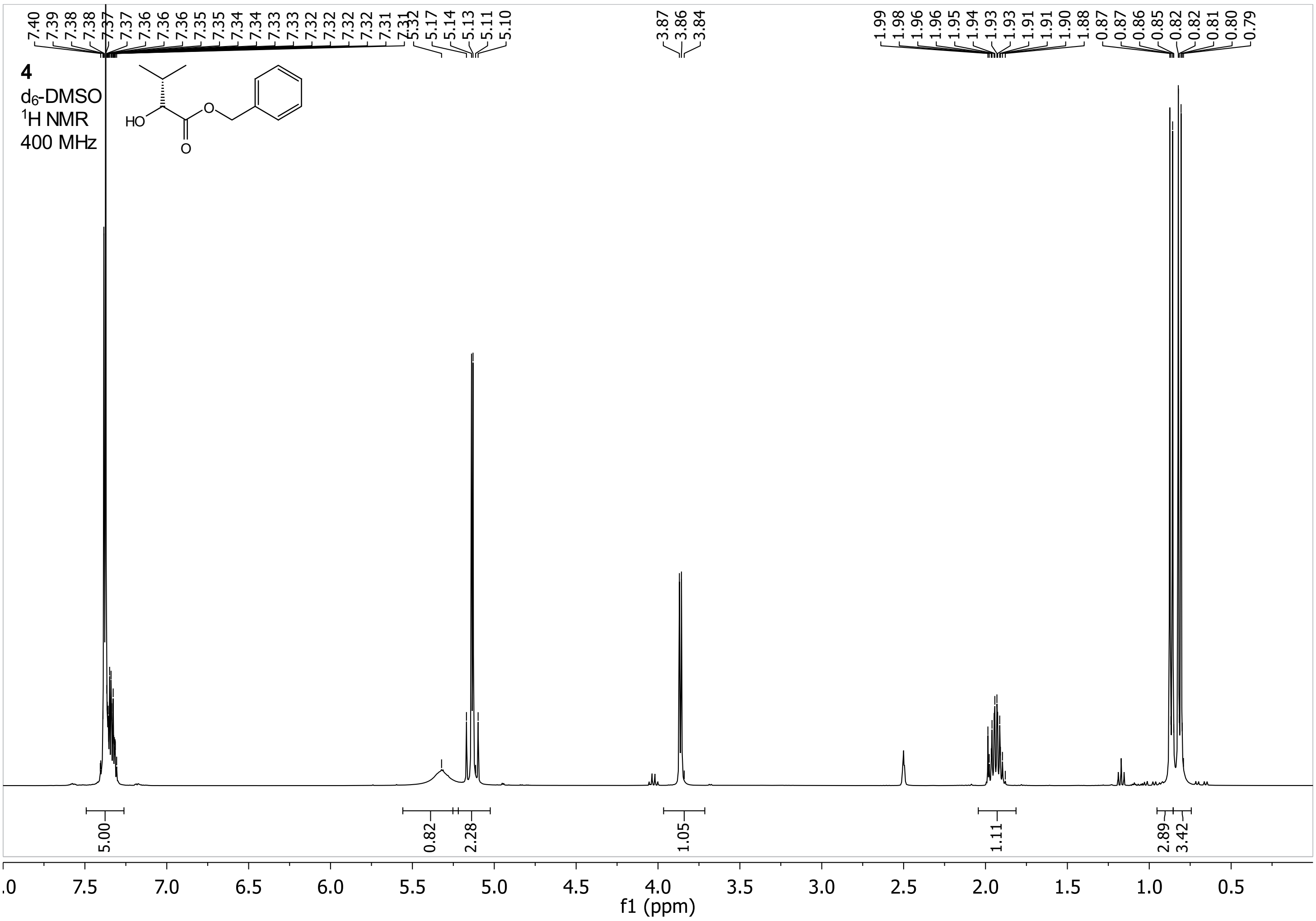
4
d6-DMSO
1H NMR
400 MHz
HO
O
O
7.40 7.39 7.38 7.38 7.37 7.37 7.37 7.36 7.36 7.36 7.35 7.35 7.34 7.34 7.33 7.33 7.32 7.32 7.32 7.32 7.32 7.31 7.31 5.32 5.17 5.14 5.13 5.11 5.10
3.87 3.86 3.84
1.99 1.98 1.96 1.96 1.95 1.94 1.93 1.93 1.93 1.91 1.91 1.90 1.88 0.87 0.87 0.86 0.85 0.82 0.82 0.81 0.80 0.79
5.00
0.82
2.28
1.05
1.11
2.89
3.42
f1 (ppm)

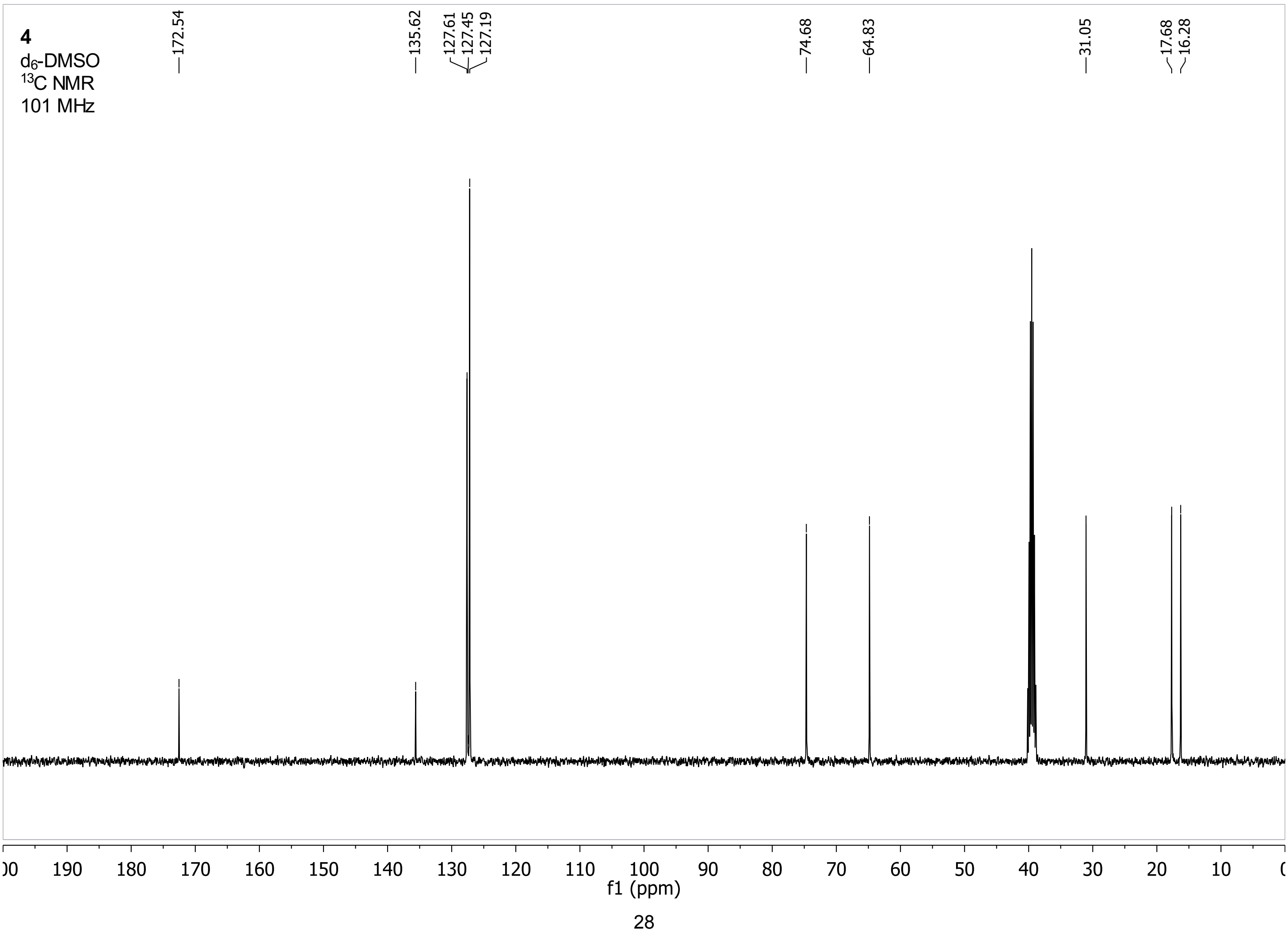
4
d6-DMSO
13C NMR
101 MHz
172.54
135.62 127.61 127.45 127.19
74.68
64.83
31.05
17.68 16.28
f1 (ppm)

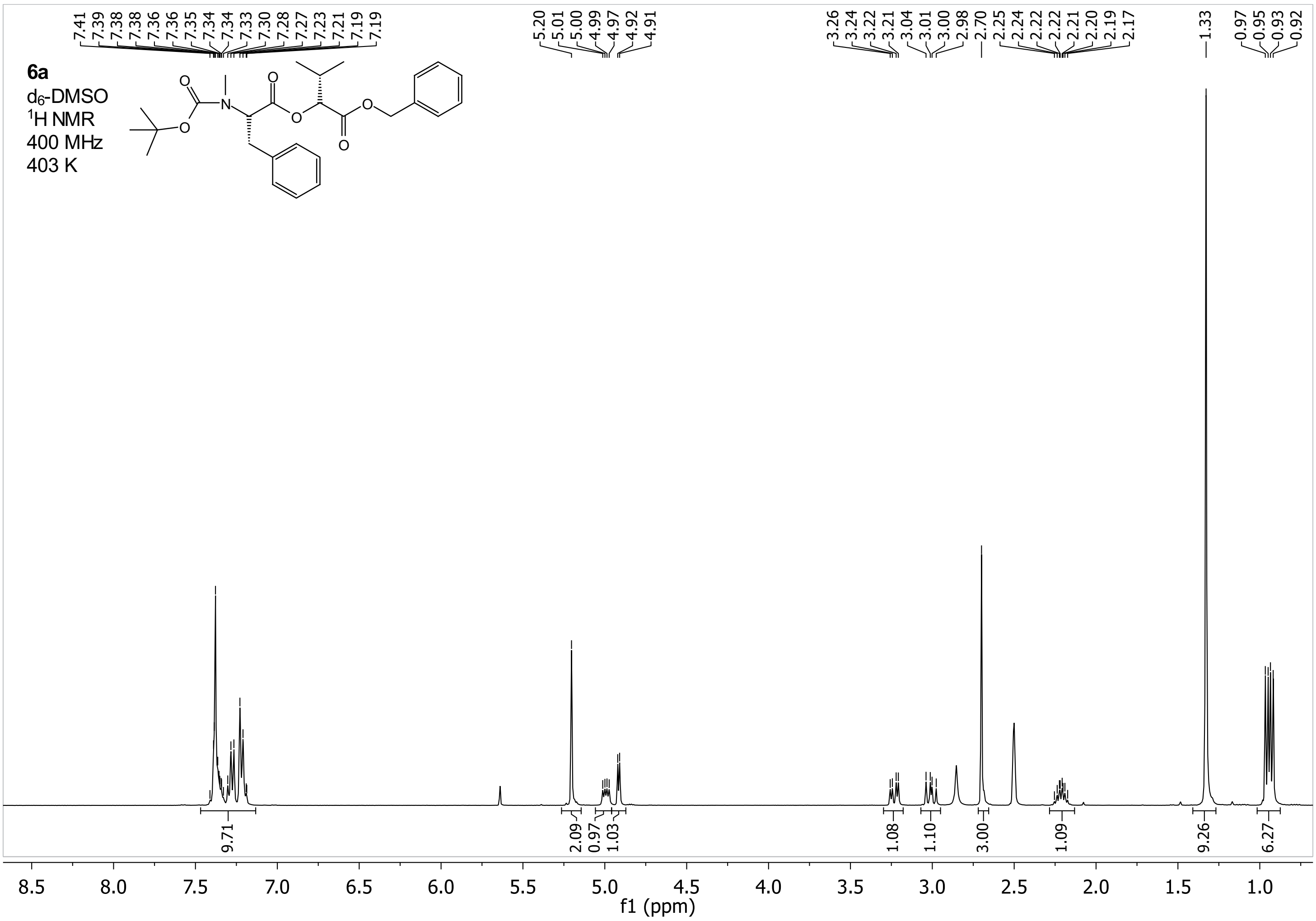
6a
d6-DMSO
1H NMR
400 MHz
403 K
7.41 7.39 7.38 7.38 7.36 7.36 7.35 7.34 7.34 7.33 7.30 7.28 7.27 7.23 7.21 7.19 7.19
5.20 5.01 5.00 4.99 4.97 4.92 4.91
3.26 3.24 3.22 3.21 3.04 3.01 3.00 2.98 2.70 2.25 2.24 2.22 2.22 2.21 2.20 2.19 2.17
1.33
0.97 0.95 0.93 0.92
9.71
2.09 0.97 1.03
1.08 1.10 3.00 1.09
9.26 6.27
8.5 8.0 7.5 7.0 6.5 6.0 5.5 5.0 4.5 4.0 3.5 3.0 2.5 2.0 1.5 1.0
f1 (ppm)

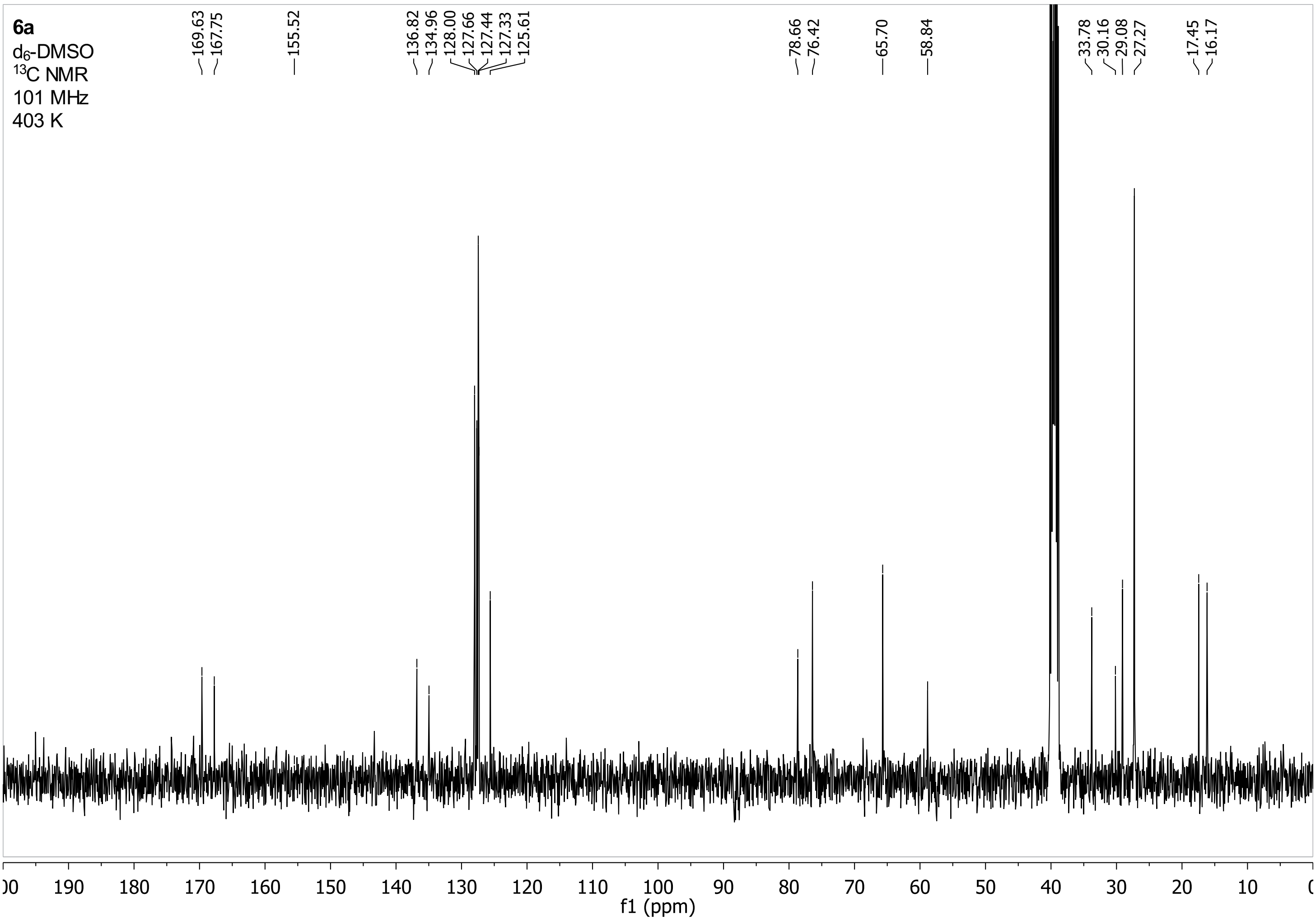
6a
d6-DMSO
13C NMR
101 MHz
403 K
169.63 167.75
155.52
136.82 134.96 128.00 127.66 127.44 127.33 125.61
78.66 76.42
65.70
58.84
33.78 30.16 29.08 27.27
17.45 16.17
190 180 170 160 150 140 130 120 110 100 90 80 70 60 50 40 30 20 10
f1 (ppm)

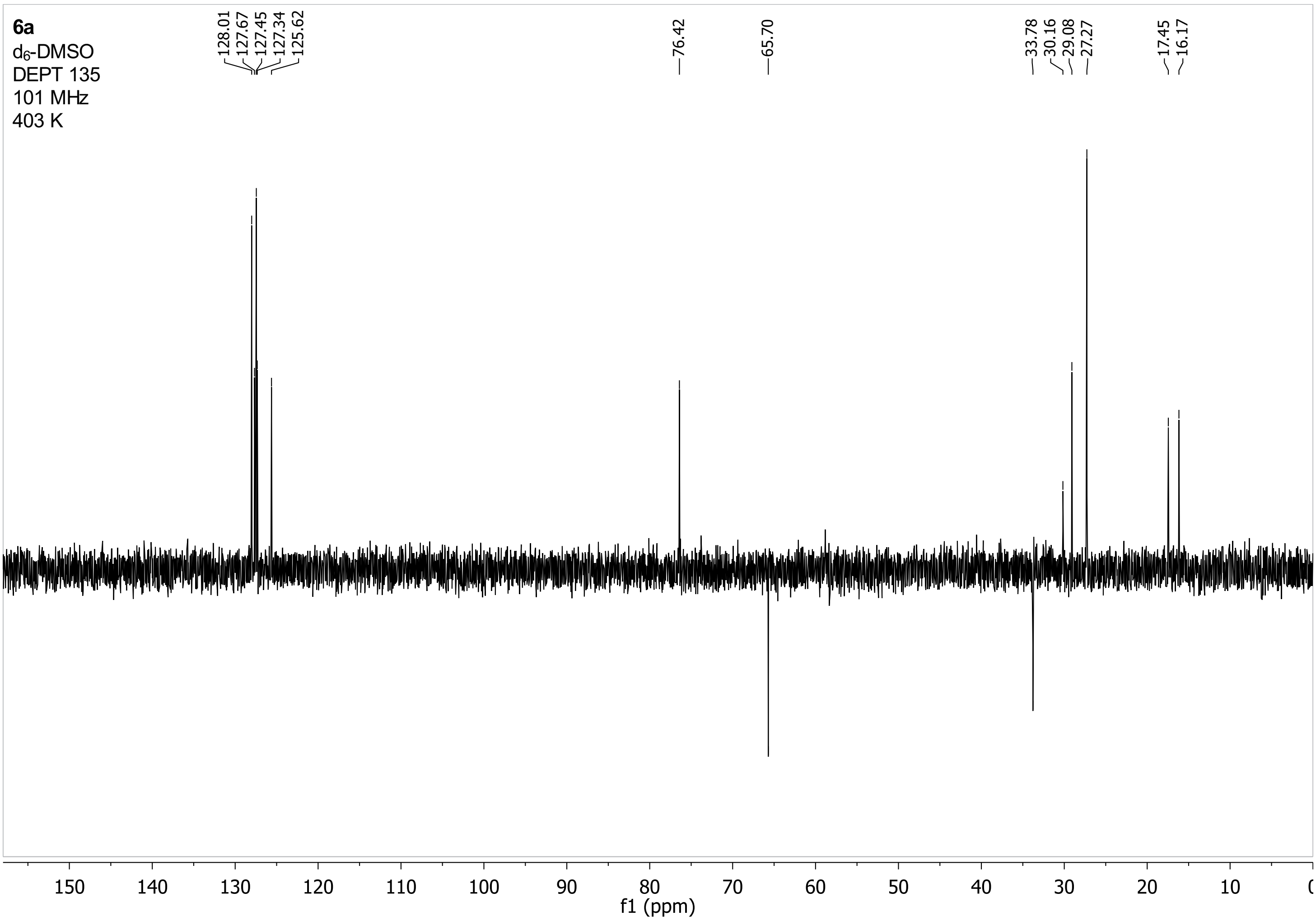
6a
d6-DMSO
DEPT 135
101 MHz
403 K
128.01
127.67
127.45
127.34
125.62
76.42
65.70
33.78
30.16
29.08
27.27
17.45
16.17
f1 (ppm)

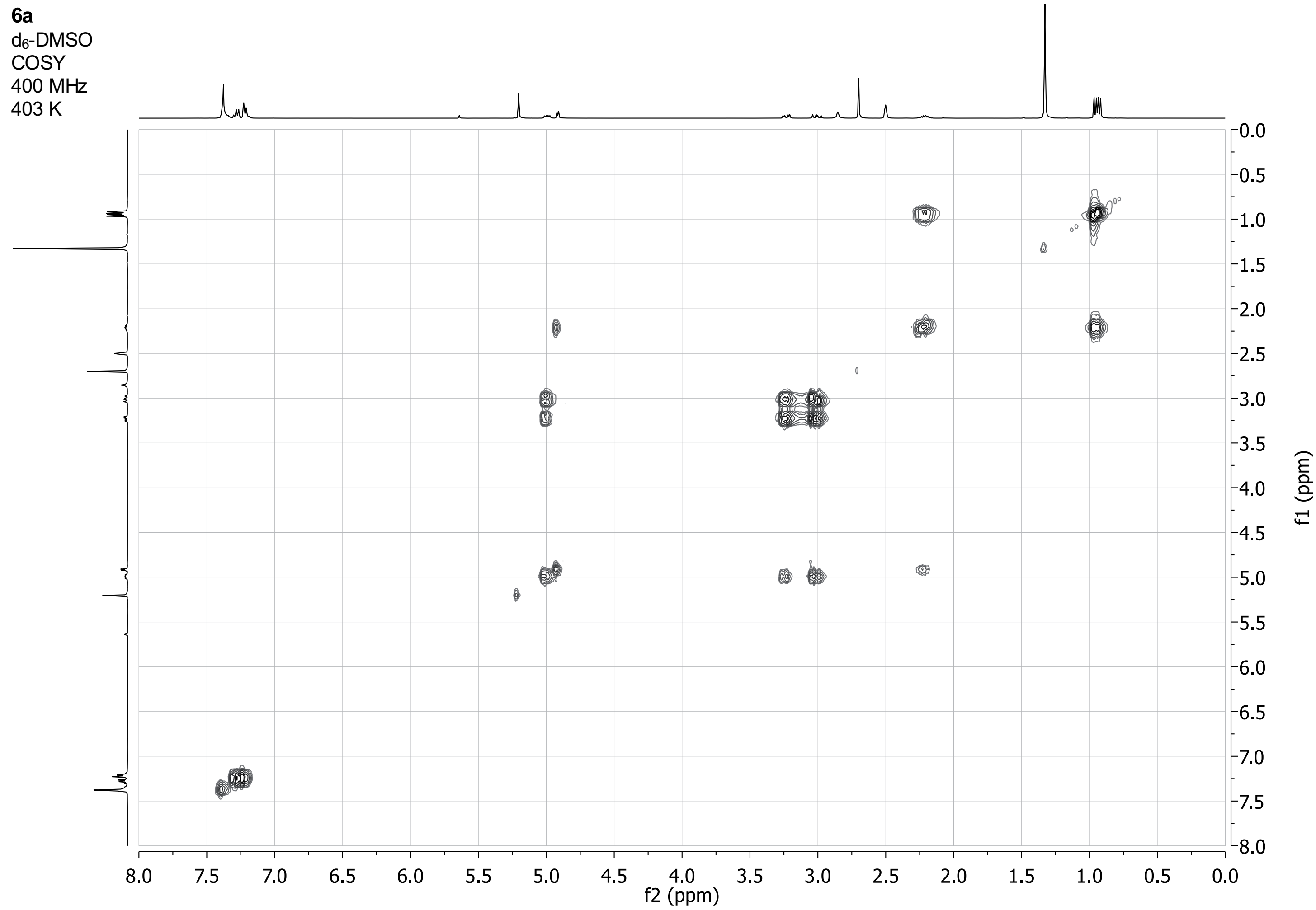
6a
d6-DMSO
COSY
400 MHz
403 K
f2 (ppm)
f1 (ppm)

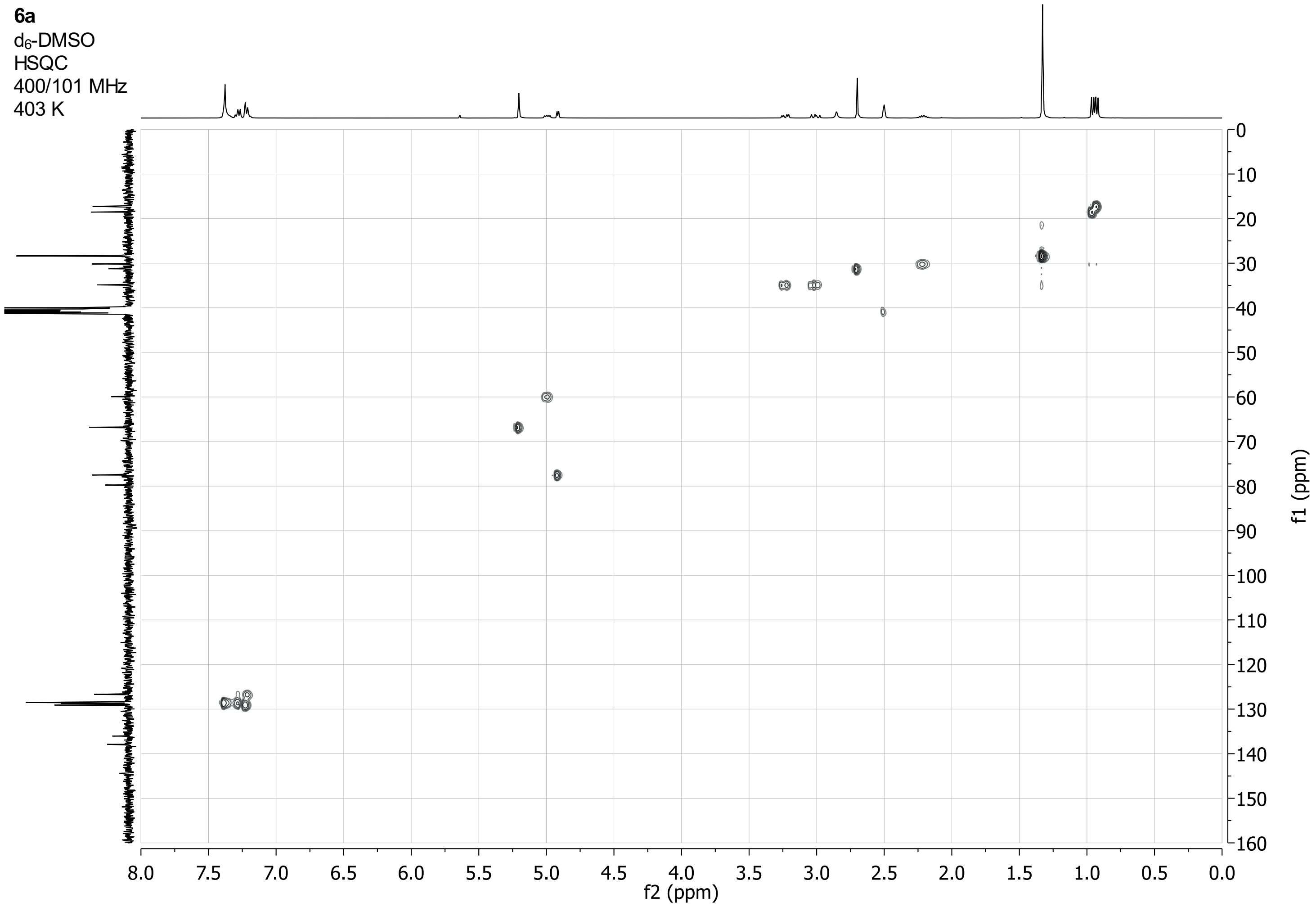
6a
d6-DMSO
HSQC
400/101 MHz
403 K
f1 (ppm)
f2 (ppm)

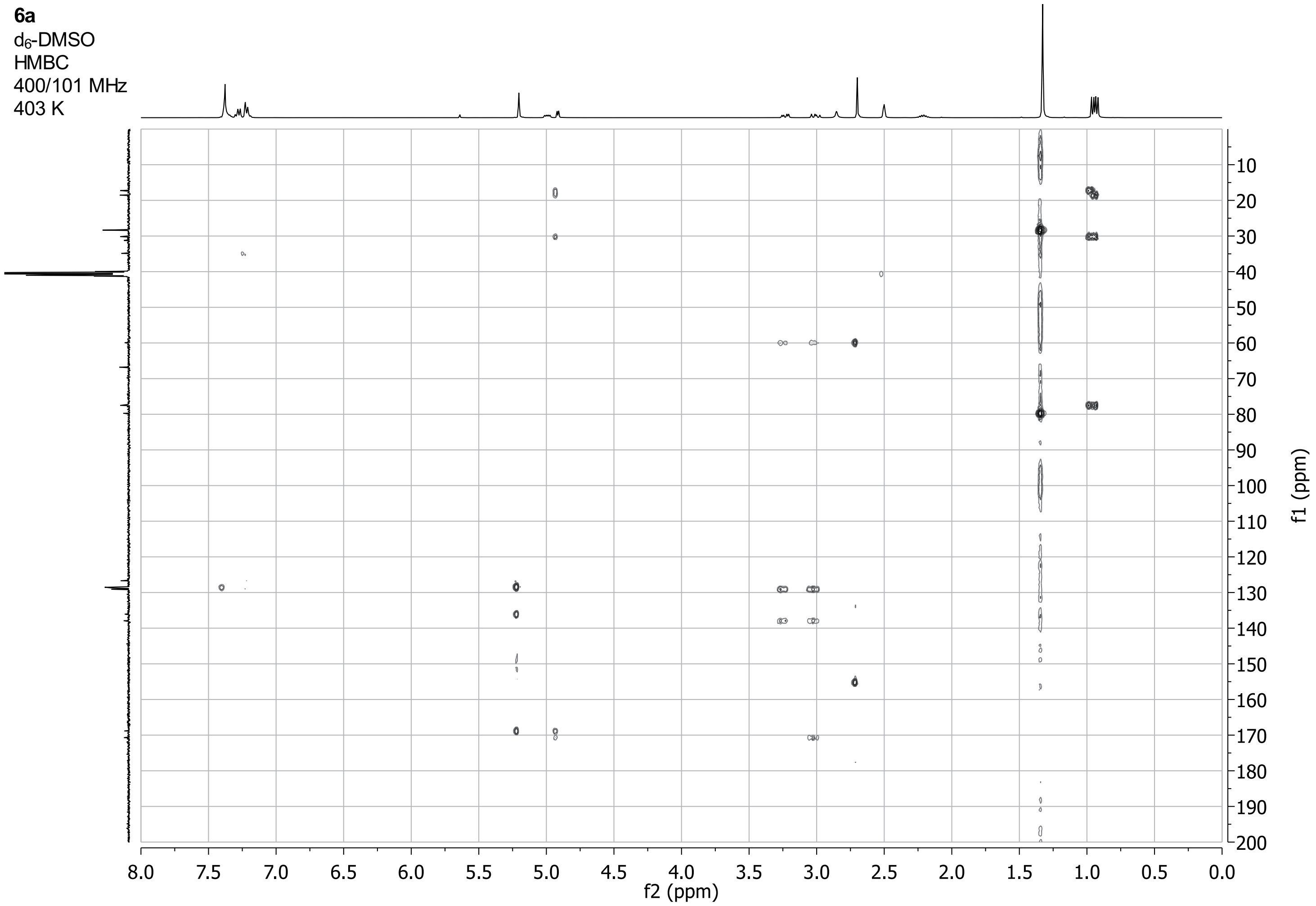
6a
d6-DMSO
HMBC
400/101 MHz
403 K
f1 (ppm)
f2 (ppm)

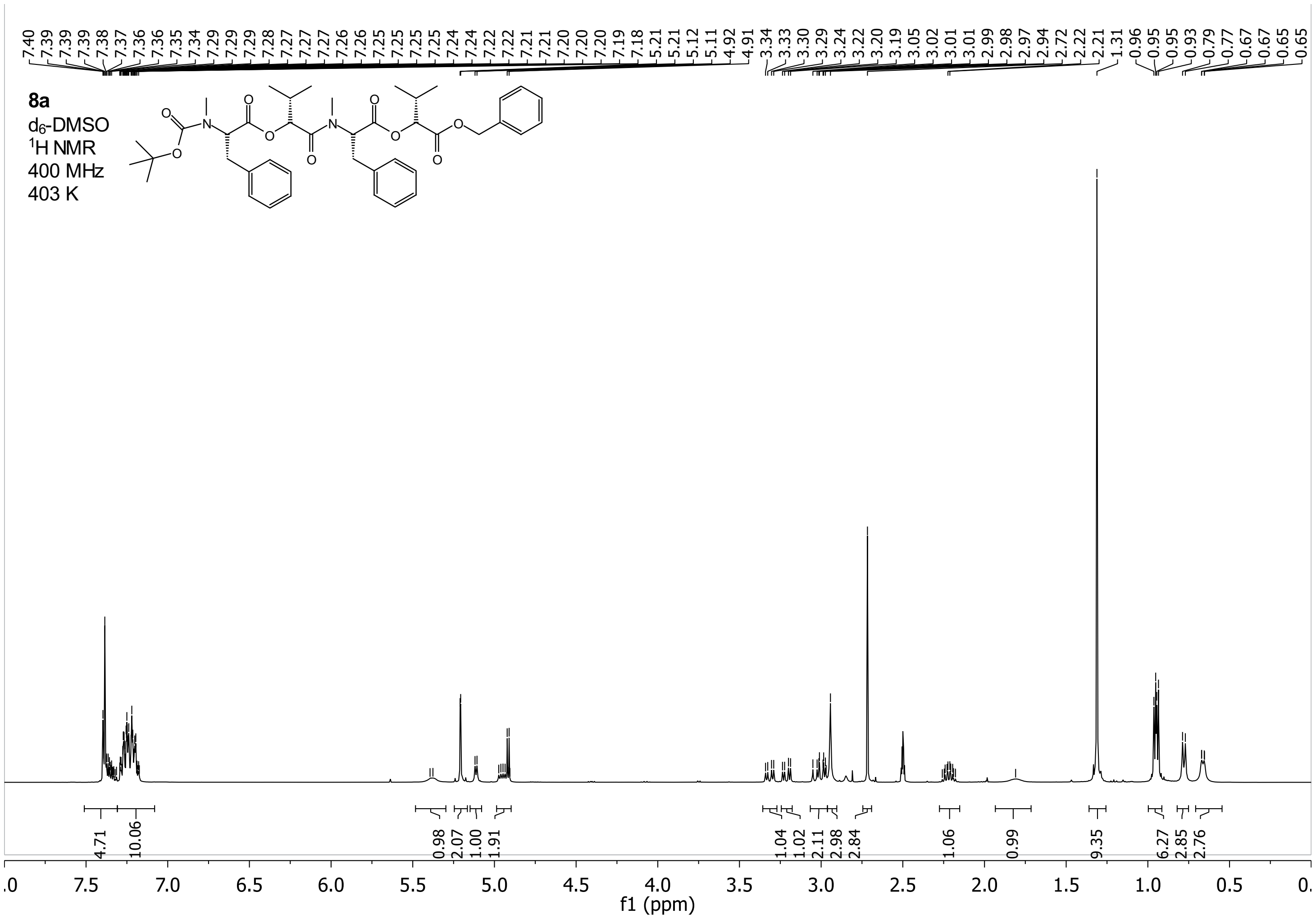
8a
d6-DMSO
1H NMR
400 MHz
403 K
7.40 7.39 7.39 7.39 7.38 7.37 7.36 7.36 7.35 7.34 7.29 7.29 7.29 7.28 7.27 7.27 7.27 7.26 7.26 7.25 7.25 7.25 7.25 7.24 7.24 7.22 7.22 7.21 7.21 7.21 7.20 7.20 7.20 7.19 7.18 5.21 5.21 5.12 5.11 4.92 4.91 3.34 3.33 3.30 3.29 3.24 3.22 3.20 3.19 3.05 3.02 3.01 3.01 2.99 2.98 2.97 2.94 2.72 2.22 2.21 1.31 0.96 0.95 0.95 0.93 0.79 0.77 0.67 0.65 0.65
4.71 10.06 0.98 2.07 1.00 1.91 1.04 1.02 2.11 2.98 2.84 1.06 0.99 9.35 6.27 2.85 2.76
f1 (ppm)

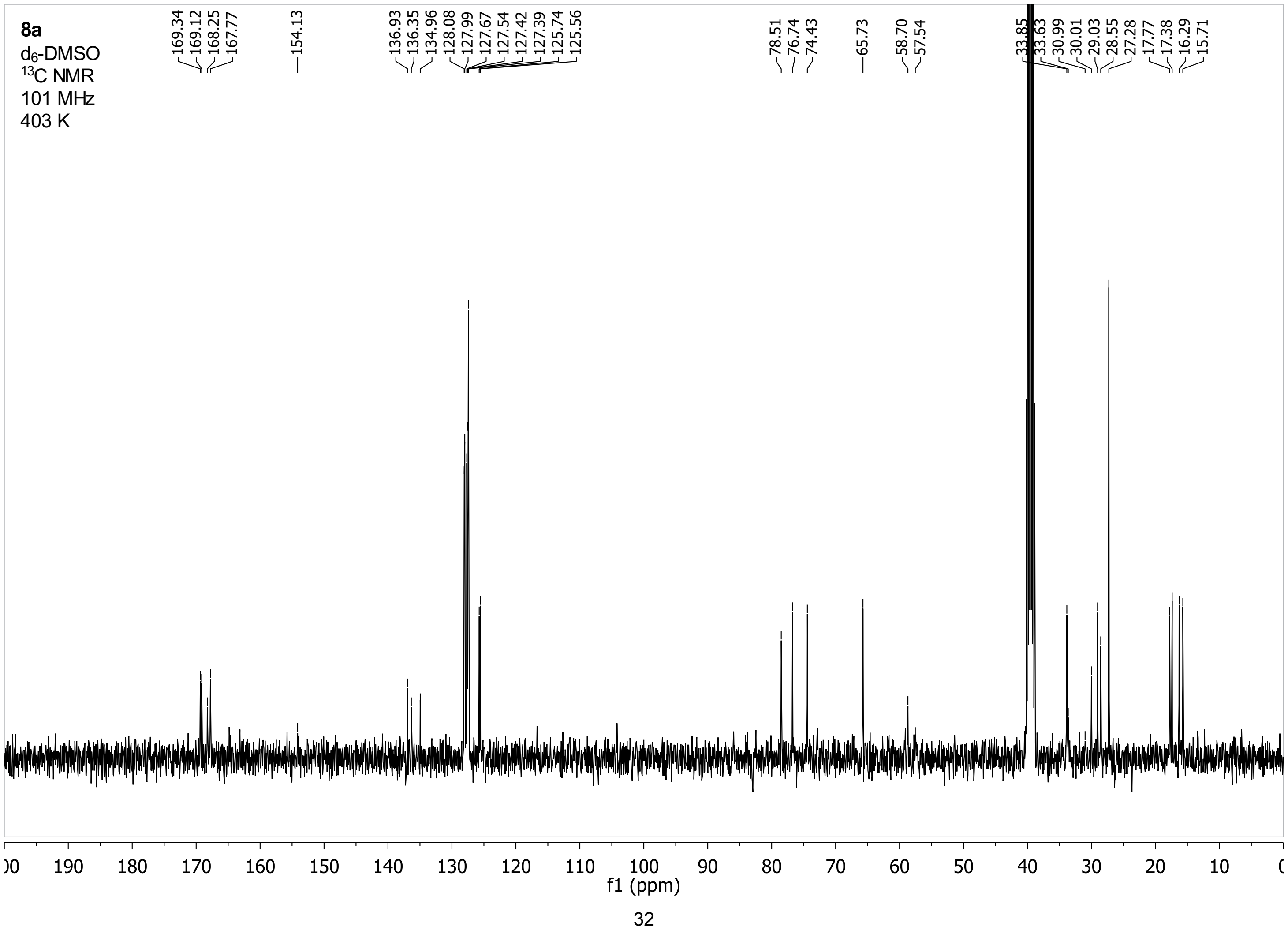
8a
d6-DMSO
13C NMR
101 MHz
403 K
169.34 169.12 168.25 167.77 154.13 136.93 136.35 134.96 128.08 127.99 127.67 127.54 127.42 127.39 125.74 125.56 78.51 76.74 74.43 65.73 58.70 57.54 33.85 33.63 30.99 30.01 29.03 28.55 27.28 17.77 17.38 16.29 15.71
f1 (ppm)

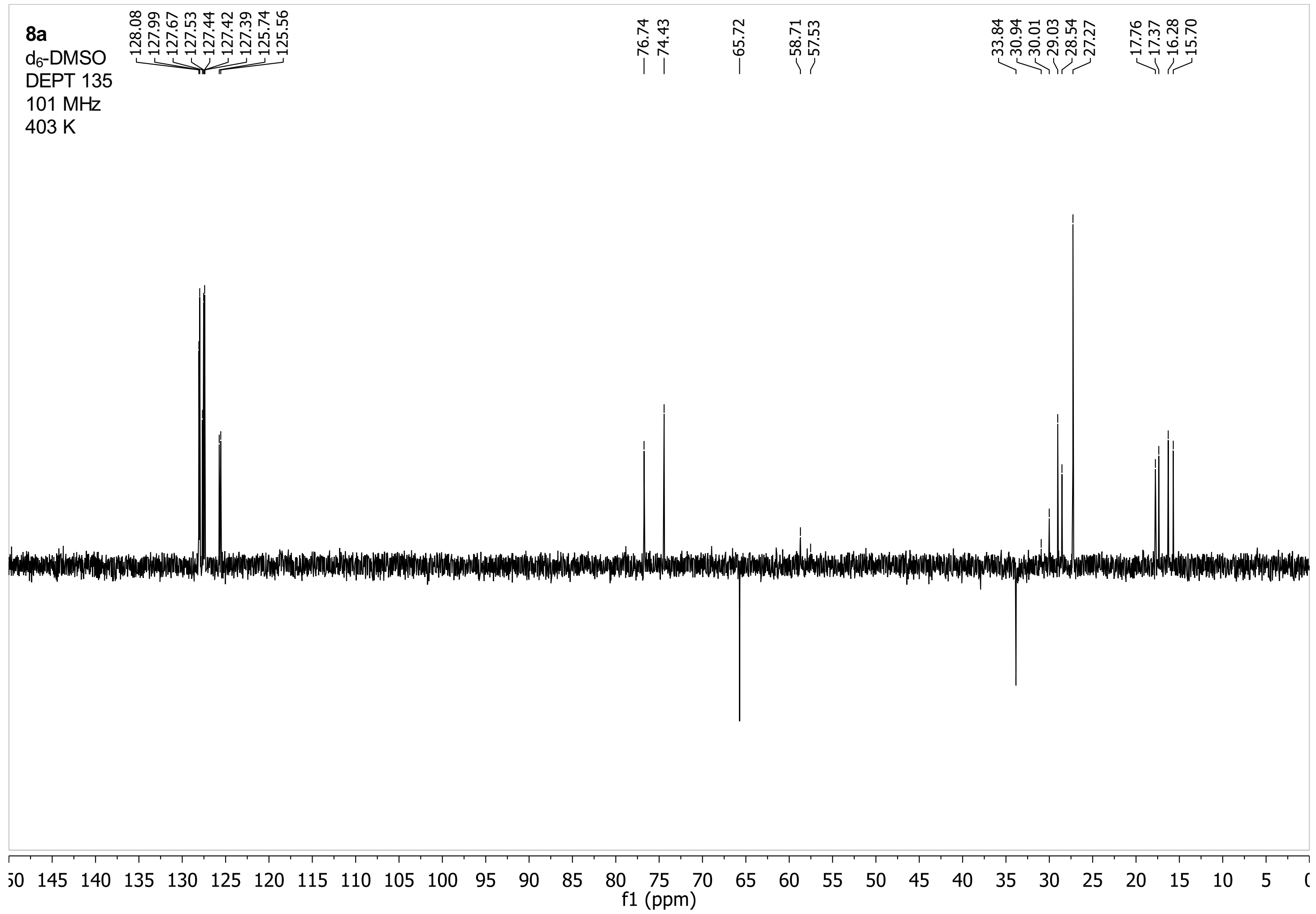
8a
d6-DMSO
DEPT 135
101 MHz
403 K
128.08
127.99
127.67
127.53
127.44
127.42
127.39
125.74
125.56
76.74
74.43
65.72
58.71
57.53
33.84
30.94
30.01
29.03
28.54
27.27
17.76
17.37
16.28
15.70
f1 (ppm)

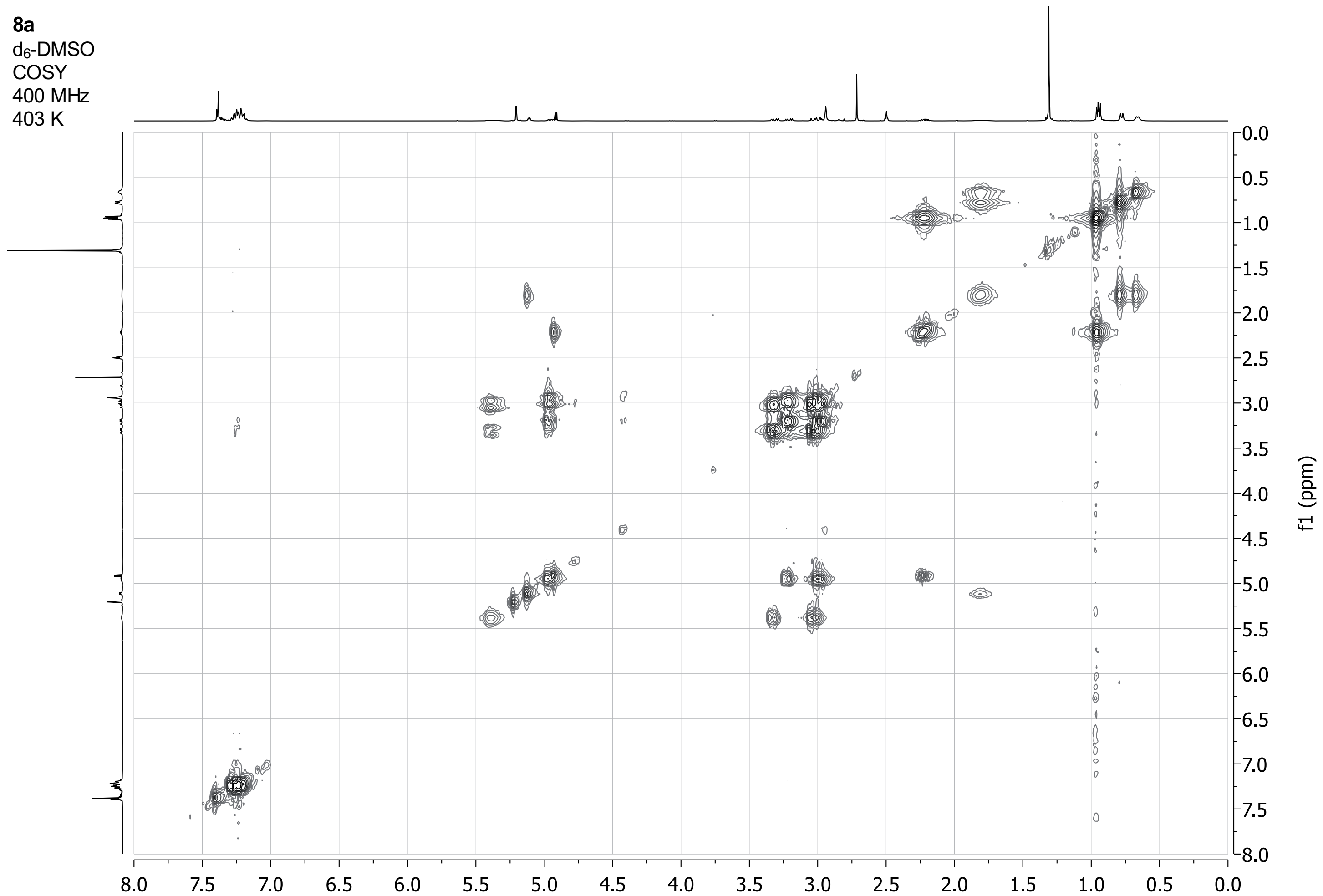
8a
d6-DMSO
COSY
400 MHz
403 K
f2 (ppm)
f1 (ppm)

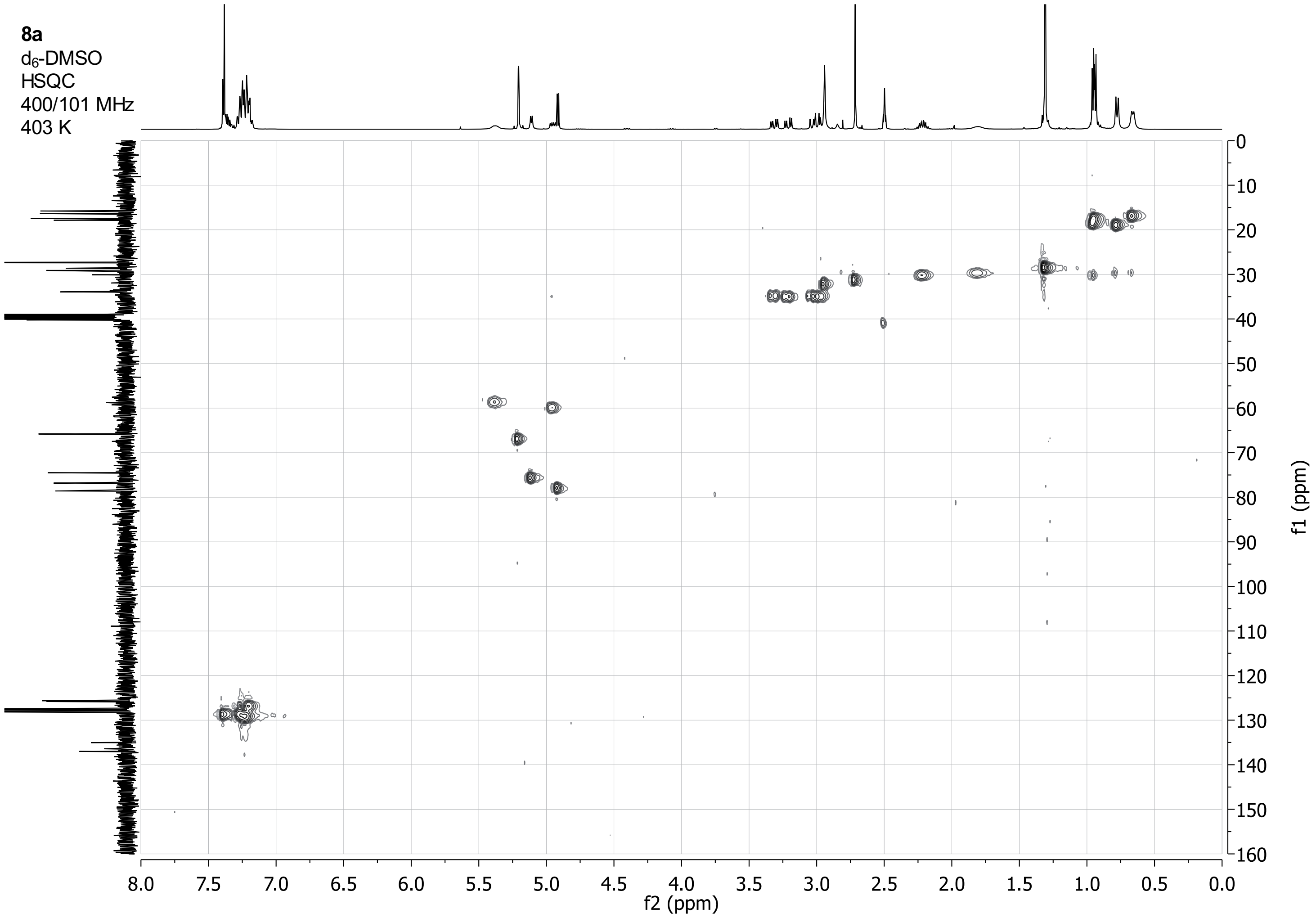
8a
d6-DMSO
HSQC
400/101 MHz
403 K
f1 (ppm)
f2 (ppm)

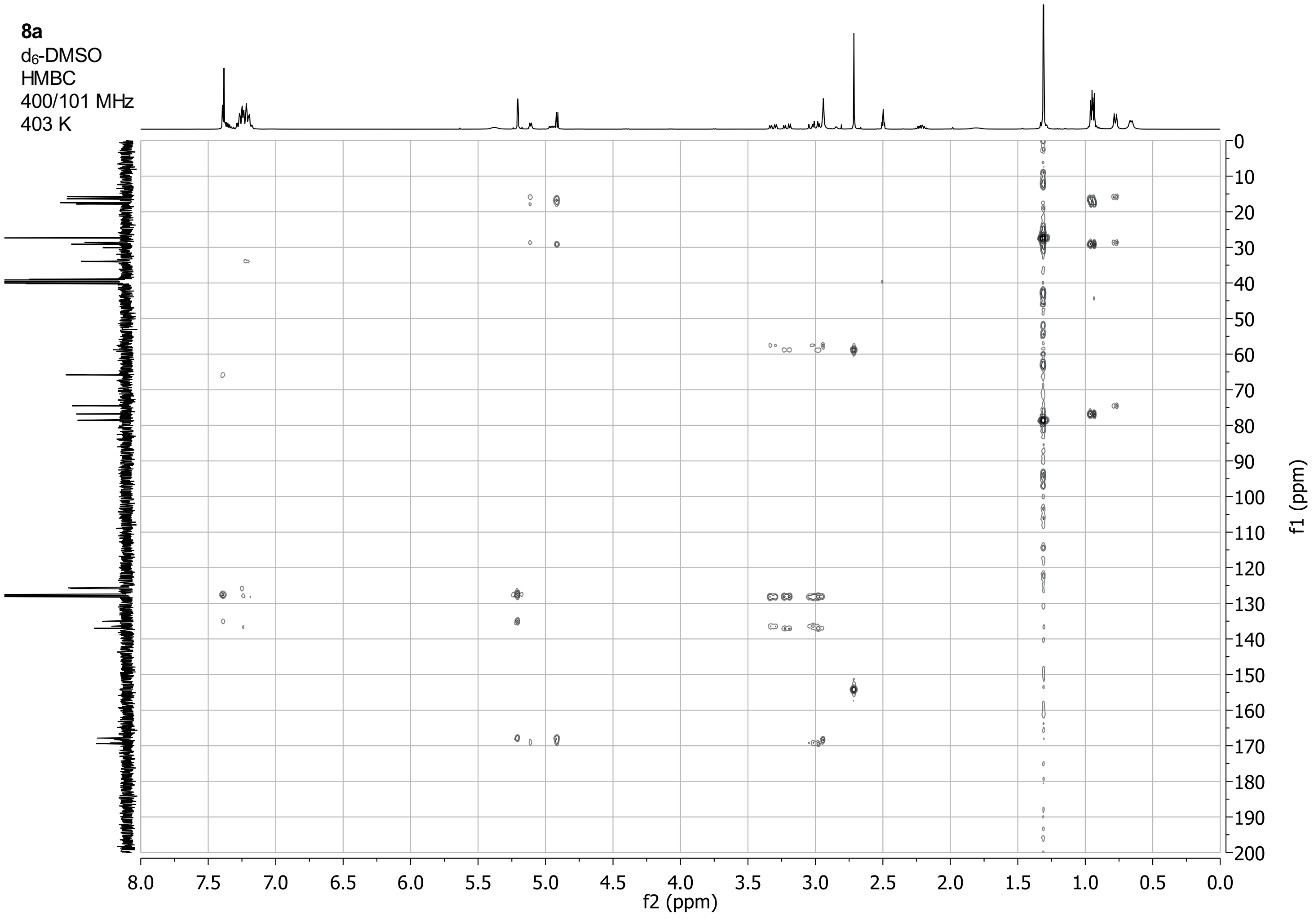
8a
d6-DMSO
HMBC
400/101 MHz
403 K
f1 (ppm)
f2 (ppm)

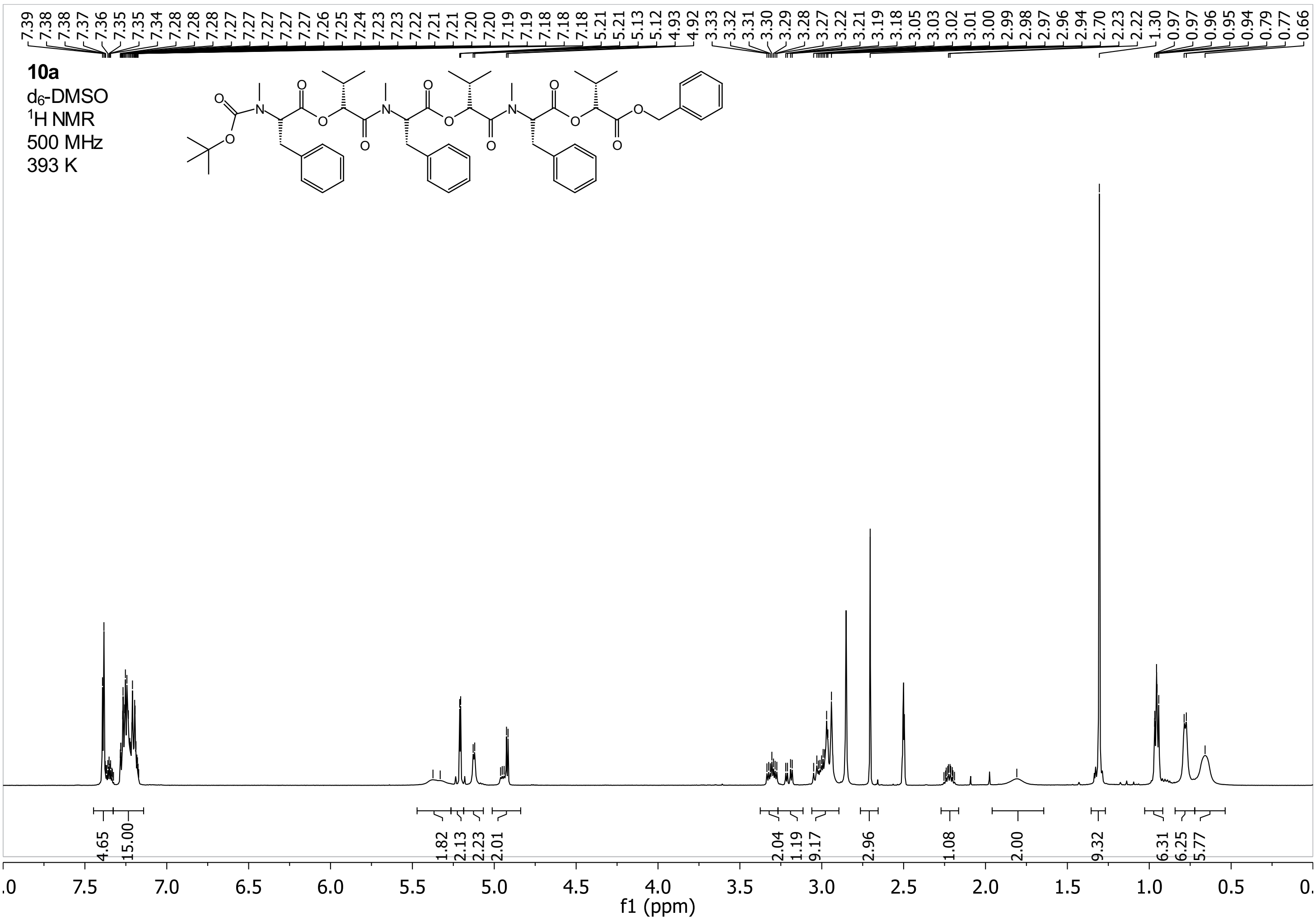
10a
d6-DMSO
1H NMR
500 MHz
393 K
7.39 7.38 7.38 7.37 7.36 7.35 7.35 7.34 7.28 7.28 7.28 7.27 7.27 7.27 7.27 7.27 7.26 7.25 7.24 7.23 7.23 7.22 7.21 7.21 7.20 7.20 7.19 7.19 7.18 7.18 7.18 5.21 5.21 5.13 5.12 4.93 4.92 3.33 3.32 3.31 3.30 3.29 3.28 3.27 3.22 3.21 3.19 3.18 3.05 3.03 3.02 3.01 3.00 2.99 2.98 2.97 2.96 2.94 2.70 2.23 2.22 1.30 0.97 0.97 0.96 0.95 0.94 0.79 0.77 0.66
4.65 15.00 1.82 2.13 2.23 2.01 2.04 1.19 9.17 2.96 1.08 2.00 9.32 6.31 6.25 5.77
f1 (ppm)

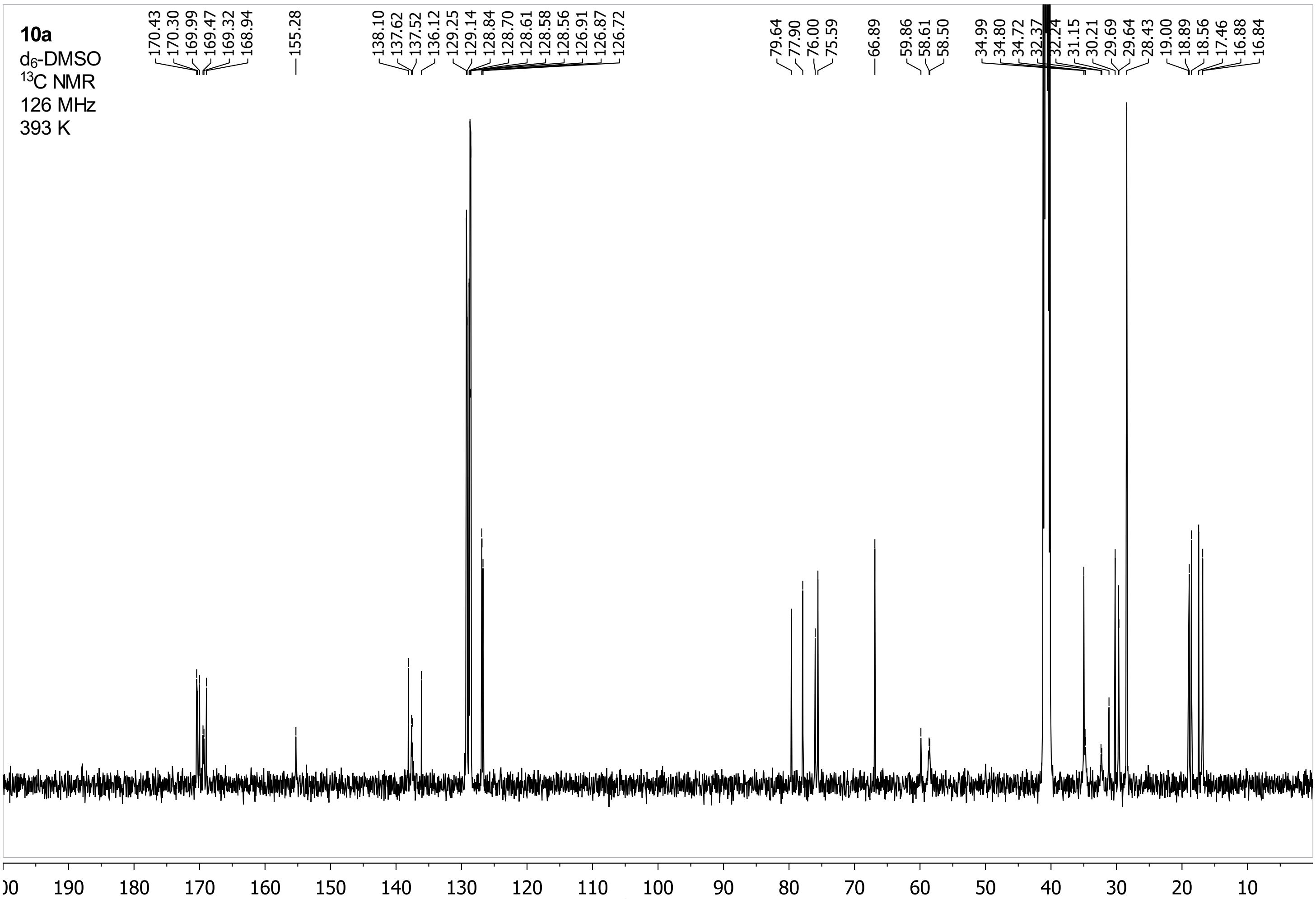
10a
d6-DMSO
13C NMR
126 MHz
393 K
170.43 170.30 169.99 169.47 169.32 168.94 155.28 138.10 137.62 137.52 136.12 129.25 129.14 128.84 128.70 128.61 128.58 128.56 126.91 126.87 126.72 79.64 77.90 76.00 75.59 66.89 59.86 58.61 58.50 34.99 34.80 34.72 32.37 32.24 31.15 30.21 29.69 29.64 28.43 19.00 18.89 18.56 17.46 16.88 16.84
f1 (ppm)

10a
d_6-DMSO
COSY
500 MHz
393 K

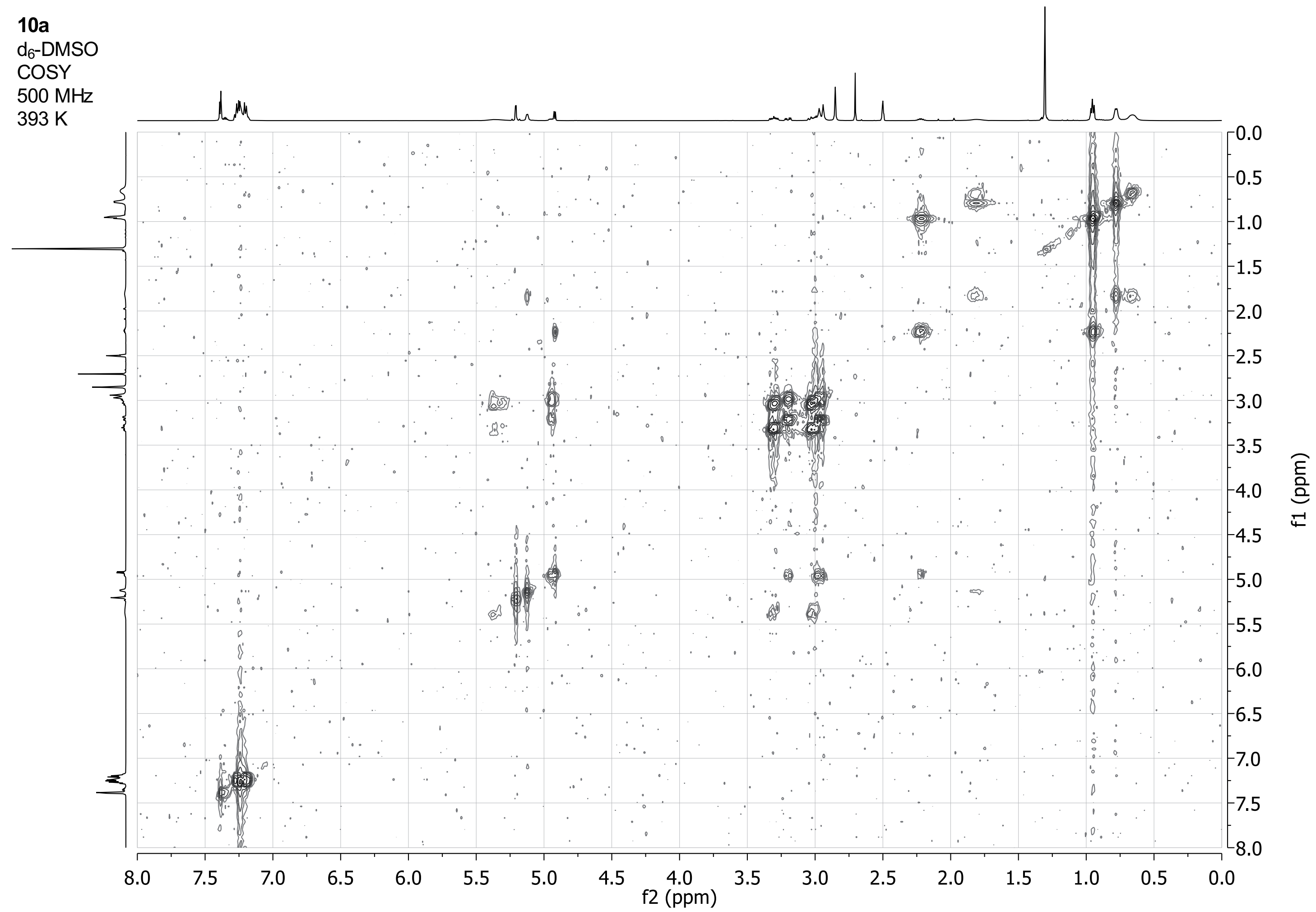

10a
d_6-DMSO
HSQC
500/126 MHz
393 K

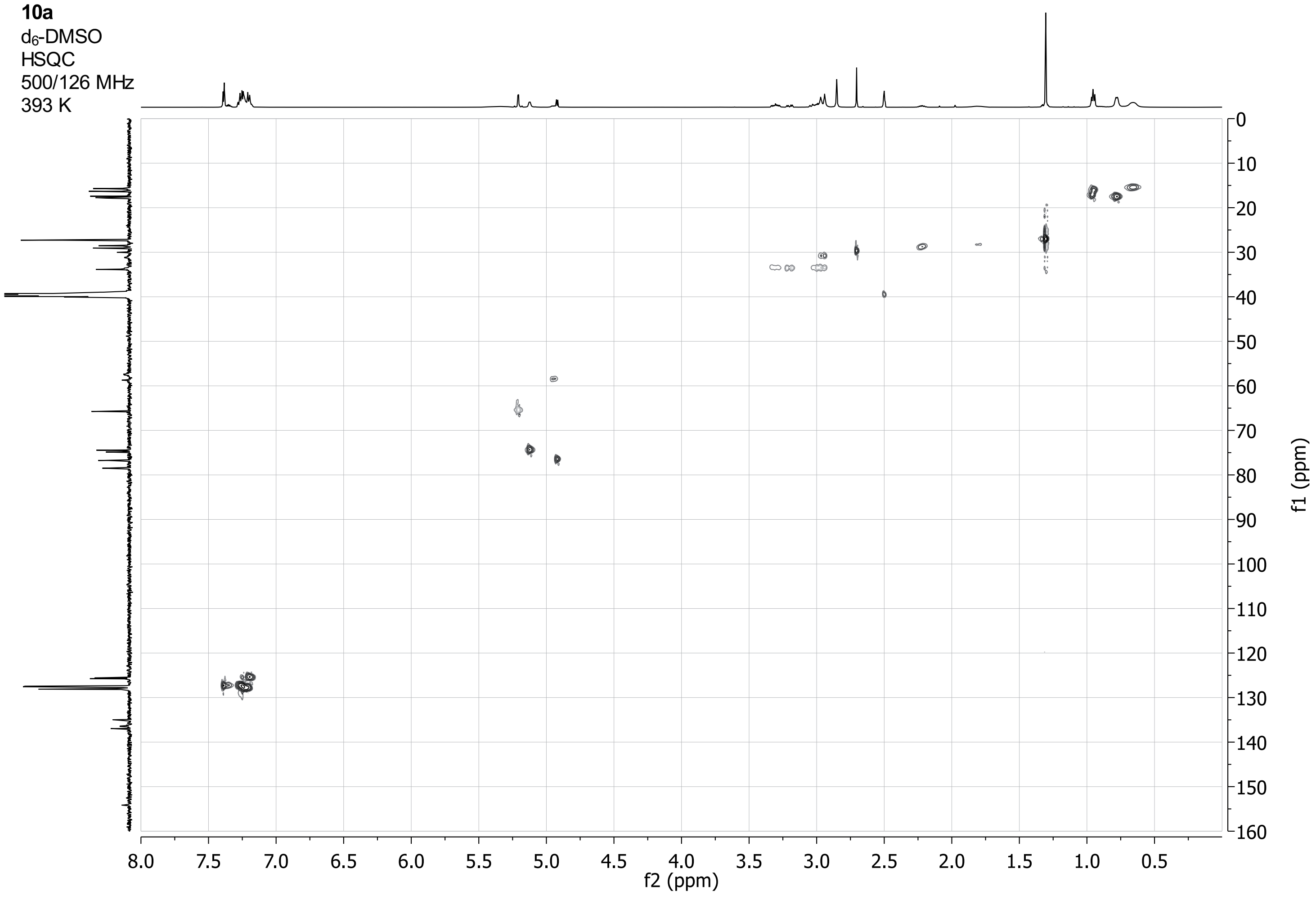

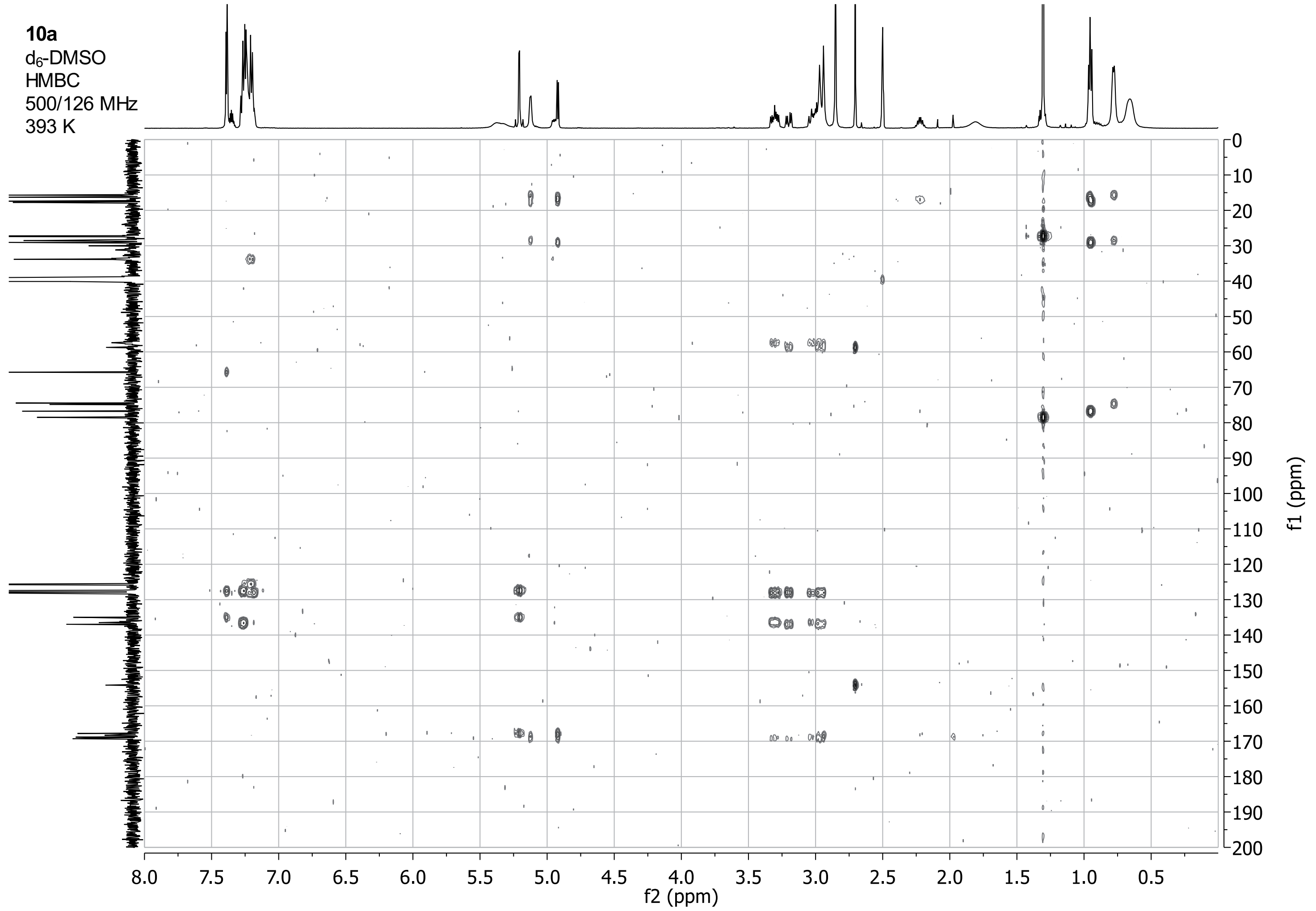
10a
d6-DMSO
HMBC
500/126 MHz
393 K
f1 (ppm)
f2 (ppm)

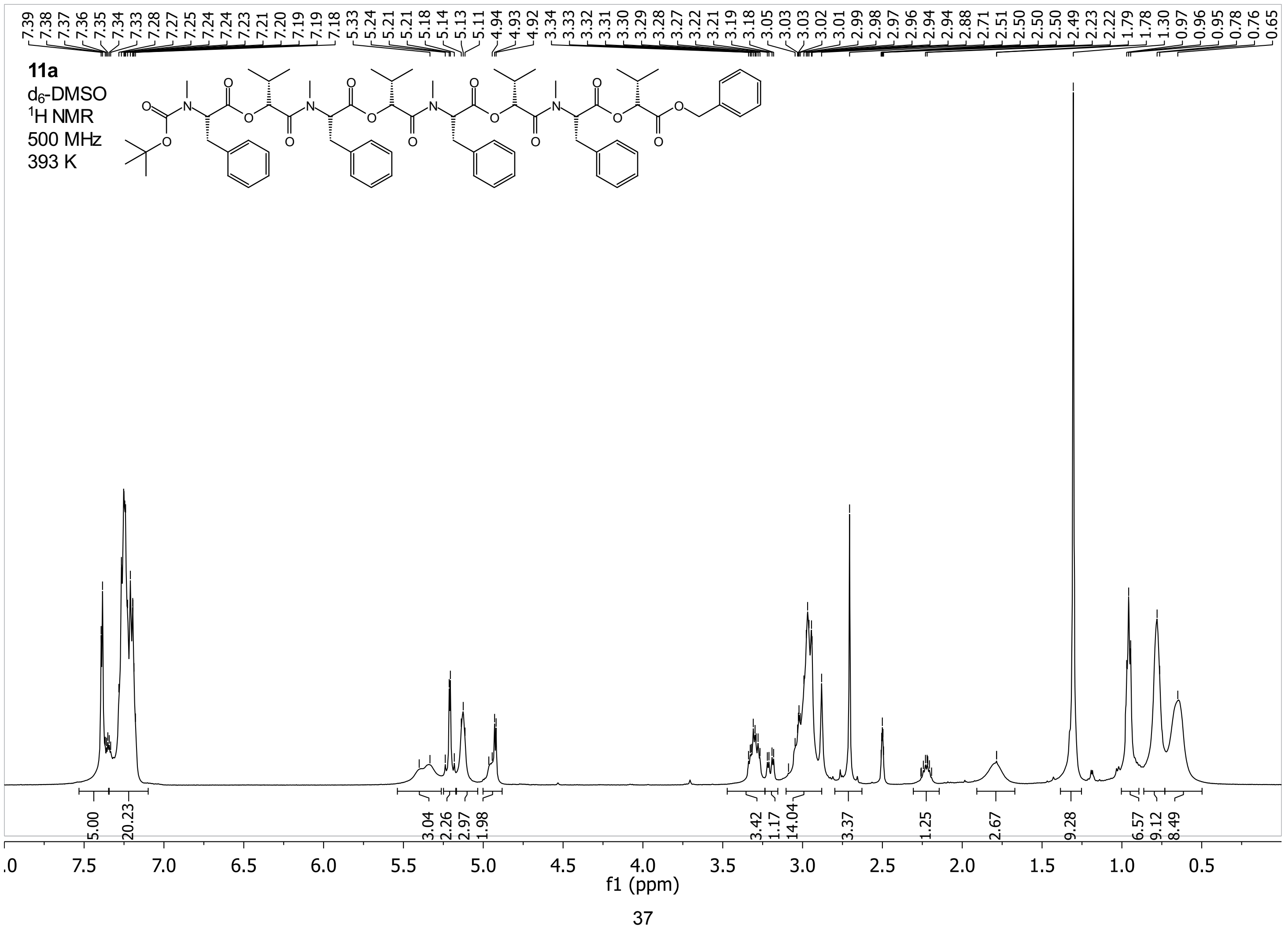
11a
d6-DMSO
1H NMR
500 MHz
393 K
f1 (ppm)

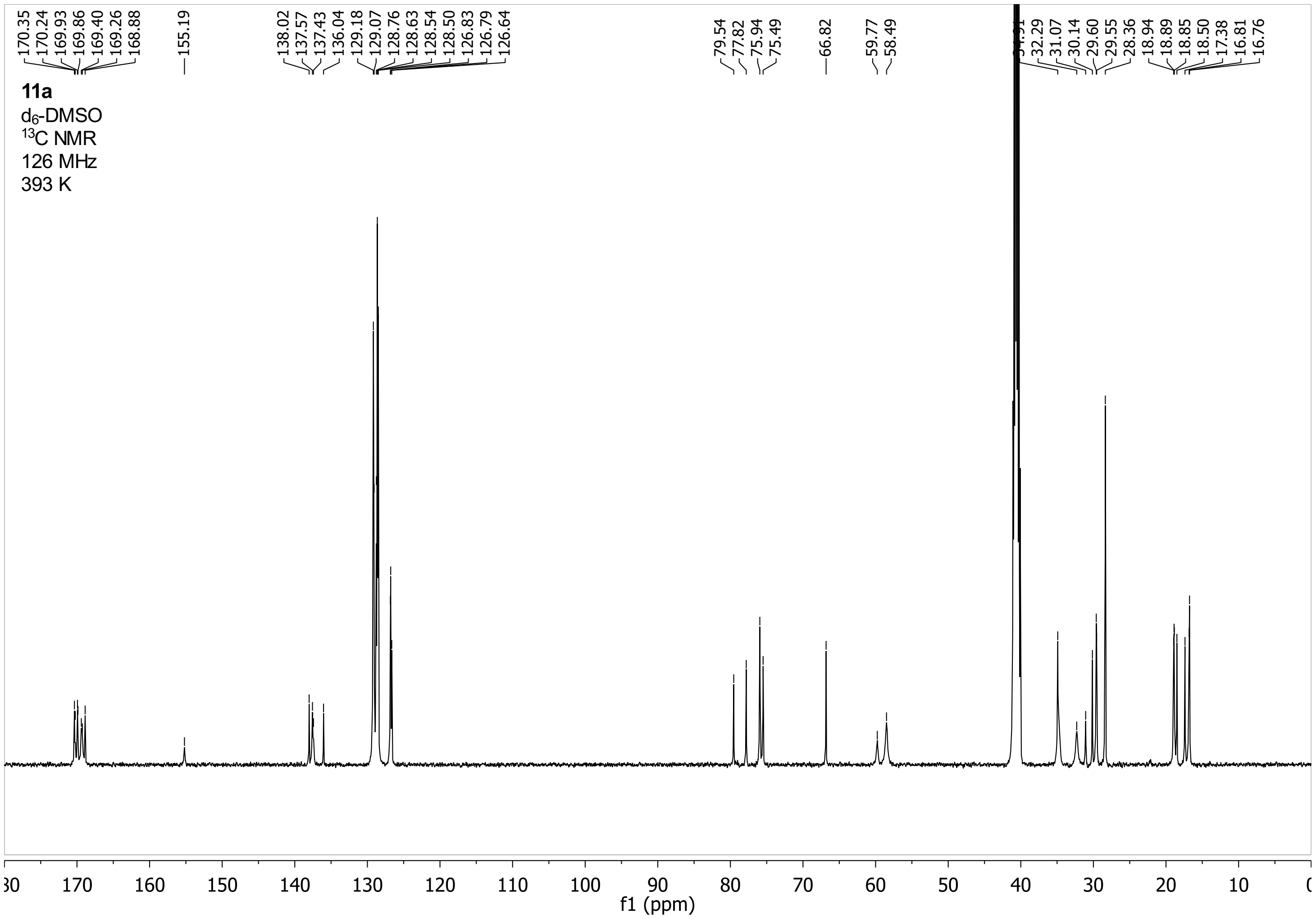
11a
d_6-DMSO
^{13}C NMR
126 MHz
393 K
170.35
170.24
169.93
169.86
169.40
169.26
168.88
155.19
138.02
137.57
137.43
136.04
129.18
129.07
128.76
128.63
128.54
128.50
126.83
126.79
126.64
79.54
77.82
75.94
75.49
66.82
59.77
58.49
32.29
31.07
30.14
29.60
29.55
28.36
18.94
18.89
18.85
18.50
17.38
16.81
16.76
f1 (ppm)

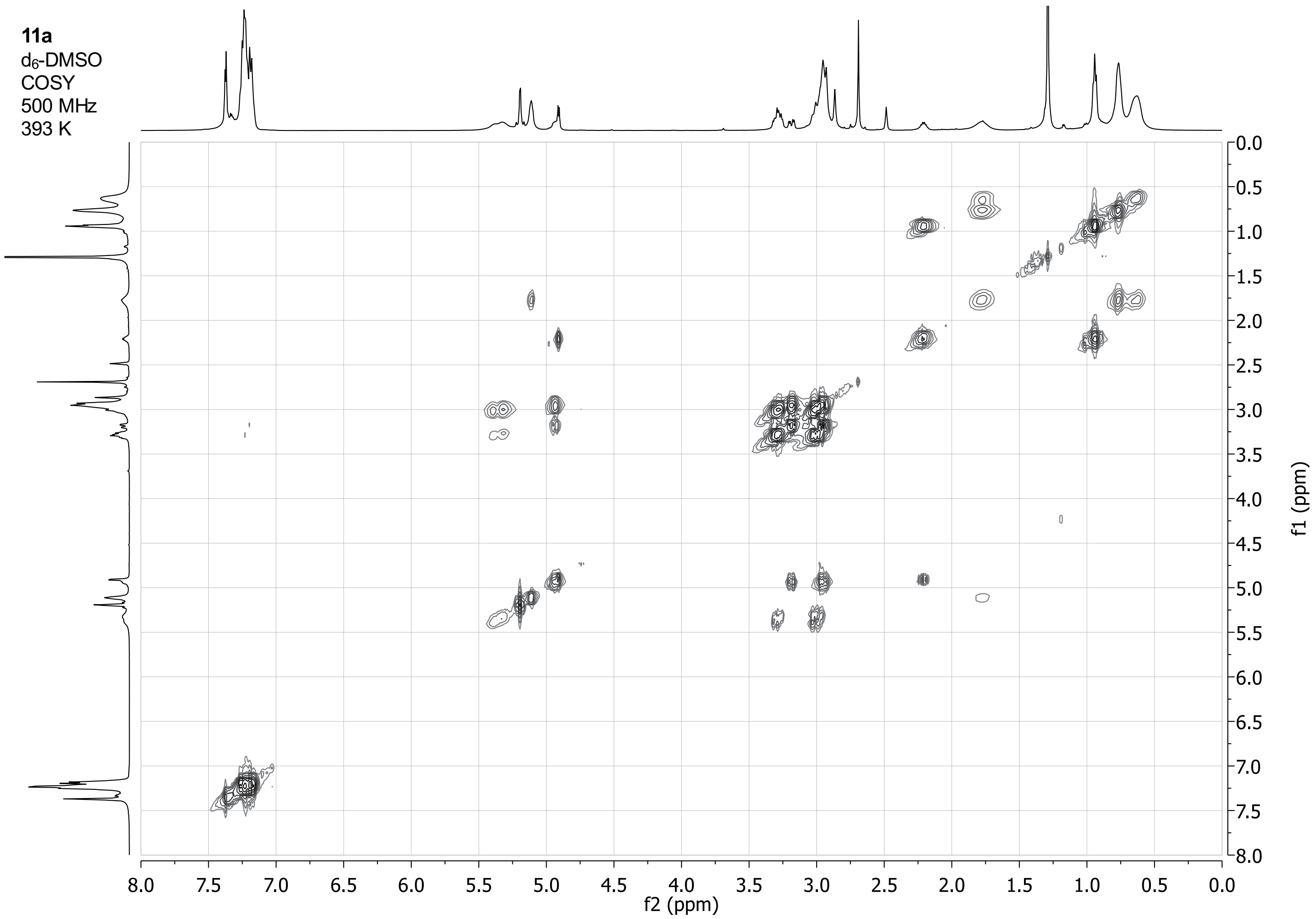
11a
d_6-DMSO
COSY
500 MHz
393 K
f2 (ppm)
f1 (ppm)

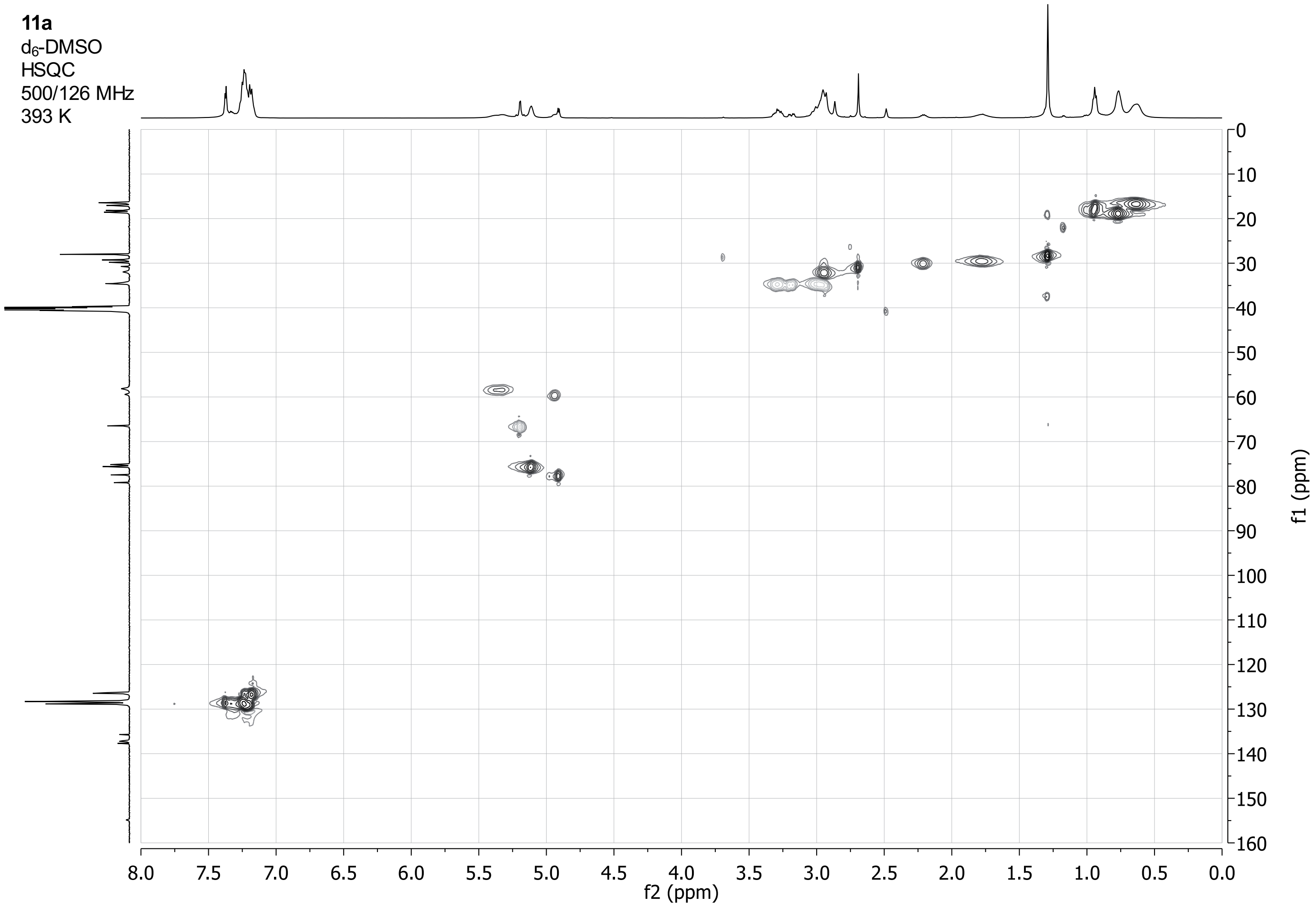
11a
d6-DMSO
HSQC
500/126 MHz
393 K
f1 (ppm)
f2 (ppm)

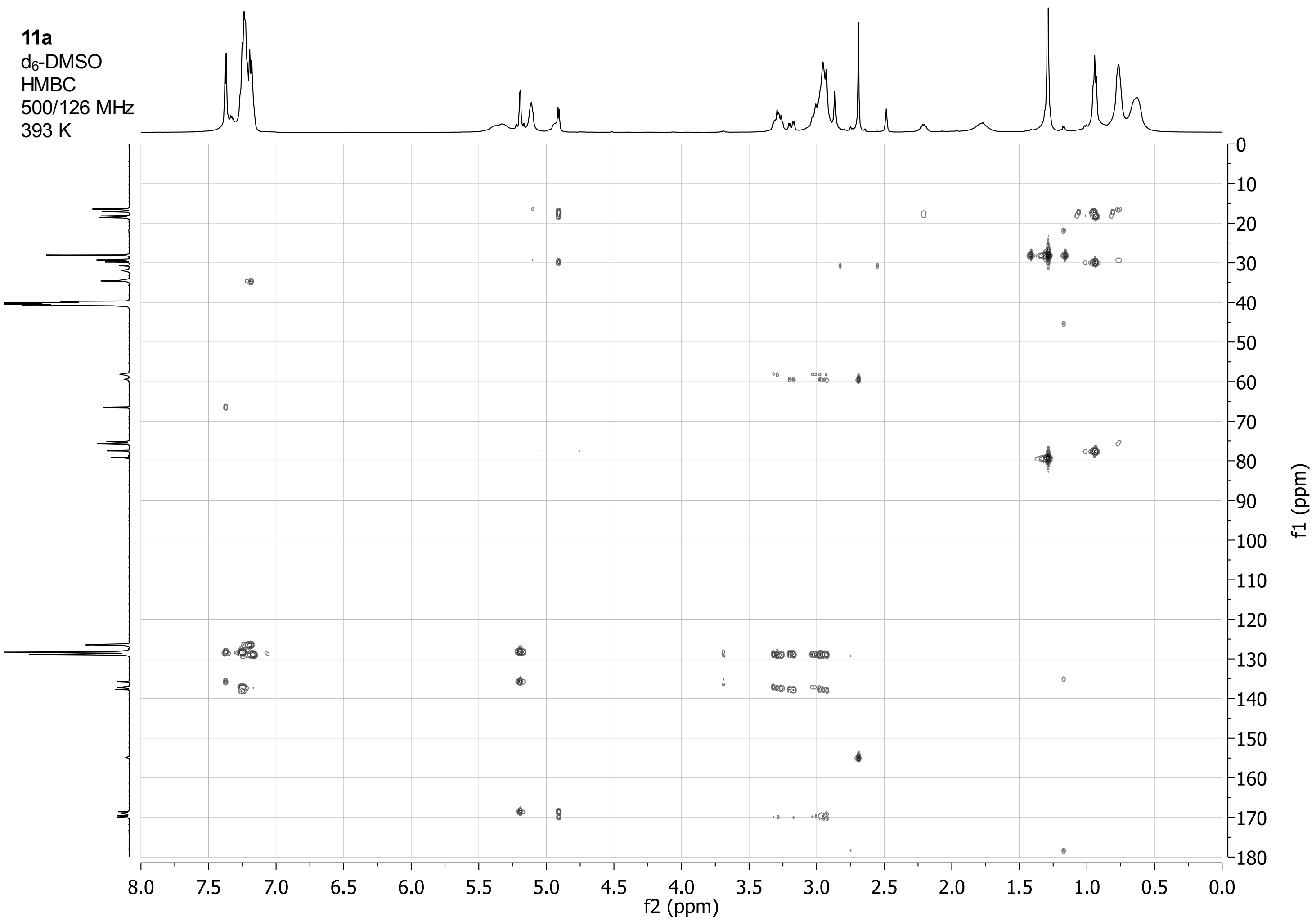
11a
d6-DMSO
HMBC
500/126 MHz
393 K
f1 (ppm)
f2 (ppm)

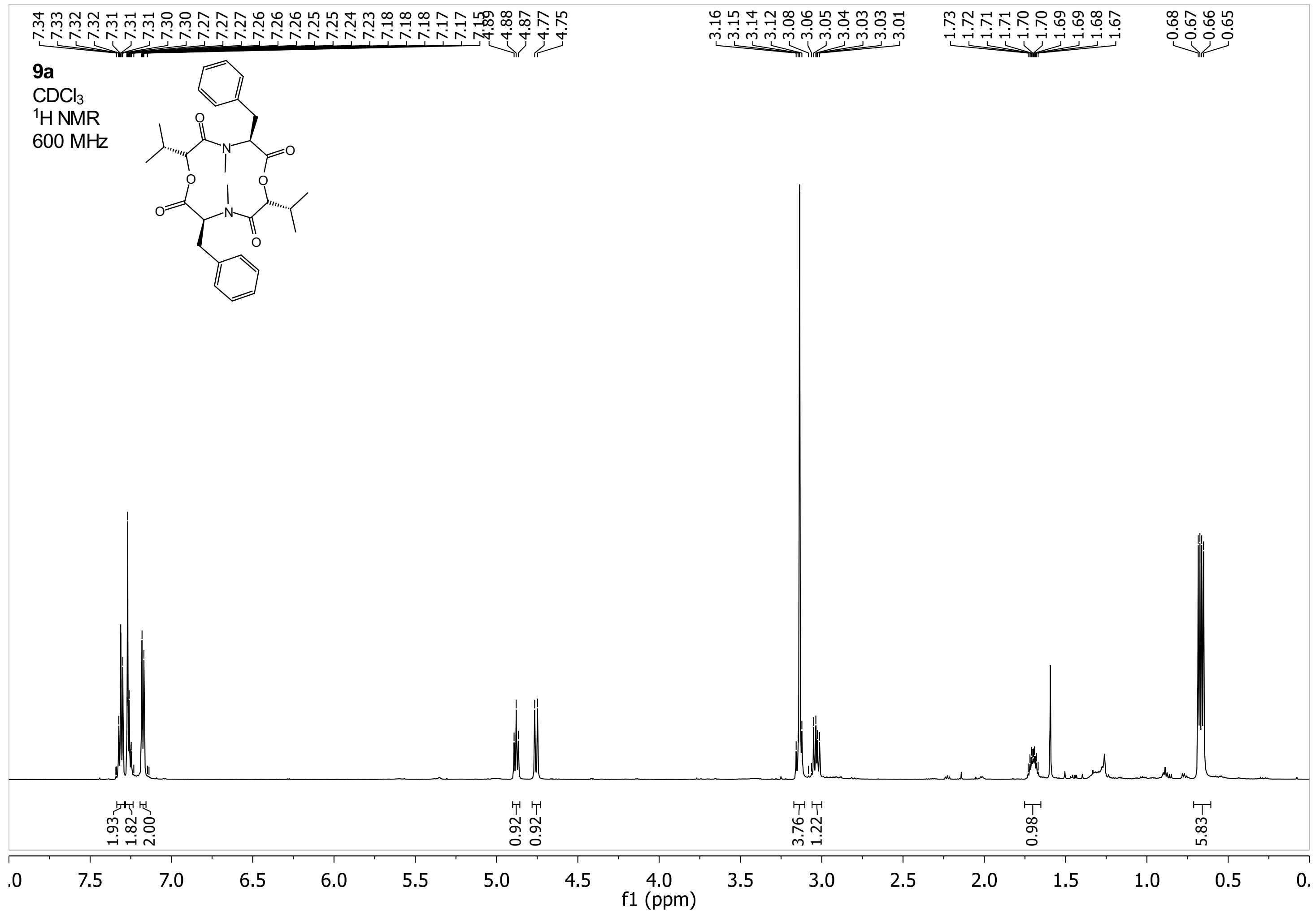
9a
CDCl3
1H NMR
600 MHz
7.34 7.33 7.32 7.32 7.31 7.31 7.31 7.30 7.30 7.27 7.27 7.27 7.26 7.26 7.26 7.25 7.25 7.24 7.23 7.18 7.18 7.18 7.17 7.17 7.15 4.89 4.88 4.87 4.77 4.75
3.16 3.15 3.14 3.12 3.08 3.06 3.05 3.04 3.03 3.03 3.01
1.73 1.72 1.71 1.71 1.70 1.70 1.69 1.69 1.68 1.67
0.68 0.67 0.66 0.65
1.93 1.82 2.00 0.92 0.92 3.76 1.22 0.98 5.83
f1 (ppm)

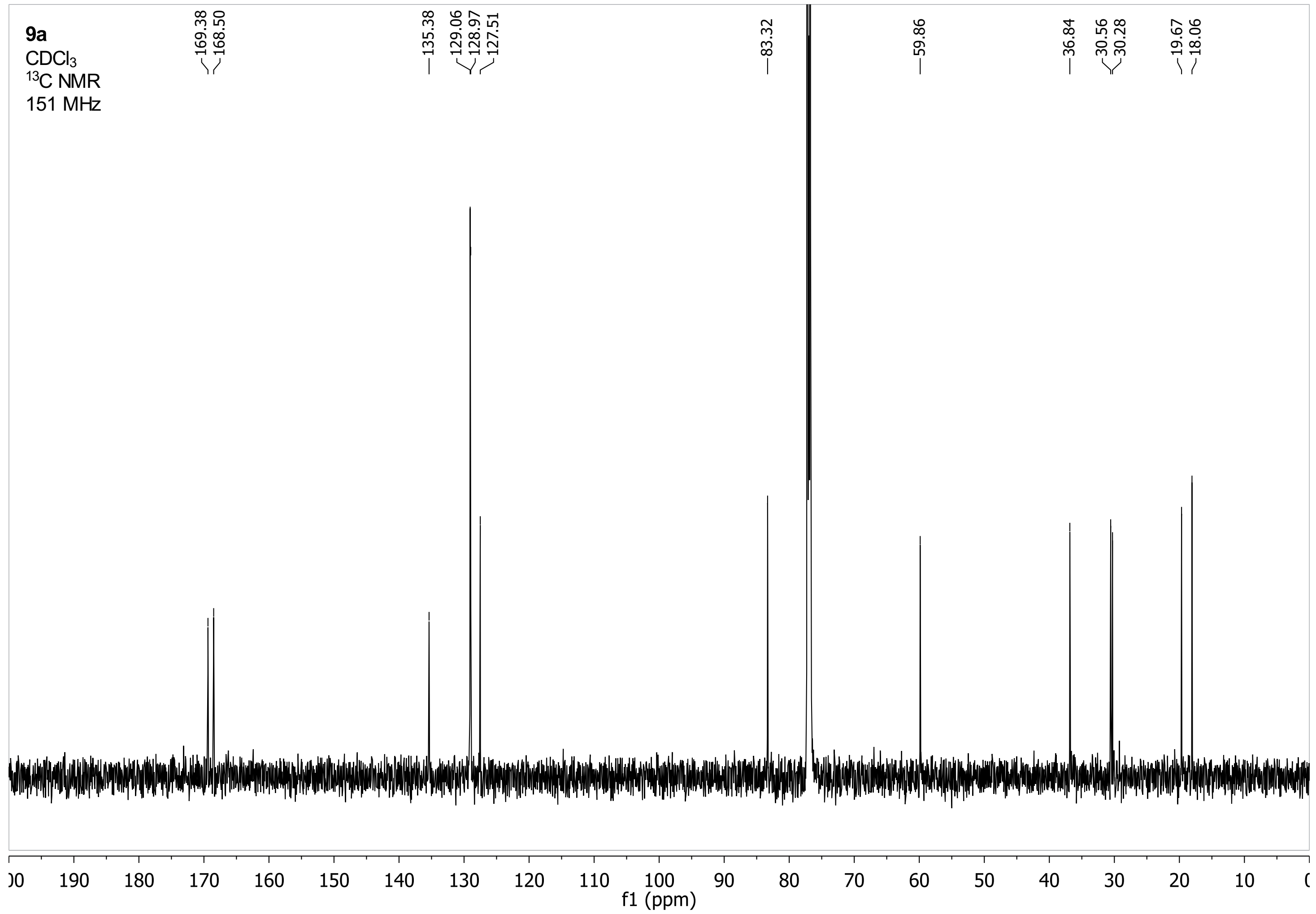
9a
CDCl3
13C NMR
151 MHz
169.38 168.50
135.38 129.06 128.97 127.51
83.32
59.86
36.84 30.56 30.28
19.67 18.06
f1 (ppm)

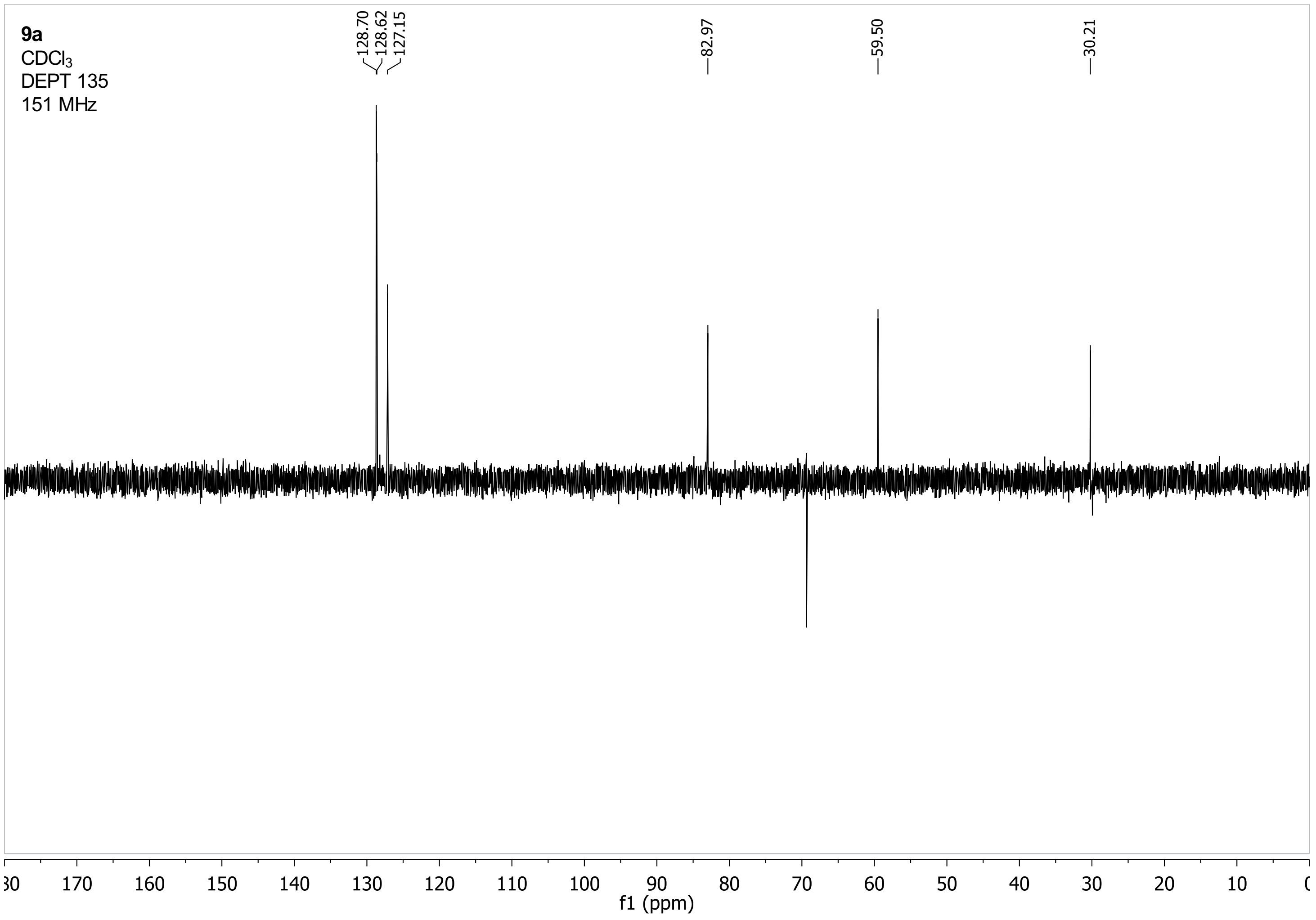
9a
CDCl3
DEPT 135
151 MHz
128.70
128.62
127.15
82.97
59.50
30.21
f1 (ppm)

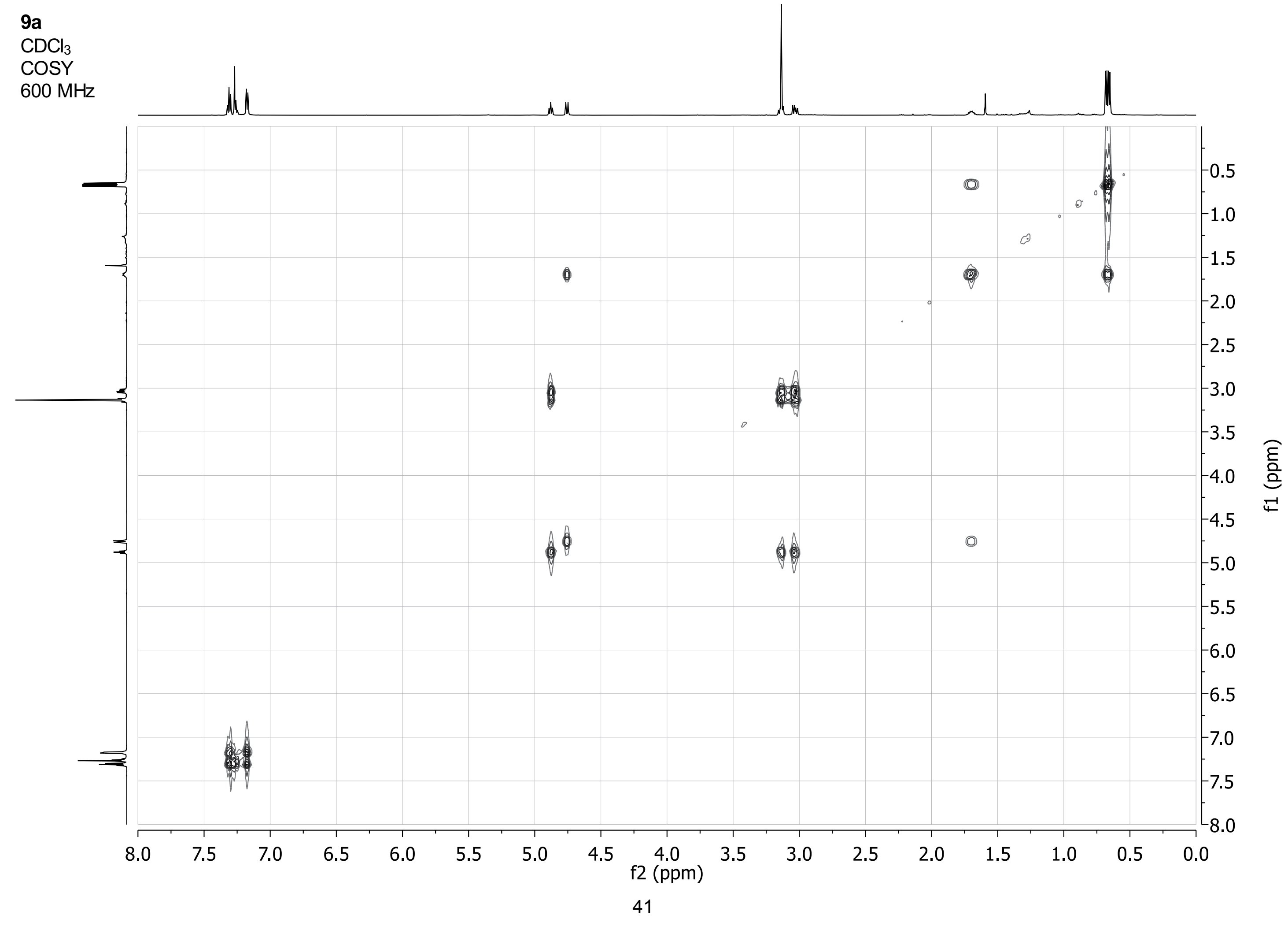
9a
CDCl3
COSY
600 MHz
f2 (ppm)
f1 (ppm)

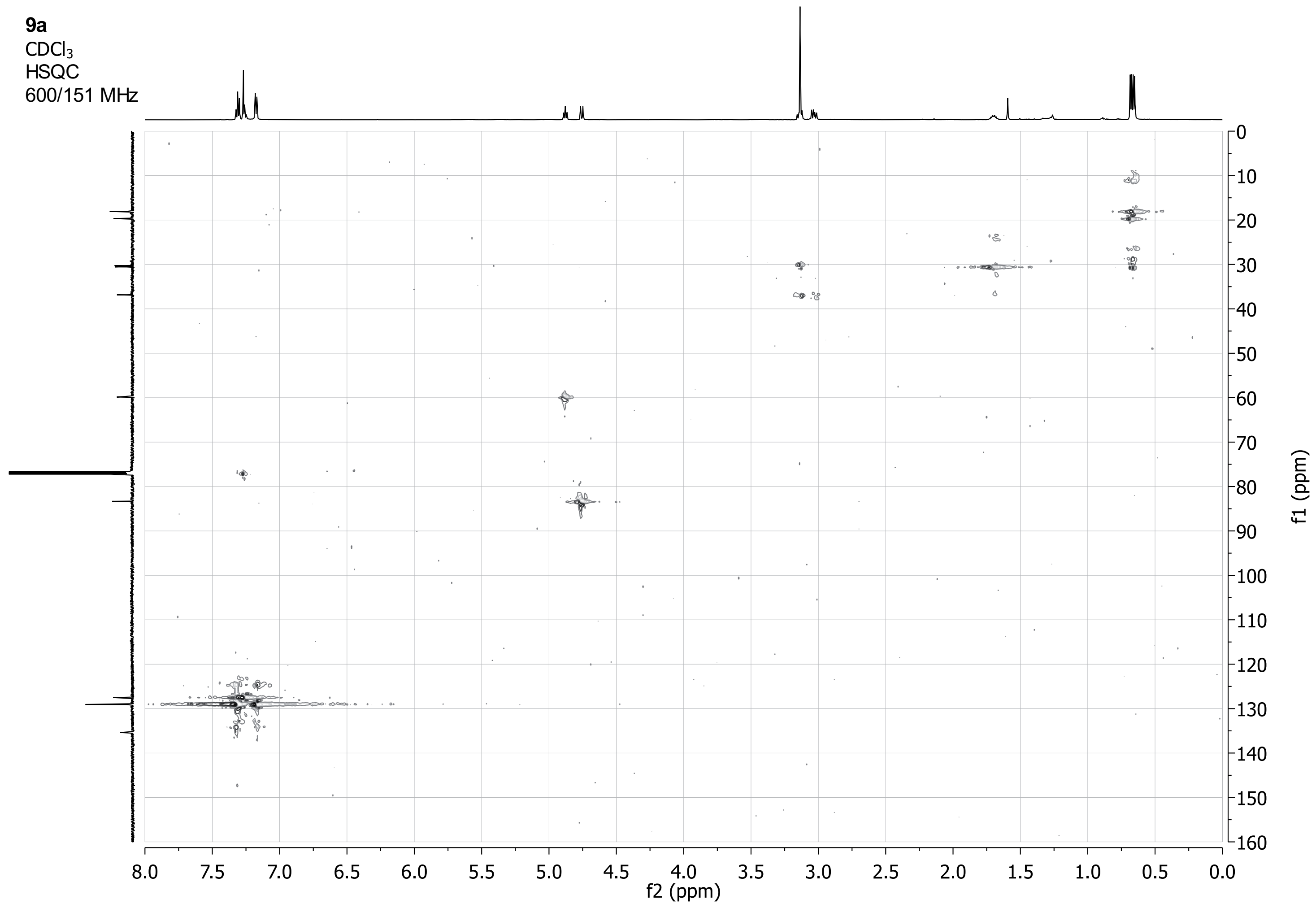

9a
$CDCl_3$
HSQC
600/151 MHz
f1 (ppm)
f2 (ppm)

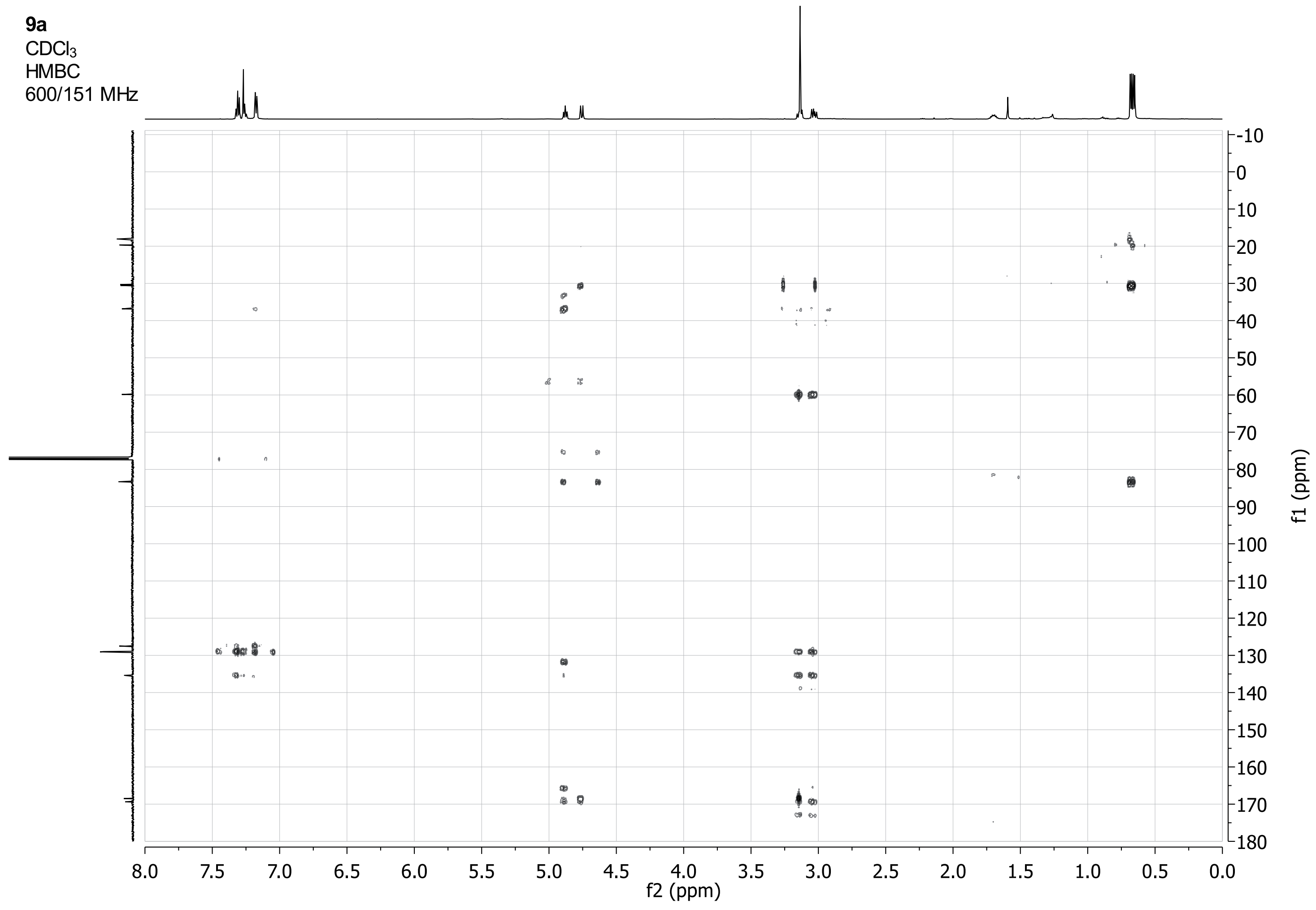

9a
$CDCl_3$
HMBC
600/151 MHz
f1 (ppm)
f2 (ppm)

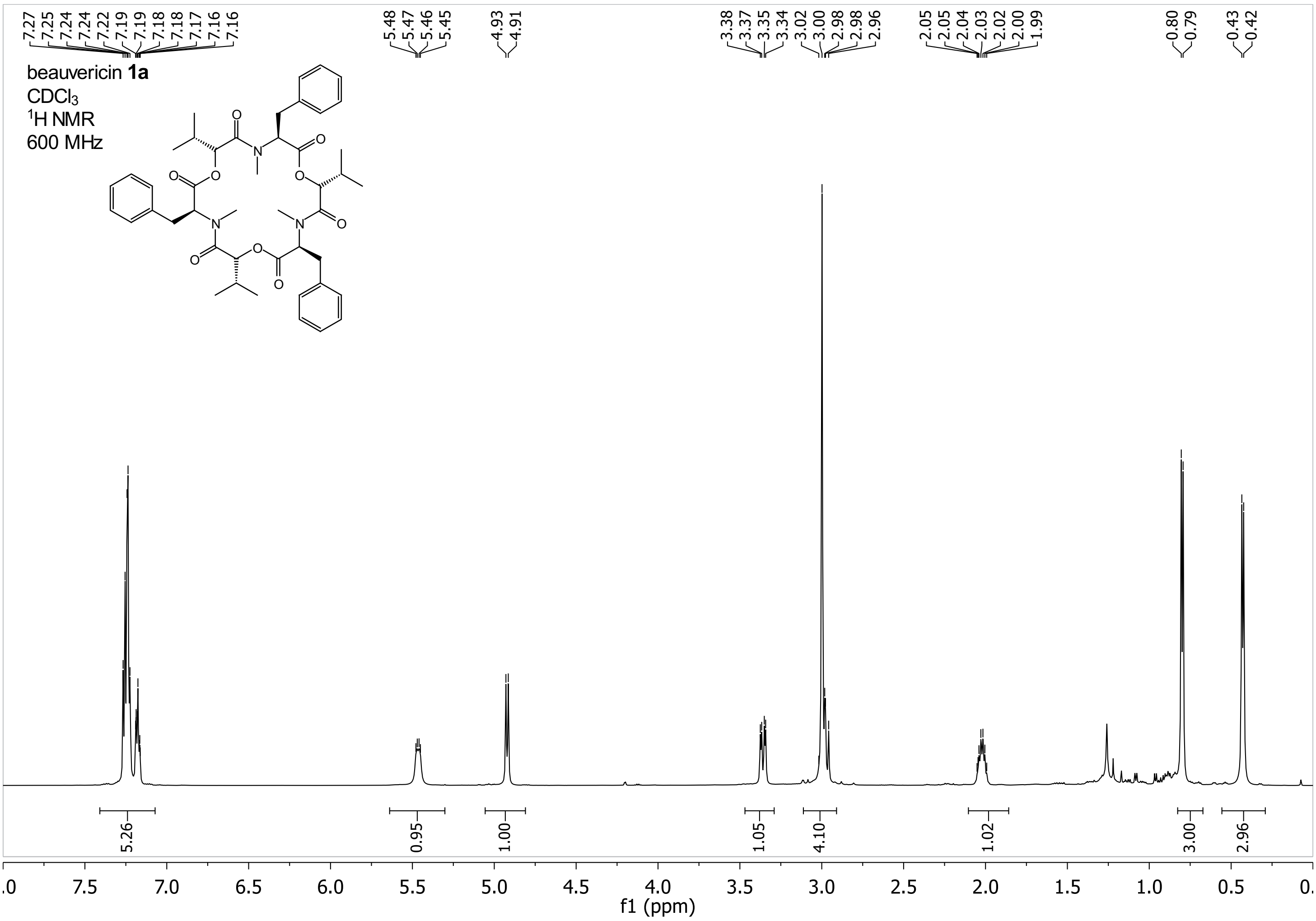
beauvericin 1a
CDCl3
1H NMR
600 MHz
7.27 7.25 7.24 7.24 7.22 7.19 7.19 7.18 7.18 7.17 7.16 7.16
5.48 5.47 5.46 5.45
4.93 4.91
3.38 3.37 3.35 3.34 3.02 3.00 2.98 2.98 2.96
2.05 2.05 2.04 2.03 2.02 2.00 1.99
0.80 0.79
0.43 0.42
5.26
0.95
1.00
1.05
4.10
1.02
3.00
2.96
f1 (ppm)

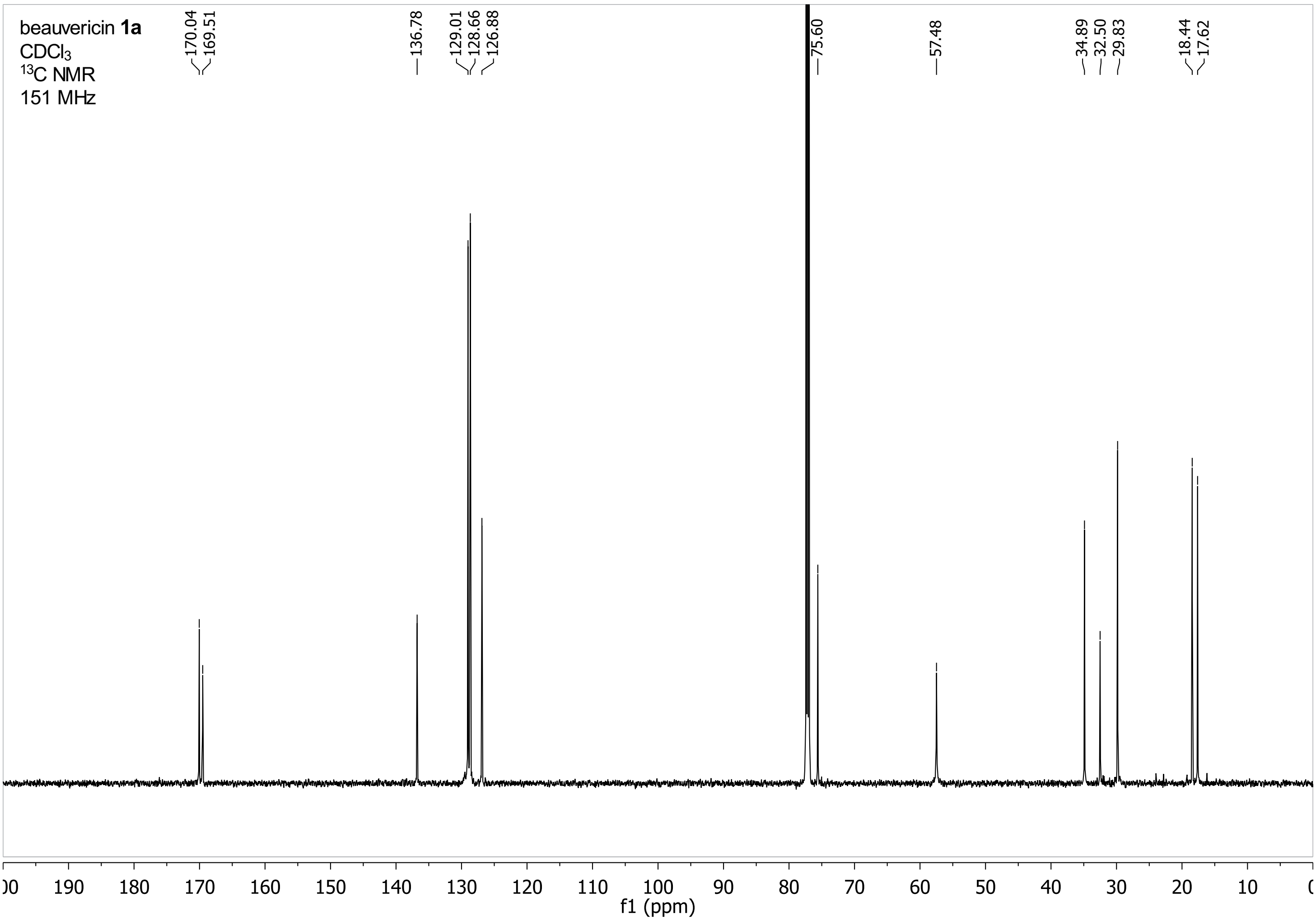
beauvericin 1a
CDCl3
13C NMR
151 MHz
170.04 169.51
136.78
129.01 128.66 126.88
75.60
57.48
34.89 32.50 29.83
18.44 17.62
f1 (ppm)

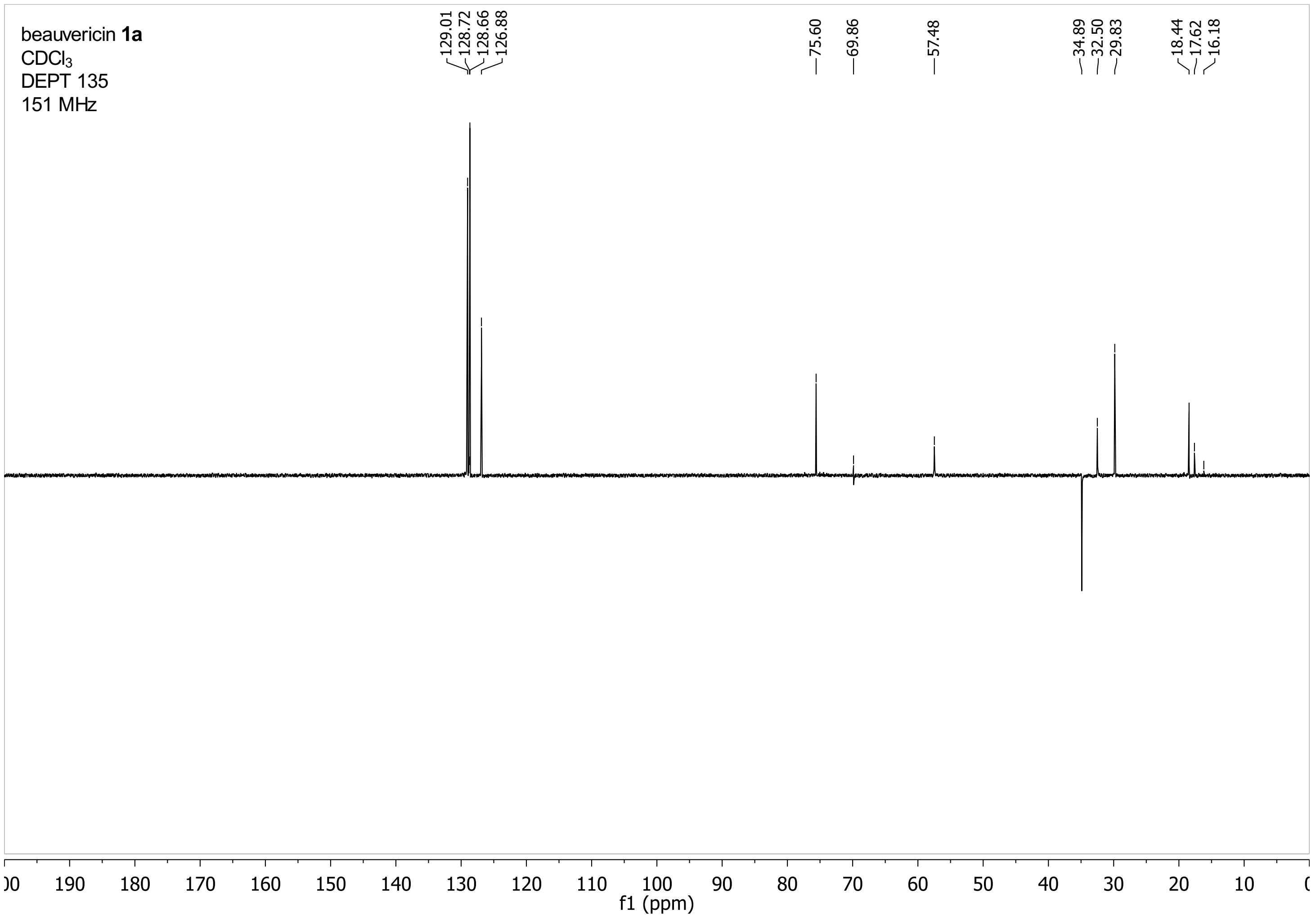
beauvericin **1a**
$CDCl_3$
DEPT 135
151 MHz
129.01
128.72
128.66
126.88
75.60
69.86
57.48
34.89
32.50
29.83
18.44
17.62
16.18
f1 (ppm)

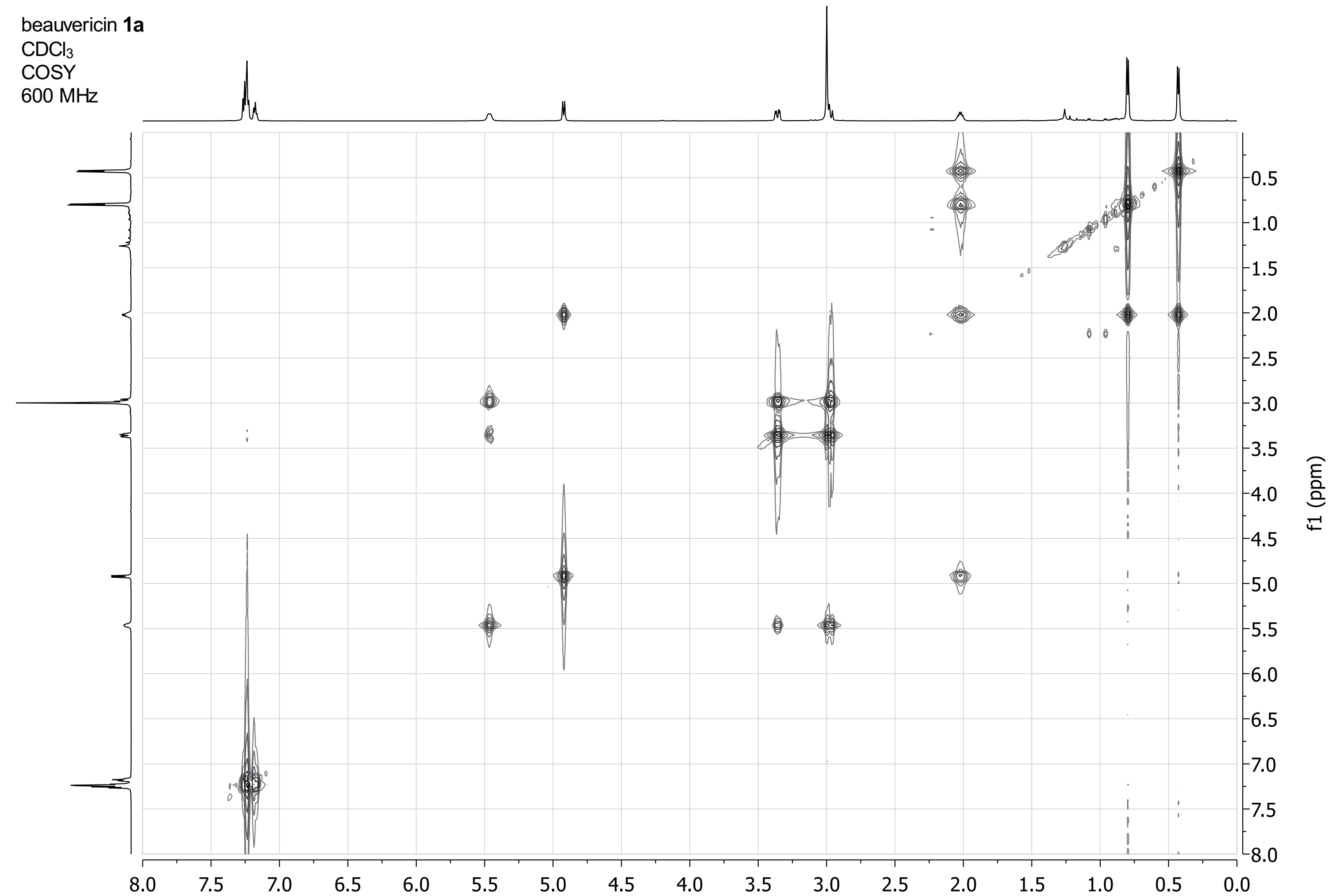
beauvericin **1a**
$CDCl_3$
COSY
600 MHz
f2 (ppm)
f1 (ppm)

beauvericin **1a**
$CDCl_3$
HSQC
600/151 MHz

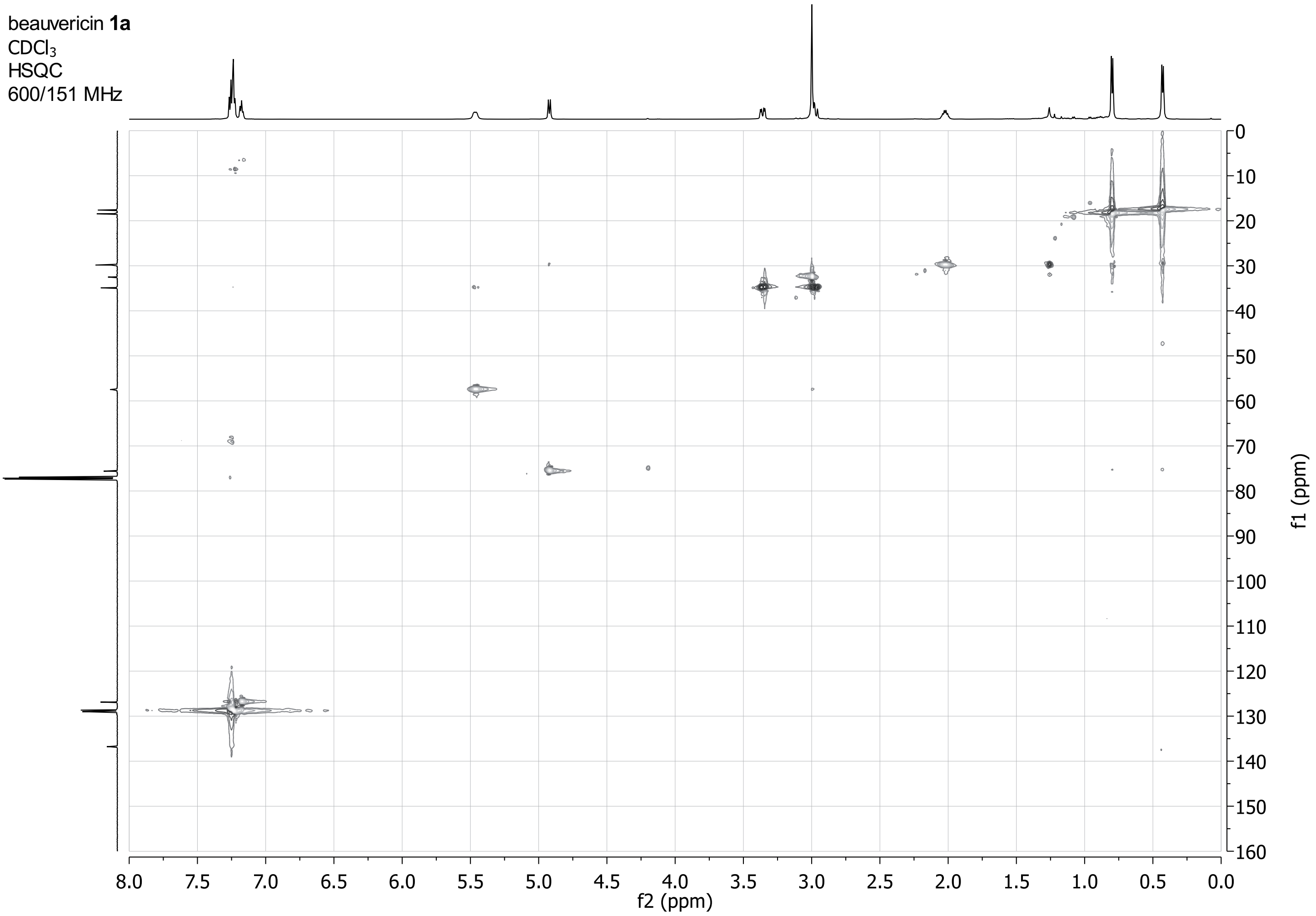

beauvericin **1a**
$CDCl_3$
HMBC
600/151 MHz

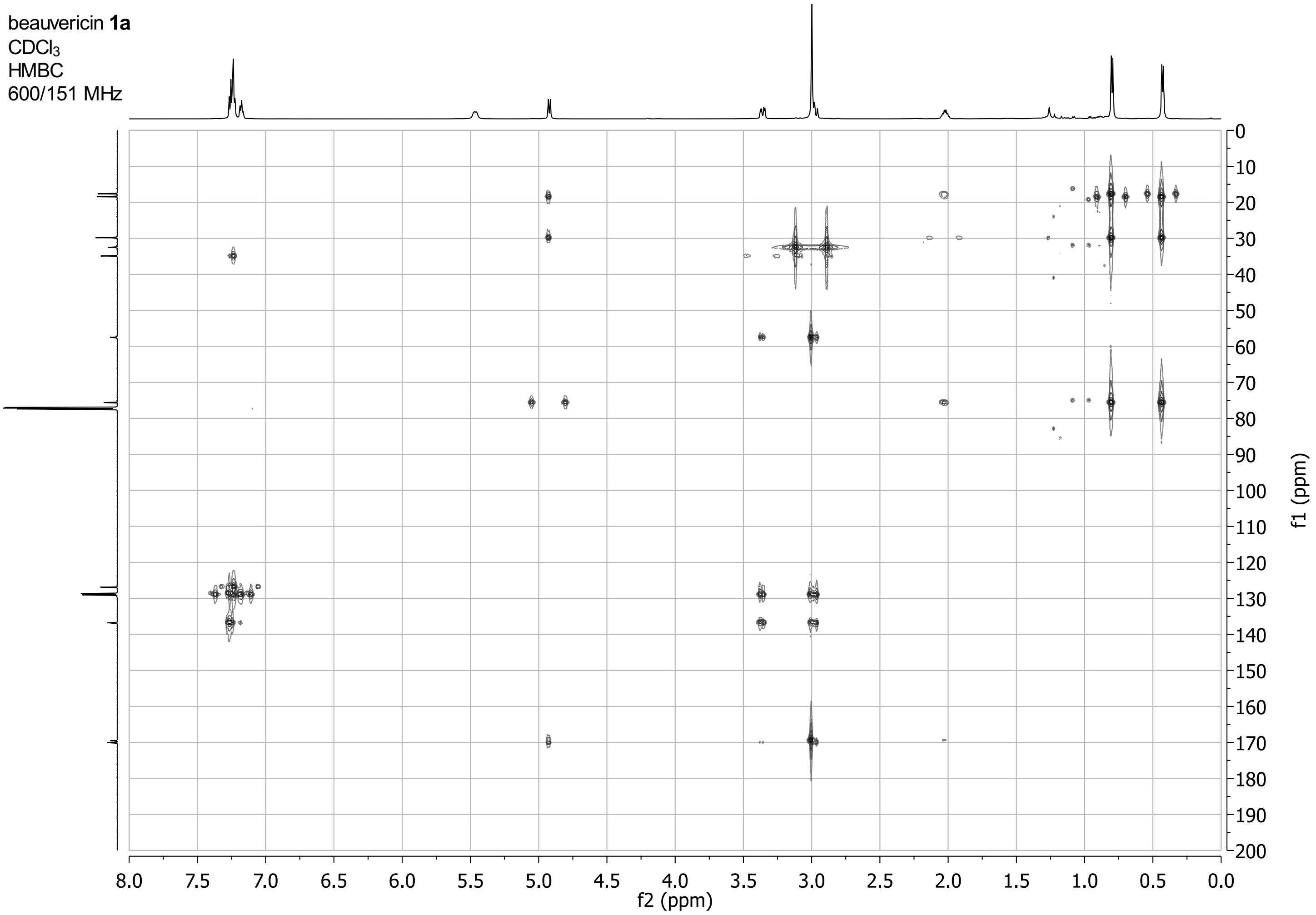

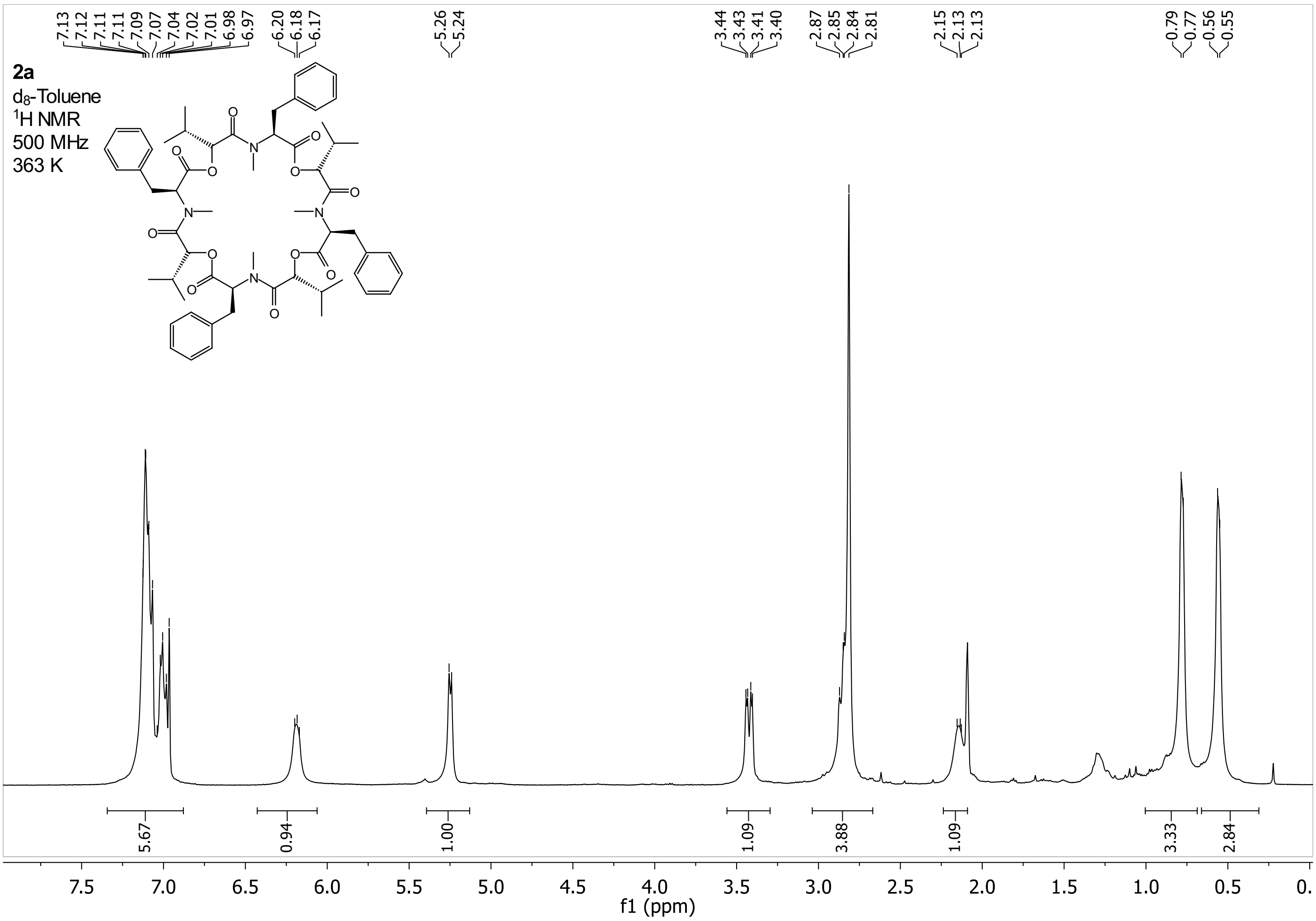
7.13 7.12 7.11 7.11 7.09 7.07 7.04 7.02 7.01 6.98 6.97 6.20 6.18 6.17 5.26 5.24 3.44 3.43 3.41 3.40 2.87 2.85 2.84 2.81 2.15 2.13 2.13 0.79 0.77 0.56 0.55
2a
d8-Toluene
1H NMR
500 MHz
363 K
5.67 0.94 1.00 1.09 3.88 1.09 3.33 2.84
f1 (ppm)

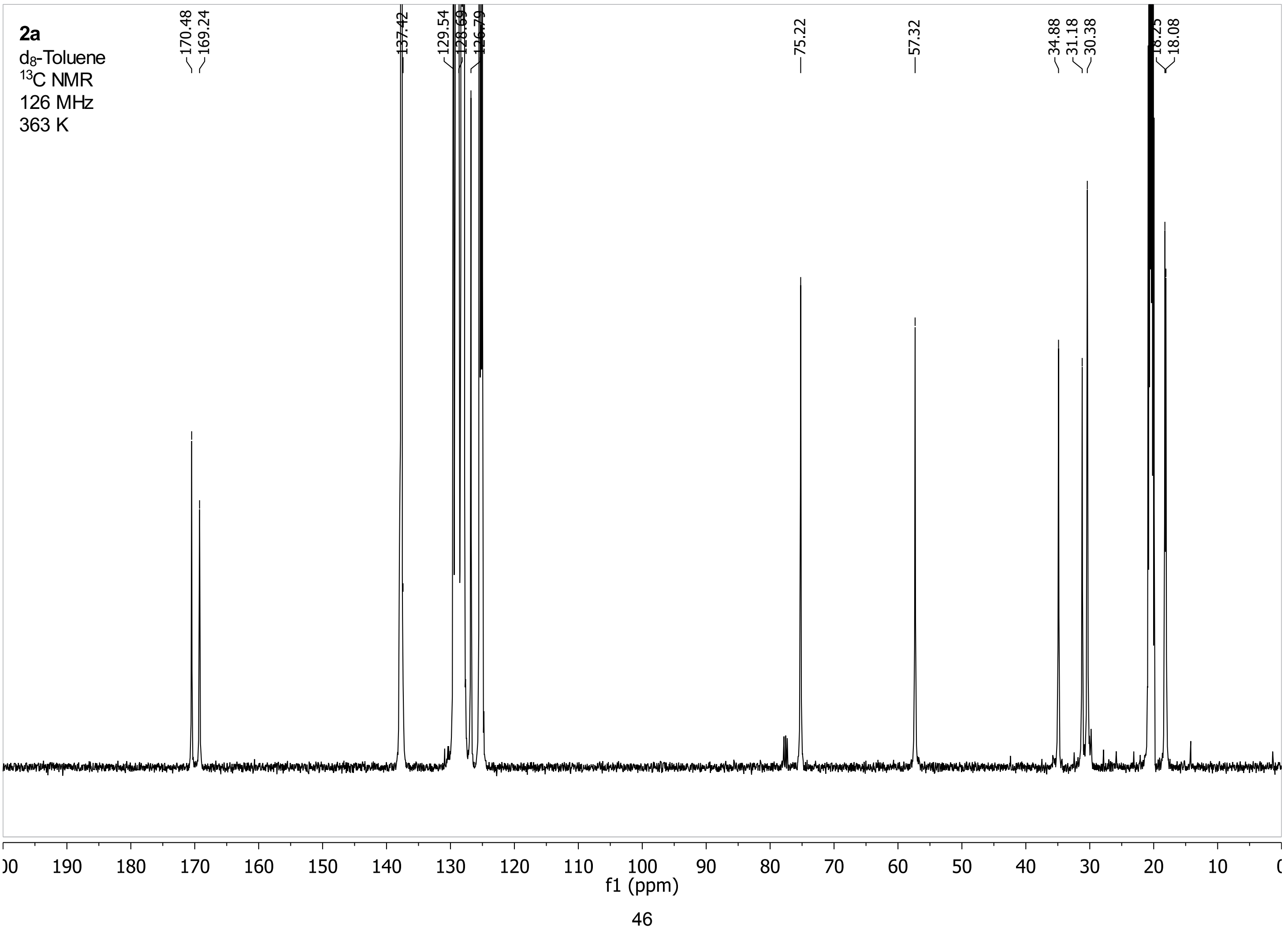
2a
d8-Toluene
13C NMR
126 MHz
363 K
170.48 169.24 137.42 129.54 128.69 126.79 75.22 57.32 34.88 31.18 30.38 18.25 18.08
f1 (ppm)

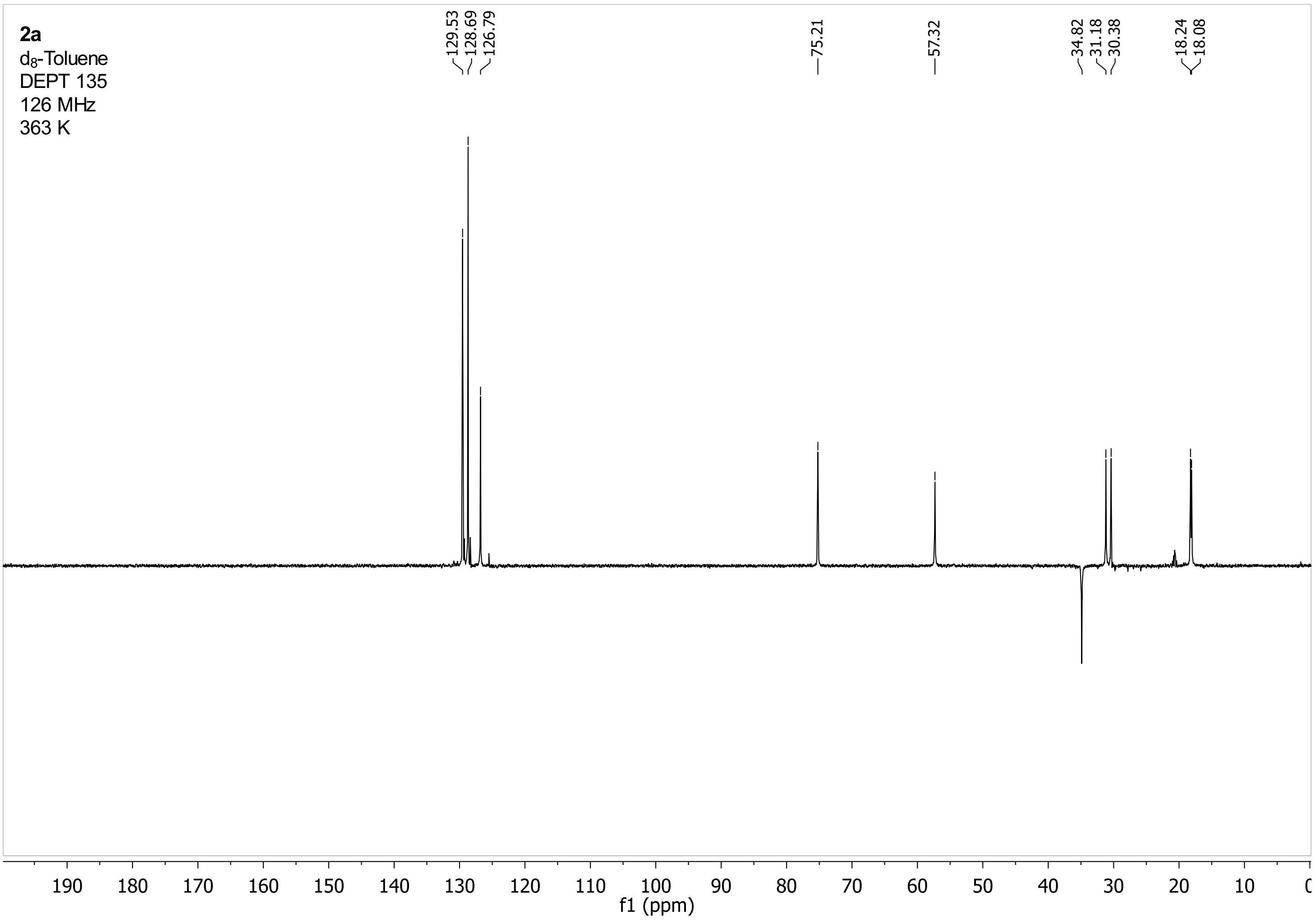
2a
d8-Toluene
DEPT 135
126 MHz
363 K
129.53
128.69
126.79
75.21
57.32
34.82
31.18
30.38
18.24
18.08
f1 (ppm)

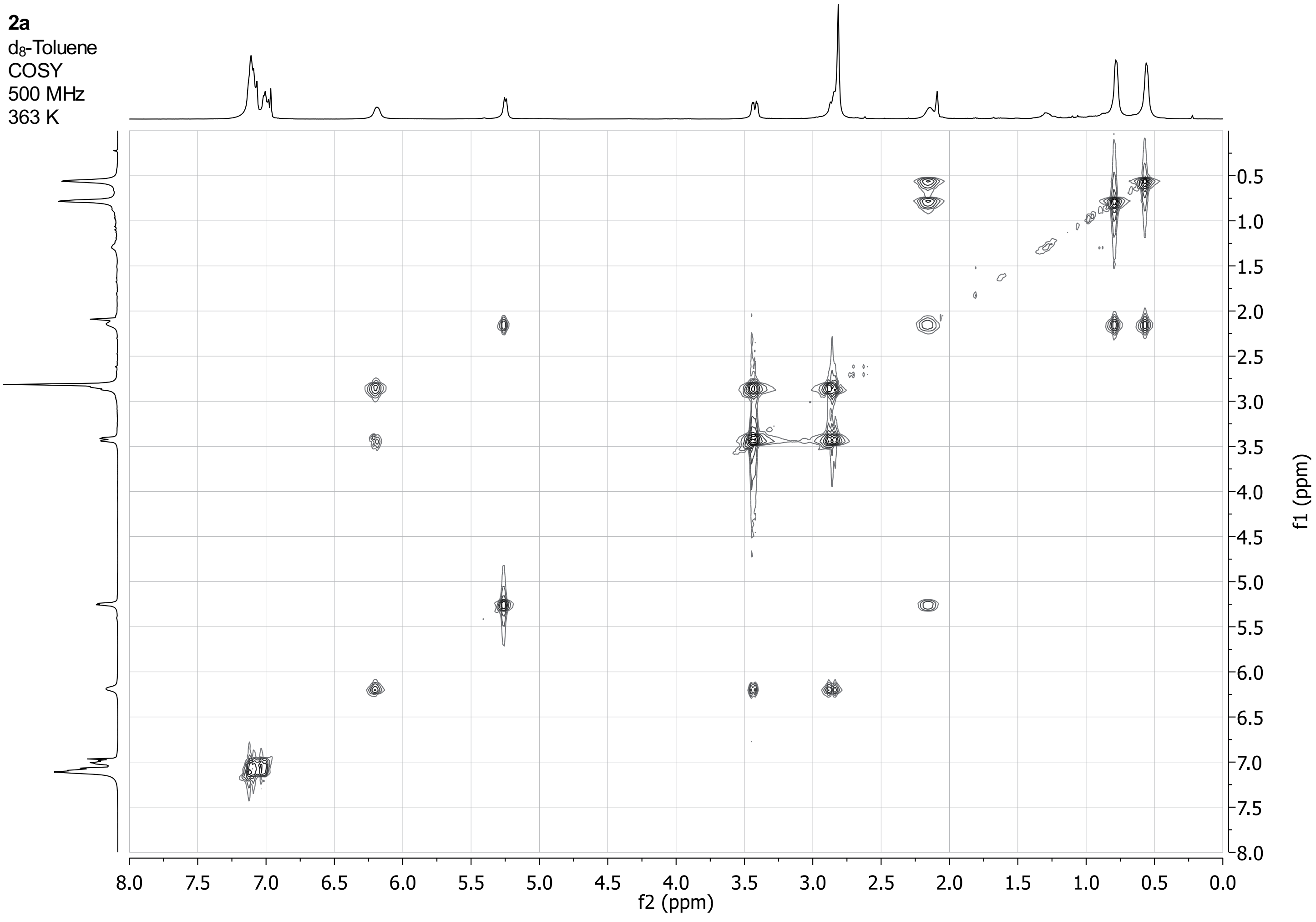
2a
d8-Toluene
COSY
500 MHz
363 K
f2 (ppm)
f1 (ppm)

2a
d_8-Toluene
HSQC
500/126 MHz
363 K

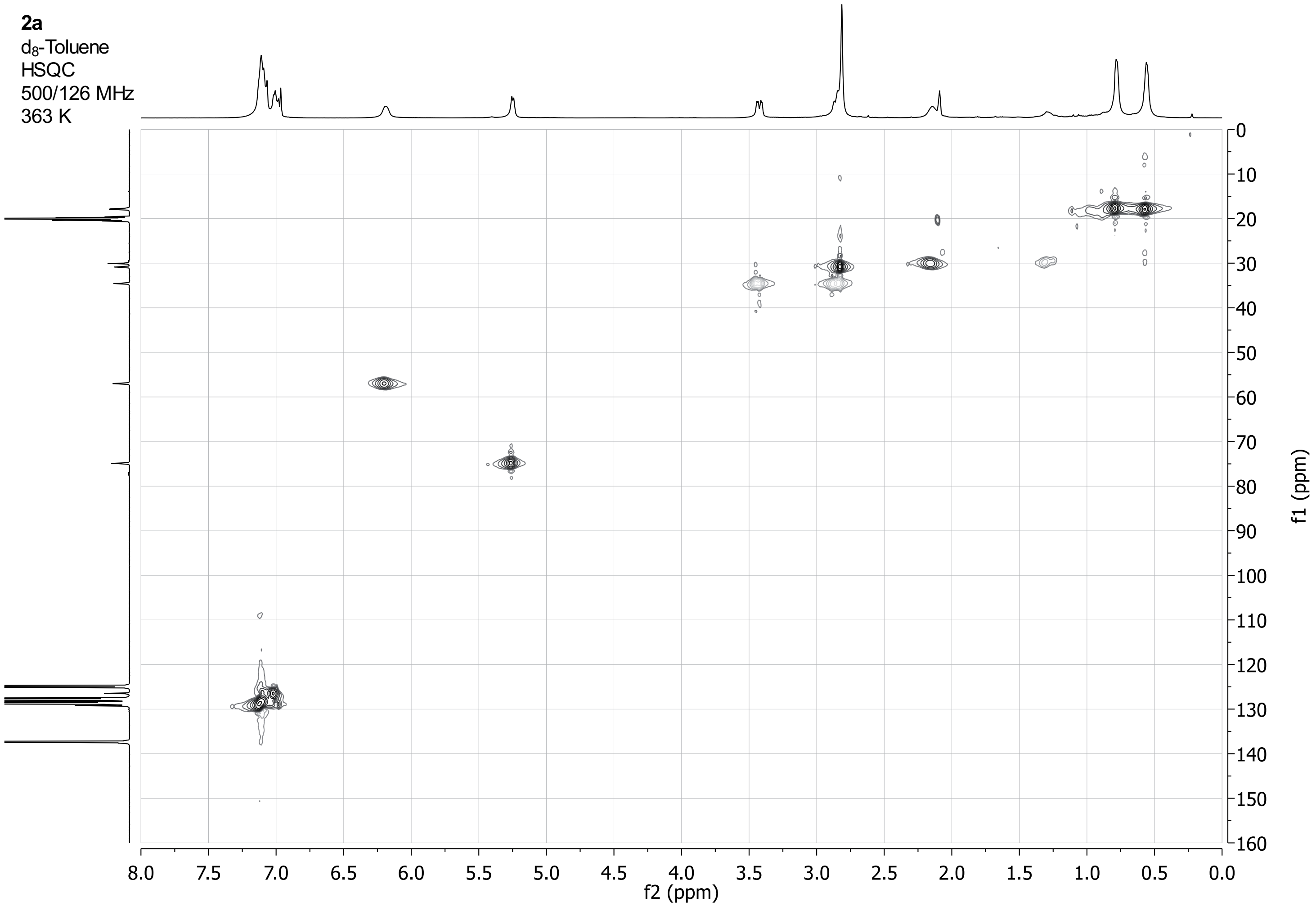

2a
d_8-Toluene
HMBC
500/126 MHz
363 K

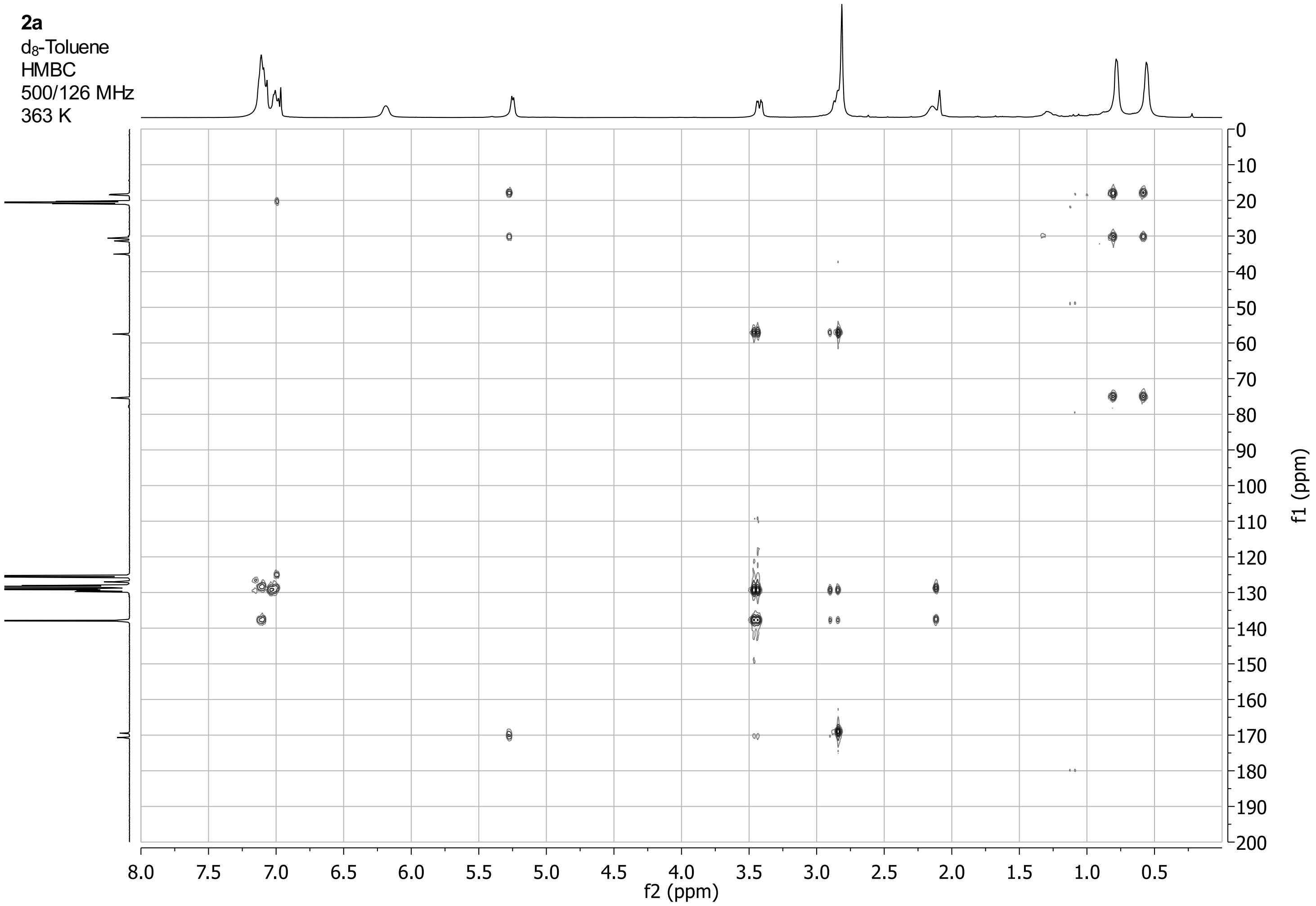

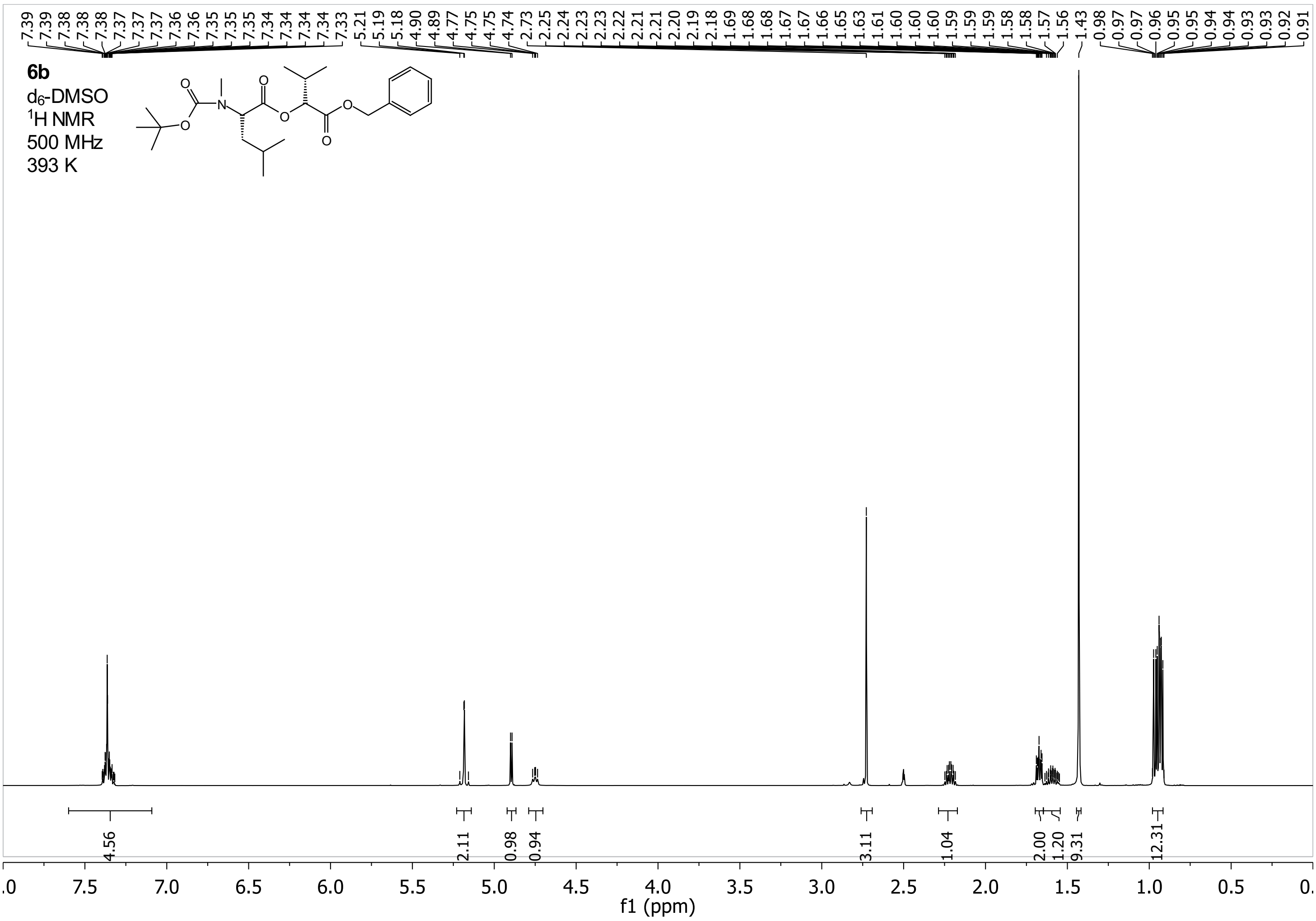
6b
d6-DMSO
1H NMR
500 MHz
393 K
f1 (ppm)

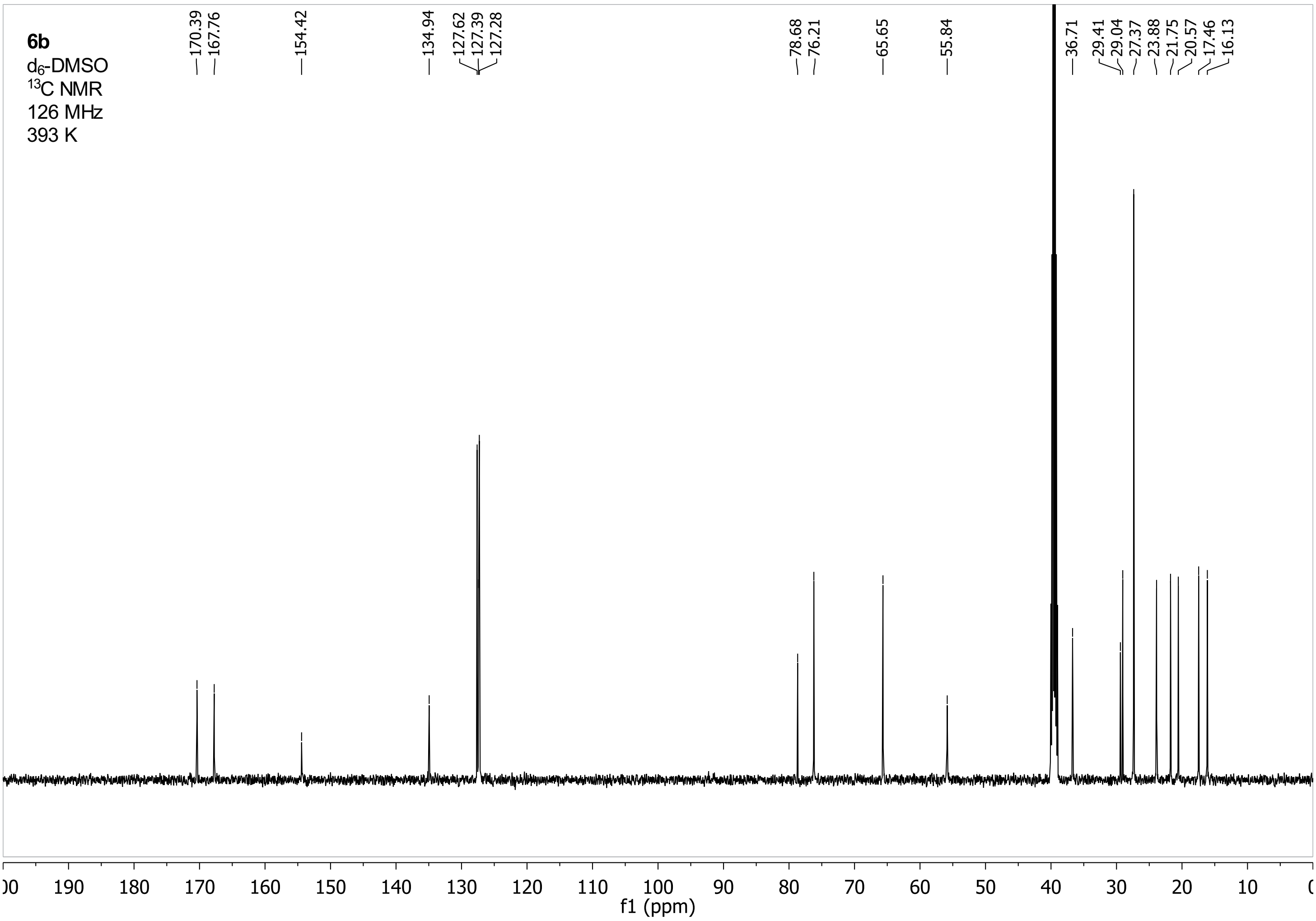
6b
d6-DMSO
13C NMR
126 MHz
393 K
f1 (ppm)

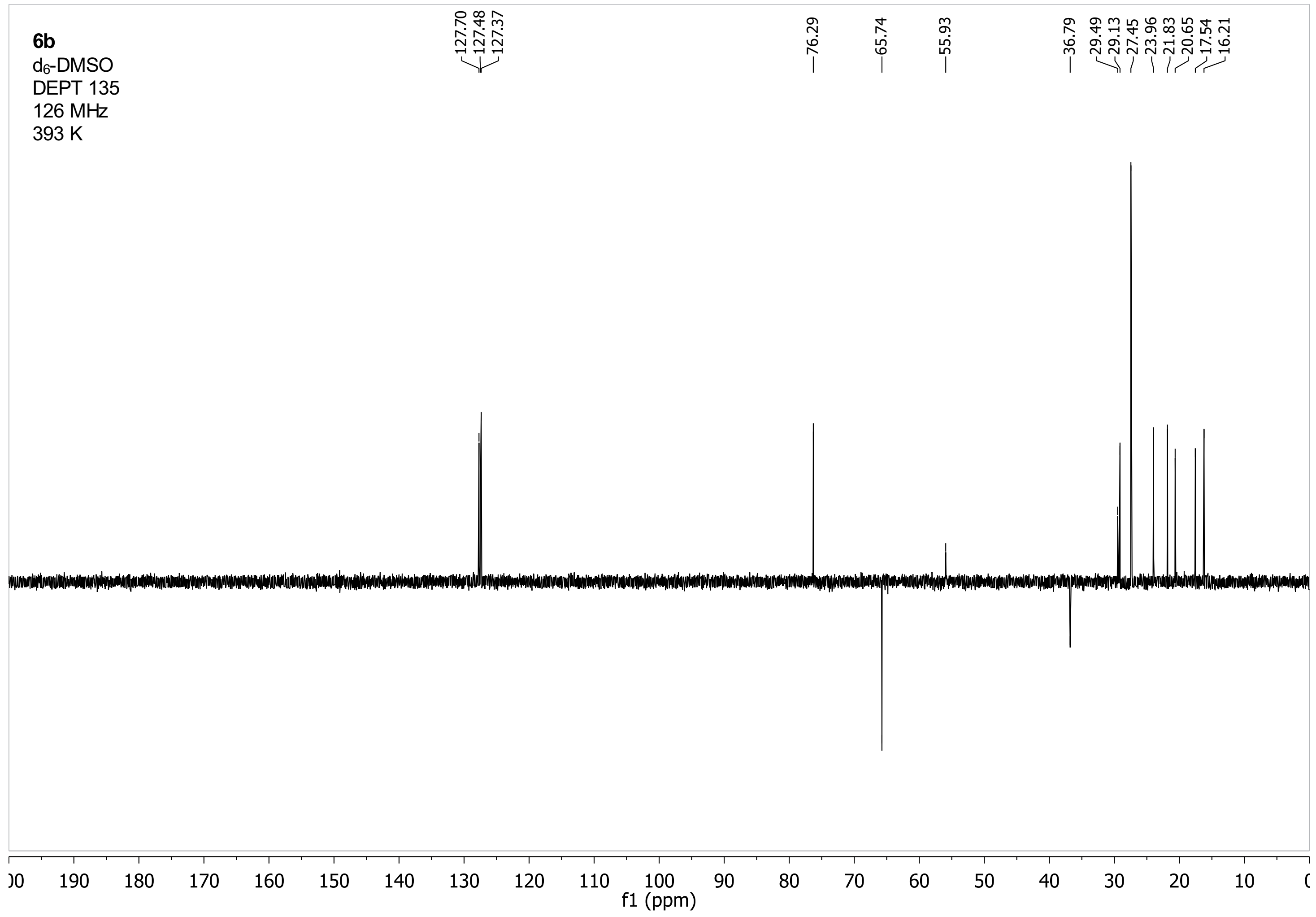
6b
d6-DMSO
DEPT 135
126 MHz
393 K
127.70
127.48
127.37
76.29
65.74
55.93
36.79
29.49
29.13
27.45
23.96
21.83
20.65
17.54
16.21
f1 (ppm)

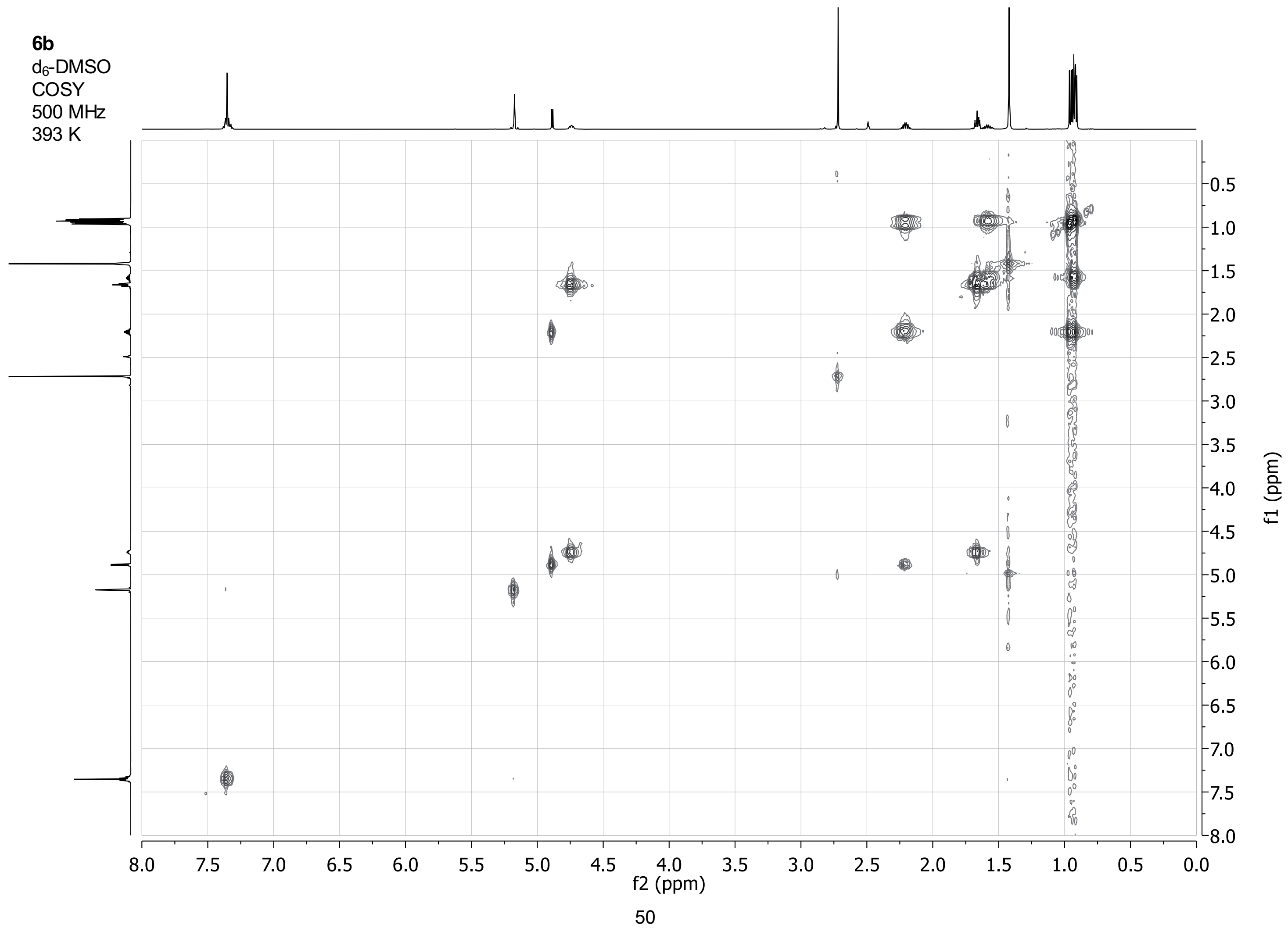
6b
d6-DMSO
COSY
500 MHz
393 K
f2 (ppm)
f1 (ppm)

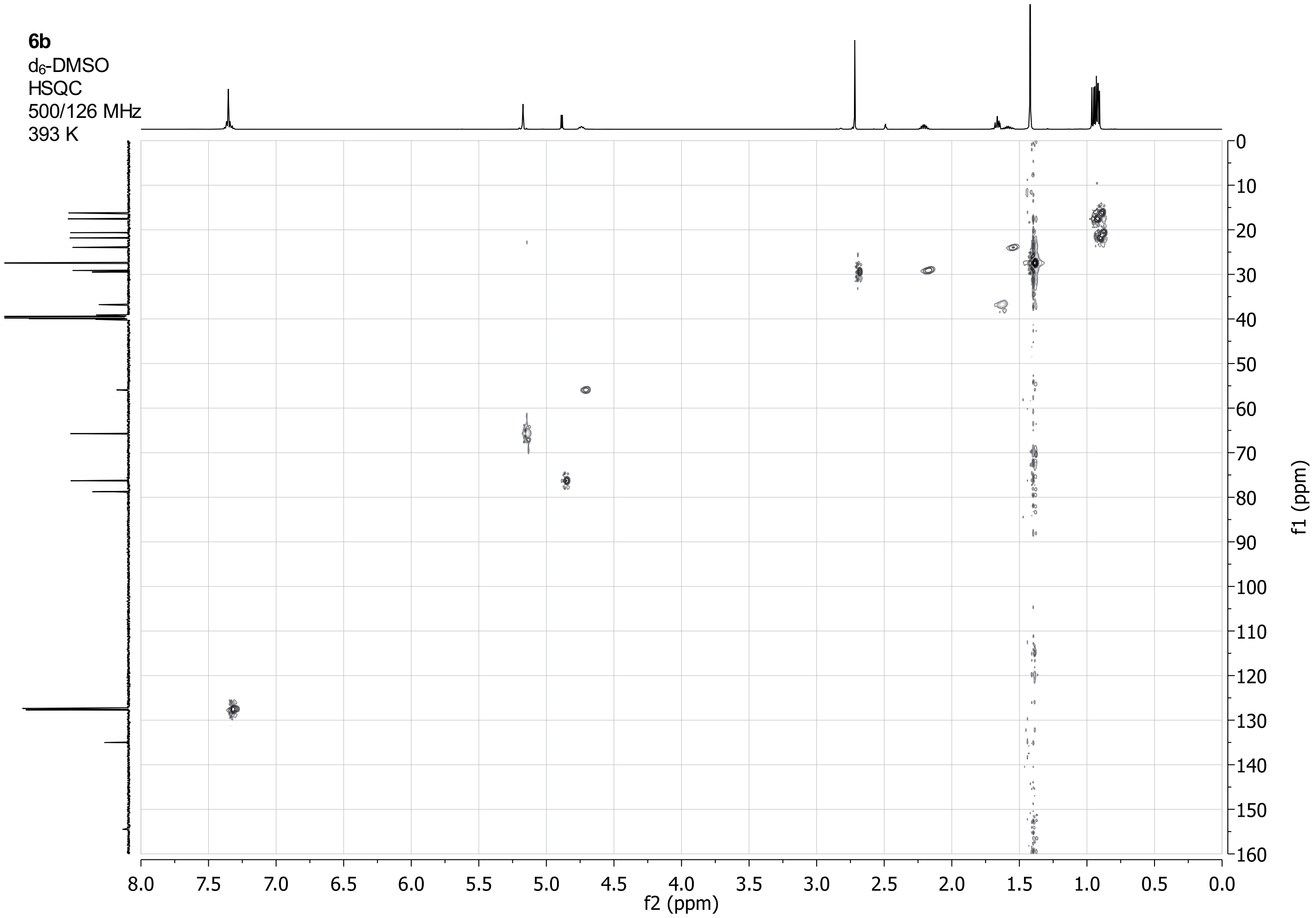
6b
d6-DMSO
HSQC
500/126 MHz
393 K
f1 (ppm)
f2 (ppm)

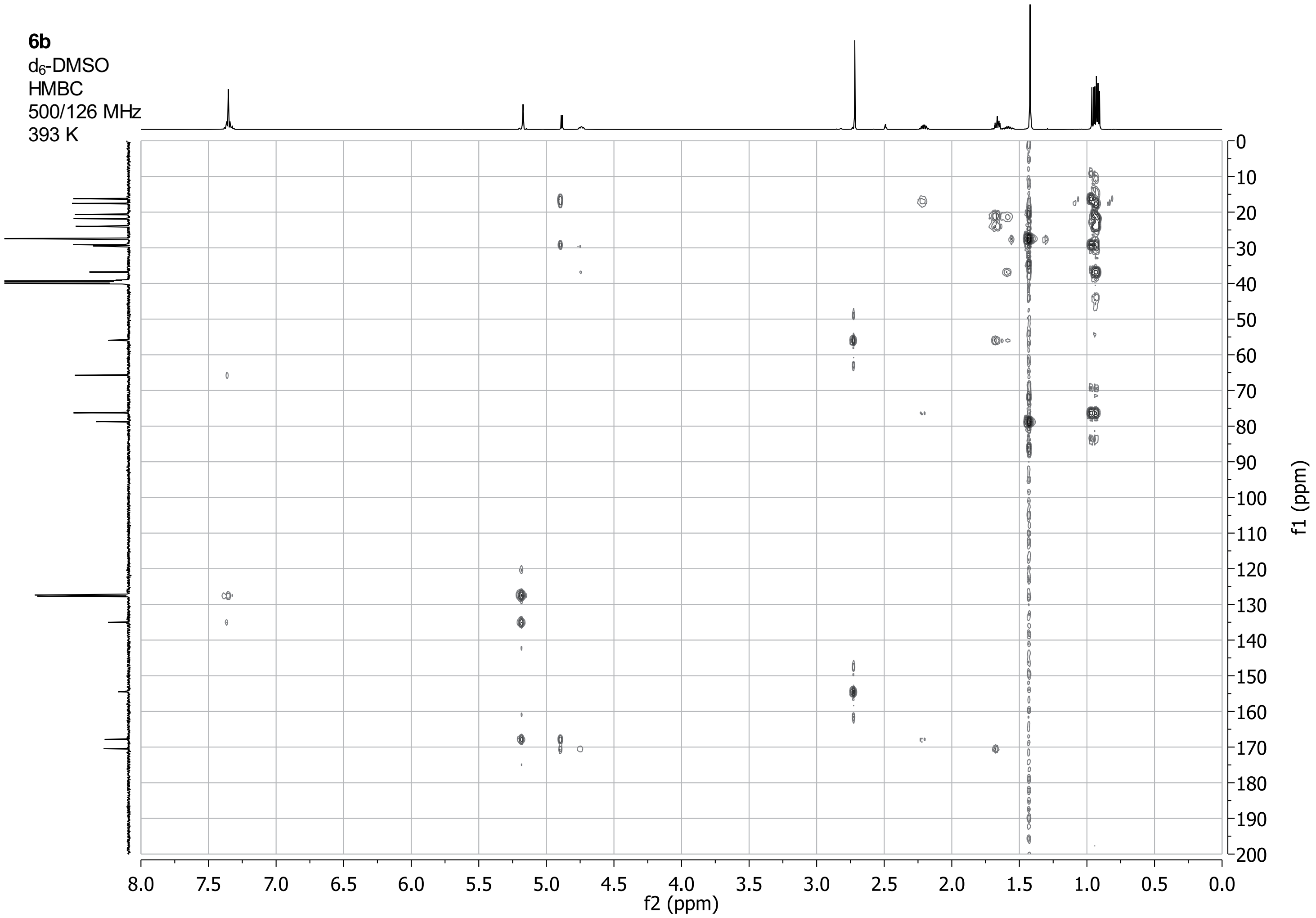
6b
d6-DMSO
HMBC
500/126 MHz
393 K
f1 (ppm)
f2 (ppm)

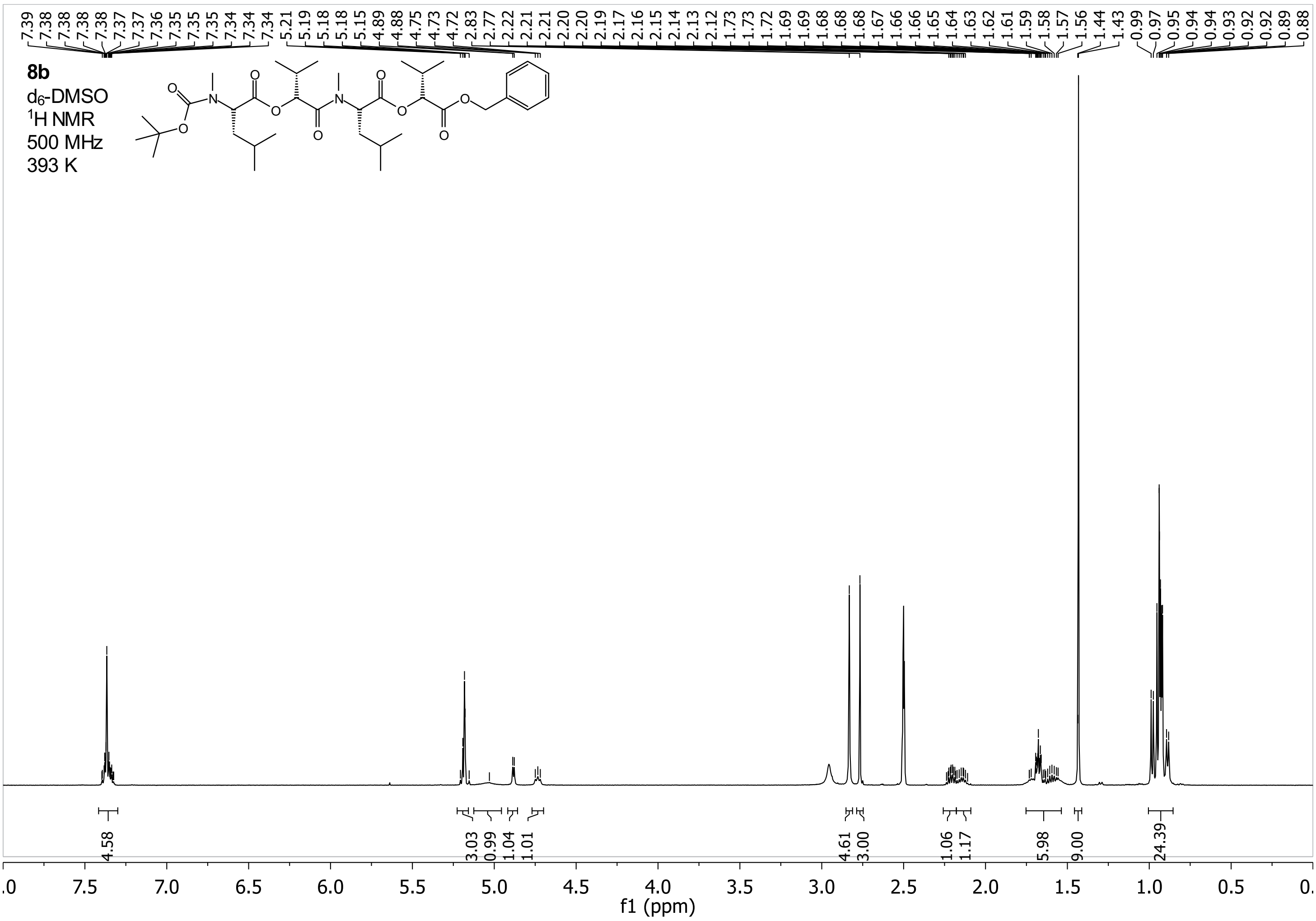
8b
d6-DMSO
1H NMR
500 MHz
393 K
7.39 7.38 7.38 7.38 7.38 7.37 7.37 7.36 7.35 7.35 7.35 7.34 7.34 7.34 5.21 5.19 5.18 5.18 5.15 4.89 4.88 4.75 4.73 4.72 2.83 2.77 2.22 2.22 2.21 2.21 2.20 2.20 2.19 2.17 2.16 2.15 2.14 2.13 2.12 1.73 1.73 1.72 1.69 1.69 1.68 1.68 1.68 1.67 1.66 1.66 1.65 1.64 1.63 1.62 1.61 1.59 1.58 1.57 1.56 1.44 1.43 0.99 0.97 0.95 0.94 0.94 0.93 0.92 0.92 0.89 0.88
4.58 3.03 0.99 1.04 1.01 4.61 3.00 1.06 1.17 5.98 9.00 24.39
7.5 7.0 6.5 6.0 5.5 5.0 4.5 4.0 3.5 3.0 2.5 2.0 1.5 1.0 0.5
f1 (ppm)

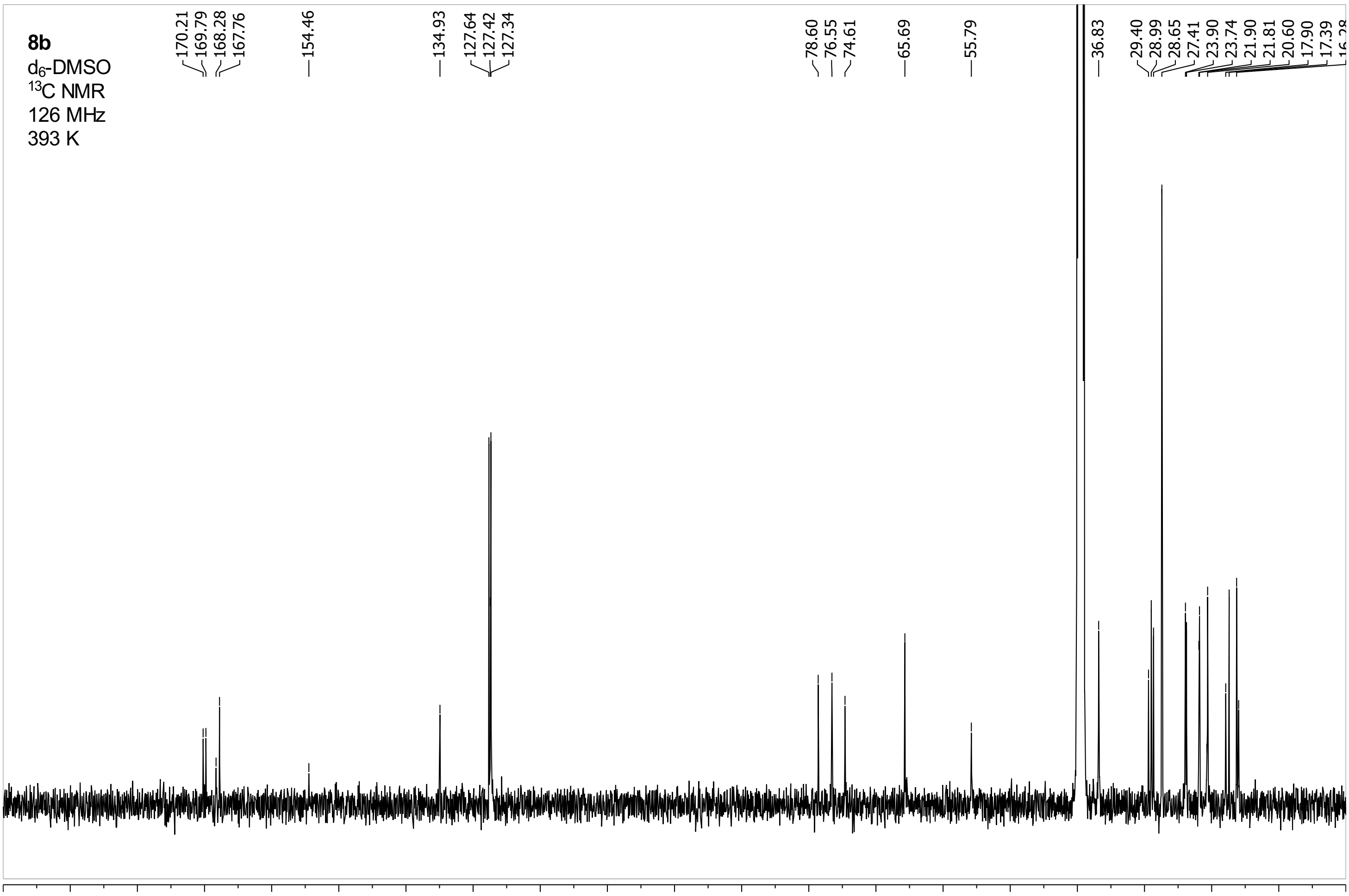
8b
d6-DMSO
13C NMR
126 MHz
393 K
170.21 169.79 168.28 167.76 154.46 134.93 127.64 127.42 127.34 78.60 76.55 74.61 65.69 55.79 36.83 29.40 28.99 28.65 27.41 23.90 23.74 21.90 21.81 20.60 17.90 17.39
190 180 170 160 150 140 130 120 110 100 90 80 70 60 50 40 30 20 10
f1 (ppm)

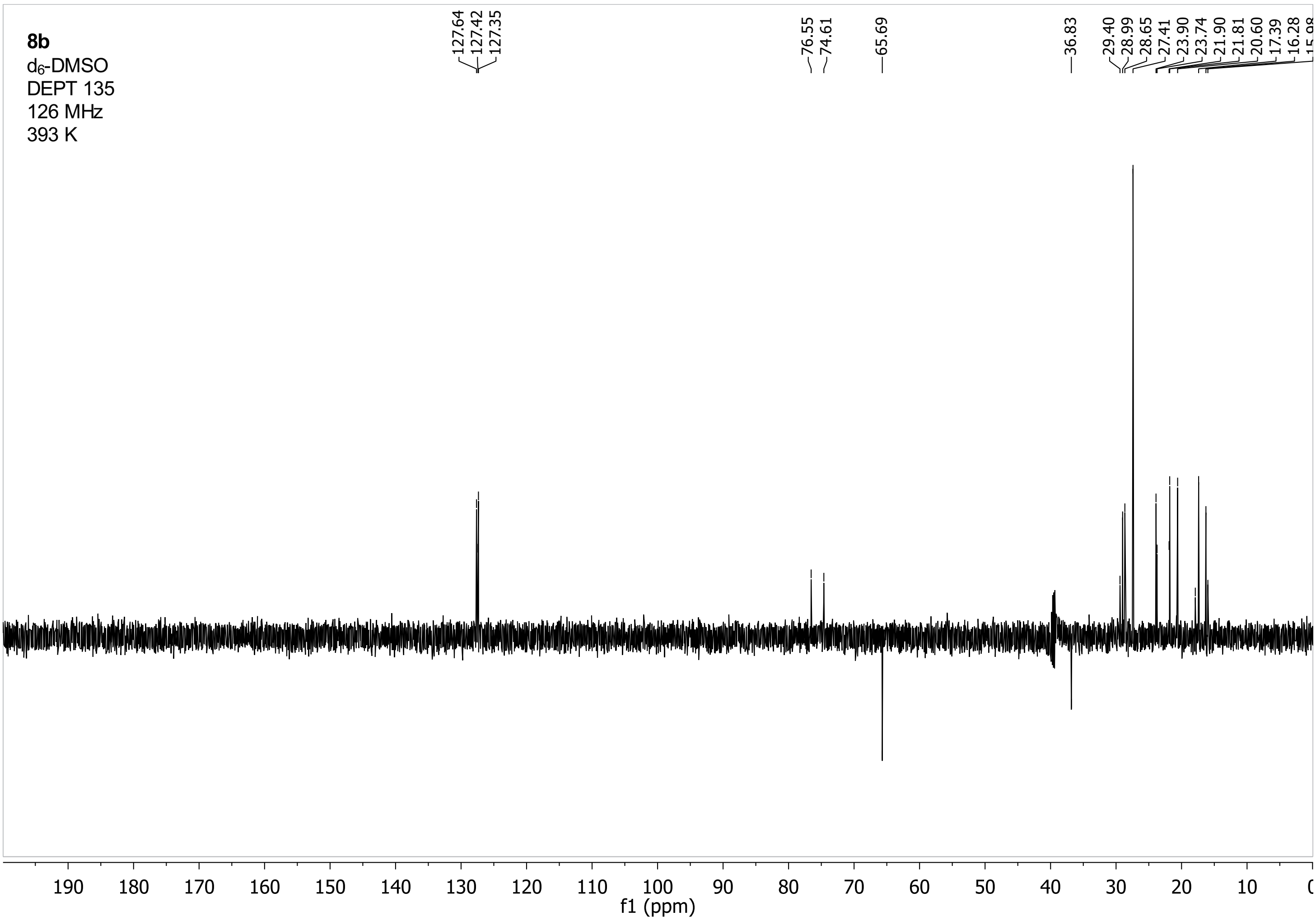
8b
d6-DMSO
DEPT 135
126 MHz
393 K
127.64
127.42
127.35
76.55
74.61
65.69
36.83
29.40
28.99
28.65
27.41
23.90
23.74
21.90
21.81
20.60
17.39
16.28
f1 (ppm)

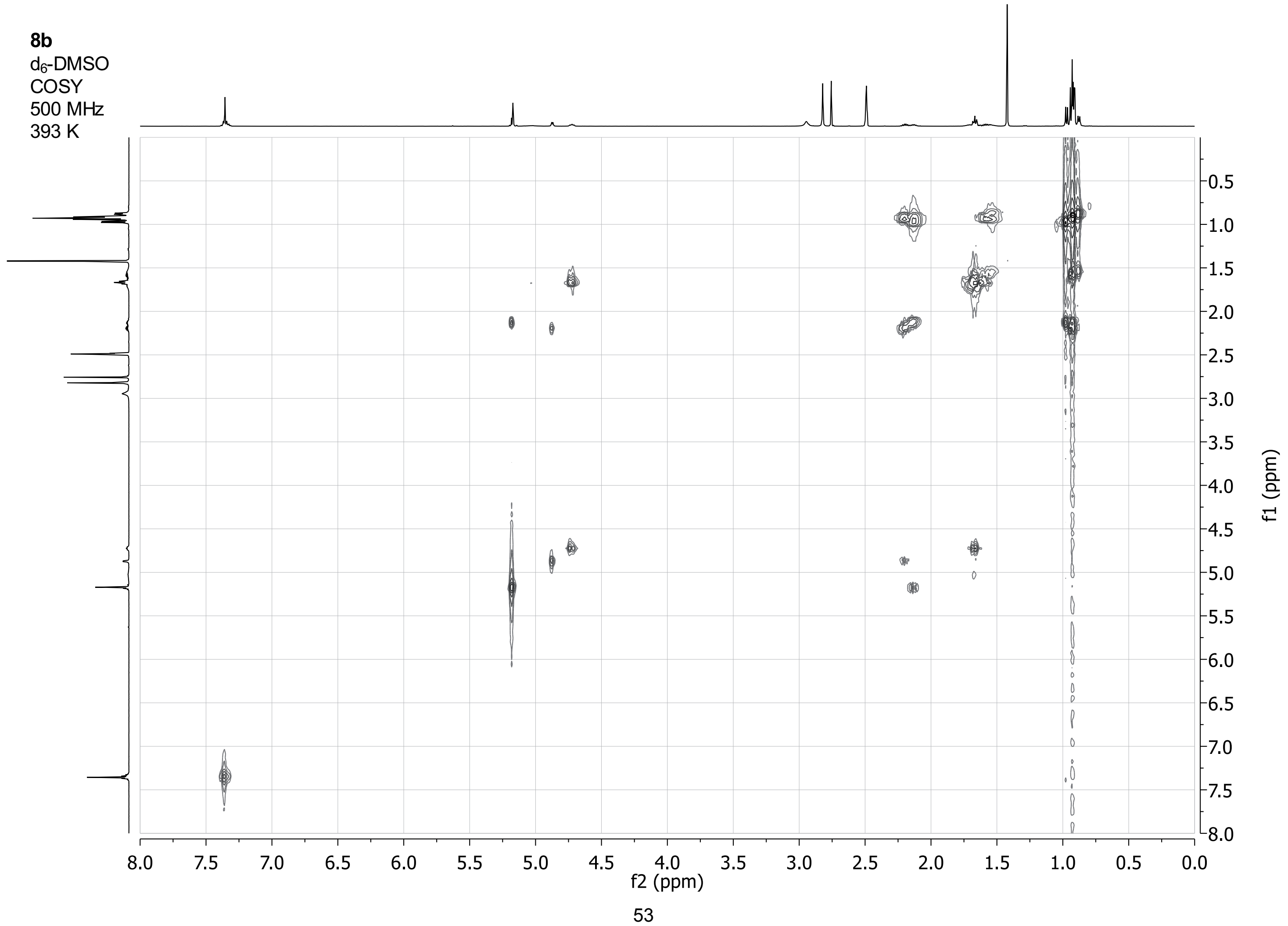
8b
d6-DMSO
COSY
500 MHz
393 K
f2 (ppm)
f1 (ppm)

8b
d$_6$-DMSO
HSQC
500/126 MHz
393 K

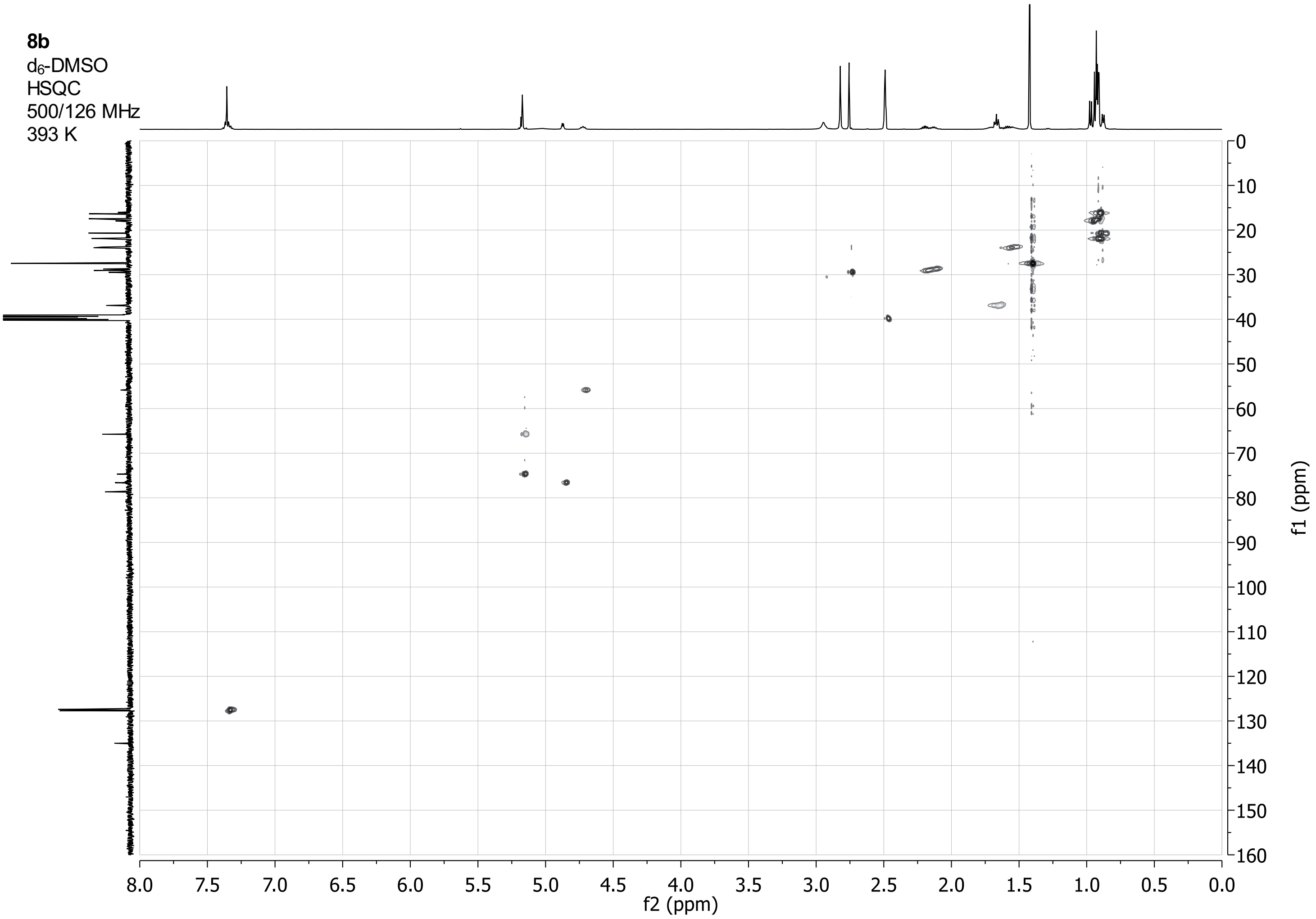

8b
d$_6$-DMSO
HMBC
500/126 MHz
393 K

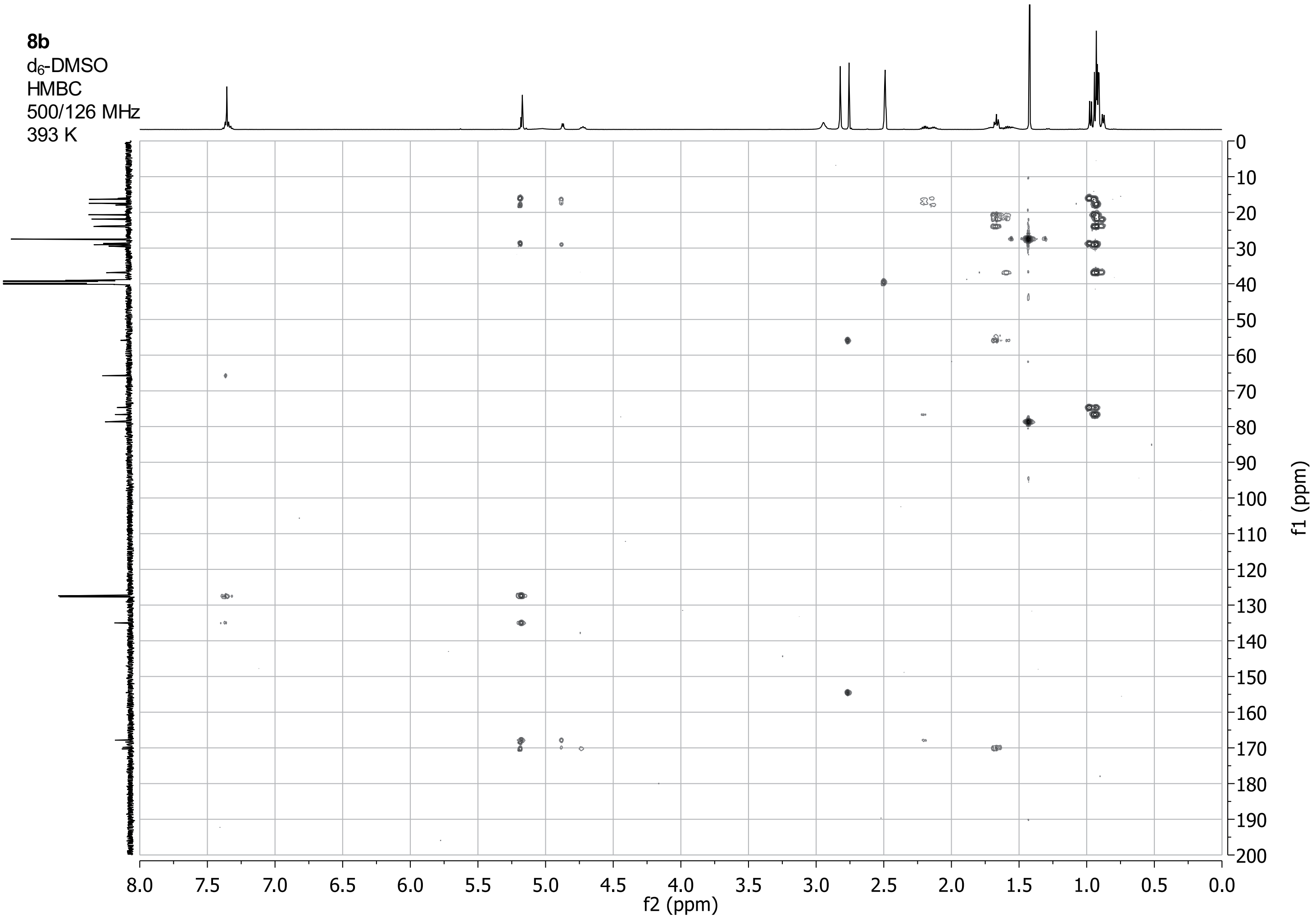

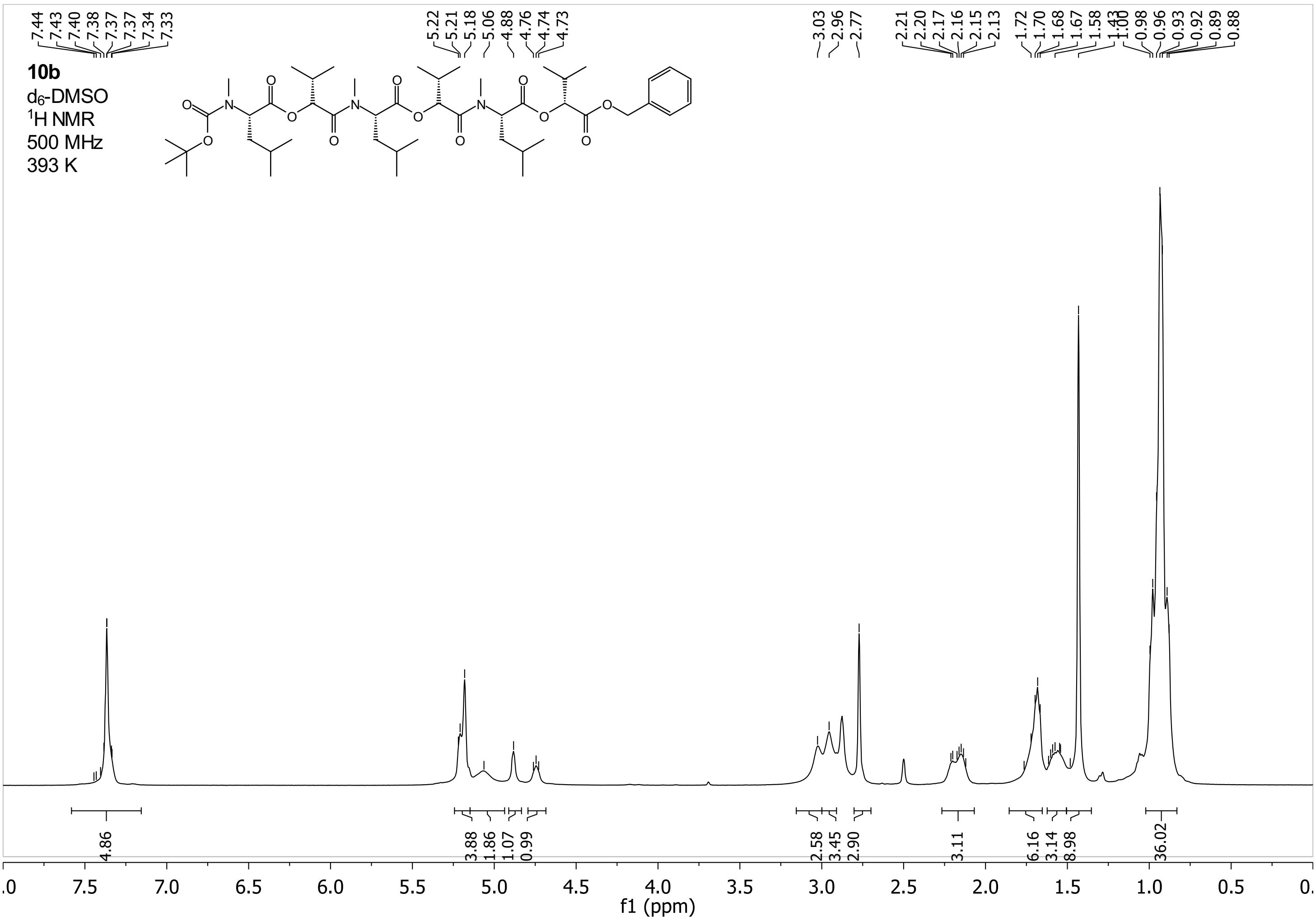
10b
d6-DMSO
1H NMR
500 MHz
393 K
7.44 7.43 7.40 7.38 7.37 7.37 7.34 7.33
5.22 5.21 5.18 5.06 4.88 4.76 4.74 4.73
3.03 2.96 2.77
2.21 2.20 2.17 2.16 2.15 2.13
1.72 1.70 1.68 1.67 1.58 1.43 1.00 0.98 0.96 0.93 0.92 0.89 0.88
4.86
3.88 1.86 1.07 0.99
2.58 3.45 2.90
3.11
6.16 3.14 8.98
36.02
f1 (ppm)

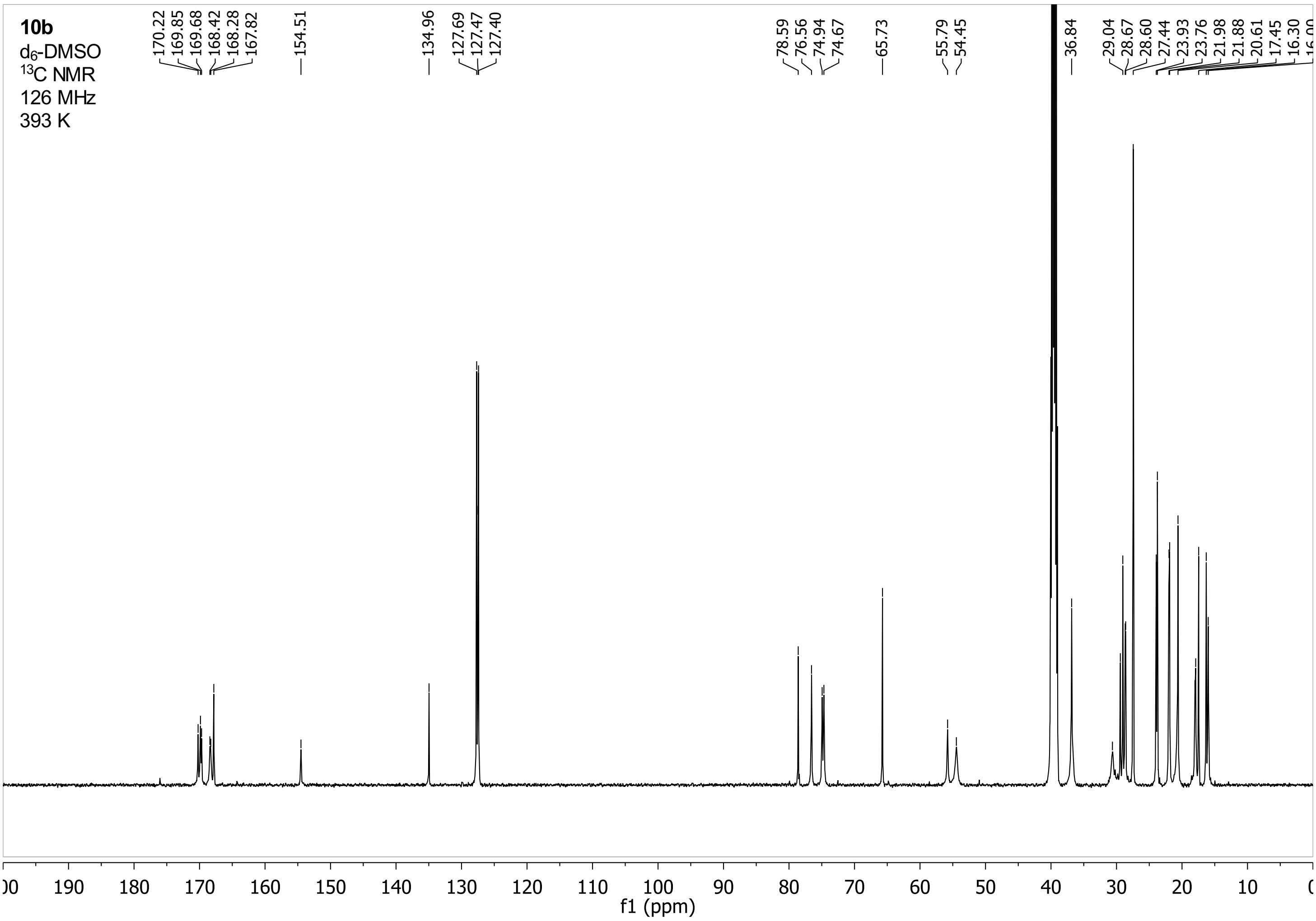
10b
d6-DMSO
13C NMR
126 MHz
393 K
170.22 169.85 169.68 168.42 168.28 167.82
154.51
134.96 127.69 127.47 127.40
78.59 76.56 74.94 74.67
65.73
55.79 54.45
36.84
29.04 28.67 28.60 27.44 23.93 23.76 21.98 21.88 20.61 17.45 16.30
f1 (ppm)

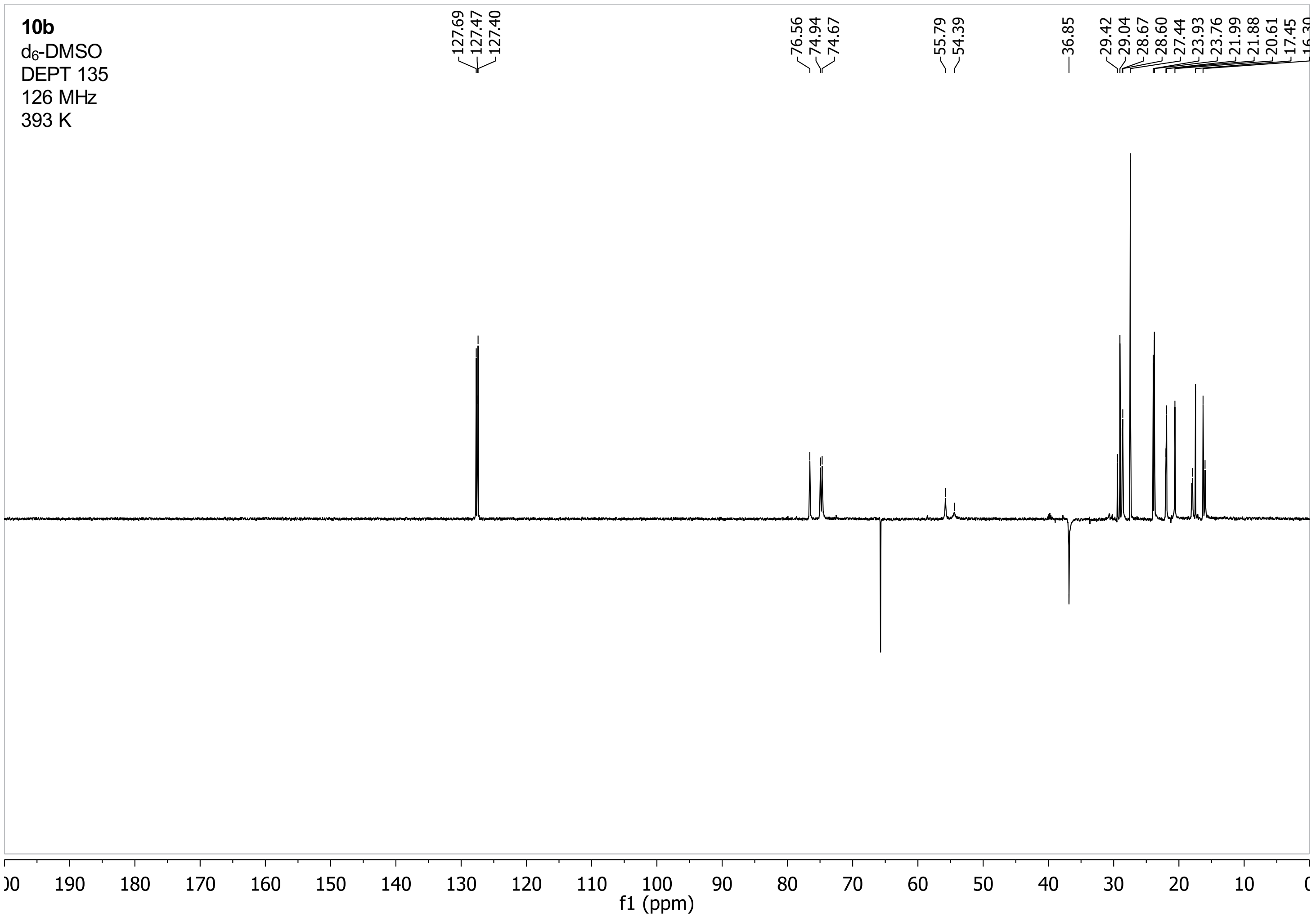
10b
d6-DMSO
DEPT 135
126 MHz
393 K
127.69
127.47
127.40
76.56
74.94
74.67
55.79
54.39
36.85
29.42
29.04
28.67
28.60
27.44
23.93
23.76
21.99
21.88
20.61
17.45
f1 (ppm)

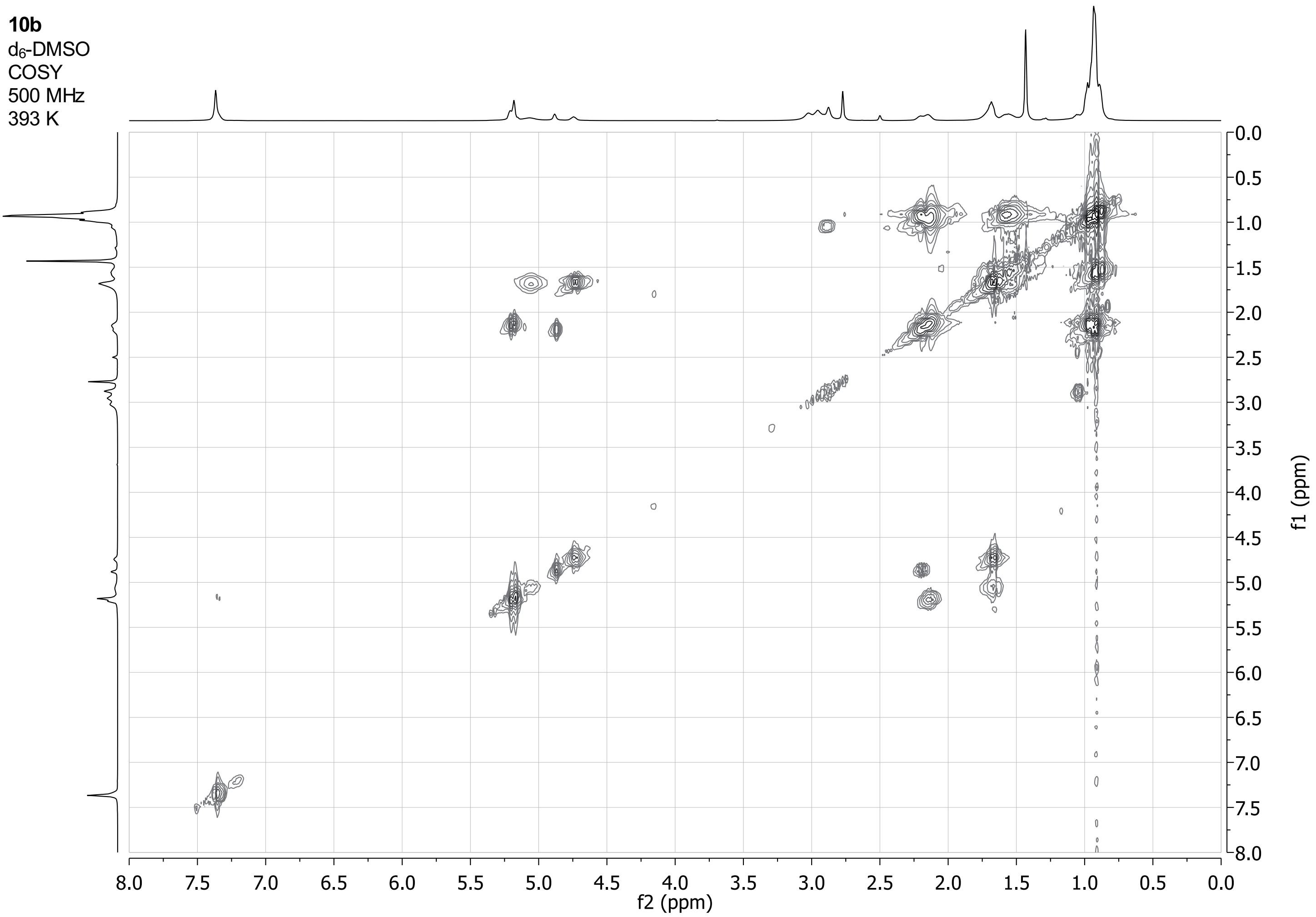
10b
d6-DMSO
COSY
500 MHz
393 K
f2 (ppm)
f1 (ppm)

10b
d_6-DMSO
HSQC
500/126 MHz
393 K

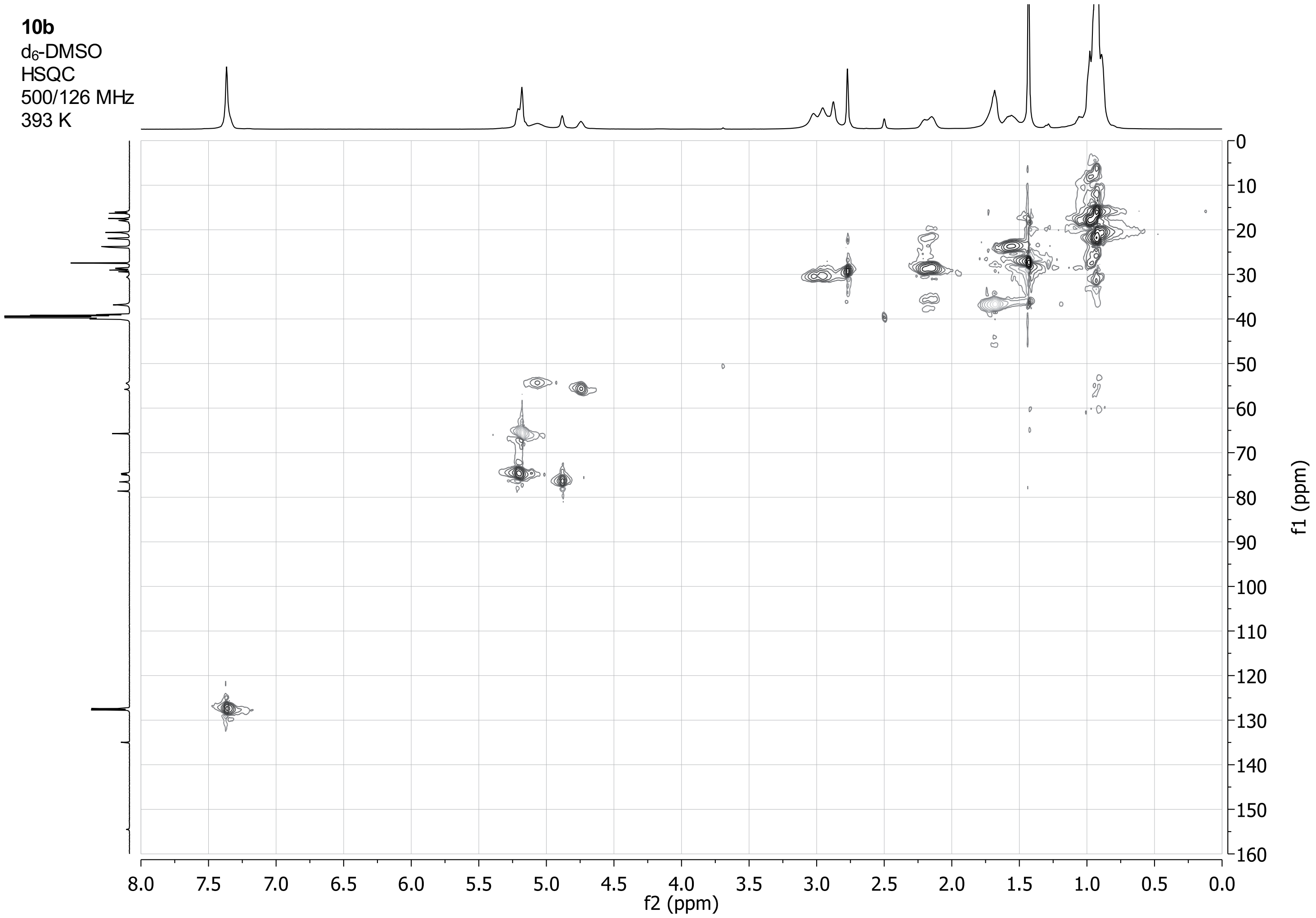

10b
d_6-DMSO
HMBC
500/126 MHz
393 K

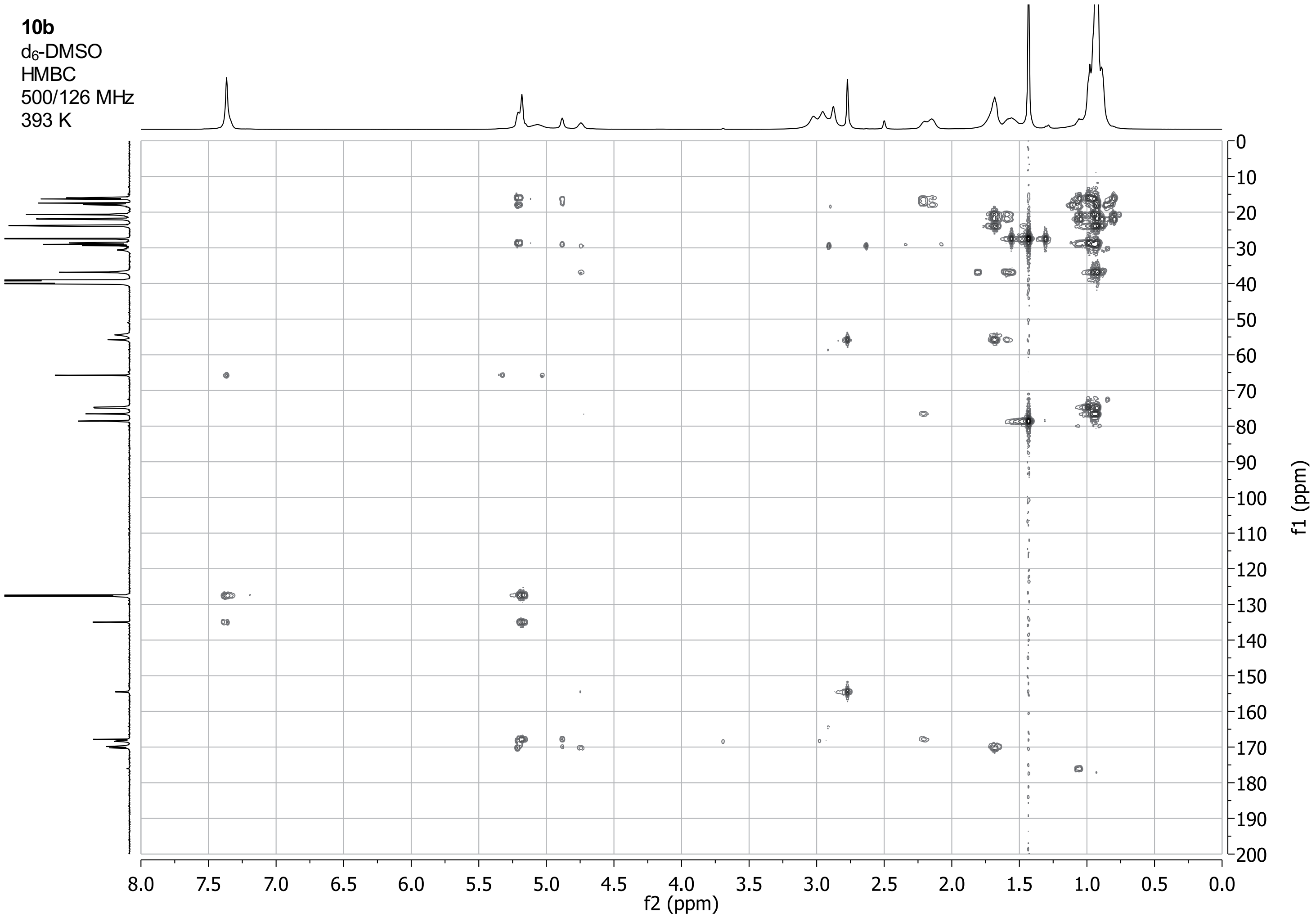

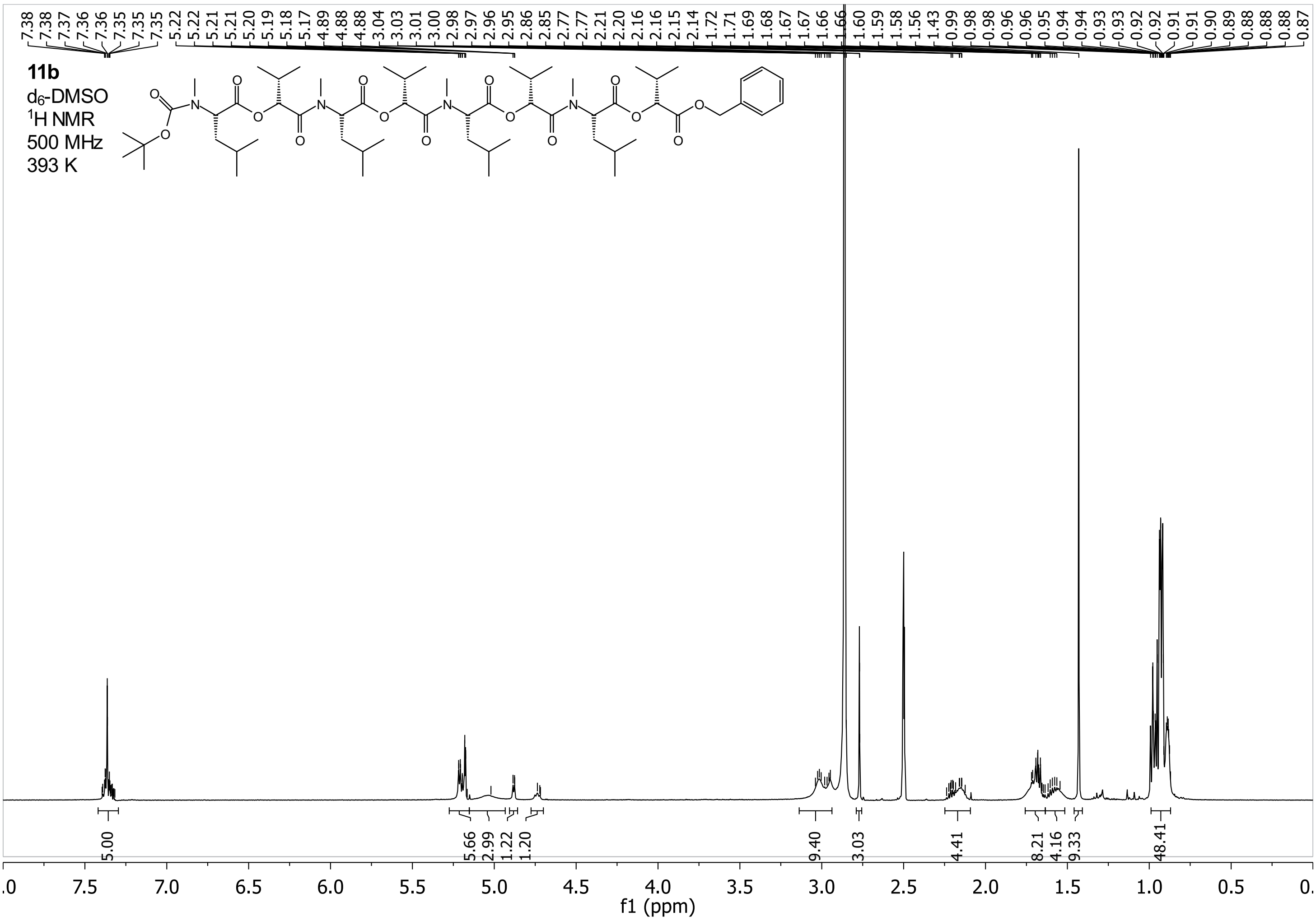

11b
d6-DMSO
1H NMR
500 MHz
393 K
7.38 7.38 7.37 7.36 7.36 7.35 7.35 7.35 5.22 5.22 5.21 5.20 5.19 5.18 5.17 4.89 4.88 4.88 3.04 3.03 3.01 3.00 2.98 2.97 2.96 2.95 2.86 2.85 2.77 2.77 2.21 2.20 2.16 2.16 2.15 2.14 1.72 1.71 1.69 1.68 1.67 1.67 1.66 1.66 1.60 1.59 1.58 1.56 1.43 0.99 0.98 0.98 0.96 0.96 0.95 0.94 0.94 0.93 0.93 0.92 0.92 0.91 0.91 0.90 0.89 0.88 0.88 0.88 0.87
5.00 5.66 2.99 1.22 1.20 9.40 3.03 4.41 8.21 4.16 9.33 48.41
f1 (ppm)

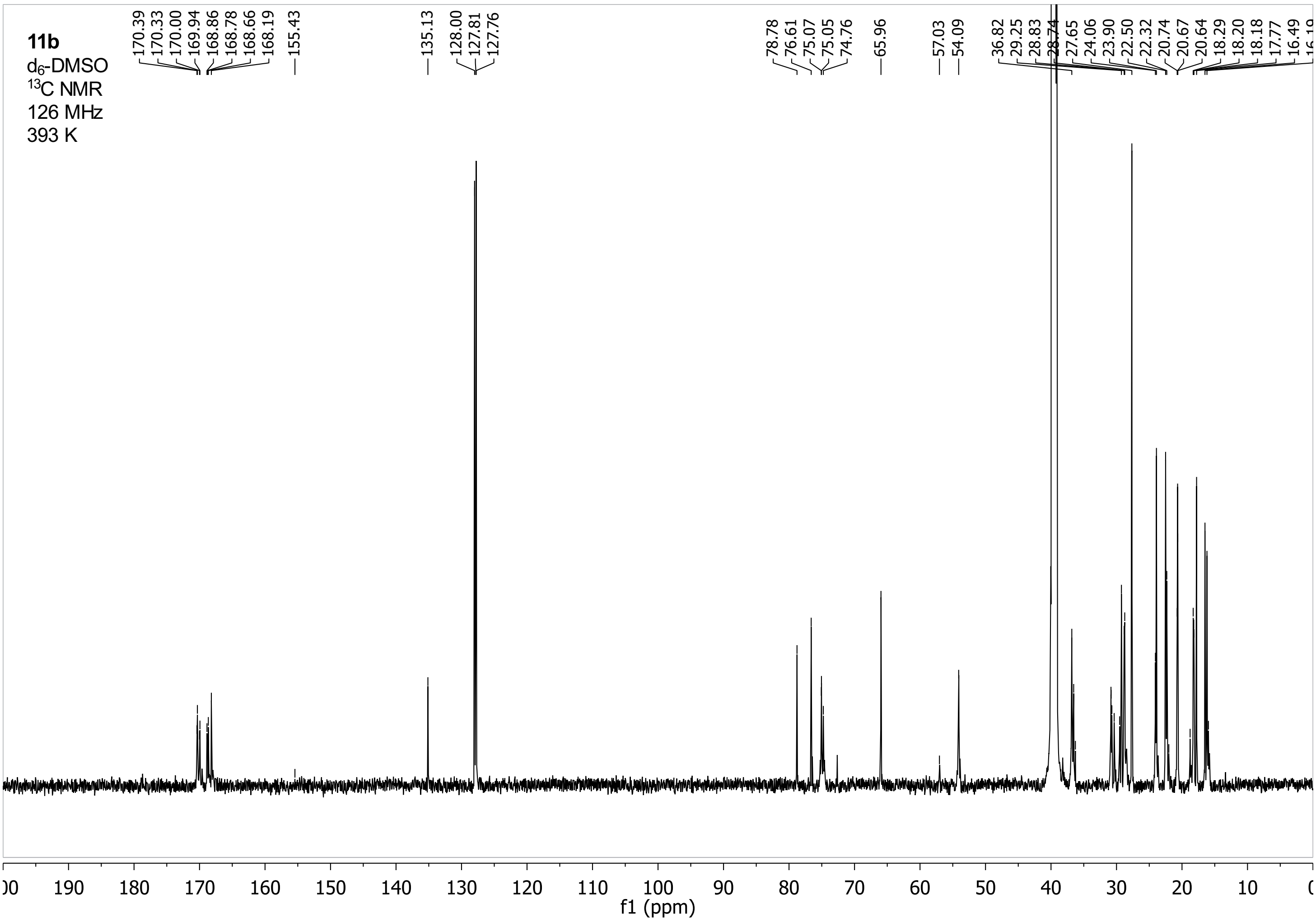

11b
d6-DMSO
13C NMR
126 MHz
393 K
170.39 170.33 170.00 169.94 168.86 168.78 168.66 168.19 155.43 135.13 128.00 127.81 127.76 78.78 76.61 75.07 75.05 74.76 65.96 57.03 54.09 36.82 29.25 28.83 28.74 27.65 24.06 23.90 22.50 22.32 20.74 20.67 20.64 18.29 18.20 18.18 17.77 16.49
f1 (ppm)

11b
d_6-DMSO
COSY
500 MHz
393 K

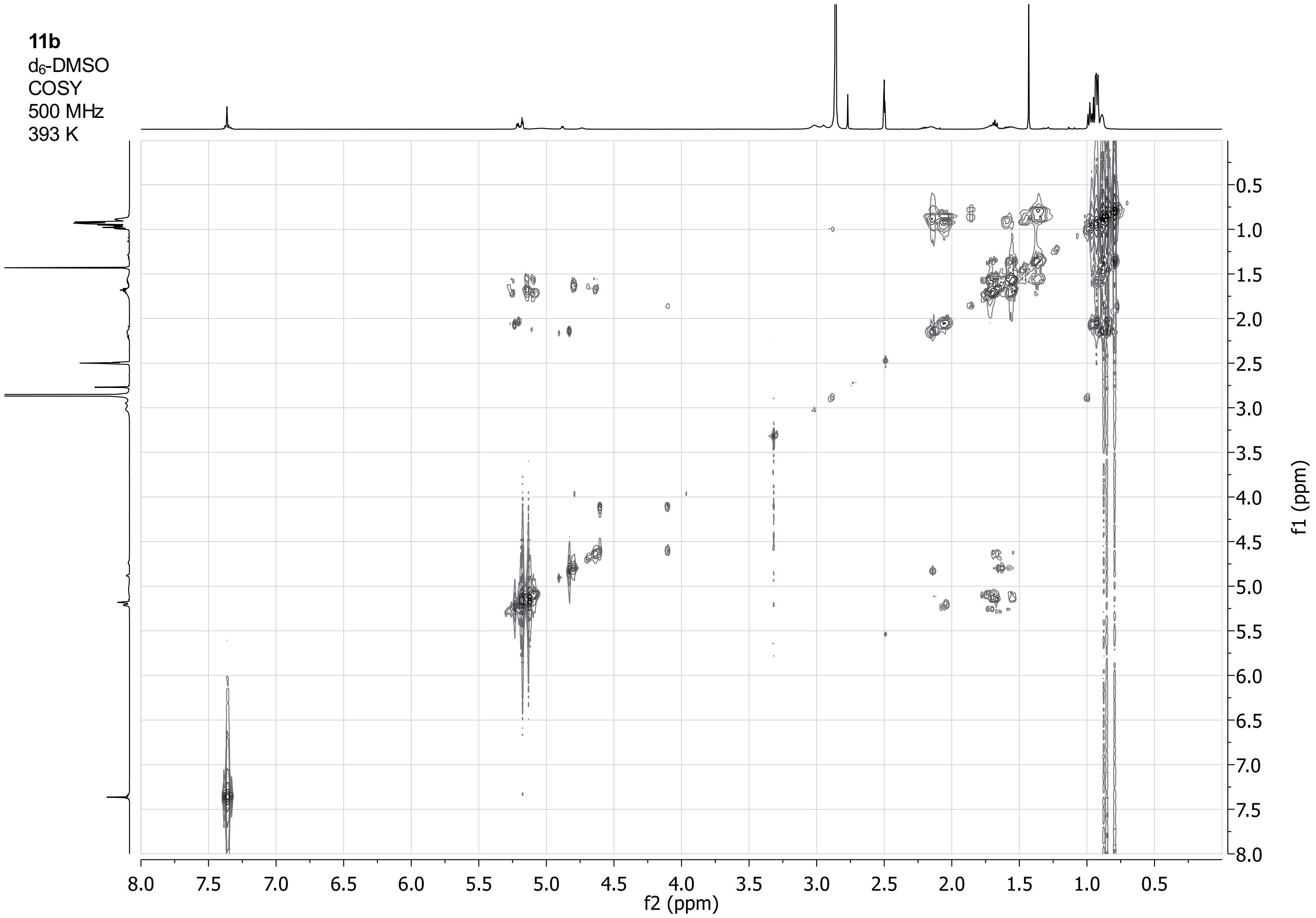

11b
d_6-DMSO
HSQC
500/126 MHz
393 K

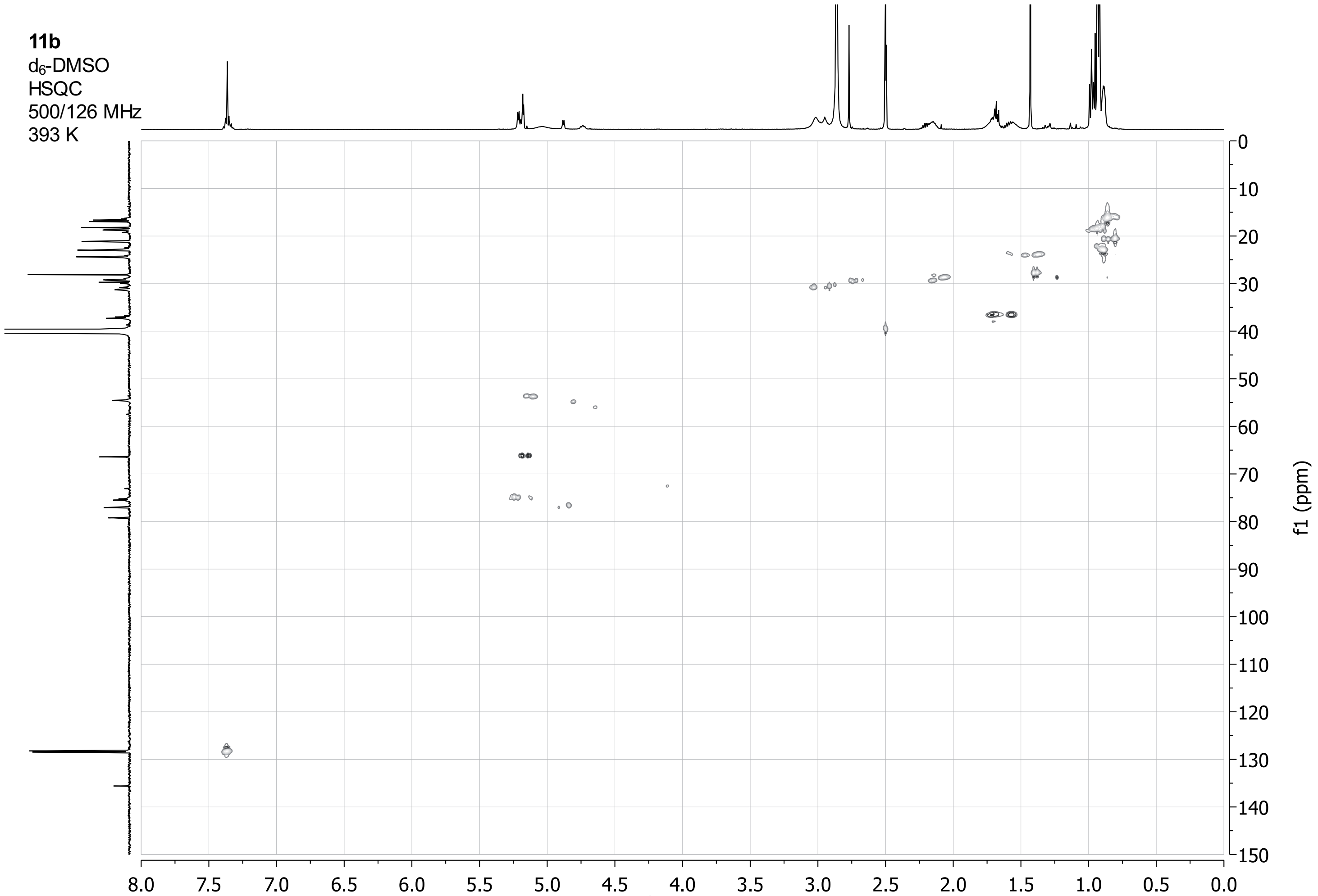

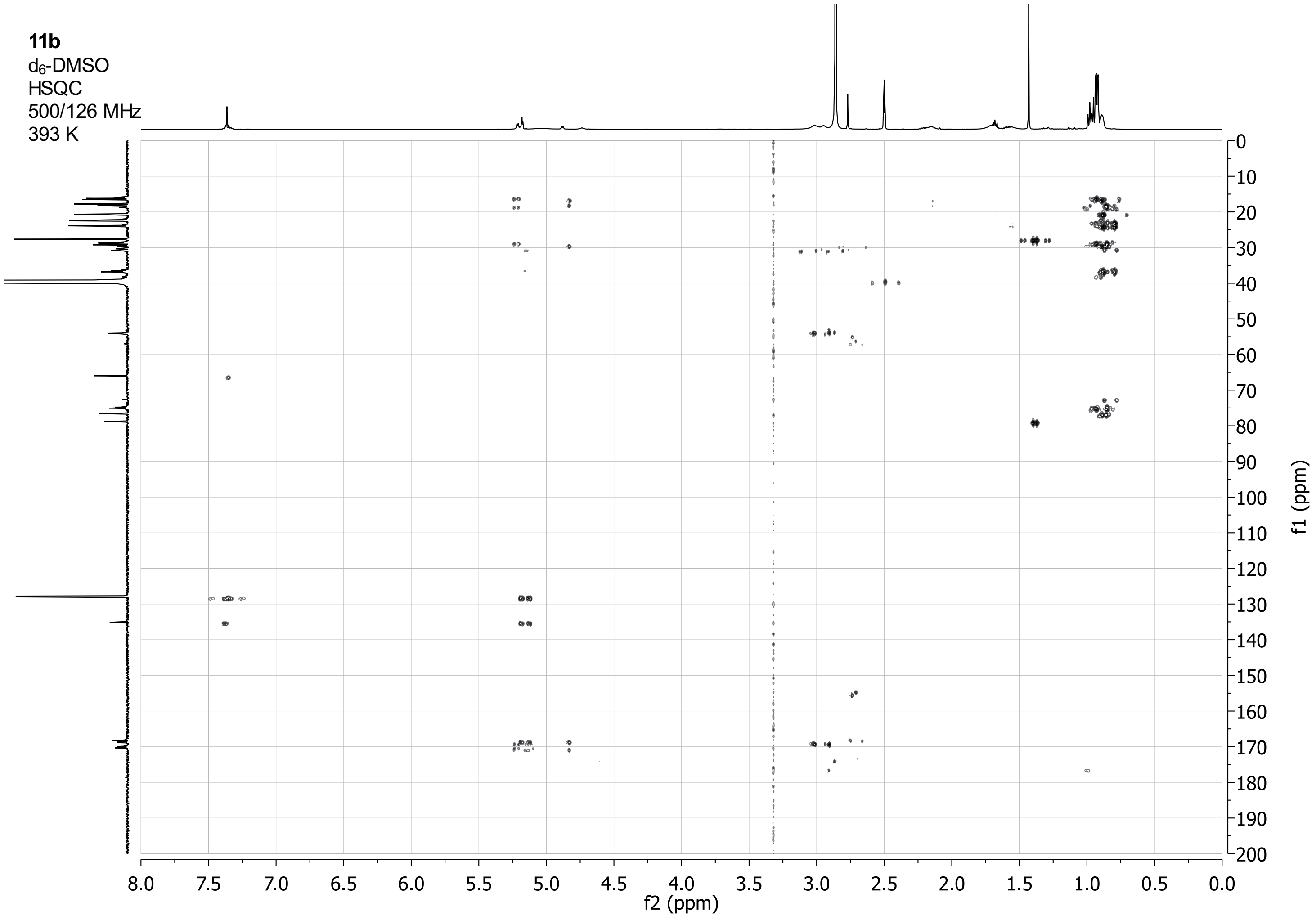
11b
d6-DMSO
HSQC
500/126 MHz
393 K
f1 (ppm)
f2 (ppm)

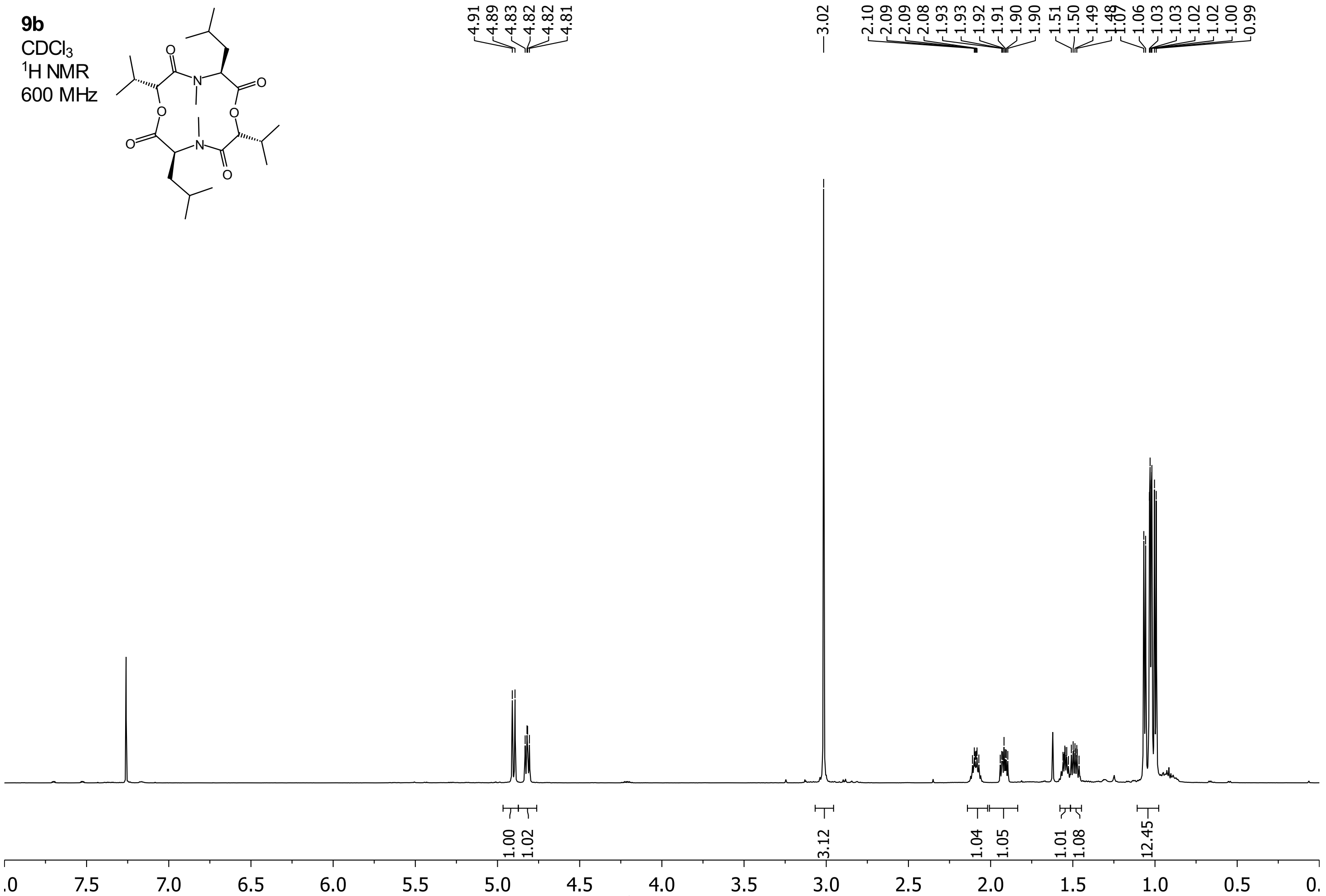
9b
CDCl3
1H NMR
600 MHz
f1 (ppm)

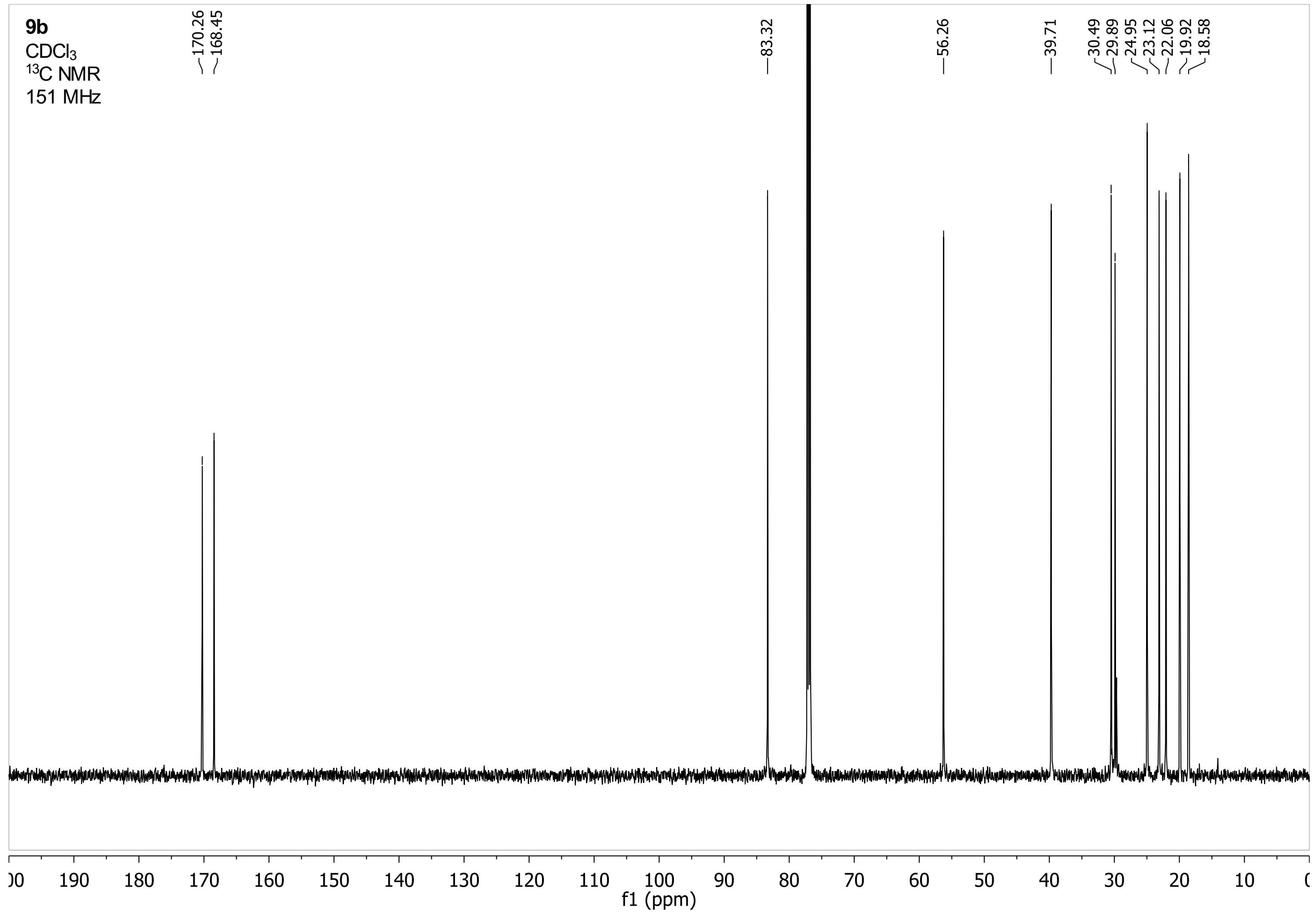

9b
$CDCl_3$
^{13}C NMR
151 MHz
170.26
168.45
83.32
56.26
39.71
30.49
29.89
24.95
23.12
22.06
19.92
18.58
f1 (ppm)

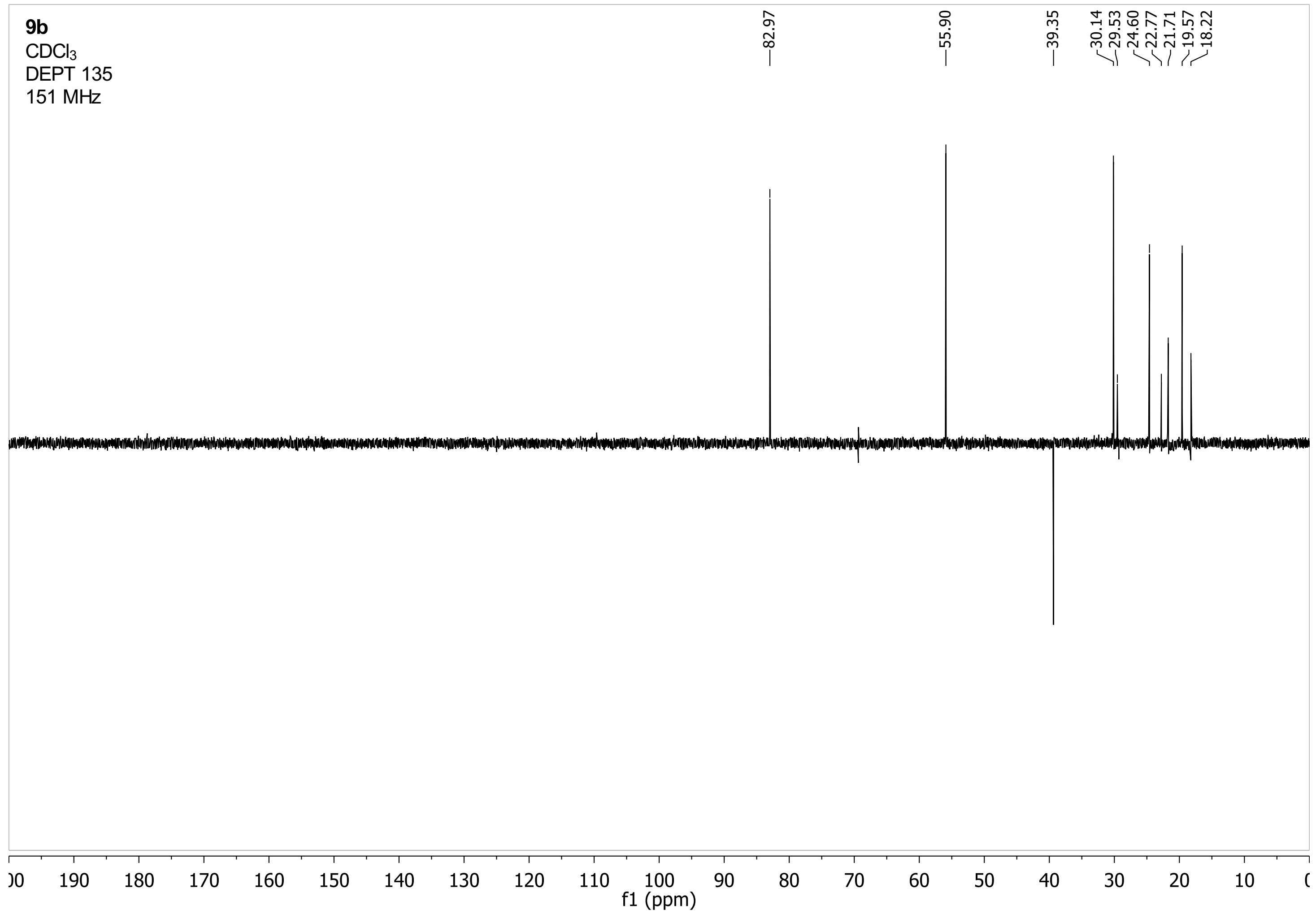

9b
$CDCl_3$
DEPT 135
151 MHz
82.97
55.90
39.35
30.14
29.53
24.60
22.77
21.71
19.57
18.22
f1 (ppm)

9b
$CDCl_3$
COSY
600 MHz

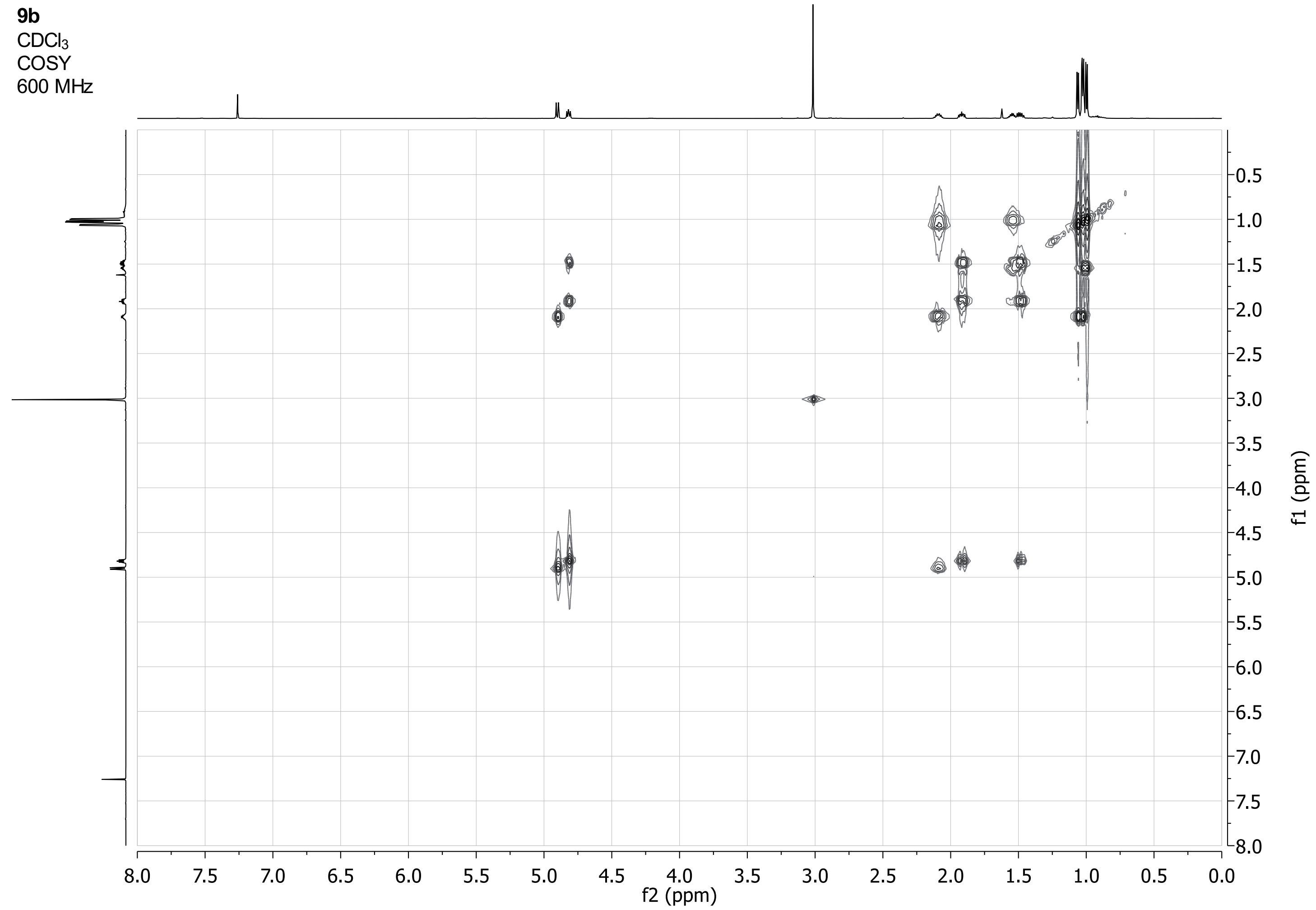

9b
$CDCl_3$
HSQC
600/151 MHz

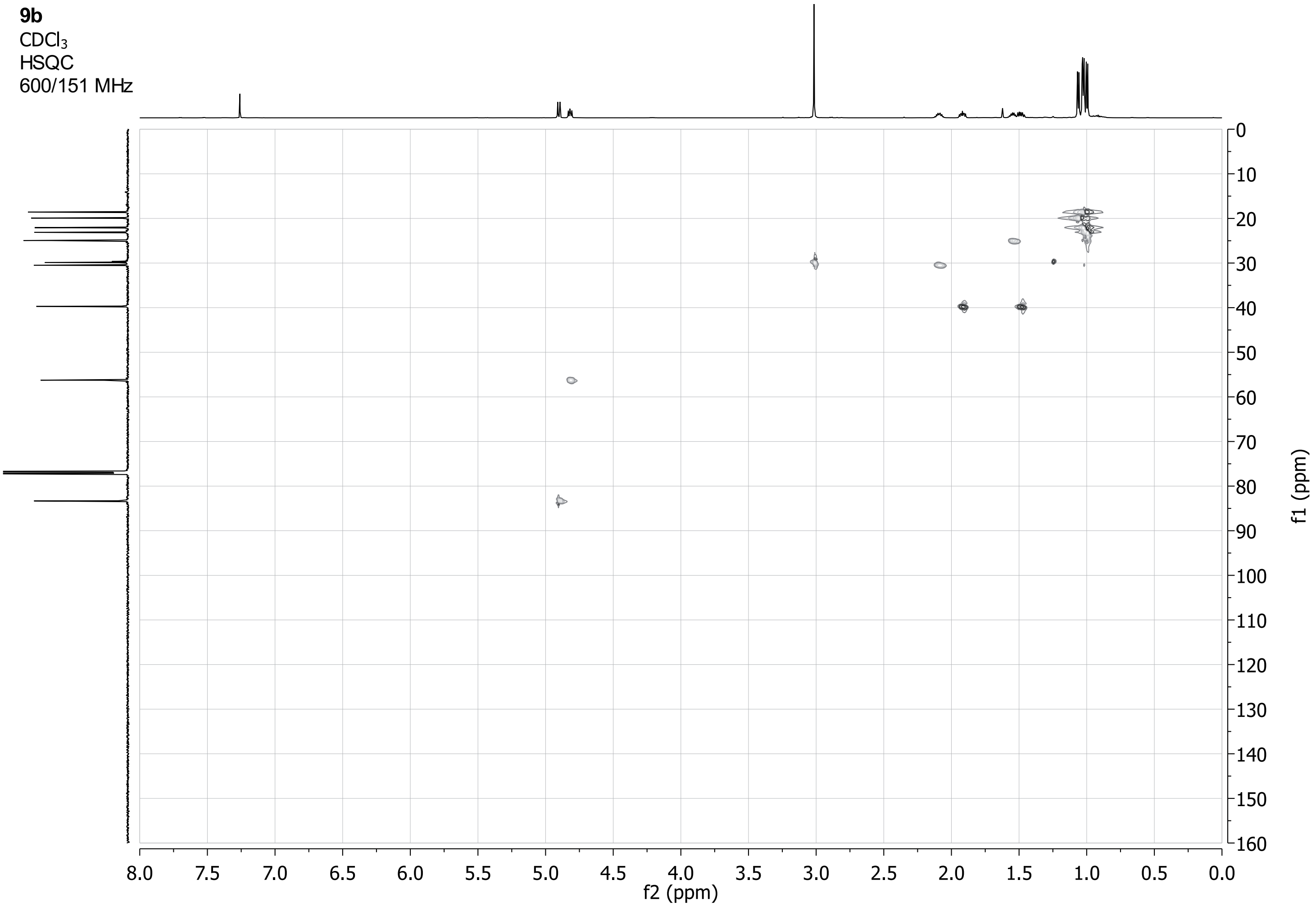

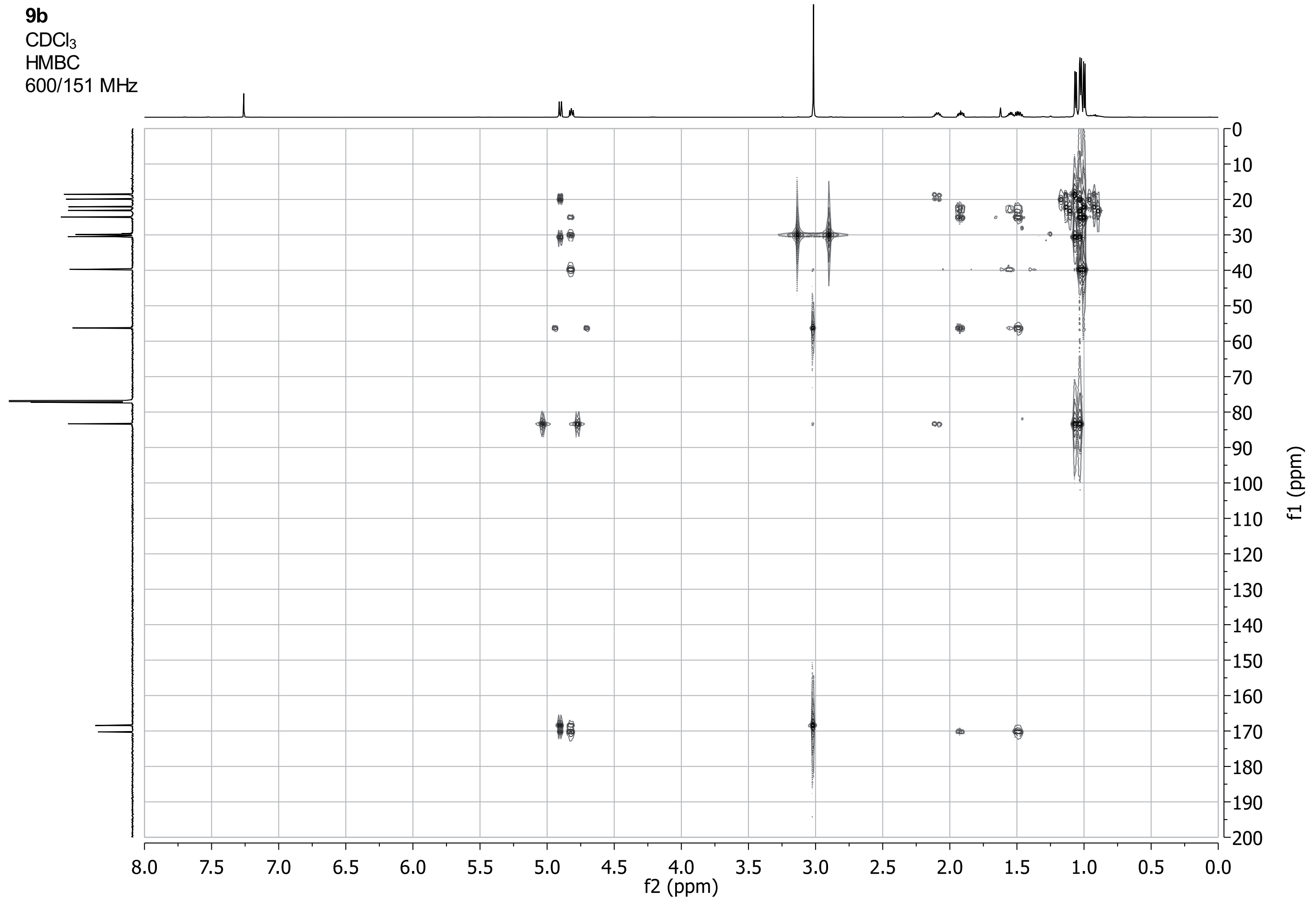
9b
CDCl3
HMBC
600/151 MHz
f2 (ppm)
f1 (ppm)

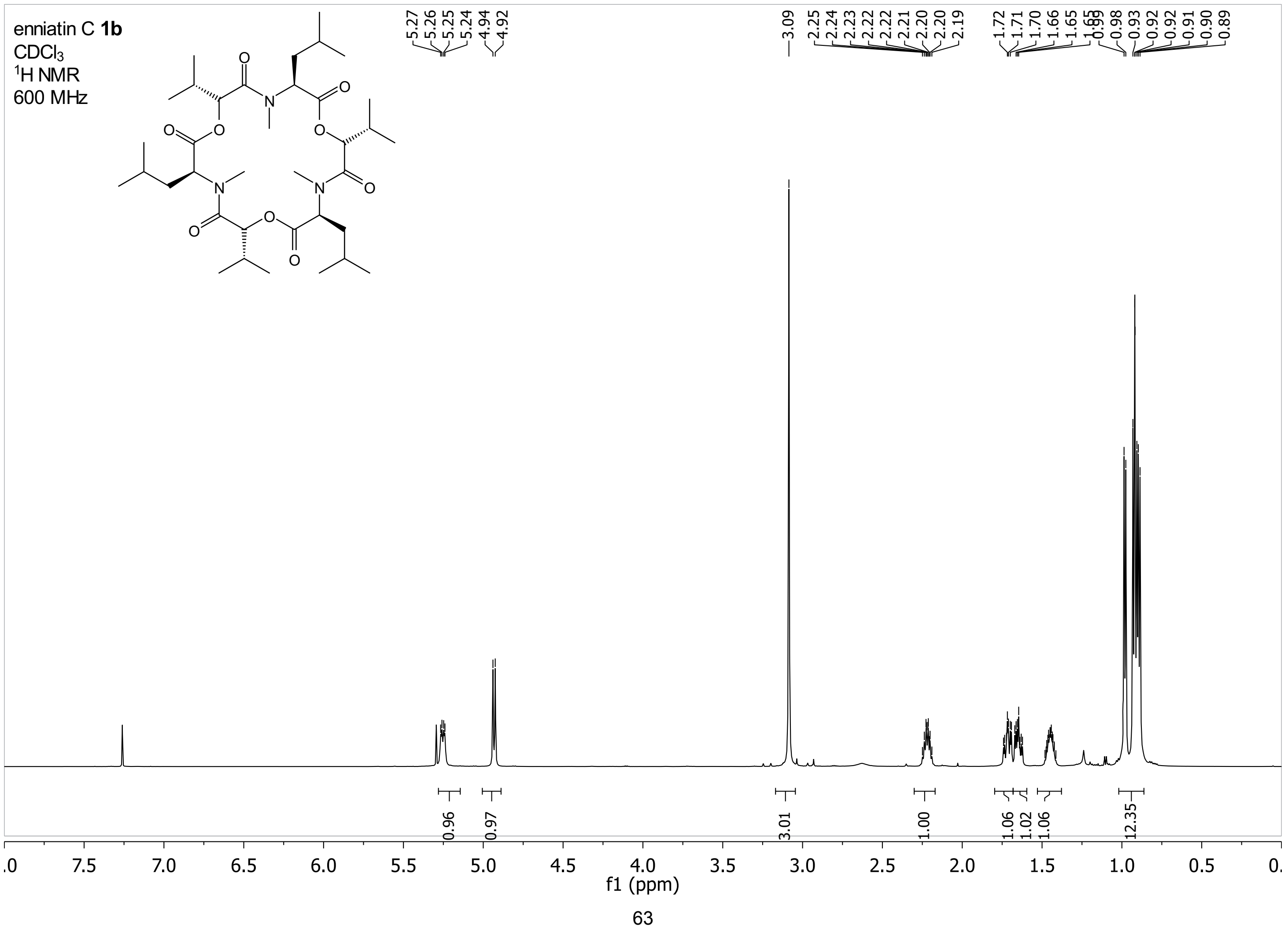
enniatin C 1b
CDCl3
1H NMR
600 MHz
f1 (ppm)

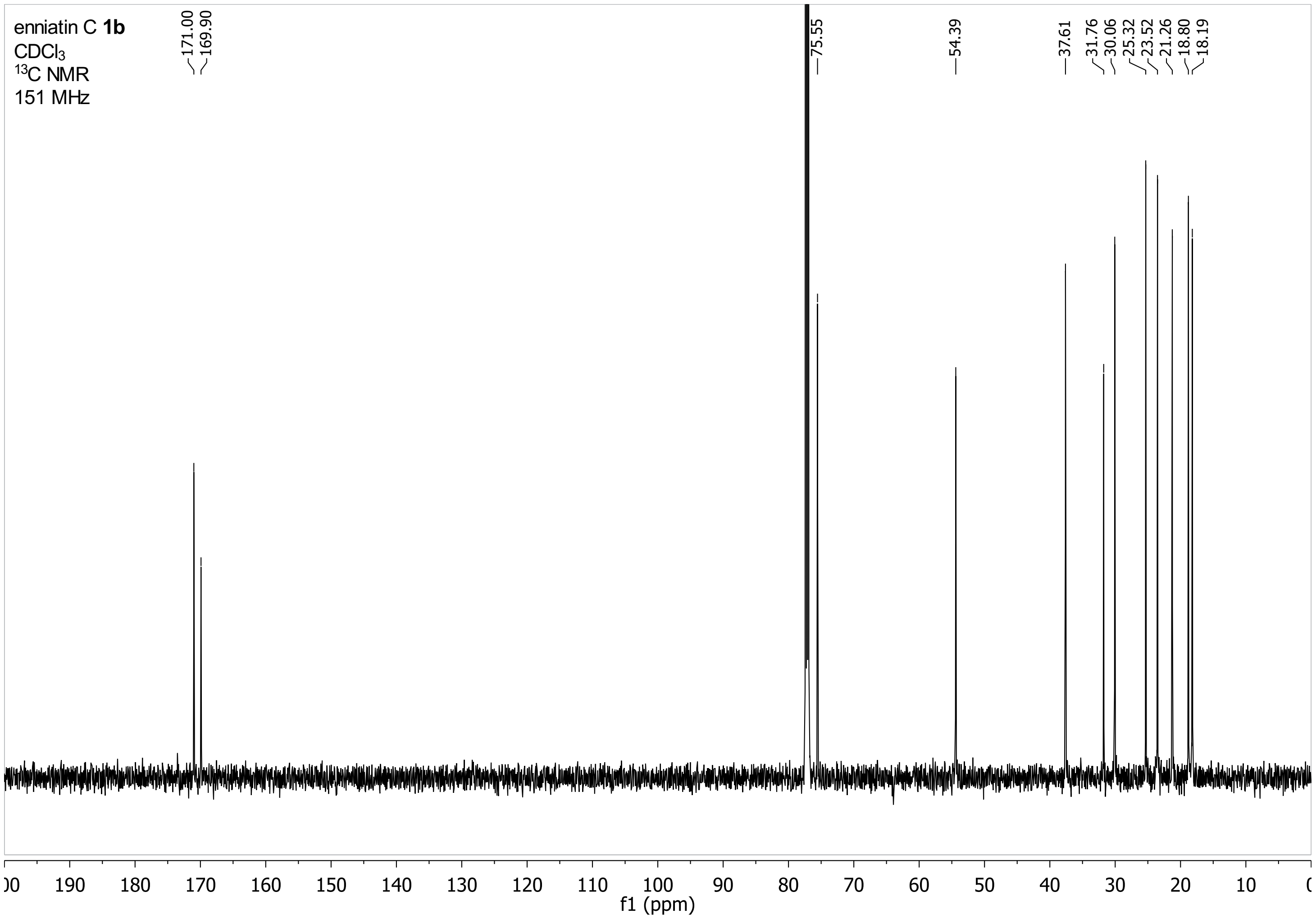
enniatin C **1b**
$CDCl_3$
^{13}C NMR
151 MHz
171.00
169.90
75.55
54.39
37.61
31.76
30.06
25.32
23.52
21.26
18.80
18.19
f1 (ppm)

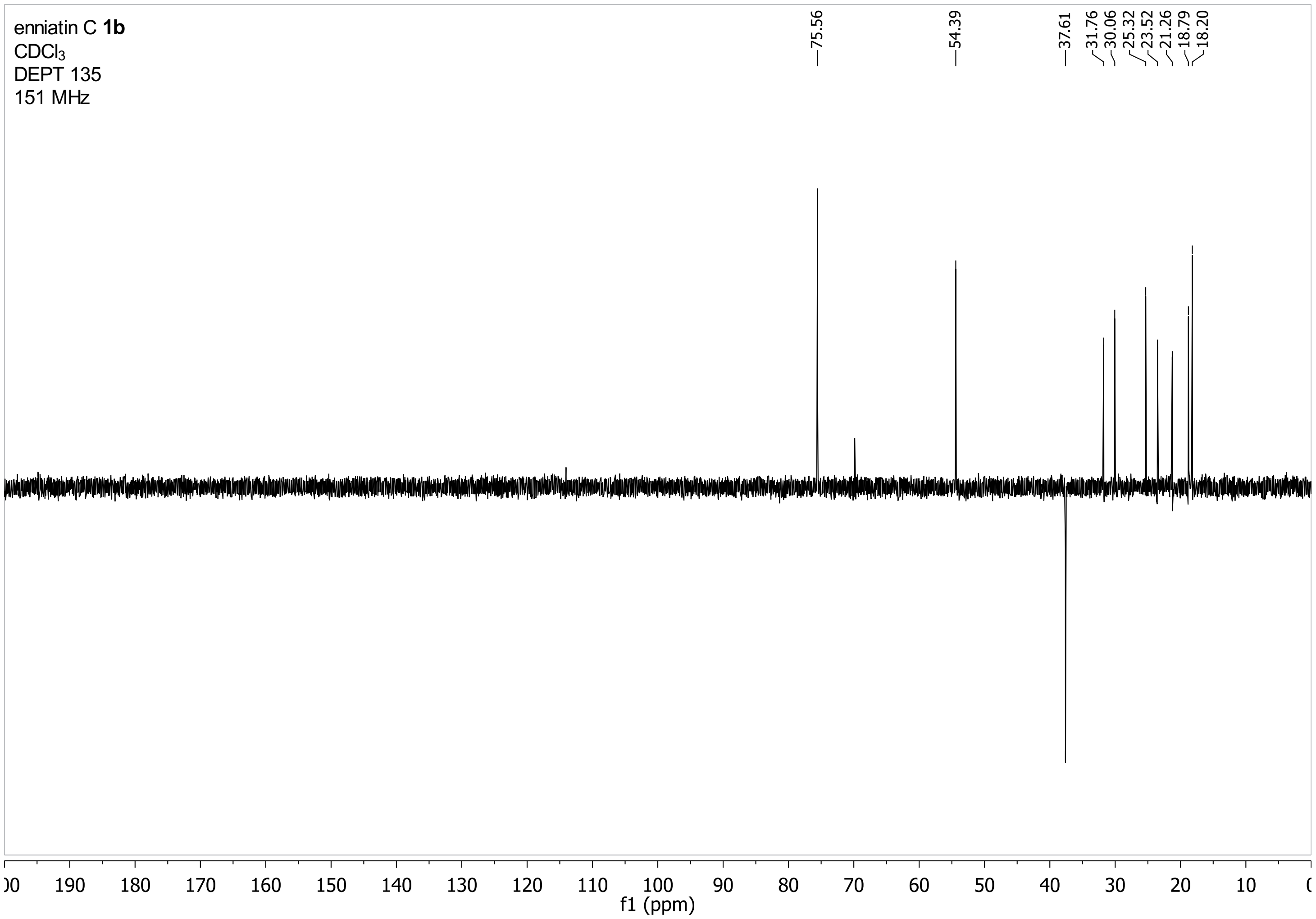
enniatin C **1b**
$CDCl_3$
DEPT 135
151 MHz
75.56
54.39
37.61
31.76
30.06
25.32
23.52
21.26
18.79
18.20
f1 (ppm)

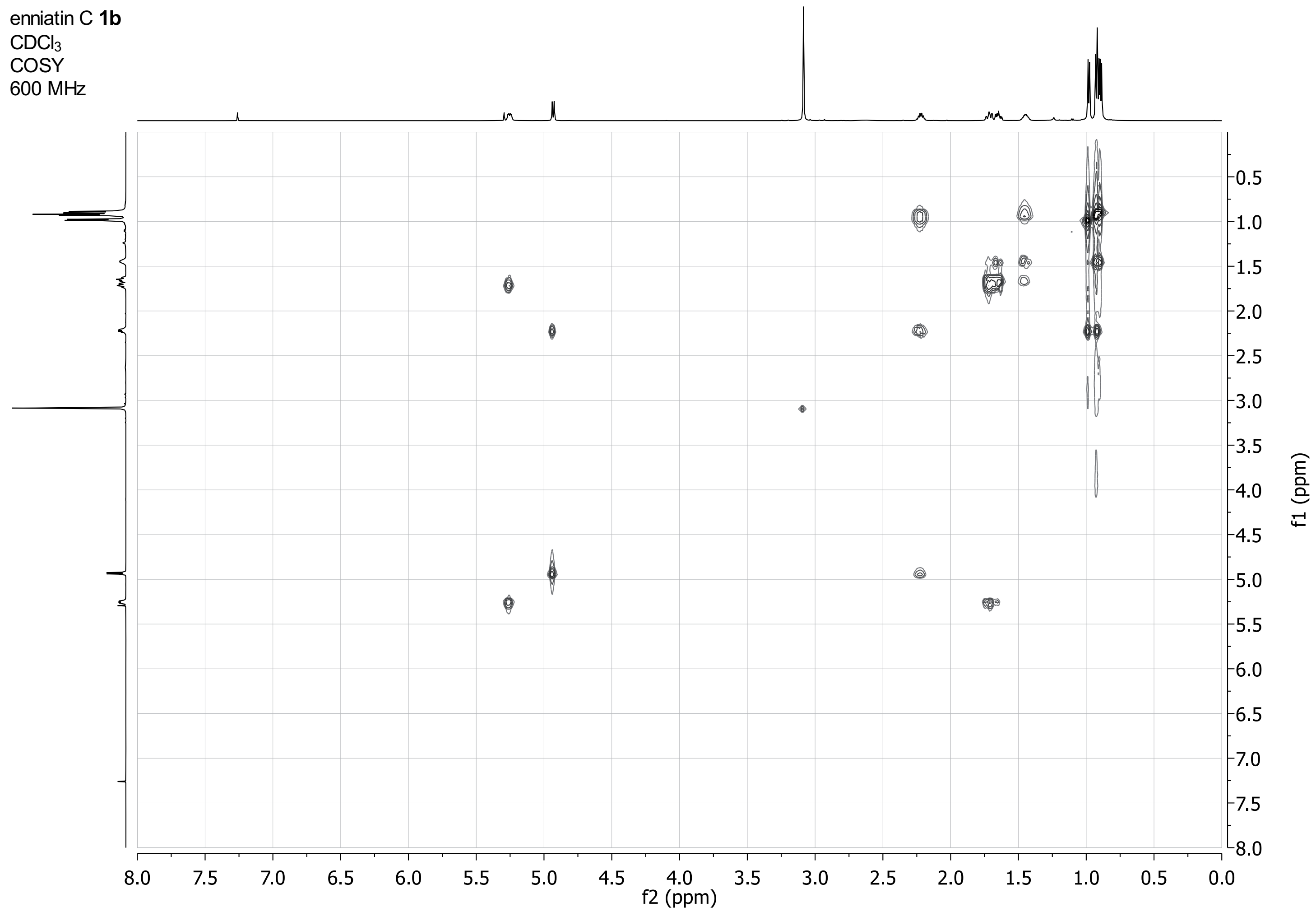
enniatin C **1b**
$CDCl_3$
COSY
600 MHz
f2 (ppm)
f1 (ppm)

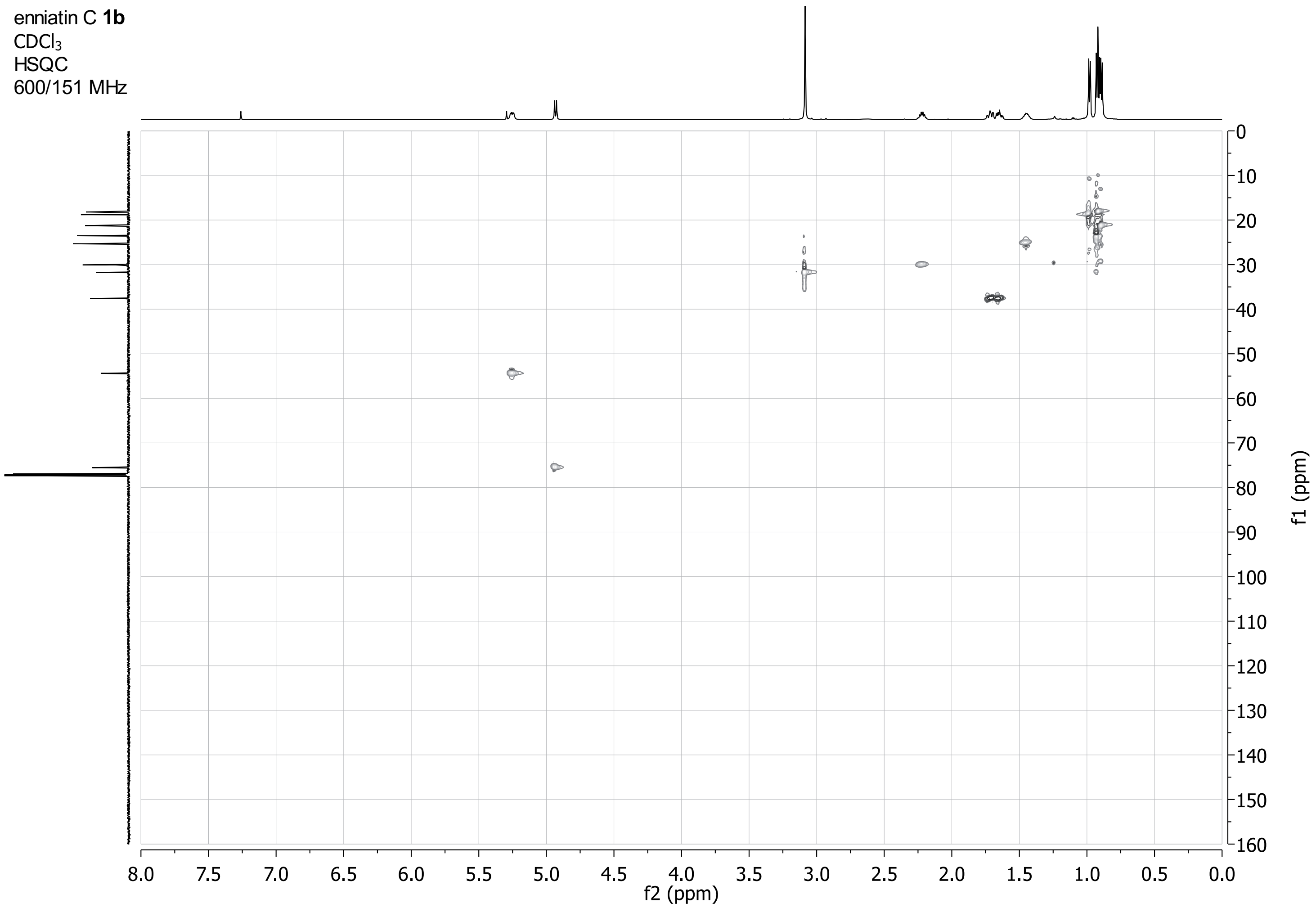
enniatin C **1b**
$CDCl_3$
HSQC
600/151 MHz
f2 (ppm)
f1 (ppm)

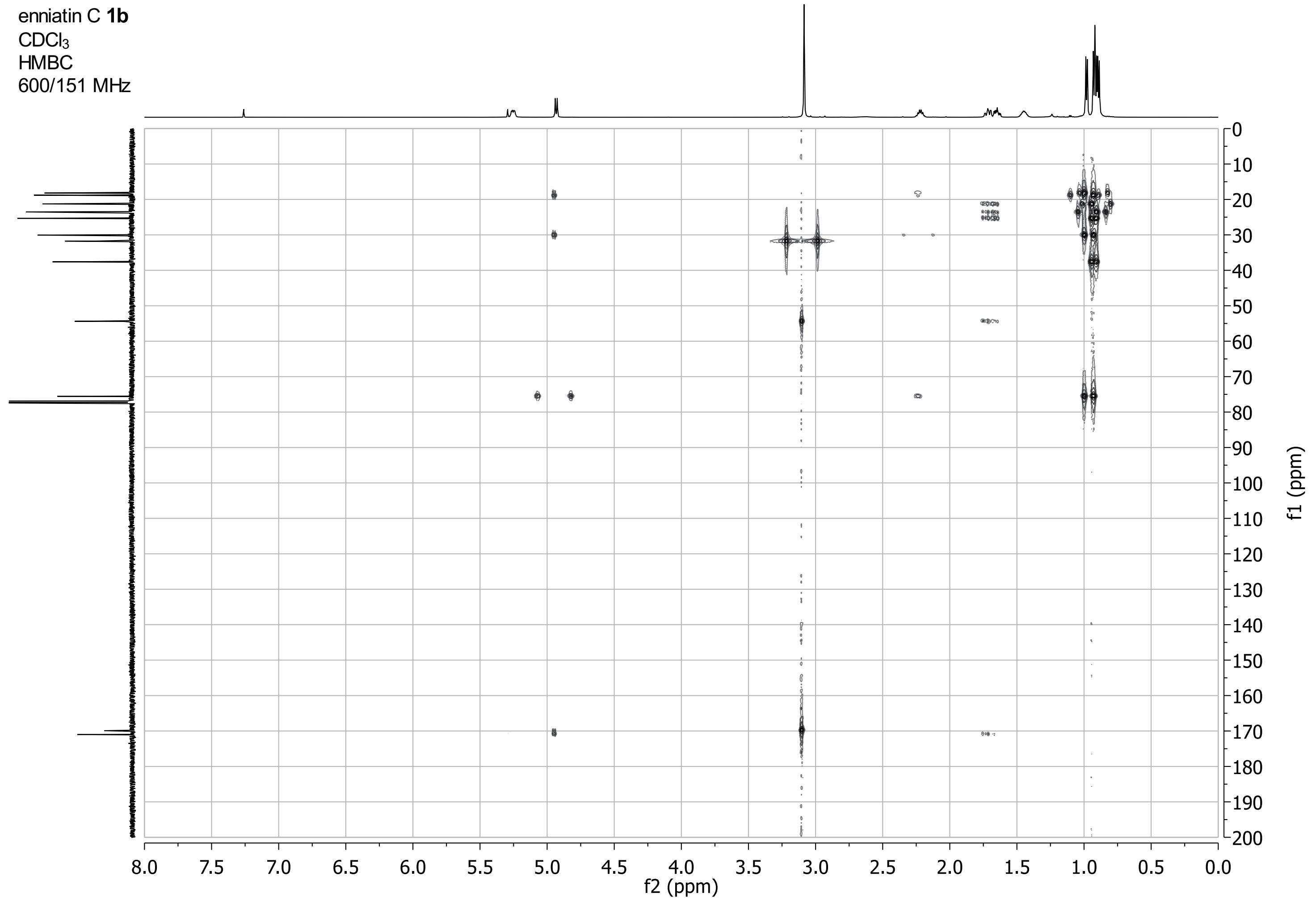
enniatin C **1b**
$CDCl_3$
HMBC
600/151 MHz
f2 (ppm)
f1 (ppm)

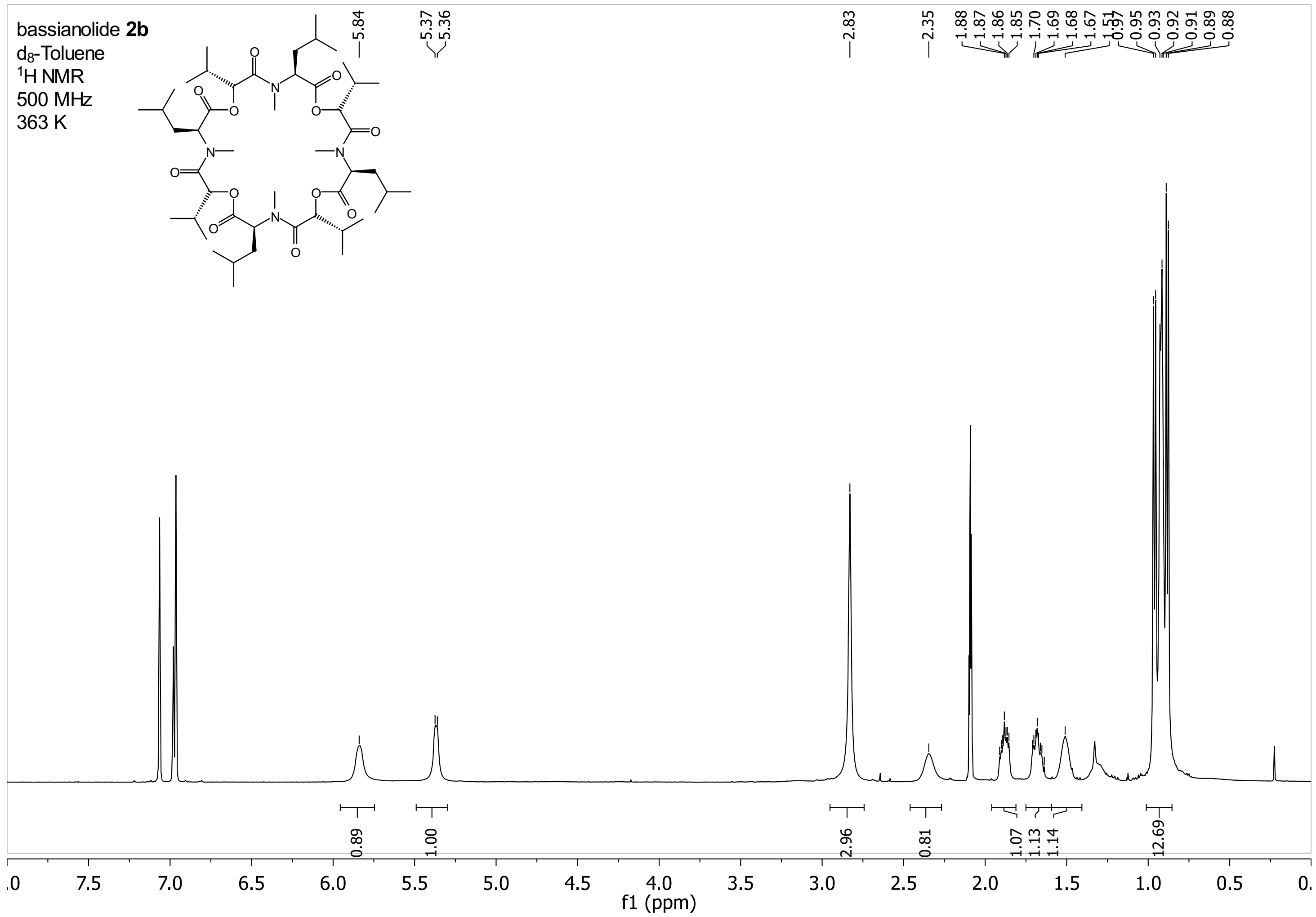
bassianolide **2b**
d_8-Toluene
^{1}H NMR
500 MHz
363 K
5.84
5.37
5.36
2.83
2.35
1.88
1.87
1.86
1.85
1.70
1.69
1.68
1.67
1.51
0.97
0.95
0.93
0.92
0.91
0.89
0.88
0.89
1.00
2.96
0.81
1.07
1.13
1.14
12.69
f1 (ppm)

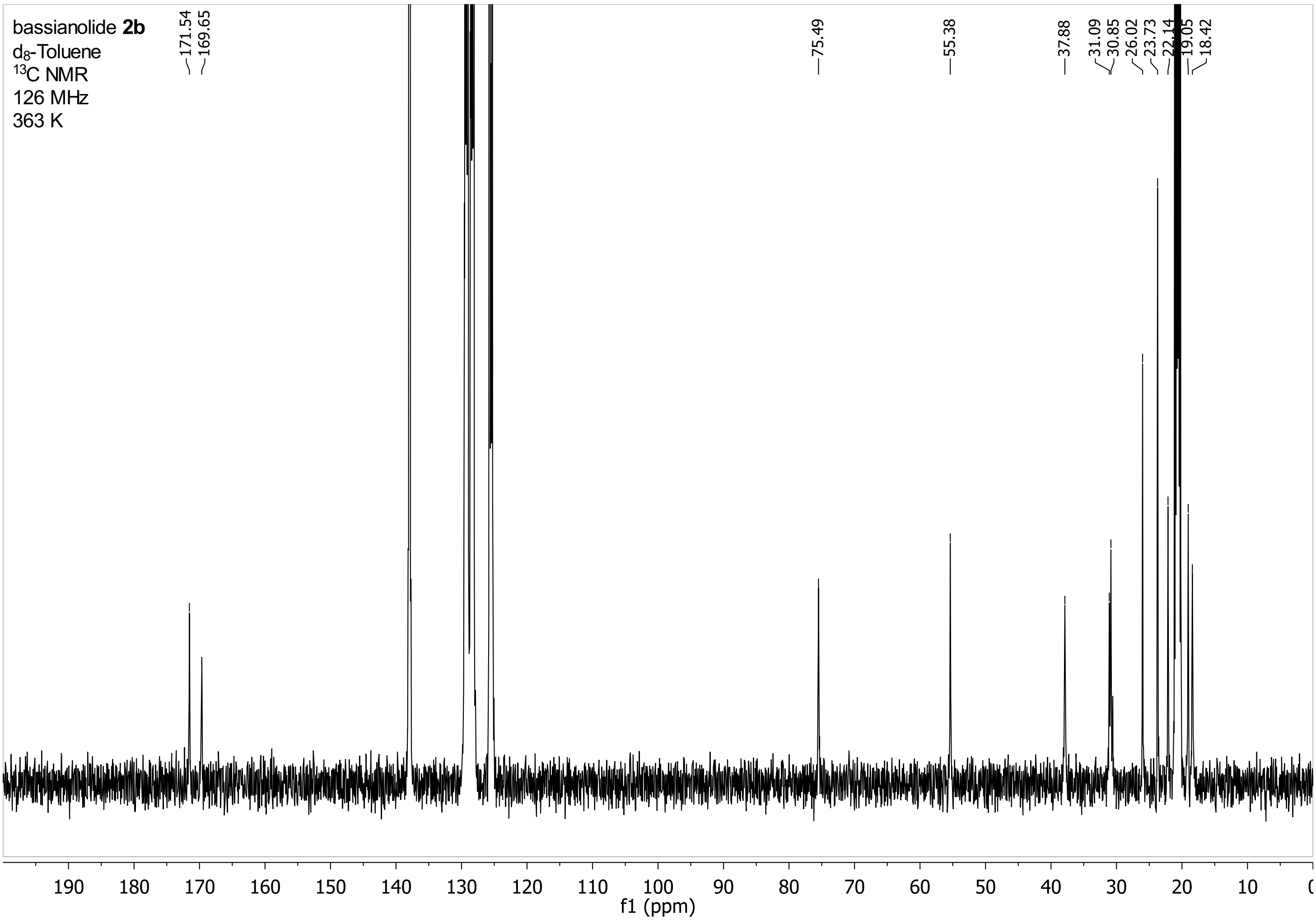
bassianolide **2b**
d_8-Toluene
^{13}C NMR
126 MHz
363 K
171.54
169.65
75.49
55.38
37.88
31.09
30.85
26.02
23.73
22.14
19.05
18.42
f1 (ppm)

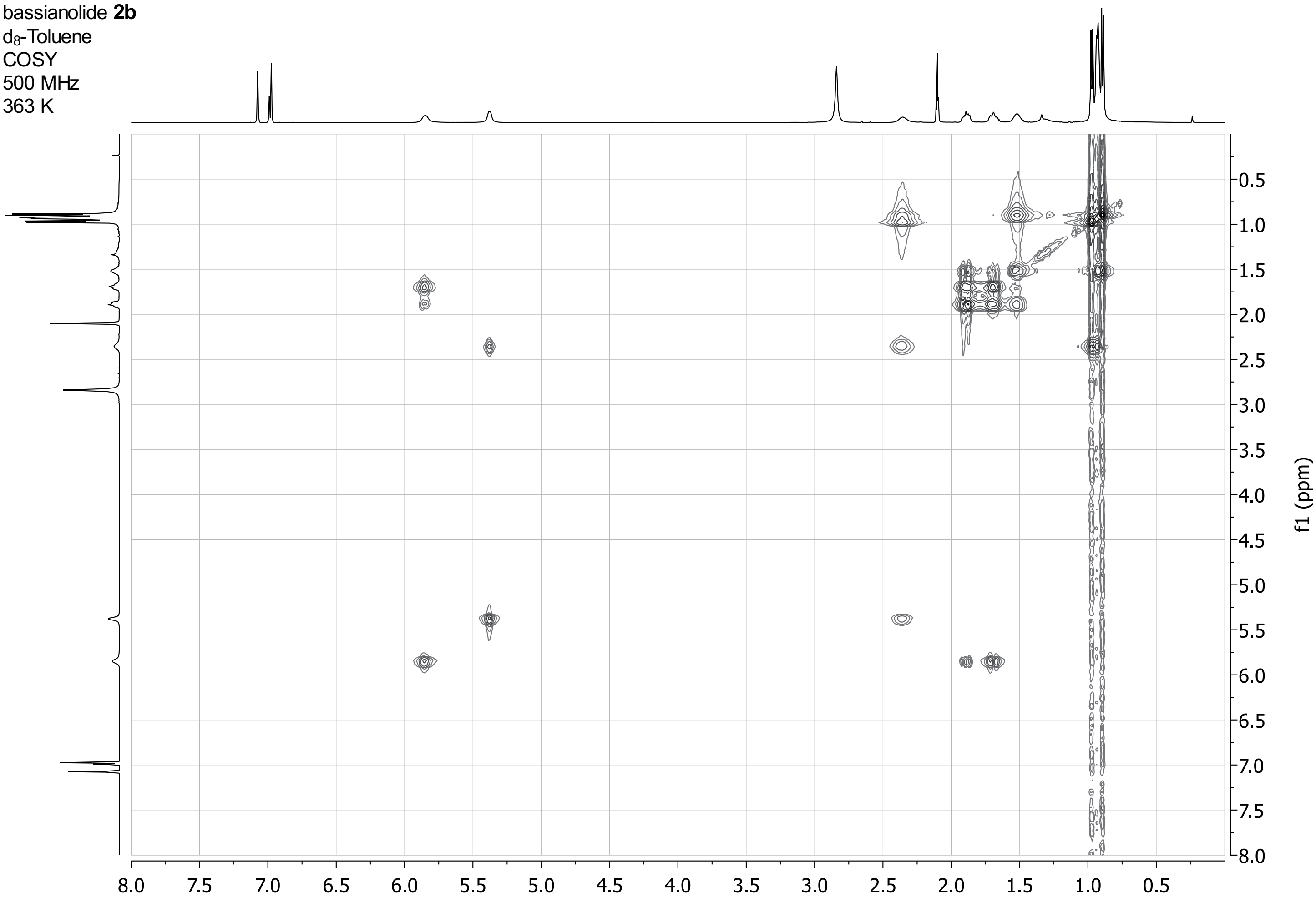
bassianolide **2b**
d_8-Toluene
COSY
500 MHz
363 K
f2 (ppm)
f1 (ppm)

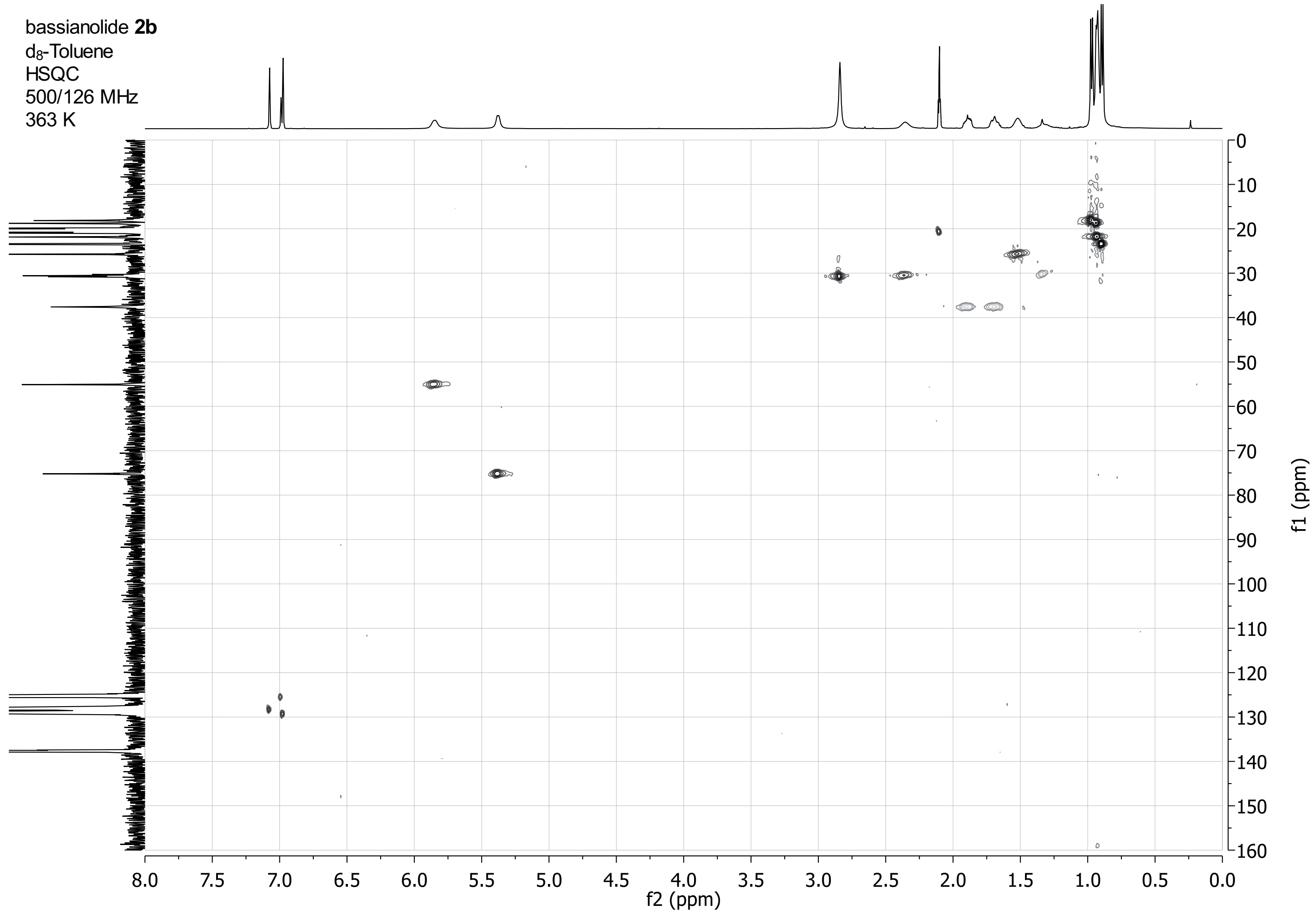
bassianolide **2b**
d_8-Toluene
HSQC
500/126 MHz
363 K
f2 (ppm)
f1 (ppm)

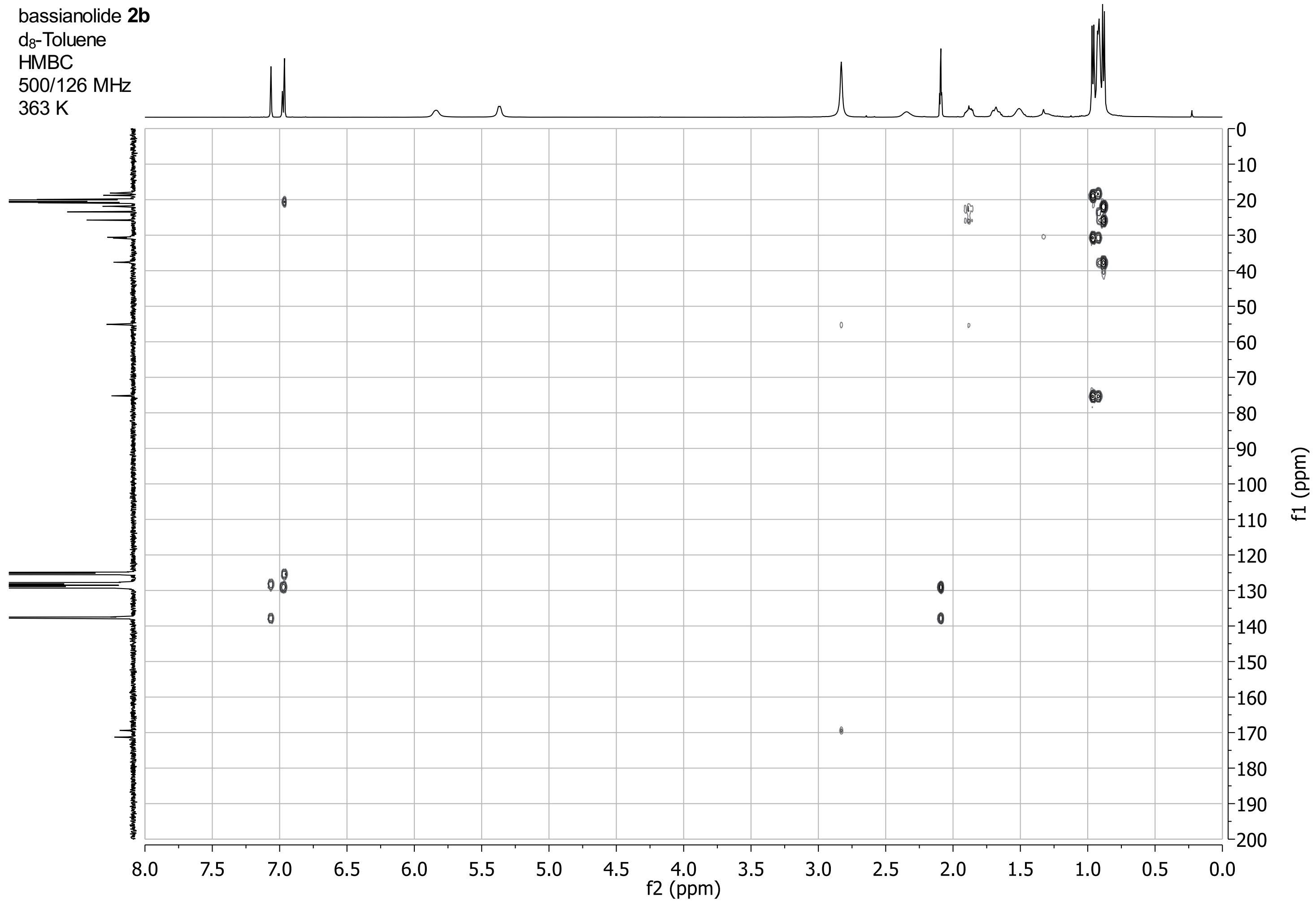
bassianolide **2b**
d_8-Toluene
HMBC
500/126 MHz
363 K
f2 (ppm)
f1 (ppm)
